# Supplementary material for: Maternal prenatal anxiety and depression and trajectories of cardiometabolic risk factors across childhood and adolescence: a prospective cohort study
Source: BMJ Open. 2021 Dec 15;11(12):e051681. doi: 10.1136/bmjopen-2021-051681 (PMC8679111; doi:10.1136/bmjopen-2021-051681)
Supplement: Author's manuscript [file bmjopen-2021-051681.draft_revisions.pdf]

BMJ Open is committed to open peer review. As part of this commitment we make the peer review history of every article we publish publicly available.

When an article is published we post the peer reviewers' comments and the authors' responses online. We also post the versions of the paper that were used during peer review. These are the versions that the peer review comments apply to.

The versions of the paper that follow are the versions that were submitted during the peer review process. They are not the versions of record or the final published versions. They should not be cited or distributed as the published version of this manuscript.

BMJ Open is an open access journal and the full, final, typeset and author-corrected version of record of the manuscript is available on our site with no access controls, subscription charges or pay-per-view fees (http://bmjopen.bmj.com).

If you have any questions on BMJ Open's open peer review process please email info.bmjopen@bmj.com

Maternal prenatal anxiety and depression and trajectories of cardiometabolic risk factors across childhood and adolescence: a prospective cohort study

Journal:	*BMJ Open*
Manuscript ID	bmjopen-2021-051681
Article Type:	Original research
Date Submitted by the Author:	27-Mar-2021
Complete List of Authors:	Matvienko-Sikar, Karen; University College Cork<br>O Neill, Kate; University College Cork<br>Fraser, Abigail; University of Bristol, Population Health Sciences<br>Hayes, Catherine; Trinity College Dublin<br>Howe, Laura; University of Bristol, Population Health Sciences<br>Huizink, A. C.; VU University Amsterdam, Department of Clinical, Neuro<br>Kearney, Patricia; University College Cork<br>Khashan, Ali; University College Cork, School of Public Health; Irish Centre for Maternal and Child Health Research (INFANT) Centre, Cork University Maternity Hospital, Cork,<br>Redsell, Sarah A.; University of Nottingham<br>O'Keeffe, Linda M; University College Cork, School of Public Health; MRC Integrative Epidemiology Unit at the University of Bristol, Population Health Sciences
Keywords:	Anxiety disorders < PSYCHIATRY, Depression & mood disorders < PSYCHIATRY, Cardiac Epidemiology < CARDIOLOGY, Prenatal diagnosis < OBSTETRICS, EPIDEMIOLOGY

SCHOLARONE™
Manuscripts

I, the Submitting Author has the right to grant and does grant on behalf of all authors of the Work (as defined in the below author licence), an exclusive licence and/or a non-exclusive licence for contributions from authors who are: i) UK Crown employees; ii) where BMJ has agreed a CC-BY licence shall apply, and/or iii) in accordance with the terms applicable for US Federal Government officers or employees acting as part of their official duties; on a worldwide, perpetual, irrevocable, royalty-free basis to BMJ Publishing Group Ltd ("BMJ") its licensees and where the relevant Journal is co-owned by BMJ to the co-owners of the Journal, to publish the Work in this journal and any other BMJ products and to exploit all rights, as set out in our licence.

The Submitting Author accepts and understands that any supply made under these terms is made by BMJ to the Submitting Author unless you are acting as an employee on behalf of your employer or a postgraduate student of an affiliated institution which is paying any applicable article publishing charge ("APC") for Open Access articles. Where the Submitting Author wishes to make the Work available on an Open Access basis (and intends to pay the relevant APC), the terms of reuse of such Open Access shall be governed by a Creative Commons licence – details of these licences and which Creative Commons licence will apply to this Work are set out in our licence referred to above.

Other than as permitted in any relevant BMJ Author's Self Archiving Policies, I confirm this Work has not been accepted for publication elsewhere, is not being considered for publication elsewhere and does not duplicate material already published. I confirm all authors consent to publication of this Work and authorise the granting of this licence.

For peer review only

Maternal prenatal anxiety and depression and trajectories of cardiometabolic risk factors across childhood and adolescence: a prospective cohort study

Karen Matvienko-Sikar, School of Public Health, University College Cork, Ireland, Karen.msikar@ucc.ie

Kate N O' Neill, School of Public Health, University College Cork, Ireland, kate.oneill@ucc.ie

Abigail Fraser, Population Health Sciences, Bristol Medical School, University of Bristol, Oakfield House, Oakfield Grove, Bristol, UK, BS82BN, Abigail.fraser@bristol.ac.uk

Catherine Hayes, School of Medicine, Trinity College Dublin, Dublin, Ireland, Hayesc9@tcd.ie

Laura D Howe, Population Health Sciences, Bristol Medical School, University of Bristol, Oakfield House, Oakfield Grove, Bristol, UK, BS82BN, laura.howe@bristol.ac.uk

Anja C Huizink, Department of Clinical, Neuro- and Developmental Psychology, VU University Amsterdam, Amsterdam, The Netherlands, a.c.huizink@vu.nl

Patricia M Kearney, School of Public Health, University College Cork, Ireland, patricia.kearney@ucc.ie

Ali Khashan, Irish Centre for Maternal and Child Health Research (INFANT) Centre, Cork University Maternity Hospital, Cork, T12 YE02, Ireland; School of Public Health, University College Cork, Ireland, a.khashan@ucc.ie

Sarah Redsell, University of Nottingham, University Park, Nottingham, UK, NG7 2RD, sarah.redsell@nottingham.ac.uk

Linda M O'Keeffe[1, 2, 7], School of Public Health, University College Cork, Ireland; Population Health Sciences, Bristol Medical School, University of Bristol, Oakfield House, Oakfield Grove, Bristol, UK, BS82BN; MRC Integrative Epidemiology Unit at the University of Bristol, Oakfield House, Oakfield Grove, Bristol, UK, BS82BN, linda.okeeffe@ucc.ie

Corresponding author: Dr Karen Matvienko-Sikar, School of Public Health, 4th Floor Western Gateway Building, University College Cork, Ireland. karen.msikar@ucc.ie ,00353214205530

Word count: 3091

For peer review only

Abstract

Objectives: Quantifying long-term offspring cardiometabolic health risks associated with maternal prenatal anxiety and depression can guide cardiometabolic risk prevention. This study examines associations between maternal prenatal anxiety and depression, and offspring cardiometabolic risk from birth to 18 years.

Design. This study uses data from the Avon Longitudinal Study of Parents and Children (ALSPAC) cohort.

Participants. Participants were 526-8,606 mother-offspring pairs from the ALSPAC cohort.

Setting. British birth cohort set, Bristol, UK.

Primary and secondary outcomes. Exposures were anxiety (Crown-Crisp Inventory score) and depression (Edinburgh Postnatal Depression Scale score) measured at 18 and 32 weeks gestation. Outcomes were trajectories of offspring body mass index; fat mass; lean mass; pulse rate; glucose, diastolic and systolic blood pressure; triglycerides, high-density lipoprotein cholesterol and non-high-density lipoprotein cholesterol, and insulin from birth/early childhood to 18 years. Exposures were analysed categorically using clinically relevant, cut-offs and continuously to examine associations across the distribution of prenatal anxiety and depression.

Results. We found no strong evidence of associations between maternal anxiety and depression, and offspring trajectories of any cardiometabolic risk factors, except for small, inconsistent associations with fat mass trajectories that attenuated upon confounder

For peer review only - http://bmjopen.bmj.com/site/about/guidelines.xhtml

adjustment. For instance, in unadjusted analyses, anxiety at both 18 and 32 weeks was associated with a 1.8% (95% Confidence Interval (CI), 0.29,3.33) higher mean BMI, which spanned the null (difference (95% CI): 0.7% (-0.76,2.13) after adjustment for confounders.

Conclusions. This is the first examination of maternal prenatal anxiety and depression and trajectories of offspring cardiometabolic risk. Our findings suggest that prevention of maternal prenatal anxiety and depression may have limited impact on offspring cardiometabolic health across the first two decades of life.

Keywords: Anxiety; Depression; Cardiomatabolic Risk; Pregnancy: Child Trajectories

Article Summary

Strengths and limitations

- This study presents a prospective measurement of anxiety and depression twice during pregnancy, including analysis of repeated measures of 11 key cardiometabolic risk factors from birth to 18 years;
- Use of multilevel models accounting for clustering of repeated measures within individuals, and correlation between measures over time.
- Included participants tend to be predominantly white women, and were more advantaged than those excluded due to missing exposure, confounder or outcome data, limiting generalizability.
- Anxiety and depression exposures were self-reported.

For peer review only

Introduction

Maternal prenatal anxiety and depression are estimated to affect up to 25% of women[1]. Maternal prenatal anxiety and depression can result from a range of general and pregnancy-specific factors[2]. Maternal prenatal anxiety and depression are associated with adverse offspring outcomes such as low birth weight, short gestational age[3], obstetric complications[4], and poor offspring developmental and health outcomes[5,6]. Adverse offspring outcomes may result from intrauterine programming involving endocrine, inflammatory and immunological processes[6–8]. Epigenetic changes[8] and dysregulation of the maternal hypothalamic pituitary adrenal (HPA) axis are suggested to result from prenatal distress[9]; dysregulated prenatal HPA axis activity may programme foetal HPA axis activity, reactivity and later health outcomes[6,9]. It is recognised that there may be 'critical windows' of vulnerability during which exposure to prenatal anxiety and depression are particularly deleterious to offspring health[10,11]. However, chronic anxiety and/or depression (experienced over a prolonged period) during pregnancy, may also lead to adverse offspring outcomes[7]. It is further argued that such pathways, as well as behavioural and environmental mechanisms, increase risk of unfavourable offspring cardiometabolic health outcomes, including increased adiposity and poor cardiovascular function[6,12–14].

To date, inconsistent associations have been observed between maternal prenatal anxiety and depression, and child and adolescent cardiometabolic risk factors including high blood pressure[12,15], increased insulin resistance[12,14] and overweight[13,16–19]. Little is known about effects of timing of prenatal maternal anxiety and depression exposures on offspring cardiometabolic outcomes, though there is some evidence that later exposures may confer increased risk[13]. There is some limited evidence for differential associations by 'type' of exposure, such as type of psychological distress exposure (e.g. stress, anxiety and

For peer review only

depression), particularly for anthropometric outcomes[14], though this is not well understood. Studies examining associations of prenatal anxiety and depression with cardiometabolic health outcomes have predominantly examined associations with outcomes at a single time point [e.g.] [12,14]. Examining early-life trajectories of cardiometabolic health outcomes provides insights into if and when associations emerge during childhood and adolescence, and whether associations persist over time. This is particularly important as cardiovascular risk originates early in life and can track to adulthood[20]. Such examinations are needed to highlight potential mechanisms, and inform the nature and timing of prevention efforts for both maternal prenatal anxiety and depression, and offspring cardiometabolic risk.

The objective of this study is to examine associations of maternal anxiety and depression with offspring cardiometabolic health outcome trajectories from birth to 18 years using data from the Avon Longitudinal Study of Parents and Children (ALSPAC).

For peer review only

Methods

Study participants

ALSPAC is a prospective birth cohort study in Southwest England[20,21]. Pregnant women resident in one of the three Bristol-based health districts with an expected delivery date between April 1, 1991 and December 31, 1992 were invited to participate. The study has been described elsewhere in detail[20,21]. ALSPAC initially enrolled a cohort of 14,451 pregnancies, from which 13,761 women provided informed consent and had 13,867 live births. Research clinics were held when the offspring were approximately 7, 9, 10, 11, 13, 15, and 18 years old. Ethical approval for the study was obtained from the ALSPAC Ethics and Law Committee and the Local Research Ethics Committees; http://www.bristol.ac.uk/alspac/researchers/research-ethics/. The study website contains details of all the data that are available through a fully searchable data dictionary http://www.bristol.ac.uk/alspac/researchers/our-data/.

Study exposures

We derived and separately analysed associations between different indices of maternal prenatal anxiety and depression, and trajectories of cardiometabolic health outcomes.

Maternal self-report prenatal anxiety

Maternal prenatal anxiety was measured at 18 and 32 weeks gestation using the eight items from the anxiety subscale of Crown Crisp Experiential Index (CCEI[22]); further details in Supplementary methods S1. For our primary analysis, a score ≥85th percentile (≥85th percentile = 8) was used to define anxiety[6]. We then categorised prenatal anxiety as ‘anxiety at 18 weeks’, ‘anxiety at 32 weeks’, ‘anxiety at both time points’, and ‘anxiety at neither time point’ (reference group). In secondary analyses, anxiety was analysed as a continuous

For peer review only - http://bmjopen.bmj.com/site/about/guidelines.xhtml

measure at 18 and 32 weeks separately and taking the mean of anxiety scores at 18 and 32 weeks to explore associations with offspring cardiometabolic health across the entire distribution (to examine associations at preclinical and clinical levels).

Maternal self-report depression

Maternal prenatal depression was measured at 18 and 32 weeks gestation using the Edinburgh Postnatal Depression Scale (EPDS[23]); further details in Supplementary methods S1. For our primary analysis, a score of ≥13 was used to define clinical depression[20]. We categorised maternal prenatal depression as 'depression at 18 weeks', 'depression at 32 weeks', 'depression at both time points', and 'depression at neither time point' (reference group). The continuous measure of depression using the EPDS was also examined. In secondary analyses, depression was analysed as a continuous measure at 18 and 32 weeks separately and taking the mean of depression scores at 18 and 32 weeks to explore associations with offspring cardiometabolic health across the entire distribution (to examine associations at preclinical and clinical levels).

Study outcomes

Anthropometry

Body Mass Index (BMI: weight (kg) divided by height squared (m^2)) was calculated from 1 to 18 years using data from research clinics, routine offspring health clinics, health visitor records and parent-reported questionnaires. Whole body less head, and central fat and lean mass were derived from whole body dual energy X-ray absorptiometry (DEXA) scans assessed 5 times at ages 9, 11, 13, 15, and 18 using a Lunar prodigy narrow fan beam densitometer.

For peer review only - http://bmjopen.bmj.com/site/about/guidelines.xhtml

Systolic Blood Pressure (SBP), Diastolic Blood Pressure (DBP) and pulse rate

At each clinic (ages 7, 9, 10, 11, 13, 15 and 18) offspring SBP, DBP and pulse rate were measured at least twice. Measures were taken when the offspring was sitting and at rest with the arm supported, using a validated device and a cuff size appropriate for upper arm circumference. The mean of the two final measures was used here.

Blood based biomarkers

Insulin was measured from cord blood at birth and from research clinic samples at age 9, 15 and 18 years. Non-fasting glucose was available at age 7; fasting glucose was available at age 9, 15 and 18 years.

Triglycerides, high-density lipoprotein cholesterol (HDL-c) and total cholesterol were measured in cord blood at birth and from venous blood subsequently. Samples were non-fasted at 7 and 9 years; fasting measures were available from clinics at 15 and 18 years. Non-HDL-c was calculated by subtracting HDL-c from total cholesterol at each measurement occasion. Trajectories of blood-based biomarkers are thus a combination of measures from cord blood, fasting bloods and non-fasting bloods, with most measures obtained through standard clinical chemistry assays.

Further details on measurement sources for outcomes are available in Supplementary methods S2.

Covariates

We considered the following as potential confounders: household social class, parity, and maternal age, education, smoking during pregnancy, pre-pregnancy BMI, all measured by

For peer review only

198 mother- or mother's partner-completed questionnaires; details in Supplementary methods S3.
199 Offspring birth weight is a potential mediator of associations of prenatal anxiety and
200 depression with later child cardiometabolic outcomes[3,6]and was not adjusted for in our
201 analyses.

202

203 Statistical analysis

204 Sex-specific patterns of change in each risk factor have been modelled previously using
205 multilevel model[24,25] and were used here as outcome trajectories. Multilevel models
206 estimate mean trajectories of the outcome while accounting for the non-independence (i.e.
207 clustering) of repeated measurements within individuals, change in scale and variance of
208 measures over time, and differences in the number and timing of measurements between
209 individuals. Models include all available data from all eligible participants under a Missing at
210 Random (MAR) assumption.

211 Trajectories of BMI were modelled using fractional polynomials[26]; all other risk factors
212 were estimated using linear splines (two levels for all models: measurement occasion and
213 individual). Fractional polynomial terms and linear spline periods were selected based on
214 model fit statistics and examination of observed data. Lean mass and fat mass included three
215 periods of change; from 9 to 13, 13 to 15, 15 to 18; SBP, DBP and pulse rate included three
216 periods of change from 7 to 12, 12 to 16, 16 to 18; HDL-c included two periods of change
217 from 0 to 7, 7 to 18; non-HDL-c and triglycerides included two periods of change from 0 to
218 9, 9 to 18; insulin included three periods of change, from 0 to 9, 9 to 15, 15 to 18; glucose
219 included two periods of change from 7-15 and 15 to 18. Further information on the modelling
220 of these trajectories is described in Supplementary methods S4 and Supplementary table Ss 1
221 to 7.

For peer review only - http://bmjopen.bmj.com/site/about/guidelines.xhtml

Association between maternal prenatal anxiety and depression and trajectories of offspring cardiometabolic health outcomes

Associations between maternal prenatal anxiety and depression, and trajectories of cardiometabolic risk factors were examined by including an interaction between the categories of each exposure (primary analyses) or mean scores for continuous analyses (secondary analyses) and fractional polynomial age terms or linear spline periods. Based on previous modelling of outcomes, the mean outcome trajectory for each risk factor was allowed to vary by sex. We also explored whether associations between each prenatal maternal anxiety and depression exposure differed between females and males by including an interaction term between each exposure and sex. These analyses demonstrated no strong evidence of a sex interaction in the association of prenatal maternal anxiety and depression and cardiometabolic health outcome trajectories; thus all analyses were subsequently performed and presented sex-combined. We performed unadjusted and confounder adjusted analyses for all models.

Values of cardiometabolic risk factors that had a skewed distribution (BMI, fat mass, insulin and triglyceride) were (natural) log transformed prior to analysis; differences between each exposure category and the reference group from these models are calculated on the log scale. These values were then back transformed and are interpreted as the ratio of geometric means. Fat mass and lean mass were adjusted for height using the time-and sex-varying power of height that best resulted in a height-invariant measure[20]. All trajectories were modelled in MLwiN version 3.04, using the runmlwin command in Stata version 16.

For peer review only - http://bmjopen.bmj.com/site/about/guidelines.xhtml

Participants and measures included in analyses

Participants with measures of maternal prenatal anxiety and depression and at least one measure of a risk factor, and complete data on all confounders were included in analyses. Offspring that reported being pregnant at the 18-year clinic (n=6) were excluded from analyses at that time point only. Supplementary figure S 1 shows a flow diagram for the study.

Patient and public Involvement

This was a secondary analysis of data from the ALSPAC birth cohort and did not involve patient and public involvement.

For peer review only

Results

Supplementary table S8 shows the number of offspring with available measures of cardiometabolic risk factors at each age. The number of mother–offspring pairs included in analyses ranged from 526 participants (1,464 repeated measures) for analyses of insulin to 8,606 (80,796 repeated measures) for analyses of BMI. Prevalence of anxiety was 7.5% (n=651) at 18 weeks only, 9% (n=792) at 32 weeks only, and 12% (n=1026) at both 18 and 32 weeks. Prevalence of probable clinical depression was 5.5% (n=474) at 18 weeks only; 7.1% (n=610) at 32 weeks only; 6.3% (n=542) at both 18 and 32 weeks. Table 1 shows characteristics of participants by maternal prenatal anxiety levels (note, patterns of participant characteristics were similar for categories of prenatal maternal depression and are not shown here). Women with anxiety at any point during pregnancy (n=2469, 29%) were more likely to have lower education, and to smoke during pregnancy. Mother-offspring pairs included in analyses of BMI (n=8,606 and maximum available sample size of all analyses) tended to have higher education and lower levels of smoking during pregnancy compared to those excluded due to missing exposure, outcome or confounder data (n=5,261 to 13,341) (Supplementary table S9).

For peer review only

Table 1 Characteristics of ALSPAC participants included in analysis, by maternal anxiety levels during pregnancy

	No anxiety during pregnancy n=6,137	Anxiety at 18 weeks gestation only n=651	Anxiety at 32 weeks gestation only n=792	Anxiety at 18 & 32 weeks gestation n=1026
	**n (%)**	**n (%)**	**n (%)**	**n (%)**
**Household social class †**				
Professional	936(15.3)	74(11.4)	87(11.0)	93(9.1)
Managerial & Technical	2673(43.6)	282(43.3)	310(39.1)	408(39.8)
Non-Manual	1574(25.6)	174(26.7)	222(28.0)	284(27.7)
Manual	677(11.0)	86(13.2)	107(13.5)	167(16.3)
Part Skilled & Unskilled	277(4.5)	35(5.4)	66(8.3)	74(7.2)
**Maternal education**				
Less than O level	1410(23.0)	152(23.3)	249(31.4)	313(30.5)
O level*	2228(36.3)	257(39.5)	273(34.5)	409(39.9)
A level	1549(25.2)	167(25.7)	170(21.5)	207(20.2)
Degree or above	950(15.5)	75(11.5)	100(12.6)	97(9.5)
**Mother's Partner's highest educational qualification**				
Less than O level	1675(28.0)	189(30.0)	272(35.9)	352(36.1)
O level*	1320(22.1)	151(24.0)	176(23.2)	225(23.1)
A level	1711(28.6)	167(26.6)	196(25.9)	263(26.9)
Degree or Above	1271(21.3)	122(19.4)	114(15.0)	136(13.9)
**Maternal smoking during pregnancy**				
No	5040(82.1)	480(73.7)	587(74.1)	674(65.7)
Yes	1097(17.9)	171(26.3)	205(25.9)	352(34.3)
**Parity**				
0	2795(45.5)	319(49.0)	352(44.4)	427(41.6)
1	2286(37.2)	220(33.8)	289(36.5)	364(35.5)
2	1056(17.2)	112(17.2)	151(19.1)	235(22.9)
	**Mean (SD)**	**Mean (SD)**	**Mean (SD)**	**Mean (SD)**
**Gestational age (weeks)**	39.5(1.8)	39(1.7)	39(1.6)	39(1.8)
**Birth weight (g)**	3431(535)	3395(527)	3417(500)	3423(546)
**Pre-pregnancy BMI (kg/m^2)**	22.9(3.7)	23(4.2)	23(3.8)	23(4.0)
**Maternal age (years)**	29.0(4.5)	28(4.9)	28(5.0)	28(4.9)

Number of participants available for analyses of BMI (n=8,606) used as the denominator in this table given the varying sample sizes included in analyses. † Household social class was measured as the highest of the mother's or her partner's occupational social class using data on job title and details of occupation collected about the mother and her partner from the mother's questionnaire at 32 weeks gestation. Social class was derived using the standard occupational classification (SOC) codes developed by the United Kingdom Office of Population Census and Surveys and classified as I professional, II managerial and technical, IIINM non-manual, IIIM manual, and IV&V part skilled occupations and unskilled occupations

For peer review only

* O levels equate to current GCSEs demonstrating education level at aged 16.

Anxiety during pregnancy

In unadjusted analyses, anxiety at both 18 and 32 weeks was associated with a 1.8% (95% Confidence Interval (CI), 0.29,3.33) higher mean BMI (Supplementary table S10) and 6.33% (95% CI, 0.86, 11.79) higher fat mass at age 18 years (Supplementary table S11) compared with no anxiety at either time point. In confounder-adjusted analyses, associations with BMI and fat mass attenuated (Figure 1, Supplementary table S10 and S11). For instance, the difference in fat mass at 18 years between offspring of women who experienced anxiety at 18 and 32 weeks and those who did not was 4.77% (95% CI, -0.51, 10.05).

In unadjusted analyses, anxiety at 18 weeks only was associated with higher SBP and pulse rate at 18 years (Supplementary table S12) compared with no anxiety at either time point. Anxiety at 18 weeks only was also associated with higher DBP at 7 years; this difference persisted at age 18 years (difference at 18 years; 0.97 mmHg (95% CI, 0.13, 1.81). Anxiety at 32 weeks only was associated with 0.84 mmHg (95% CI, 0.05, 1.64) higher DBP at age 18. Findings were similar in confounder-adjusted analyses (Figure 2 and Supplementary table S12).

In unadjusted and confounder-adjusted analyses, we found no strong evidence that prenatal anxiety was associated with trajectories of lean mass from 9 to 18 years (Figure 1 and Supplementary table S13), glucose from 7 to 18 years (Figure 3 and Supplementary table S 14) and insulin (Figure 3 and Supplementary table S15), triglyceride (Figure 4 and Supplementary table S15), HDL-c and non-HDL-c (Figure 4 and Supplementary table S16), all from birth to 18 years.

For peer review only - http://bmjopen.bmj.com/site/about/guidelines.xhtml

Continuous analyses

Results for all analyses were similar when prenatal maternal anxiety was examined as a continuous exposure at each time point separately or when the mean of the two measures was examined (see Supplementary figure S2- 12).

Depression during pregnancy

In unadjusted analyses, depression at both 18 and 32 weeks gestation was associated with 2.7% (95% CI, 0.71,4.78) higher BMI and 6.61% (-0.74,13.96) higher fat mass at 18 years (Supplementary table S17 and S18) compared with no depression at either time point. These associations attenuated in adjusted analyses (Supplementary figure S13, Supplementary table S17 and S18); for instance, the difference in fat mass at 18 years for offspring of women who experienced depression at 18 and 32 weeks was 2.32% (95% CI, -4.58, 9.22). Depression at 18 weeks only was associated with higher SBP at age 18 in both unadjusted and confounder adjusted analyses (Supplementary figure S14 and Supplementary table S19). In unadjusted and confounder-adjusted analyses, we found no strong evidence that depression during pregnancy was associated with trajectories of DBP and pulse rate from 7 to 18 (Supplementary figure S14 and Supplementary table S19), lean mass from 9 to 18 years (Supplementary figure S13 and Supplementary table S20), glucose from 7 to 18 years (Supplementary figure S15 and Supplementary table S21), insulin (Supplementary figure S15 and Supplementary table S22), triglyceride (Supplementary figure S16 and Supplementary table S24), HDL-c and non-HDL-c (Supplementary figure S16 and Supplementary table S23), all from birth to 18 years.

For peer review only - http://bmjopen.bmj.com/site/about/guidelines.xhtml

Continuous analyses

Results for all analyses were similar when prenatal maternal depression was examined as a continuous exposure at each time point separately or when the mean of the two measures was examined (see Supplementary figures S17-27).

[Figures 1 to 4]

For peer review only

Discussion

In this large, contemporary prospective birth cohort study with repeated assessment of exposures and outcomes, we found no strong evidence that maternal prenatal anxiety and depression during pregnancy were associated with offspring cardiometabolic risk factors from birth to 18 years, regardless of whether exposure occurred at 18 weeks or 32 weeks gestation or both.

Though unadjusted analyses indicated associations between prenatal maternal anxiety and depression and offspring anthropometric outcomes, these were attenuated when confounders were adjusted for. Our findings differ from previous research suggesting maternal prenatal anxiety and depression adversely impact offspring health and developmental outcomes[12,13,27] . A recent examination of the impact of mid-pregnancy maternal prenatal depression and anxiety identified associations with higher triglycerides in females and higher pulse rate in males at 10 years[14]. However, similar to our study, this study did not find associations between maternal prenatal depression and anxiety and offspring blood pressure, glucose, insulin, or serum lipids[14]. Similarly, a number of prospective cohort studies have reported a lack of evidence to support associations between anxiety and depression and blood pressure[15], adiposity[17–19] and glucose and insulin resistance[12]; such studies examined cardiometabolic risk factors at static time points only however.

Lack of observed associations in the current study may be because in-utero maternal anxiety and depression exposure may be too early to impact on offspring cardiometabolic trajectories over the long term[13]. A recent review of associations between maternal stress and child weight outcomes found that later childhood exposure was more strongly associated with

For peer review only - http://bmjopen.bmj.com/site/about/guidelines.xhtml

offspring weight than exposure in infancy[28]. Thus we may not have observed associations here because maternal prenatal anxiety and depression confers little impact on offspring cardiometabolic health across the early life course, irrespective of gestational timing of exposure. However, given our identification of some associations with offspring fat mass in this study, future work is needed to further examine effects of maternal prenatal anxiety and depression on offspring cardiometabolic health in the first two decades of life.

Strengths and Limitations

Strengths of this study include: prospective measurement of anxiety and depression twice during pregnancy; analysis of repeated measures of 11 key cardiometabolic risk factors from birth to 18 years; use of multilevel models accounting for clustering of repeated measures within individuals, and correlation between measures over time. Examination of anxiety and depression as both categorical and continuous exposures is a further strength, which enabled examination of exposures in terms of clinical cut-offs, and incremental increases in exposure levels across the entire distribution of anxiety and depression; this is important because exposure to subclinical levels my still result in adverse outcomes that would be missed if data were examined categorically only. Limitations include generalisability of findings, as included participants tend to be more advantaged than those excluded due to missing exposure, confounder or outcome data. The range in participant numbers between analyses due to participant follow-up may represent selection bias and further impact generalisability of findings. In addition, ALSPAC includes a high proportion (~98%) white women, limiting representativeness of the findings for non-White ethnicities and ability to perform subgroup analyses for different ethnicity groups. Similarly, ALSPAC included live births only; women experiencing acute and/or chronic distress may have experienced spontaneous abortion, leading to live birth bias[29]. Self-reporting of prenatal anxiety and depression is a further

For peer review only

limitation because self-reports do not tend to correlate well with psychophysiological indicators that could program risk[30].

Conclusion

Our findings suggest that maternal prenatal anxiety and depression do not impact offspring cardiometabolic health outcomes in the first two decades of life; these findings may provide reassurance to women experiencing prenatal anxiety and/or depression that any impacts on offspring cardiometabolic health from birth to the end of adolescence are likely to be small. Approaches and strategies to prevent and/or reduce maternal prenatal anxiety and depression may have limited impact on offspring cardiometabolic health in the first two decades of life.

Figure Legends

Figure 1 Mean predicted confounder adjusted trajectories of lean mass (9 to 18 years), log fat mass (9 to 18 years) and log BMI (1 to 18 years), by maternal anxiety levels during pregnancy

Figure 2 Mean predicted confounder adjusted trajectories of pulse rate, SBP and DBP from 7 to 18 years, by maternal anxiety levels during pregnancy

Figure 3 Mean predicted confounder adjusted trajectories of HDL-c, log triglyceride and non-HDL-c from birth to 18 years, by maternal anxiety levels during pregnancy

Figure 4 Mean predicted confounder adjusted trajectories of log insulin (birth to 18 years) and glucose (7 to 18 years), by maternal anxiety levels during pregnancy

For peer review only

Funding Statement

The UK Medical Research Council and Wellcome (Grant ref: 217065/Z/19/Z) and the University of Bristol provide core support for ALSPAC. This publication is the work of the authors and KMS and LMOK will serve as guarantors for the contents of this paper. KMS is supported by a Health Research Board of Ireland (HRB) Applying Research to Policy and Practice award (HBR-ARPP- A011). LMOK and KON are supported by a HRB Emerging Investigator Award (EIA-2019-007). KON is also supported by a HRB Research Leader Award (RL/2013/7) and a HRB Interdisciplinary Capacity Enhancement Award (HRB ICE 2015-1026). LDH and AF are supported by Career Development Awards from the UK Medical Research Council (grants MR/M020894/1 and MR/M009351/1, respectively). LDH and AF work in a unit that receives funds from the UK Medical Research Council (grant MC_UU_00011/3, MC_UU_00011/6). The research funders had no role in the study design; data collection, analysis, and interpretation of data; writing of the manuscript; or the decision to submit the manuscript for publication.

Competing interests statement

None of the authors have any conflicts of interest to declare.

Data availability

Data used in this study was from the Avon Longitudinal Study of Parents and Children (ALSPAC), requests for access to this data can be made to the ALSPAC executive committee.

For peer review only - http://bmjopen.bmj.com/site/about/guidelines.xhtml

Author contributions

KMS conceptualised the study, conducted analyses, drafted and revised the manuscript. KON conducted analyses, and critically reviewed and revised the manuscript. AF critically reviewed and revised the manuscript. CH contributed to interpretation of findings and critically reviewed and revised the manuscript. LDH critically reviewed and revised the manuscript. ACH contributed to interpretation of findings and critically reviewed and revised the manuscript. PMK contributed to interpretation of findings and critically reviewed and revised the manuscript. AK contributed to interpretation of findings and critically reviewed and revised the manuscript. SR contributed to interpretation of findings and critically reviewed and revised the manuscript. LOK conceptualised the study, conducted analyses, drafted and revised the manuscript. All authors approved the final manuscript.

Acknowledgements

We are extremely grateful to all the families who took part in this study, the midwives for their help in recruiting them, and the whole ALSPAC team, which includes interviewers, computer and laboratory technicians, clerical workers, research scientists, volunteers, managers, receptionists and nurses. We are also grateful to Dr Darren Dahly in the Health Research Board Clinical Research Facility Cork for feedback on an earlier draft of this manuscript.

For peer review only

References

1. Pearson RM, Carnegie RE, Cree C, Rollings C, Rena-Jones L, Evans J, et al. Prevalence of Prenatal Depression Symptoms Among 2 Generations of Pregnant Mothers: The Avon Longitudinal Study of Parents and Children. JAMA network open. United States: American Medical Association; 2018;1:e180725.

2. Dunkel Schetter C. Psychological Science on Pregnancy: Stress Processes, Biopsychosocial Models, and Emerging Research Issues. Annu Rev Psychol. 2011;62:531–58.

3. Grote NK, Bridge JA, Gavin AR, Melville JL, Iyengar S, Katon WJ. A Meta-analysis of Depression During Pregnancy and the Risk of Preterm Birth, Low Birth Weight, and Intrauterine Growth Restriction. Arch Gen Psychiatry. 2010;67:1012.

4. Alder J, Fink N, Bitzer J, Hösli I, Holzgreve W. Depression and anxiety during pregnancy: A risk factor for obstetric, fetal and neonatal outcome? A critical review of the literature. The Journal of Maternal-Fetal & Neonatal Medicine. 2007;20:189–209.

5. O'Connor TG, Heron J, Golding J, Beveridge M, Glover V. Maternal antenatal anxiety and children's behavioural/emotional problems at 4 years. Br J Psychiatry. 2002;180:502–8.

6. Entringer S. Impact of stress and stress physiology during pregnancy on child metabolic function and obesity risk: Current Opinion in Clinical Nutrition and Metabolic Care. 2013;16:320–7.

7. Wadhwa P, Buss C, Entringer S, Swanson J. Developmental Origins of Health and Disease: Brief History of the Approach and Current Focus on Epigenetic Mechanisms. Semin Reprod Med. 2009;27:358–68.

8. Mulligan C, D'Errico N, Stees J, Hughes D. Methylation changes at *NR3C1* in newborns associate with maternal prenatal stress exposure and newborn birth weight. Epigenetics. 2012;7:853–7.

9. Entringer S, Buss C, Rasmussen JM, Lindsay K, Gillen DL, Cooper DM, et al. Maternal cortisol during pregnancy and infant adiposity: a prospective investigation. The Journal of Clinical Endocrinology & Metabolism. 2016;jc.2016-3025.

10. Van den Bergh BRH, Mulder EJH, Mennes M, Glover V. Antenatal maternal anxiety and stress and the neurobehavioural development of the fetus and child: links and possible mechanisms. A review. Neuroscience & Biobehavioral Reviews. 2005;29:237–58.

11. Glover V. Annual Research Review: Prenatal stress and the origins of psychopathology: an evolutionary perspective: Prenatal stress and the origins of psychopathology. Journal of Child Psychology and Psychiatry. 2011;52:356–67.

12. van Dijk AE, Dawe K, Deanfield J, Stronks K, Gemke RJBJ, Vrijkotte TGM, et al. The association of maternal prenatal psychosocial stress with vascular function in the child at age

For peer review only - http://bmjopen.bmj.com/site/about/guidelines.xhtml

10-11 years: findings from the Avon longitudinal study of parents and children. European journal of preventive cardiology. England: Sage; 2014;21:1097–108.

13. Vehmeijer FOL, C V Silva C, Derks IPM, El Marroun H, Oei EHG, Felix JF, et al. Associations of Maternal Psychological Distress during Pregnancy with Childhood General and Organ Fat Measures. Childhood obesity (Print). United States: Mary Ann Liebert; 2019;15:313–22.

14. Silva CCV, Vehmeijer FOL, El Marroun H, Felix JF, Jaddoe VWV, Santos S. Maternal psychological distress during pregnancy and childhood cardio-metabolic risk factors. Nutrition, metabolism, and cardiovascular diseases : NMCD. Netherlands: Elsevier; 2019;29:572–9.

15. van Dijk AE, van Eijsden M, Stronks K, Gemke RJBJ, Vrijkotte TGM. The association between prenatal psychosocial stress and blood pressure in the child at age 5-7 years. PloS one. United States: Public Library of Science; 2012;7:e43548.

16. Matvienko-Sikar K, Cooney J, Flannery C, Murphy J, Khashan A, Huizink A. Maternal stress in the first 1000 days and risk of childhood obesity: a systematic review. Journal of Reproductive and Infant Psychology. 2020;1–25.

17. Ertel KA, Huang T, Rifas-Shiman SL, Kleinman K, Rich-Edwards J, Oken E, et al. Perinatal weight and risk of prenatal and postpartum depressive symptoms. Annals of epidemiology. United States: Elsevier; 2017;27:695.

18. Braungart-Rieker JM, Lefever JB, Planalp EM, Moore ES. Body Mass Index at 3 Years of Age: Cascading Effects of Prenatal Maternal Depression and Mother-Infant Dynamics. The Journal of pediatrics. United States: Mosby; 2016;177:128.

19. Ingstrup KG, Schou Andersen C, Ajslev TA, Pedersen P, Sørensen TIA, Nohr EA. Maternal Distress during Pregnancy and Offspring Childhood Overweight. Journal of obesity. United States: Hindawi Publishing Corporation; 2012;2012:462845.

20. Fraser, A. O'Keeffe,L.M., Howe, L. Accounting for height in indices of body composition during childhood and adolescence. Wellcome open research. 4.

21. Boyd A, Golding J, Macleod J, Lawlor DA, Fraser A, Henderson J, et al. Cohort Profile: The 'Children of the 90s'—the index offspring of the Avon Longitudinal Study of Parents and Children. International Journal of Epidemiology. 2013;42:111–27.

22. Birtchnell J, Evans C, Kennard J. The total score of the Crown-Crisp Experiential Index: A useful and valid measure of psychoneurotic pathology. British Journal of Medical Psychology. 1988;61:255–66.

23. Cox JL, Holden JM, Sagovsky R. Detection of Postnatal Depression: Development of the 10-item Edinburgh Postnatal Depression Scale. Br J Psychiatry. 1987;150:782–6.

24. O'Keeffe LM, Simpkin AJ, Tilling K, Anderson EL, Hughes AD, Lawlor DA, et al. Sex-specific trajectories of measures of cardiovascular health during childhood and adolescence: A prospective cohort study. Atherosclerosis. 2018;278:190–6.

For peer review only

25. O'Keeffe LM, Simpkin AJ, Tilling K, Anderson EL, Hughes AD, Lawlor DA, et al. Data on trajectories of measures of cardiovascular health in the Avon Longitudinal Study of Parents and Children (ALSPAC). Data in Brief. 2019;23:103687.

26. Royston P, Altman DG. Regression Using Fractional Polynomials of Continuous Covariates: Parsimonious Parametric Modelling. Applied Statistics. 1994;43:429.

27. Fan F, Zou Y, Tian H, Zhang Y, Zhang J, Ma X, et al. Effects of maternal anxiety and depression during pregnancy in Chinese women on children's heart rate and blood pressure response to stress. Journal of human hypertension. England: Nature Publishing Group; 2016;30:171–6.

28. Tate EB, Wood W, Liao Y, Dunton GF. Do stressed mothers have heavier children? A meta-analysis on the relationship between maternal stress and child body mass index: Maternal stress and child obesity. Obes Rev. 2015;16:351–61.

29. Liew Z, Olsen J, Cui X, Ritz B, Arah OA. Bias from conditioning on live birth in pregnancy cohorts: an illustration based on neurodevelopment in children after prenatal exposure to organic pollutants. International Journal of Epidemiology. 2015;44:345–54.

30. Himes K, Simhan H. Plasma Corticotropin-Releasing Hormone and Cortisol Concentrations and Perceived Stress among Pregnant Women with Preterm and Term Birth. Amer J Perinatol. 2011;28:443–8.

For peer review only

For peer review only - http://bmjopen.bmj.com/site/about/guidelines.xhtml

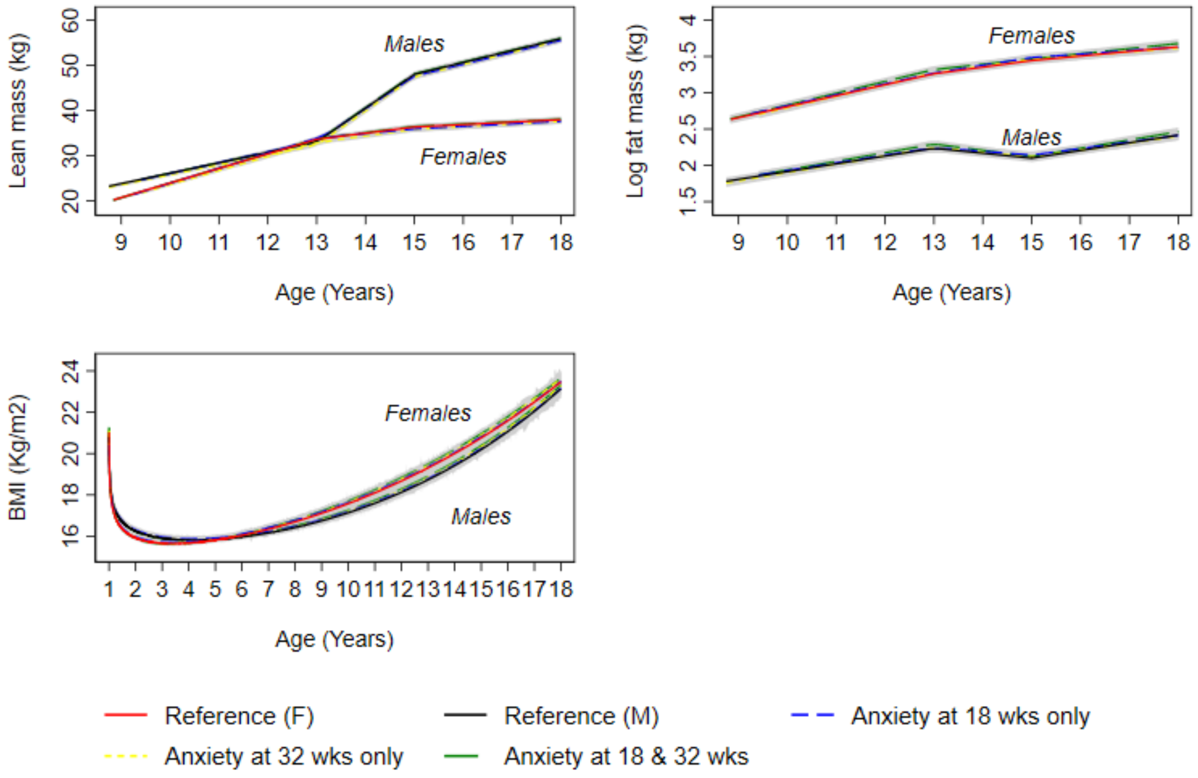

Figure 1 Mean predicted confounder adjusted trajectories of lean mass (9 to 18 years), log fat mass (9 to 18 years) and log BMI (1 to 18 years), by maternal anxiety levels during pregnancy

Legend: Trajectories are adjusted for maternal age at birth of offspring, parental household socioeconomic position, parity, maternal pre-pregnancy BMI and maternal smoking during pregnancy. Anxiety at 18 and 32 weeks gestation is defined as ≥85th percentile of the anxiety subscale of the Crown-Crisp Index CCEI) for the whole cohort at each time point separately (≥85th percentile = 8 at both 18 and 32 weeks gestation).

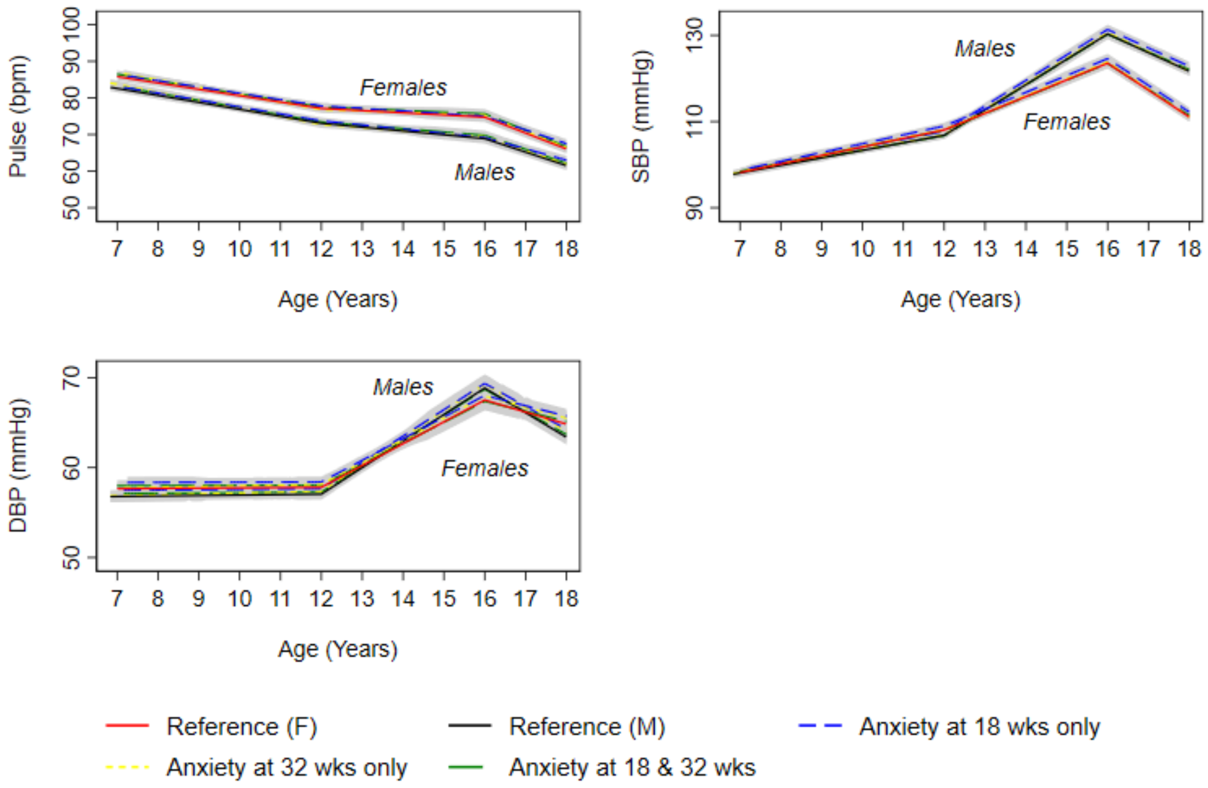

Figure 2 Mean predicted confounder adjusted trajectories of pulse rate, SBP and DBP from 7 to 18 years, by maternal anxiety levels during pregnancy

Legend: DBP, diastolic blood pressure; SBP, systolic blood pressure.
Trajectories are adjusted for maternal age at birth of offspring, parental household socioeconomic position, parity, maternal pre-pregnancy BMI and maternal smoking during pregnancy. Anxiety at 18 and 32 weeks gestation is defined as ≥85th percentile of the anxiety subscale of the Crown-Crisp Index CCEI) for the whole cohort at each time point separately (≥85th percentile = 8 at both 18 and 32 weeks gestation).

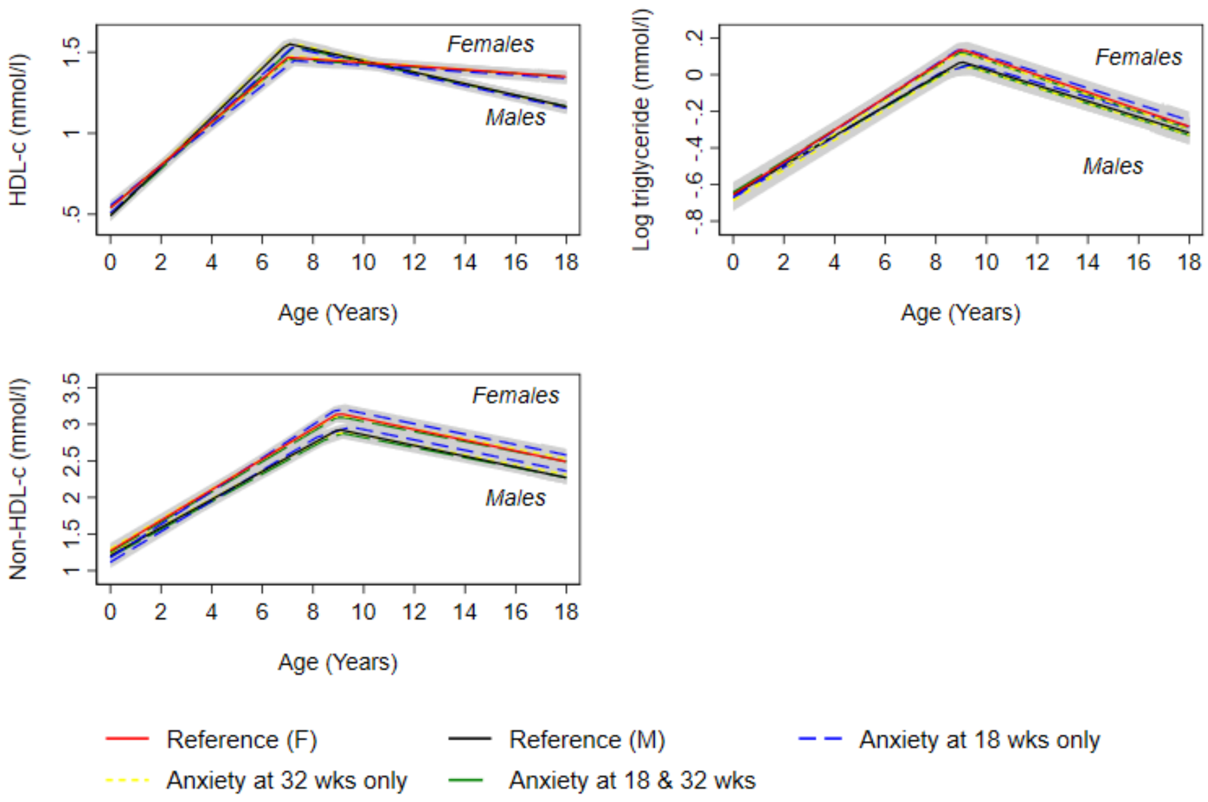

Figure 3 Mean predicted confounder adjusted trajectories of HDL-c, log triglyceride and non-HDL-c from birth to 18 years, by maternal anxiety levels during pregnancy

Legend: HDL-c, high-density lipoprotein-cholesterol; non-HDL-c.
Trajectories are adjusted for maternal age at birth of offspring, parental household socioeconomic position, parity, maternal pre-pregnancy BMI and maternal smoking during pregnancy. Anxiety at 18 and 32 weeks gestation is defined as ≥85th percentile of the anxiety subscale of the Crown-Crisp Index CCEI) for the whole cohort at each time point separately (≥85th percentile = 8 at both 18 and 32 weeks gestation).

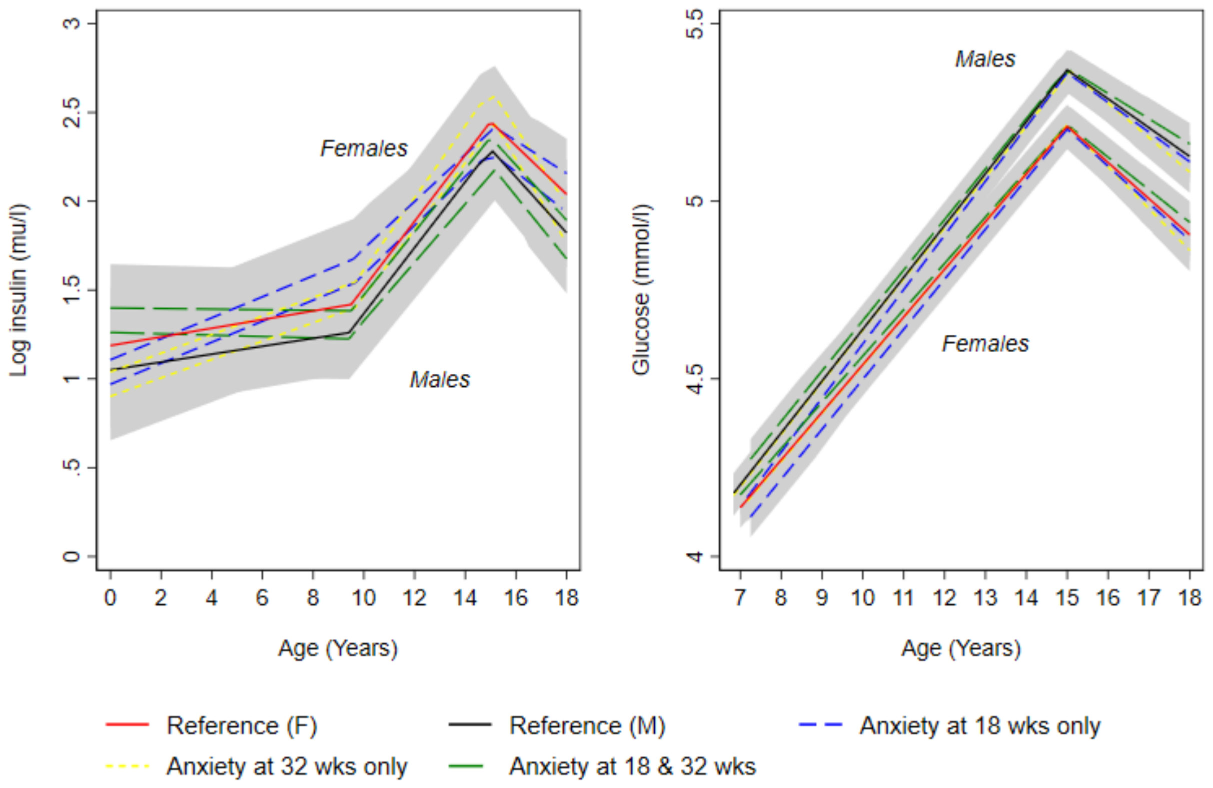

Figure 4 Mean predicted confounder adjusted trajectories of log insulin (birth to 18 years) and glucose (7 to 18 years), by maternal anxiety levels during pregnancy

Legend: Trajectories are adjusted for maternal age at birth of offspring, parental household socioeconomic position, parity, maternal pre-pregnancy BMI and maternal smoking during pregnancy. Anxiety at 18 and 32 weeks gestation is defined as ≥85th percentile of the anxiety subscale of the Crown-Crisp Index CCEI) for the whole cohort at each time point separately (≥85th percentile = 8 at both 18 and 32 weeks gestation).

review only

Supplemental Material

Maternal prenatal anxiety and depression and trajectories of cardiometabolic risk factors across childhood and adolescence: a prospective cohort study

Karen Matvienko-Sikar[1], Kate N O' Neill[1], Abigail Fraser[2], Catherine Hayes[3], Laura D Howe[2], Anja C Huizink[4], Patricia M Kearney[1], Ali Khashan[1,5], Sarah Redsell[6], Linda M O'Keeffe[1, 2, 7]

1. School of Public Health, University College Cork, Ireland

2. Population Health Sciences, Bristol Medical School, Oakfield House, Oakfield Grove, Bristol, UK, BS82BN

3. School of Medicine, Trinity College Dublin, Dublin, Ireland

4. Department of Clinical, Neuro- and Developmental Psychology, VU University Amsterdam, Amsterdam, The Netherlands

5. Irish Centre for Maternal and Child Research (INFANT) Centre, Cork University Maternity Hospital, Cork, T12 YE02, Ireland

6. University of Nottingham, University Park, Nottingham, UK, NG7 2RD

7. MRC Integrative Epidemiology Unit at the University of Bristol, Oakfield House, Oakfield Grove, Bristol, UK, BS82BN

Corresponding author: Dr Karen Matvienko-Sikar, School of Public Health, 4th Floor Western Gateway Building, University College Cork, Ireland. karen.msikar@ucc.ie

For peer review only - http://bmjopen.bmj.com/site/about/guidelines.xhtml

List of contents

Supplemental Methods

Supplemental Methods S1 Details of study exposure measurement and prevalence
Supplemental Methods S2 Details of measurement sources for cardiometabolic risk factors
Supplemental Methods S3 Details on measurement of confounders
Supplemental Methods S4 Details of model selection for outcomes

Supplemental Tables

Supplemental Table S1 Model details for log BMI trajectories
Supplemental Table S2 Model details for log fat mass trajectories
Supplemental Table S3 Model details for lean mass trajectories
Supplemental Table S4 Model details for SBP, DBP and pulse rate trajectories
Supplemental Table S5 Model details for glucose trajectories
Supplemental Table S6 Model details for log insulin and triglyceride trajectories
Supplemental Table S7 Model details for HDL-c and non-HDL-c trajectories
Supplemental Table S8 Number of participants with cardiometabolic measures at each time point
Supplemental Table S9 Characteristics at birth of the mothers of children included in models compared with those excluded due to missing exposure, outcome or confounder data
Supplemental Table S10 Mean trajectories of BMI estimated from multilevel models, by maternal anxiety during pregnancy
Supplemental Table S11 Mean trajectories of fat mass estimated from multilevel models, by maternal anxiety during pregnancy
Supplemental Table S12 Mean trajectories of blood pressure and pulse rate estimated from multilevel models, by maternal anxiety during pregnancy
Supplemental Table S13 Mean trajectories of lean mass estimated from multilevel models, by maternal anxiety during pregnancy
Supplemental Table S14 Mean trajectories of glucose estimated from multilevel models, by maternal anxiety during pregnancy
Supplemental Table S15 Mean trajectories of log insulin and triglyceride estimated from multilevel models, by maternal anxiety during pregnancy
Supplemental Table S16 Mean trajectories of HDL-c and non-HDL-c estimated from multilevel models, by maternal anxiety during pregnancy
Supplemental Table S17 Mean trajectories of BMI estimated from multilevel models, by maternal depression during pregnancy
Supplemental Table S18 Mean trajectories of fat mass estimated from multilevel models, by maternal depression during pregnancy
Supplemental Table S19 Mean trajectories of blood pressure and pulse rate estimated from multilevel models, by maternal depression during pregnancy
Supplemental Table S20 Mean trajectories of lean mass estimated from multilevel models, by maternal depression during pregnancy
Supplemental Table S21 Mean trajectories of glucose estimated from multilevel models, by maternal depression during pregnancy
Supplemental Table S22 Mean trajectories of log insulin and triglyceride estimated from multilevel models, by maternal depression during pregnancy
Supplemental Table S23 Mean trajectories of HDL-c and non-HDL-estimated from multilevel models, by maternal depression during pregnancy

Supplemental Figures

Supplemental Figure S1 Flow diagram of study design
Supplemental Figure S2 Mean predicted confounder adjusted trajectories of lean mass (9 to 18 years), by continuous maternal anxiety levels during pregnancy
Supplemental Figure S3 Mean predicted confounder adjusted trajectories of log fat mass (9 to 18 years), by continuous maternal anxiety levels during pregnancy
Supplemental Figure S4 Mean predicted confounder adjusted trajectories of log BMI (1 to 18 years), by continuous maternal anxiety levels during pregnancy
Supplemental Figure S5 Mean predicted confounder adjusted trajectories of pulse rate from 7 to 18 years by continuous maternal anxiety levels during pregnancy
Supplemental Figure S6 Mean predicted confounder adjusted trajectories of SBP from 7 to 18 years by continuous maternal anxiety levels during pregnancy

For peer review only

Supplemental Figure S7 Mean predicted confounder adjusted trajectories of DBP from 7 to 18 years by continuous maternal anxiety levels during pregnancy
Supplemental Figure S8 Mean predicted confounder adjusted trajectories of log insulin from (birth to 18 years) by continuous maternal anxiety levels during pregnancy
Supplemental Figure S9 Mean predicted confounder adjusted trajectories of glucose (7 to18 years), by continuous maternal anxiety levels during pregnancy
Supplemental Figure S10 Mean predicted confounder adjusted trajectories of HDL-c (birth to18 years), by continuous maternal anxiety levels during pregnancy
Supplemental Figure S11 Mean predicted confounder adjusted trajectories of triglyceride (birth to18 years), by continuous maternal anxiety levels during pregnancy
Supplemental Figure S12 Mean predicted confounder adjusted trajectories of non-HDL-c (birth to18 years), by continuous maternal anxiety levels during pregnancy
Supplemental Figure S13 Mean predicted confounder adjusted trajectories of lean mass (9 to 18 years), log fat mass (9 to 18 years) and log BMI (1 to 18 years), by categorical maternal depression levels during pregnancy
Supplemental Figure S14 Mean predicted confounder adjusted trajectories of pulse rate, SBP and DBP from 7 to 18 years, by categorical maternal depression levels during pregnancy
Supplemental Figure S15 Mean predicted confounder adjusted trajectories of log insulin (birth to 18 years) and glucose (7 to18 years), by categorical maternal depression levels during pregnancy
Supplemental Figure S16 Mean predicted confounder adjusted trajectories of HDL-c, triglyceride and non-HDL-c from birth to 18 years, by categorical maternal depression levels during pregnancy
Supplemental Figure S17 Mean predicted confounder adjusted trajectories of lean mass (9 to 18 years), by continuous maternal depression levels during pregnancy
Supplemental Figure S18 Mean predicted confounder adjusted trajectories of log fat mass (9 to 18 years), by continuous maternal depression levels during pregnancy
Supplemental Figure S19 Mean predicted confounder adjusted trajectories of log BMI (1 to 18 years), by continuous maternal depression levels during pregnancy
Supplemental Figure S20 Mean predicted confounder adjusted trajectories of pulse rate from 7 to 18 years by continuous maternal depression levels during pregnancy
Supplemental Figure S21 Mean predicted confounder adjusted trajectories of SBP from 7 to 18 years by continuous maternal depression levels during pregnancy
Supplemental Figure S22 Mean predicted confounder adjusted trajectories of DBP from 7 to 18 years by continuous maternal depression levels during pregnancy
Supplemental Figure S23 Mean predicted confounder adjusted trajectories of log insulin from (birth to 18 years) by continuous maternal depression levels during pregnancy
Supplemental Figure S24 Mean predicted confounder adjusted trajectories of glucose (7 to18 years), by continuous maternal depression levels during pregnancy
Supplemental Figure S25 Mean predicted confounder adjusted trajectories of HDL-c (birth to18 years), by continuous maternal depression levels during pregnancy
Supplemental Figure S26 Mean predicted confounder adjusted trajectories of triglyceride (birth to18 years), by continuous maternal depression levels during pregnancy
Supplemental Figure S27 Mean predicted confounder adjusted trajectories of non-HDL-c (birth to18 years), by continuous maternal depression levels during pregnancy

For peer review only

Supplemental Methods S1 Details of study exposure measurement and prevalence

Maternal self-report prenatal anxiety

Maternal prenatal anxiety was measured using the eight items from the anxiety subscale of Crown Crisp Experiential Index (CCEI). The CCEI is a validated self-report measure, with anxiety items rated on a 4-point scale ("very often" to "never"); a higher score is indicative of higher anxiety. Items ask women how they feel 'at this stage in pregnancy' and include "Do you sometimes feel panicky". This scale has previously demonstrated internal consistency coefficients above 0.80 in the ALSPAC cohort during pregnancy[8].

Maternal self-report depression

Prenatal depression was measured using the Edinburgh Postnatal Depression Scale (EPDS). This is a validated 10-item self-report measure, designed and used to screen women for depression before and after pregnancy. The EPDS has an internal consistency coefficient of .87[2]. Items ask women to rate how they felt in the last week in relation to statements such as, 'Things have been getting on top of me'. A higher score is indicative of greater depressive symptoms, with a score of 13 and above considered indicative of depression; the cut-off of 13 is typically used to determine presence of clinical depression[33].

For peer review only

Supplemental Methods S2 Details of measurement sources for cardiometabolic risk factors

Details on measurement of height and weight at research clinics

Data from age 1 onwards are included in this analysis. We did not include measures before 1 year because of the difficulty in accurately modelling BMI from birth through the whole of childhood due to its early peak followed by adiposity rebound. From 1 to 5 years, measures were available from routine child health clinics for most children and extracted from health visitor records, which form part of standard child care in the UK. Data were also available from research clinic measurements on a random 10% subsample of the cohort. All cohort members were invited to research clinics from age 7 onwards. Across all ages parent-reported measures were available.

At the clinics, crown-heel length for children aged four to 25 months was measured using a Harpenden Neonatometer and from 25 months onwards standing height was measured using a Leicester Height Measure; weight was measured using Fereday 100kg combined scale (four-month clinic), Soenhle scale or Seca scale model 724 (eight-month clinic), Seca 724 or Seca 835 (12-month clinic), Seca 835 (18 months onwards). From age 7 years, all children were invited to annual clinics, at which standing height was measured to the last complete mm using the Harpenden Stadiometer and weight was measured to the nearest 0.1kg using the Tanita Body Fat Analyser (Model TBF 305).

Details on measurement of blood pressure

A Dinamap 9301 Vital Signs Monitor (Morton Medical, London) was used at 7, 9, 11, 15 and 18 years; an Omron MI-5 was used at the 10-year clinic; a Dinamap 8100 Vital Signs Monitor (Morton Medical) was used at the 12-year clinic.

Details on measurement of blood based biomarkers

Non-fasting glucose was measured at age 7 years using Nuclear Magnetic Resonance (NMR) spectroscopy. In a random 10% of the cohort at age 9 years, fasting glucose and insulin were also available; these were taken as part of a continuation of an earlier sub-study called “Child in Focus” that included approximately 10% of the overall cohort. Fasting glucose and insulin were available from research clinics held when participants were 15 and 18 years old[54, 55].

Plasma lipid assays (triglyceride and high-density lipoprotein cholesterol (HDL-c)) were performed by modification of the standard Lipid Research Clinics Protocol using enzymatic reagents for lipid determination. All assay coefficients of variation were <5%. Samples were collected after an overnight fast and were analysed by the hexokinase method. Insulin was measured by an ELISA (Mercodia, Uppsala, Sweden) that does not cross-react with proinsulin. All assay coefficients of variation were <5%.

Details on measurement on biomarkers using Nuclear Magnetic Resonance (NMR) spectroscopy

A comprehensive profiling of offspring circulating lipids, lipoproteins, and metabolites was done by a high-throughput NMR metabolomics platform, providing a snapshot of offspring serum metabolome. At age 7, this was done on fasted blood samples and glucose is included in our analyses. At age 15 and 18, non-fasted bloods were used.

For peer review only - http://bmjopen.bmj.com/site/about/guidelines.xhtml

Supplemental Methods S3 Details on measurement of confounders

Data on confounders was collected using self-report measures at 18 and/or 32 weeks gestation.

Household social class was measured as the highest of the mother's or her partner's occupational social class using data on job title and details of occupation collected about the mother and her partner from the mother's questionnaire at 32 weeks gestation. Social class was derived using the standard occupational classification (SOC) codes developed by the United Kingdom Office of Population Census and Surveys and classified as I professional, II managerial and technical, IIINM non-manual, IIIM manual, and IV&V part skilled occupations and unskilled occupation.

Parity data was collected using a self-report item asking 'How many times have you been pregnant altogether before this time?', with responses categorised as 0, 1 or 2 based on participant responses.

Maternal age at delivery was collected as self-report data and measured in years.

Maternal Education maternal education was reported at 32 weeks gestation according to increasing levels of achievement. These levels were: less than an Ordinary level (O Level), which was categorised as no education or a certificate of secondary education, subject-specific qualifications at a lower level than O levels that were obtained by age 16 years; O level, which are subject-specific qualifications generally obtained at age 16 years; Advanced level (A Level), which are subject-specific qualifications generally obtained at age 18 years; and university degree or above.

Maternal smoking during pregnancy was self-reported in response to the question "How many times per day did you smoke" at 18 weeks; and "How many cigarettes per day are you yourself smoking at the moment" at 32 weeks. Prenatal smoking was categorised as No smoking or Yes smoking in the current study based on responses to these questions.

Maternal pre-pregnancy BMI data was collected using a self-report measure at 12 weeks gestation. Women were asked "What was your weight before you started this pregnancy? (please indicate whether stones, pounds or kilos)" and "How tall are you? (Please indicate whether feet, inches or metres)". BMI was calculated as kg/m^2.

For peer review only

Supplemental Methods S4 Details of model selection for outcomes

Two approaches, fractional polynomials and linear splines were used in the modelling of trajectories as described previously.

Fractional polynomials were used for BMI, due to the complex pattern of change in BMI during childhood and adolescence. Fractional polynomials involve raising age to many combinations of powers, resulting in a wide range of possible curves and offering more flexibility than standard polynomial approaches.

Linear splines were used to model all other outcomes, as too few measurement occasions were available to permit modelling using fractional polynomials or other age term combinations. Linear splines allow knot points to be fitted at different ages to derive periods of change that are approximately linear. Models were derived by initially examining observed data for each risk factor. We also plotted mean values for each risk factor on each measurement occasion to assist on decisions regarding knot points. We compared observed and predicted measurements for a selection of suitable models for each risk factor. We examined rates of change between time periods in order to examine whether changes between periods were similar or different. In cases where rates of change between two spline periods appeared identical, the fit of models with reduced splines was explored. We also compared model fit statistics (Akaike's Information Criterion) for several models with different knot points (with knot points placed at whole years closest to mean age at clinics due to a greater density of measures). Systolic blood pressure (SBP) and diastolic blood pressure (DBP) models have been modelled previously and are described elsewhere in detail.

In all models, age (in years) was centred at the first available measure. For each risk factor except insulin, we included all participants with at least one measure of the risk factor in each multilevel model, under a missing at random (MAR) assumption, to minimise selection bias. The observations of participants who reported being pregnant at the 18-year clinic were excluded from the multilevel models at that time point only (n=6). Models for insulin included participants with at least one measure before and after 11 years of age to improve model fit due to the sparsity of measures at the earlier time points (birth and 9 years).

BMI has been modelled previously using fractional polynomials and is described elsewhere[34]. BMI was modelled from age 1 as BMI is not considered an appropriate measure of adiposity in infants. Briefly, BMI was log transformed due to skewness of the data and fractional polynomials were used where age was raised to various combinations of powers (each of the following single powers, plus each combination of two powers: 0.5, 1, 2, 3, -0.5, -1, -2, natural log), from which we selected the best fitting curve (the one with the lowest likelihood value). The resulting curve contained three age terms including log age, log age* age and log age *age^2. To account for the likely reduced accuracy of parent-reported measurements, a binary indicator of measurement source (research clinic or health records versus parent-report) was included as a fixed effect. The variance of measurement occasion-level residuals (the differences between observed and predicted measurements) was allowed to vary with age for log BMI. The model took the form of: $\log BMI_{ij} = (\beta_0+u_{0j}+e_{0ij}) + (\beta_1+u_{1j})(\ln(age)_{ij}) + (\beta_2+u_{2j})(age*\ln(age)_{ij}) + (\beta_3+u_{3j})(age^2*\ln(age)_{ij}) + (\beta_8+e_{1ij})(measurement\_source_{ij}) + e_{ij}(age\_months_{ij})$ where for person j at measurement occasion i; β's represent fixed effect coefficients, u_{0j} to u_{3j} indicate person-specific random effects for the intercept and linear, quadratic and cubic age terms respectively, and e_1 represents the occasion-specific residuals or measurement error which was allowed to vary with age and according to measurement source.

Fat mass and **lean mass** were measured on 5 occasions between 9 and 18 years. Fat mass was log transformed due to skewness of the data. Knots were placed at 13 and 15 resulting in three periods of change; from 9-13, 13-15, 15-18. Both models were adjusted for a time-and sex-varying height co-variate which was included as a fixed effect For lean mass, the covariance between the second spline from 13 to 15 and third spline from 15 to 18 was set to zero to improve model convergence. The models took the form of: $\log$ fat $mass_{ij}$ / lean $mass_{ij} = \beta_0 + u_{0j} + (\beta_1 + u_{1j})s_{ij1} + (\beta_2 + u_{2j})s_{ij2} + (\beta_3 + u_{3j})s_{ij3} + \beta_4$ (age&sex adjusted height covariate)$_{ij} + e_{ij}$ where for person j at measurement occasion i; β_0 represents the fixed effect coefficient for the average intercept, β_1 to β_3 represent fixed effect coefficients for the average linear slopes of each linear spline, β_4 represents the fixed effect coefficient for the average difference in measurements between individuals of different heights, u_{0j} to u_{3j} indicate person-specific random effects for the intercept and slopes respectively, and e_{ij} represents the occasion-specific residuals or measurement error which was allowed to vary with age.

SBP, DBP and **pulse rate** were measured at 7 time points from 7 to 18 years[35-38]. The knots for all models were placed at 12 and 16 resulting in three periods of change; from 7 to 12, 12-16 and 16-18. All models included a

For peer review only - http://bmjopen.bmj.com/site/about/guidelines.xhtml

fixed effect to adjust for the use of the use of an Omron MI-5 machine to measure SBP in 10-year clinic which differed from all other clinics and a binary time indicator as a level 1 random effect of age less than or greater than 10 years to account for changing measurement error with age. The models took the form of: SBP_{ij} /DBP_{ij} /$pulse_{ij} = \beta_0 + u_{0j} + (\beta_1 + u_{1j})s_{ij1} + (\beta_2 + u_{2j})s_{ij2} + (\beta_3 + u_{3j})s_{ij3} + \beta_4(machine)_{ij} + e_{ij}(age\_binary_{ij})$ where for person j at measurement occasion i; β_0 represents the fixed effect coefficient for the average intercept, β_1 to β_3 represent fixed effect coefficients for the average linear slopes of each linear spline, β_4 represents the fixed effect coefficient for the average difference in measurements between the machine used at the 10 year clinic compared to the machine used at other clinics, u_{0j} to u_{3j} indicate person-specific random effects for the intercept and slopes respectively, and e_{ij} represents the occasion-specific residuals or measurement error which was allowed to vary with age.

Triglyceride and **HDL-c** were measured 5 times from birth to 18 years. Triglyceride was log transformed due to the skewness of the data. **Non-HDL-c** was derived by subtracting HDL-c from total cholesterol. Knots for triglyceride and non-HDL-c were placed at 9 and 15 years resulting in two periods of change; from birth to 9 years and 9 to 18. Knots were placed at age 7 and 15 years for HDL-c resulting in two periods of change; from birth to 7 years and 7 - 18 years. The models took the form of: log $triglyceride_{ij}$/$HDL\text{-}c_{ij}$/$non\text{-}HDL\text{-}c_{ij} = \beta_0 + u_{0j} + (\beta_1 + u_{1j})s_{ij1} + (\beta_2 + u_{2j})s_{ij2} + e_{ij}$ where for person j at measurement occasion i; β_0 represents the fixed effect coefficient for the average intercept, β_1 and β_2 represent fixed effect coefficients for the average linear slopes of each linear spline, u_{0j} to u_{3j} indicate person-specific random effects for the intercept and slopes respectively, and e_{ij} represents the occasion-specific residuals or measurement error.

Glucose was measured on four occasions (7, 9, 15, and 18). A knot was placed at 15 resulting in two periods of change; from 7 to 15 and 15 to 18. Due to few available repeated measures of glucose, we modelled the person-specific random effects as a single linear slope rather than a function of the splines as was done in all other linear spline models. This allowed person specific variation from the average trajectory but under the assumption that person-specific deviation from the mean trajectory was constant over time. The model took the form of: $glucose_{ij} = \beta_0 + u_{0j} + (\beta_1)s_{ij1} + (\beta_2)s_{ij2} + u_{1j}{*}age + e_{ij}$ where for person j at measurement occasion i; β_0 represents the fixed effect coefficient for the average intercept, β_1 and β_2 represent fixed effect coefficients for the average linear slopes of each linear spline, u_{0j} to u_{1j} indicate person-specific random effects for the intercept and slope respectively, and e_{ij} represents the occasion-specific residuals or measurement error.

Insulin was measured on four occasions (birth, 9, 15, and 18). Due to the sparsity of measures at birth and 9 years, the model for insulin was restricted to participants with at least one measure before and after age 11, to improve model fit. Knots were placed at 9 and 15 resulting in three periods of change; from birth to 9 years, 9 to 15 and 15 to 18. Insulin was log transformed due to skewness of the data. The model took the form of: log $insulin_{ij} = \beta_0 + u_{0j} + (\beta_1 + u_{1j})s_{ij1} + (\beta_2 + u_{2j})s_{ij2} + (\beta_3 + u_{3j}))s_{ij3+}\ e_{ij}$ where for person j at measurement occasion i; β_0 represents the fixed effect coefficient for the average intercept, β_1 to β_3 represent fixed effect coefficients for the average linear slopes of each linear spline, u_{0j} to u_{3j} indicate person-specific random effects for the intercept and slopes respectively, and e_{ij} represents the occasion-specific residuals or measurement error.

For peer review only

Supplemental Table S1 Model details for log BMI trajectories

	No of contributing individuals		Assessment of model fit			
	Total number of observations	Number of individuals with 1 measure	Mean observed BMI, log (kg/m^2) (SD) [a]	Mean predicted BMI, log (kg/m^2) (SD) [a]	Mean difference (observed – predicted), log (kg/m^2) [a]	95% level of agreement between observed and predicted, log (kg/m^2) [a]
**Overall**	80796	8606				
**1-3 years**	11042	7131	2.84 (0.09)	2.84 (0.06)	-0.01	-0.17 to 0.15
**3-7 years**	21216	7734	2.78 (0.10)	2.79 (0.07)	-0.01	-0.17 to 0.15
**7-9 years**	7033	6077	2.77 (0.12)	2.80 (0.10)	-0.03	-0.17 to 0.10
**9-11 years**	9850	5828	2.84 (0.14)	2.85 (0.12)	-0.01	-0.12 to 0.10
**11-13 years**	11069	5318	2.90 (0.16)	2.90 (0.13)	0.0002	-0.11 to 0.12
**13-15 years**	12118	5312	2.97 (0.16)	2.97 (0.14)	0.0002	-0.13 to 0.13
**15-18 years**	8468	4518	3.07 (0.16)	3.09 (0.15)	-0.02	-0.14 to 0.10

BMI, Body Mass Index; SD, standard deviation
[a] BMI is natural log transformed. All values are in log form.

For peer review only

Supplemental Table S2 Model details for log fat mass trajectories

	No of contributing individuals		Assessment of model fit			
	Total number of observations	Number of individuals with 1 measure	Mean observed fat mass, log (kg) (SD) [a]	Mean predicted fat mass, log (kg) (SD)[a]	Mean difference (observed – predicted), log (kg)[a]	95% level of agreement between observed and predicted, log (kg)[a]
**Overall**	21615	6032				
**9 years**	5134	5134	1.97 (0.57)	1.97 (0.54)	-0.01	-0.18 to 0.17
**9-13 years**	10099	10099	2.13 (0.59)	2.12 (0.56)	0.01	-0.20 to 0.21
**13-15 years**	4371	4371	2.44 (0.59)	2.46 (0.55)	-0.02	-0.28 to 0.24
**15-18 years**	7145	7145	2.63 (0.60)	2.63 (0.57)	0.01	-0.19 to 0.20

SD, standard deviation
[a] Fat mass is natural log transformed. All values are in log form.

For peer review only

Supplemental Table S3 Model details for lean mass trajectories

	No of contributing individuals		Assessment of model fit			
	Total number of observations	Number of individuals with 1 measure	Mean observed lean mass, kg (SD)	Mean predicted lean mass, kg (SD)	Mean difference (observed – predicted), kg	95% level of agreement between observed and predicted, kg
**Overall**	21615	6032				
**9 years**	5134	5134	24.56 (3.16)	24.57 (2.81)	-0.01	-2.53 to 2.52
**9-13 years**	10099	10099	27.10 (4.57)	27.11 (4.27)	0.00	-2.45 to 2.44
**13-15 years**	4371	4371	38.01 (6.41)	38.01 (5.97)	0.00	-2.94 to 2.95
**15-18 years**	7145	7145	44.35 (9.26)	44.35 (9.06)	0.003	-1.92 to 1.92

SD, standard deviation

For peer review only

Supplemental Table S4 Model details for SBP, DBP and pulse rate trajectories

	No of contributing individuals		Assessment of model fit			
	Total number of observations	Number of individuals with 1 measure	Mean observed SBP, DBP or pulse rate, (SD) [a]	Mean predicted SBP, DBP or pulse rate, (SD) [a]	Mean difference (observed – predicted) [a]	95% level of agreement between observed and predicted [a]
**SBP**						
**Overall**	32900	6671				
**7 years**	5672	5672	98.76 (9.13)	98.62 (5.37)	0.15	-10.70 to 11.00
**7-12 years**	20670	6479	102.50 (9.50)	102.70 (6.21)	-0.20	-11.80 to 11.40
**12-16 years**	8694	5090	115.82 (11.77)	115.35 (8.84)	0.46	-11.31 to 12.24
**16-18 years**	3536	3428	116.82 (10.15)	117.14 (7.44)	-0.31	-12.48 to 11.85
**DBP**						
**Overall**	32900	6671				
**7 years**	5672	5672	56.34 (6.55)	56.56 (3.37)	-0.22	-8.90 to 8.46
**7-12 years**	20670	6479	57.99 (6.92)	56.88 (3.39)	1.11	-9.09 to 11.31
**12-16 years**	8694	5090	61.07 (9.61)	61.92 (5.07)	-0.86	-12.62 to 10.91
16-18 years	3536	3428	64.17 (6.06)	64.49 (3.68)	-0.32	-13.14 to 12.50
**Pulse**						
**Overall**	32900	6671				
**7 years**	5672	5672	83.18 (10.65)	83.15 (6.11)	0.03	-13.08 to 13.13
**7-12 years**	20670	6479	77.57 (11.54)	78.91 (7.01)	-1.34	-15.93 to 13.25
**12-16 years**	8694	5090	74.00 (11.29)	74.08 (7.03)	-0.08	-13.88 to 13.72
**16-18 years**	3536	3428	65.59 (10.04)	65.85 (6.29)	-0.27	-14.69 to 14.16

DBP, diastolic blood pressure; SBP, systolic blood pressure; SD, standard deviation.
[a]Units are in mmHg for SBP and DBP and bpm for pulse rate.

For peer review only

For peer review only - http://bmjopen.bmj.com/site/about/guidelines.xhtml

Supplemental Table S5 Model details for glucose trajectories

	No of contributing individuals		Assessment of model fit			
	Total number of observations	Number of individuals with 1 measure	Mean observed glucose, mmol/l (SD)	Mean predicted glucose, mmol/l (SD)	Mean difference (observed – predicted), mmol/l	95% level of agreement between observed and predicted, mmol/l
**Overall**	9390	5045				
**7 years**	3907	3907	4.18 (0.50)	4.22 (0.24)	-0.03	-0.59 to 0.53
**7-15 years**	4576	4065	4.29 (0.55)	4.28 (0.27)	0.01	-0.59 to 0.61
**15-18 years**	4812	3203	5.12 (0.39)	5.13 (0.17)	-0.01	-0.65 to 0.62

Mmol/l, millimoles per litre; SD, standard deviation

For peer review only

Supplemental Table S6 Model details for log insulin and log triglyceride trajectories

	No of contributing individuals		Assessment of model fit			
	Total number of observations	Number of individuals with 1 measure	Mean observed triglycerides, (SD) [a]	Mean predicted triglycerides, (SD) [a]	Mean difference (observed – predicted)[a]	95% level of agreement between observed and predicted
**Log insulin**						
**Overall**	1464	526				
**Birth**	206	206	1.05 (0.48)	1.04 (0.17)	0.01	-0.63 to 0.64
**0-9 years**	206	206	1.05 (0.48)	1.04 (0.17)	0.01	-0.63 to 0.64
**9-15 years**	445	443	1.58 (0.62)	1.56 (0.39)	0.02	-0.48 to 0.52
**9-18 years**	813	523	2.07 (0.53)	2.07 (0.27)	0.0001	-0.63 to 0.63
**Log triglyceride**						
**Overall**	15239	6641				
**Birth**	2963	2963	-0.71 (0.41)	-0.71 (0.15)	-0.001	-0.52 to 0.51
**0-9 years**	6808	5581	-0.34 (0.53)	-0.35 (0.36)	0.002	-0.56 to 0.56
**9-18 years**	8431	4595	-0.14 (0.41)	-0.13 (0.22)	-0.002	-0.56 to 0.55

SD, standard deviation

[a] Insulin and triglyceride are natural log transformed. All values are in log form. Units for log insulin are in milliunits per litre. Units for log triglyceride are in millimoles per litre.

For peer review only

Supplemental Table S7 Model details for HDL-c and non-HDL-c trajectories

	No of contributing individuals		Assessment of model fit			
	Total number of observations	Number of individuals with 1 measure	Mean observed HDL-c, mmol/l (SD)	Mean predicted HDL-c, mmol/l (SD)	Mean difference (observed – predicted), mmol/l	95% level of agreement between observed and predicted, mmol/l
**Overall**	15239	6641				
**Birth**	2963	2963	0.52 (0.24)	0.52 (0.11)	-0.000002	-0.24 to 0.24
**0-7 years**	6808	2965	1.09 (0.56)	1.08 (0.52)	0.01	-0.24 to 0.25
**7-18 years**	12274	5466	1.39 (0.32)	1.39 (0.24)	-0.00002	-0.25 to 0.25
**Overall**	15239	6641				
**Birth**	2963	2963	1.22 (0.53)	1.25 (0.25)	-0.02	-0.59 to 0.54
**0-9 years**	6808	5581	2.15 (1.00)	2.10 (0.82)	0.05	-0.53 to 0.63
**9-18 years**	8431	4595	2.63 (0.65)	2.67 (0.52)	-0.04	-0.59 to 0.51

HDL-c, high density lipoprotein cholesterol; Non-HDL-c, non-high density lipoprotein cholesterol; Mmol/l, millimoles per litre; SD, standard deviation.

For peer review only

Supplemental Table S8 Number of participants with cardiometabolic health outcome measures at each time point

	Birth	Age 1	Age 7	Age 9	Age 10	Age 11	Age 12	Age 13	Age 15	Age 18
BMI [a]		x	x	x	x	x	x	x	x	x
Fat/lean mass				5,134		4,958		4,348	3,738	3,437
SBP/DBP/pulse rate			5,672	5,347	5,084	4,970	4,729		3,787	3,311
Glucose			3,907	662					2,485	2,336
Lipids [b]	2,863		3,846	3,617					2,485	2,328
Insulin	206		440						417	401

DBP, diastolic blood pressure; SBP, systolic blood pressure.

[a] Measures available at each of these approximate ages and at several ages in between but exact timing and number of BMI measures not shown as measures were available from questionnaires, routine child health records and research clinics at different mean ages from 1 to 18 years.

[b] Lipids include triglyceride, high density lipoprotein cholesterol (HDL-c) and non-HDL-c.

For peer review only

For peer review only - http://bmjopen.bmj.com/site/about/guidelines.xhtml

Supplemental Table S9 Characteristics at birth of the mothers of children included in models compared with those excluded due to missing exposure, outcome or confounder data

	Participants included n= 8,606	Participants excluded n= 5,261 – 13,341 [a]
	n (%)	n (%)
**Household social class**		
Professional	1190(13.8)	346(11.7)
Managerial & Technical	3673(42.7)	1148(38.9)
Non-Manual	2254(26.2)	690(23.4)
Manual	1037(12.0)	525(17.8)
Part Skilled & Unskilled	452(5.3)	240(8.1)
**Maternal education**		
Less than O level	2124(24.7)	1624(42.1)
O level	3167(36.8)	1151(29.8)
A level	2093(24.3)	700(18.1)
Degree or above	1222(14.2)	382(9.9)
**Partners highest educational qualification**		
Less than O level	2488(29.8)	1655(45.4)
O level	1872(22.4)	679(18.6)
A level	2337(28.0)	778(21.4)
Degree or Above	1643(19.7)	530(14.6)
**Maternal smoking during pregnancy**		
No	6781(78.8)	3171(67.7)
Yes	1825(21.2)	1516(32.3)
**Parity**		
0	3893(45.2)	1947(43.7)
1	3159(36.7)	1413(31.7)
2	1554(18.1)	1094(24.6)
	**Mean (SD)**	**Mean (SD)**
Child gestational age at birth	39.5(1.8)	37(8.1)
Birthweight (g)	3426(533)	3310(643)
Maternal BMI (kg/m^2)	23.0(3.8)	23(4.0)
Maternal age (years)	28.7(4.7)	27(5.2)

SD, standard deviation

[a] Denominators for excluded participants in this table vary due to different rates of missing data for characteristics shown.

For peer review only

Supplemental Table S10 Mean trajectories of BMI estimated from multilevel models, by maternal anxiety during pregnancy

	Mean trajectory (95% CI) in males (no anxiety during pregnancy) log (kg/m^2) (reference) [a]	Mean trajectory (95% CI) in females (no anxiety during pregnancy) log (kg/m^2) (reference) [a]	Mean difference in trajectory (95% CI) comparing anxiety at 18 weeks only with the reference trajectory (% difference in kg/m^2) [b]	Mean difference in trajectory (95% CI) anxiety at 32 weeks only with the reference trajectory (% difference in kg/m^2) [b]	Mean difference in trajectory (95% CI) comparing anxiety at 18 and 32 weeks with the reference trajectory (% difference in kg/m^2) [b]
**Unadjusted BMI**					
Age 1yr	2.90 (2.89,2.90)	2.89 (2.88,2.89)	0.42 (-1.00,1.84)	0.13 (-1.15,1.40)	0.3 (-0.85,1.53)
Age 3yr	2.77 (2.76,2.77)	2.75 (2.75,2.75)	0.65 (-0.08,1.38)	0.11 (-0.56,0.77)	0.0 (-0.60,0.60)
Age 7yr	2.79 (2.78,2.79)	2.80 (2.79,2.80)	0.95 (-0.01,1.91)	0.44 (-0.43,1.32)	0.6 (-0.20,1.39)
Age 9yr	2.82 (2.82,2.83)	2.84 (2.84,2.85)	1.06 (-0.11,2.24)	0.64 (-0.43,1.71)	0.9 (-0.06,1.89)
Age 11yr	2.87 (2.87,2.88)	2.90 (2.90,2.91)	1.16 (-0.18,2.50)	0.83 (-0.39,2.06)	1.2 (0.08,2.32)
Age 13yr	2.94 (2.93,2.95)	2.97 (2.96,2.97)	1.23 (-0.21,2.66)	1.03 (-0.28,2.34)	1.4 (0.24,2.65)
Age 15yr	3.02 (3.01,3.02)	3.04 (3.04,3.05)	1.27 (-0.23,2.76)	1.22 (-0.16,2.60)	1.6 (0.37,2.90)
Age 18yr	3.16 (3.15,3.16)	3.17 (3.16,3.18)	1.27 (-0.51,3.05)	1.50 (-0.16,3.17)	1.8 (0.29,3.33)
**Adjusted BMI**					
Age 1yr	2.88 (2.87,2.90)	2.87 (2.86,2.89)	0.36 (-1.06,1.79)	0.18 (-1.11,1.47)	0.3 (-0.91,1.51)
Age 3yr	2.77 (2.76,2.77)	2.75 (2.74,2.76)	0.40 (-0.32,1.13)	0.02 (-0.65,0.68)	-0.2 (-0.76,0.44)
Age 7yr	2.78 (2.78,2.79)	2.80 (2.79,2.81)	0.43 (-0.49,1.34)	0.27 (-0.57,1.11)	0.4 (-0.36,1.17)
Age 9yr	2.82 (2.81,2.83)	2.84 (2.83,2.86)	0.41 (-0.70,1.53)	0.39 (-0.63,1.41)	0.7 (-0.28,1.60)
Age 11yr	2.87 (2.86,2.89)	2.90 (2.89,2.91)	0.38 (-0.88,1.64)	0.49 (-0.66,1.65)	0.8 (-0.22,1.91)
Age 13yr	2.94 (2.92,2.95)	2.97 (2.95,2.98)	0.33 (-1.00,1.67)	0.57 (-0.66,1.80)	0.9 (-0.20,2.07)
Age 15yr	3.02 (3.00,3.03)	3.04 (3.03,3.06)	0.26 (-1.13,1.65)	0.61 (-0.67,1.90)	0.9 (-0.27,2.10)
Age 18yr	3.16 (3.14,3.18)	3.17 (3.15,3.19)	0.12 (-1.55,1.79)	0.61 (-0.96,2.19)	0.7 (-0.76,2.13)

CI, confidence interval; yr, year.

[a] BMI is presented in the natural log and values represent the mean predicted natural log of BMI at each age shown.

[b] Differences at each age are back transformed from the log scale and are interpreted as the percentage difference in the mean level in original units at each age comparing each category with the reference trajectory.

For peer review only

Supplemental Table S11 Mean trajectories of fat mass estimated from multilevel models, by maternal anxiety during pregnancy

	Mean trajectory (95% CI) in males (no anxiety during pregnancy) log (kg or kg/yr) (reference) [a]	Mean trajectory (95% CI) in females (no anxiety during pregnancy) log (kg or kg/yr) (reference) [a]	Mean difference in trajectory (95% CI) comparing anxiety at 18 weeks only with the reference trajectory (% or %/yr) [b]	Mean difference in trajectory (95% CI) comparing anxiety at 32 weeks only with the reference trajectory (% or %/yr) [b]	Mean difference in trajectory (95% CI) comparing anxiety at 18 and 32 weeks with the reference trajectory (% or %/yr) [b]
**Unadjusted**					
Age 9yr (kg) or (%)	1.79 (1.76,1.82)	2.63 (2.11,3.15)	3.62 (-2.42,9.67)	-1.46 (-6.78,3.87)	0.94 (-4.08,5.95)
Change 9-13yr (kg/yr) or (%/yr)	0.11 (0.11,0.12)	0.16 (0.15,0.17)	0.04 (-1.18,1.26)	1.00 (-0.15,2.15)	1.52 (0.44,2.60)
Change 13-15yr (kg/yr) or (%/yr)	-0.06 (-0.07,-0.05)	0.10 (0.09,0.11)	1.41 (-0.84,3.66)	0.21 (-1.86,2.28)	-1.62 (-3.49,0.26)
Change 15-18yr (kg/yr) or (%/yr)	0.10 (0.09,0.11)	0.06 (0.05,0.06)	-1.41 (-3.04,0.21)	-0.93 (-2.51,0.64)	0.81 (-0.65,2.27)
Age 18yr (kg) or (%)	2.43 (2.41,2.46)	3.64 (3.09,4.18)	2.28 (-3.83,8.40)	0.11 (-5.56,5.79)	6.33 (0.86,11.79)
**Adjusted**					
Age 9yr (kg) or (%)	1.81 (1.74,1.87)	2.66 (2.13,3.18)	1.56 (-4.21,7.32)	-2.02 (-7.17,3.14)	1.00 (-3.92,5.91)
Change 9-13yr (kg/yr) or (%/yr)	0.11 (0.09,0.12)	0.15 (0.14,0.17)	-0.13 (-1.34,1.08)	0.74 (-0.40,1.89)	1.19 (0.12,2.27)
Change 13-15yr (kg/yr) or (%/yr)	-0.07 (-0.09,-0.04)	0.09 (0.07,0.12)	1.56 (-0.70,3.82)	0.21 (-1.87,2.28)	-1.75 (-3.64,0.14)
Change 15-18yr (kg/yr) or (%/yr)	0.11 (0.09,0.12)	0.06 (0.05,0.08)	-1.47 (-3.10,0.16)	-0.93 (-2.51,0.64)	0.82 (-0.66,2.29)
Age 18yr (kg) or (%)	2.42 (2.35,2.48)	3.63 (3.08,4.18)	-0.32 (-6.10,5.45)	-1.46 (-6.88,3.97)	4.77 (-0.51,10.05)

CI, confidence interval; kg/yr, kilograms per year; %/yr, percentage per year.

[a] Fat mass was transformed using the natural log. All predicted mean values (kg) and rates of change per year (kg/yr) for the reference categories are on the log scale

[b] The difference between groups is back transformed from the log scale for ease of interpretation and is interpreted as the percentage difference in the mean level in original units comparing each category with the reference or percentage difference in change in original units per year (%/yr) comparing each category with the reference.

For peer review only - http://bmjopen.bmj.com/site/about/guidelines.xhtml

Supplemental Table S12 Mean trajectories of blood pressure and pulse rate estimated from multilevel models, by maternal anxiety during pregnancy

	Mean trajectory (95% CI) in males (no anxiety during pregnancy) (reference)	Mean trajectory (95% CI) in females (no anxiety during pregnancy) (reference)	Mean difference in trajectory (95% CI) comparing anxiety at 18 weeks only with the reference trajectory	Mean difference in trajectory (95% CI) comparing anxiety at 32 weeks only with the reference trajectory	Mean difference in trajectory (95% CI) comparing anxiety at 18 and 32 weeks with the reference trajectory
**SBP**					
**Unadjusted**					
Age 7yr (mmHg)	97.76 (97.39,98.12)	97.86 (97.48,98.23)	0.67 (-0.26,1.61)	0.34 (-0.52,1.20)	0.37 (-0.41,1.15)
Change 7-12yr (mmHg/yr)	1.65 (1.56,1.74)	1.87 (1.78,1.95)	0.08 (-0.14,0.31)	-0.01 (-0.22,0.19)	-0.07 (-0.25,0.12)
Change 12-16yr (mmHg/yr)	5.86 (5.70,6.01)	3.85 (3.70,4.00)	0.02 (-0.35,0.40)	0.02 (-0.33,0.38)	-0.04 (-0.36,0.28)
Change 16-18yr (mmHg/yr)	-4.06 (-4.40,-3.71)	-5.94 (-6.27,-5.61)	0.13 (-0.71,0.96)	-0.04 (-0.83,0.74)	0.42 (-0.30,1.15)
Age 18yr (mmHg)	121.32 (120.84,121.80)	110.73 (110.27,111.18)	1.44 (0.27,2.61)	0.28 (-0.82,1.38)	0.74 (-0.27,1.75)
**Adjusted**					
Age 7yr (mmHg)	98.91 (97.92,99.90)	99.04 (98.04,100.04)	0.53 (-0.40,1.47)	0.16 (-0.70,1.02)	0.23 (-0.55,1.01)
Change 7-12yr (mmHg/yr)	1.47 (1.24,1.71)	1.69 (1.45,1.93)	0.07 (-0.15,0.29)	-0.01 (-0.22,0.19)	-0.06 (-0.25,0.13)
Change 12-16yr (mmHg/yr)	5.97 (5.57,6.37)	3.97 (3.56,4.37)	0.04 (-0.33,0.42)	0.06 (-0.30,0.41)	-0.0001 (-0.32,0.32)
Change 16-18yr (mmHg/yr)	-4.24 (-5.16,-3.32)	-6.15 (-7.07,-5.23)	0.04 (-0.80,0.88)	-0.16 (-0.95,0.63)	0.22 (-0.52,0.95)
Age 18yr (mmHg)	121.67 (120.40,122.95)	111.07 (109.79,112.35)	1.16 (-0.02,2.33)	0.01 (-1.10,1.11)	0.38 (-0.64,1.40)
**DBP**					
**Unadjusted**					
Age 7yr (mmHg)	56.06 (55.80,56.32)	56.91 (56.64,57.18)	0.74 (0.06,1.41)	0.15 (-0.47,0.78)	0.40 (-0.17,0.96)
Change 7-12yr (mmHg/yr)	0.14 (0.07,0.21)	0.12 (0.05,0.19)	-0.02 (-0.19,0.16)	0.05 (-0.11,0.21)	-0.02 (-0.17,0.12)
Change 12-16yr (mmHg/yr)	2.87 (2.74,3.00)	2.34 (2.21,2.47)	-0.06 (-0.38,0.26)	-0.03 (-0.33,0.27)	-0.17 (-0.45,0.10)
Change 16-18yr (mmHg/yr)	-2.59 (-2.88,-2.30)	-1.17 (-1.45,-0.89)	0.27 (-0.43,0.98)	0.27 (-0.40,0.94)	0.46 (-0.14,1.07)
Age 18yr (mmHg)	63.05 (62.70,63.40)	63.65 (62.85,64.46)	0.97 (0.13,1.81)	0.84 (0.05,1.64)	0.52 (-0.20,1.24)
**Adjusted**					
Age 7yr (mmHg)	56.79 (56.08,57.51)	57.65 (56.93,58.38)	0.70 (0.02,1.38)	0.08 (-0.55,0.70)	0.33 (-0.24,0.90)
Change 7-12yr (mmHg/yr)	0.05 (-0.13,0.23)	0.03 (-0.15,0.22)	-0.02 (-0.19,0.15)	0.05 (-0.11,0.21)	-0.02 (-0.16,0.13)
Change 12-16yr (mmHg/yr)	2.95 (2.60,3.30)	2.43 (2.08,2.78)	-0.02 (-0.34,0.30)	0.03 (-0.27,0.33)	-0.09 (-0.37,0.19)
Change 16-18yr (mmHg/yr)	-2.73 (-3.52,-1.95)	-1.34 (-2.12,-0.55)	0.19 (-0.52,0.89)	0.11 (-0.56,0.78)	0.22 (-0.40,0.84)
Age 18yr (mmHg)	63.39 (62.46,64.31)	64.03 (62.85,65.21)	0.89 (0.05,1.73)	0.67 (-0.13,1.46)	0.31 (-0.42,1.04)
**Pulse rate**					
**Unadjusted**					
Age 7yr (bpm)	82.33 (81.90,82.75)	85.59 (85.16,86.03)	0.62 (-0.47,1.71)	1.18 (0.17,2.19)	0.68 (-0.23,1.59)
Change 7-12yr (bpm/yr)	-1.89 (-1.99,-1.79)	-1.73 (-1.83,-1.63)	0.01 (-0.26,0.27)	-0.30 (-0.54,-0.06)	-0.08 (-0.30,0.14)
Change 12-16yr (bpm/yr)	-0.71 (-0.87,-0.55)	-0.25 (-0.41,-0.10)	-0.09 (-0.48,0.30)	0.24 (-0.13,0.61)	0.17 (-0.16,0.51)
Change 16-18yr (bpm/yr)	-4.21 (-4.57,-3.85)	-4.83 (-5.17,-4.49)	0.62 (-0.24,1.49)	-0.03 (-0.84,0.78)	-0.04 (-0.78,0.70)
Age 18yr (bpm)	61.62 (61.10,62.15)	66.27 (65.77,66.76)	1.53 (0.26,2.80)	0.57 (-0.62,1.77)	0.92 (-0.17,2.01)
**Adjusted**					
Age 7yr (bpm)	82.57 (81.42,83.73)	85.80 (84.62,86.97)	0.62 (-0.47,1.71)	1.13 (0.12,2.14)	0.68 (-0.24,1.60)
Change 7-12yr (bpm/yr)	-1.90 (-2.18,-1.62)	-1.73 (-2.01,-1.45)	0.01 (-0.25,0.27)	-0.29 (-0.53,-0.05)	-0.06 (-0.29,0.16)
Change 12-16yr (bpm/yr)	-1.05 (-1.47,-0.63)	-0.60 (-1.03,-0.17)	-0.09 (-0.48,0.30)	0.22 (-0.15,0.58)	0.14 (-0.20,0.48)
Change 16-18yr (bpm/yr)	-3.67 (-4.62,-2.73)	-4.34 (-5.29,-3.39)	0.58 (-0.28,1.45)	-0.09 (-0.90,0.73)	-0.14 (-0.89,0.61)
Age 18yr (bpm)	61.52 (60.13,62.91)	66.06 (64.67,67.45)	1.48 (0.21,2.75)	0.37 (-0.83,1.56)	0.65 (-0.45,1.75)

For peer review only - http://bmjopen.bmj.com/site/about/guidelines.xhtml

bpm, beats per minute; bpm/yr, beats per minute per year; CI, confidence interval; mmHg, millimetres of mercury; mmHg/yr, millimetres of mercury per year.

Supplemental Table S13 Mean trajectories of lean mass estimated from multilevel models, by maternal anxiety during pregnancy

	Mean trajectory (95% CI) in males (no anxiety during pregnancy) (reference)	Mean trajectory (95% CI) in females (no anxiety during pregnancy) (reference)	Mean difference in trajectory (95% CI) comparing anxiety at 18 weeks only with the reference trajectory	Mean difference in trajectory (95% CI) comparing anxiety at 32 weeks only with the reference trajectory	Mean difference in trajectory (95% CI) comparing anxiety at 18 and 32 weeks with the reference trajectory
**Unadjusted**					
Age 9yr (kg)	23.89 (23.76,24.03)	20.78 (20.65,20.91)	-0.12 (-0.42,0.18)	-0.28 (-0.56,0.002)	-0.13 (-0.38,0.13)
Change 9-13yr (kg/yr)	2.30 (2.24,2.36)	3.22 (3.17,3.28)	0.11 (-0.02,0.24)	-0.10 (-0.22,0.02)	0.04 (-0.07,0.15)
Change 13-15yr (kg/yr)	7.69 (7.59,7.79)	1.58 (1.46,1.69)	-0.33 (-0.57, -0.08)	0.09 (-0.14,0.32)	0.003 (-0.21,0.21)
Change 15-18yr (kg/yr)	2.47 (2.40,2.55)	0.38 (0.31,0.45)	-0.0004 (-0.18,0.18)	0.04 (-0.13,0.21)	-0.03 (-0.18,0.13)
Age 18yr (kg)	55.91 (55.67,56.15)	37.96 (37.72,38.20)	-0.32 (-0.91,0.27)	-0.39 (-0.95,0.17)	-0.04 (-0.54,0.47)
**Adjusted**					
Age 9yr (kg)	23.84 (23.52,24.17)	20.76 (20.43,21.08)	-0.14 (-0.44,0.16)	-0.26 (-0.53,0.02)	-0.05 (-0.31,0.20)
Change 9-13yr (kg/yr)	2.30 (2.16,2.44)	3.22 (3.08,3.36)	0.09 (-0.04,0.22)	-0.10 (-0.22,0.01)	0.04 (-0.07,0.15)
Change 13-15yr (kg/yr)	7.48 (7.22,7.74)	1.36 (1.09,1.63)	-0.32 (-0.57,-0.07)	0.09 (-0.14,0.32)	0.01 (-0.21,0.22)
Change 15-18yr (kg/yr)	2.63 (2.44,2.82)	0.54 (0.35,0.73)	0.01 (-0.17,0.19)	0.05 (-0.12,0.22)	-0.02 (-0.17,0.14)
Age 18yr (kg)	55.90 (55.27,56.54)	37.99 (37.35,38.63)	-0.38 (-0.96,0.21)	-0.34 (-0.90,0.22)	0.07 (-0.44,0.58)

kg/yr, kilograms per year.

For peer review only

Supplemental Table S14 Mean trajectories of glucose estimated from multilevel models, by maternal anxiety during pregnancy

	Mean trajectory (95% CI) in males (no anxiety during pregnancy) (reference)	Mean trajectory (95% CI) in females (no anxiety during pregnancy) (reference)	Mean difference in trajectory (95% CI) comparing anxiety at 18 weeks only with the reference trajectory	Mean difference in trajectory (95% CI) comparing anxiety at 32 weeks only with the reference trajectory	Mean difference in trajectory (95% CI) c comparing anxiety at 18 and 32 weeks with the reference trajectory
**Unadjusted**					
Age 7yr (mmol/l)	4.19 (4.17,4.21)	4.13 (4.10,4.15)	-0.06 (-0.12,0.005)	-0.003 (-0.06,0.06)	0.03 (-0.02,0.08)
Change 7-15yr (mmol/l/yr)	0.15 (0.14,0.15)	0.13 (0.13,0.14)	0.007 (-0.005,0.02)	0.001 (-0.01,0.01)	-0.004 (-0.01,0.01)
Change 15-18yr (mmol/l/yr)	-0.08 (-0.09, -0.07)	-0.10 (-0.11, -0.09)	-0.003 (-0.04,0.03)	-0.02 (-0.05,0.01)	0.01 (-0.02,0.04)
Age 18yr (mmol/l)	5.11 (5.09,5.14)	4.89 (4.86,4.92)	-0.01 (-0.08,0.05)	-0.04 (-0.11,0.02)	0.03 (-0.02,0.09)
**Adjusted**					
Age 7yr (mmol/l)	4.21 (4.14,4.27)	4.14 (4.07,4.21)	-0.06 (-0.13,0.003)	-0.005 (-0.06,0.05)	0.04 (-0.02,0.09)
Change 7-15yr (mmol/l/yr)	0.14 (0.13,0.16)	0.13 (0.12,0.14)	0.01 (-0.005,0.02)	0.001 (-0.01,0.01)	-0.004 (-0.01,0.01)
Change 15-18yr (mmol/l/yr)	-0.08 (-0.11, -0.04)	-0.10 (-0.13, -0.06)	-0.003 (-0.04,0.03)	-0.02 (-0.05,0.02)	0.01 (-0.02,0.04)
Age 18yr (mmol/l)	5.13 (5.06,5.20)	4.91 (4.84,4.98)	-0.02 (-0.08,0.05)	-0.04 (-0.11,0.02)	0.04 (-0.02,0.09)

CI, confidence interval; mmol/l, millimole per litre; mmol/l/year, millimoles per litre per year.

For peer review only

For peer review only - http://bmjopen.bmj.com/site/about/guidelines.xhtml

Supplemental Table S15 Mean trajectories of insulin and triglyceride estimated from multilevel models, by maternal anxiety during pregnancy

	Mean trajectory (95% CI) in males (no anxiety during pregnancy) (reference) [a]	Mean trajectory (95% CI) in females (no anxiety during pregnancy) (reference) [a]	Mean difference in trajectory (95% CI) comparing anxiety at 18 weeks only with the reference trajectory (% or %/yr) [b]	Mean difference in trajectory (95% CI) comparing anxiety at 32 weeks only with the reference trajectory (% or %/yr) [b]	Mean difference in trajectory (95% CI) comparing anxiety at 18 and 32 weeks with the reference trajectory (% or %/yr) [b]
**Insulin**					
**Unadjusted**					
Birth (mu/l or %)	0.99 (0.89,1.09) 0.04	1.08 (0.99,1.18)	-7.29 (-31.57,16.99)	-13.73 (-38.87,11.41)	23.37 (-3.86,50.60)
Change 0-9yr (mu/l/yr or %/yr)	(0.03,0.06)	0.05 (0.03,0.06)	4.08 (-0.12,8.29)	2.82 (-1.64,7.27)	-2.58 (-5.91,0.75)
Change 9-15yr (mu/l/yr or %/yr)	0.14 (0.12,0.16)	0.14 (0.12,0.16)	-5.18 (-10.14, -0.22)	1.94 (-3.48,7.35)	-0.96 (-5.57,3.64)
Change 15-18yr (mu/l/yr or %/yr)	-0.14 (-0.18,-0.10)	-0.12 (-0.16, -0.08)	5.80 (-5.20,16.80)	-8.82 (-18.80,1.16)	-2.06 (-11.52,7.41)
Age 18yr (mu/l or %)	1.76 (1.68,1.85)	1.98 (1.89,2.06)	14.38 (-11.51,40.28)	-5.79 (-29.01,17.42)	-13.54 (-31.07,3.99)
**Adjusted**					
Birth (mu/l or %)	1.05 (0.78,1.32)	1.19 (0.91,1.46)	-7.71 (-31.62,16.20)	-13.80 (-38.45,10.85)	23.58 (-3.05,50.21)
Change 0-9yr (mu/l/yr or %/yr)	0.01 (-0.03,0.06)	0.02 (-0.03,0.06)	3.94 (-0.23,8.10)	2.63 (-1.76,7.02)	-2.66 (-5.93,0.62)
Change 9-15yr (mu/l/yr or %/yr)	0.19 (0.13,0.24)	0.19 (0.13,0.24)	-4.62 (-9.68,0.44)	1.58 (-3.85,7.01)	-0.96 (-5.60,3.67)
Change 15-18yr (mu/l/yr or %/yr)	-0.16 (-0.27,-0.05)	-0.14 (-0.24,-0.03)	4.58 (-6.45,15.62)	-7.53 (-17.55,2.48)	-1.89 (-11.31,7.53)
Age 18yr (mu/l or %)	1.82 (1.59,2.05)	2.04 (1.81,2.27)	12.52 (-13.06,38.11)	-5.44 (-28.32,17.45)	-13.57 (-30.83,3.70)
**Triglyceride**					
**Unadjusted**					
Birth (mmol/l or %)	-0.71 (-0.73,-0.69)	-0.70 (-0.72,-0.68)	-0.25 (-5.56,5.06)	-3.01 (-8.22,2.20)	1.66 (-2.92,6.24)
Change 0-9yr (mmol/l/yr or %/yr)	0.08 (0.08,0.09)	0.09 (0.09,0.09)	0.14 (-0.67,0.95)	0.19 (-0.60,0.98)	-0.35 (-1.04,0.33)
Change 9-18yr (mmol/l/yr or %/yr)	-0.04 (-0.04,-0.04)	-0.04 (-0.05,-0.04)	0.36 (-0.37,1.09)	0.08 (-0.60,0.77)	0.14 (-0.49,0.78)
Age 18yr (mmol/l or %)	-0.33 (-0.35,-0.31)	-0.30 (-0.32,-0.28)	4.34 (-0.82,9.51)	-0.60 (-5.30,4.11)	-0.26 (-4.66,4.13)
**Adjusted**					
Birth (mmol/l or %)	-0.66 (-0.72,-0.60)	-0.65 (-0.71,-0.59)	-1.50 (-6.63,3.63)	-2.86 (-7.96,2.25)	1.15 (-3.35,5.65)
Change 0-9yr (mmol/l/yr or %/yr)	0.08 (0.07,0.09)	0.09 (0.08,0.10)	0.25 (-0.55,1.06)	0.16 (-0.62,0.95)	-0.27 (-0.96,0.41)
Change 9-18yr (mmol/l/yr or %/yr)	-0.04 (-0.05,-0.04)	-0.05 (-0.05,-0.04)	0.28 (-0.45,1.00)	0.02 (-0.67,0.71)	-0.01 (-0.65,0.63)
Age 18yr (mmol/l or %)	-0.32 (-0.37,-0.26)	-0.28 (-0.34,-0.23)	3.29 (-1.83,8.42)	-1.24 (-5.92,3.44)	-1.35 (-5.73,3.04)

CI, confidence interval; mmol/l, millimole per litre; mmol/l/year, millimoles per litre per year; %/yr, percentage per year

[a] Insulin and triglyceride were transformed using the natural log. All predicted mean values and rates of change per year for the reference categories are on the log scale.

[b] The difference between groups is back transformed from the log scale for ease of interpretation and is interpreted as the percentage difference in the mean level in original units comparing each category with the reference or percentage difference in change in original units per year (%/yr) comparing each category with the reference.

For peer review only - http://bmjopen.bmj.com/site/about/guidelines.xhtml

Supplemental Table S16 Mean trajectories of HDL-C and non-HDL-c estimated from multilevel models, by maternal anxiety during pregnancy

	Mean trajectory (95% CI) in males (no anxiety during pregnancy) (reference)	Mean trajectory (95% CI) in females (no anxiety during pregnancy) (reference)	Mean difference in trajectory (95% CI) comparing anxiety at 18 weeks only with the reference trajectory	Mean difference in trajectory (95% CI) comparing anxiety at 32 weeks only with the reference trajectory	Mean difference in trajectory (95% CI) c comparing anxiety at 18 and 32 weeks with the reference trajectory
**HDL-c**					
**Unadjusted**					
Birth (mmol/l)	0.50 (0.49,0.51)	0.55 (0.53,0.56)	0.01 (-0.02,0.04)	0.01 (-0.02,0.04)	-0.003 (-0.03,0.02)
Change 0-7yr (mmol/l/yr)	0.15 (0.15,0.15)	0.13 (0.13,0.13)	-0.005 (-0.01,0.002)	0.0004 (-0.01,0.01)	-0.001 (-0.01,0.004)
Change 7-18yr (mmol/l/yr)	-0.04 (-0.04,-0.03)	-0.01 (-0.01,-0.01)	0.0003 (-0.003,0.004)	-0.002 (-0.01,0.001)	-0.00003 (-0.003,0.003)
Age 18yr (mmol/l)	1.15 (1.14,1.17)	1.33 (1.32,1.35)	-0.02 (-0.05,0.02)	-0.01 (-0.05,0.02)	-0.01 (-0.04,0.02)
**Adjusted**					
Birth (mmol/l)	0.49 (0.46,0.53)	0.54 (0.50,0.57)	0.02 (-0.02,0.05)	0.01 (-0.02,0.04)	0.003 (-0.02,0.03)
Change 0-7yr (mmol/l/yr)	0.15 (0.14,0.16)	0.13 (0.13,0.14)	-0.005 (-0.01,0.001)	0.001 (-0.01,0.01)	-0.002 (-0.01,0.003)
Change 7-18yr (mmol/l/yr)	-0.04 (-0.04,-0.03)	-0.01 (-0.01,-0.01)	0.001 (-0.003,0.004)	-0.001 (-0.005,0.002)	0.001 (-0.002,0.004)
Age 18yr (mmol/l)	1.16 (1.13,1.20)	1.35 (1.31,1.39)	-0.01 (-0.05,0.03)	-0.005 (-0.04,0.03)	0.0005 (-0.03,0.03)
**Non-HDL-c**					
**Unadjusted**					
Birth (mmol/l)	1.23 (1.20,1.26)	1.30 (1.27,1.33)	-0.08 (-0.18,0.01)	0.03 (-0.08,0.13)	-0.02 (-0.10,0.06)
Change 0-9yr (mmol/l/yr)	0.19 (0.18,0.19)	0.21 (0.20,0.21)	0.017 (0.001,0.03)	-0.005 (-0.02,0.01)	-0.002 (-0.01,0.01)
Change 9-18yr (mmol/l/yr)	-0.07 (-0.08,-0.07)	-0.07 (-0.08,-0.07)	0.004 (-0.01,0.02)	0.01 (-0.01,0.02)	0.01 (-0.01,0.02)
Age 18yr (mmol/l)	2.27 (2.23,2.30)	2.49 (2.45,2.52)	0.11 (-0.01,0.22)	0.04 (-0.07,0.15)	0.02 (-0.08,0.12)
**Adjusted**					
Birth (mmol/l)	1.21 (1.13,1.29)	1.27 (1.19,1.35)	-0.09 (-0.19,0.01)	0.03 (-0.08,0.13)	-0.02 (-0.10,0.06)
Change 0-9yr (mmol/l/yr)	0.19 (0.18,0.20)	0.21 (0.20,0.22)	0.02 (0.002,0.03)	-0.004 (-0.02,0.01)	-0.003 (-0.02,0.01)
Change 9-18yr (mmol/l/yr)	-0.07 (-0.08,-0.06)	-0.07 (-0.08,-0.06)	0.002 (-0.01,0.02)	0.01 (-0.01,0.02)	0.005 (-0.01,0.02)
Age 18yr (mmol/l)	2.27 (2.18,2.36)	2.49 (2.40,2.58)	0.09 (-0.02,0.20)	0.04 (-0.07,0.15)	0.001 (-0.10,0.10)

CI, confidence interval; mmol/l, millimole per litre; mmol/l, millimole per litre per year.

For peer review only

For peer review only - http://bmjopen.bmj.com/site/about/guidelines.xhtml

Supplemental Table S17 Mean trajectories of BMI estimated from multilevel models, by maternal depression during pregnancy

	Mean trajectory (95% CI) in males (no depression during pregnancy) log (kg/m^2) (reference) [a]	Mean trajectory (95% CI) in females (no depression during pregnancy) log (kg/m^2) (reference) [a]	Mean difference in trajectory (95% CI) comparing depression at 18 weeks only with the reference trajectory (% difference in kg/m^2) [b]	Mean difference in trajectory (95% CI) depression at 32 weeks only with the reference trajectory (% difference in kg/m^2) [b]	Mean difference in trajectory (95% CI) comparing depression at 18 and 32 weeks with the reference trajectory (% difference in kg/m^2) [b]
**Unadjusted BMI**					
Age 1yr	2.90 (2.89,2.91)	2.89 (2.88,2.89)	0.07 (-1.60,1.75)	-0.33 (-1.79,1.13)	0.3 (-1.32,1.91)
Age 3yr	2.77 (2.76,2.77)	2.75 (2.75,2.76)	0.13 (-0.72,0.98)	0.39 (-0.36,1.15)	-0.5 (-1.34,0.26)
Age 7yr	2.79 (2.78,2.79)	2.80 (2.79,2.80)	0.51 (-0.61,1.62)	0.61 (-0.38,1.59)	0.1 (-0.97,1.11)
Age 9yr	2.82 (2.82,2.83)	2.84 (2.84,2.85)	0.70 (-0.67,2.07)	0.71 (-0.50,1.92)	0.5 (-0.77,1.80)
Age 11yr	2.88 (2.87,2.88)	2.90 (2.90,2.91)	0.88 (-0.69,2.44)	0.85 (-0.53,2.23)	1.0 (-0.48,2.46)
Age 13yr	2.94 (2.94,2.95)	2.97 (2.96,2.98)	1.04 (-0.64,2.72)	1.02 (-0.46,2.50)	1.5 (-0.10,3.07)
Age 15yr	3.02 (3.01,3.02)	3.04 (3.04,3.05)	1.19 (-0.58,2.95)	1.24 (-0.31,2.80)	2.0 (0.31,3.66)
Age 18yr	3.16 (3.15,3.16)	3.17 (3.16,3.18)	1.37 (-0.77,3.50)	1.67 (-0.21,3.55)	2.7 (0.71,4.78)
**Adjusted BMI**					
Age 1yr	2.88 (2.87,2.90)	2.87 (2.86,2.89)	0.04 (-1.66,1.73)	-0.37 (-1.83,1.10)	0.2 (-1.45,1.82)
Age 3yr	2.77 (2.76,2.78)	2.75 (2.74,2.76)	-0.19 (-1.04,0.66)	0.24 (-0.52,0.99)	-0.8 (-1.63,-0.04)
Age 7yr	2.79 (2.78,2.80)	2.80 (2.79,2.81)	-0.15 (-1.22,0.92)	0.29 (-0.66,1.23)	-0.5 (-1.49,0.52)
Age 9yr	2.82 (2.81,2.83)	2.84 (2.83,2.86)	-0.14 (-1.44,1.16)	0.28 (-0.87,1.43)	-0.2 (-1.44,1.00)
Age 11yr	2.87 (2.86,2.89)	2.90 (2.89,2.92)	-0.15 (-1.62,1.32)	0.27 (-1.03,1.57)	0.0 (-1.35,1.43)
Age 13yr	2.94 (2.92,2.95)	2.97 (2.96,2.98)	-0.18 (-1.75,1.39)	0.28 (-1.11,1.66)	0.3 (-1.21,1.76)
Age 15yr	3.02 (3.00,3.03)	3.05 (3.03,3.06)	-0.24 (-1.88,1.40)	0.30 (-1.16,1.75)	0.5 (-1.09,2.03)
Age 18yr	3.16 (3.14,3.18)	3.17 (3.16,3.19)	-0.38 (-2.39,1.63)	0.37 (-1.41,2.15)	0.7 (-1.23,2.61)

CI, confidence interval; yr, year.

[a] BMI is presented in the natural log and values represent the mean predicted natural log of BMI at each age shown.

[b] Differences at each age are back transformed from the log scale and are interpreted as the percentage difference in the mean level in original units at each age comparing each category with the reference trajectory.

For peer review only

Supplemental Table S18 Mean trajectories of fat mass estimated from multilevel models, by maternal depression during pregnancy

	Mean trajectory (95% CI) in males (no depression during pregnancy) log (kg or kg/yr) (reference) [a]	Mean trajectory (95% CI) in females (no depression during pregnancy) log (kg or kg/yr) (reference) [a]	Mean difference in trajectory (95% CI) comparing depression at 18 weeks only with the reference trajectory (% or %/yr) [b]	Mean difference in trajectory (95% CI) depression at 32 weeks only with the reference trajectory (% or %/yr) [b]	Mean difference in trajectory (95% CI) comparing depression at 18 and 32 weeks with the reference trajectory (% or %/yr) [b]
**Unadjusted**					
Age 9yr (kg) or (%)	1.79 (1.76,1.82)	2.64 (2.12,3.16)	2.03 (-5.19,9.26)	-3.60 (-9.47,2.28)	1.61 (-5.11,8.33)
Change 9-13yr (kg/yr) or (%/yr)	0.12 (0.11,0.12)	0.16 (0.15,0.17)	0.90 (-0.60,2.41)	0.77 (-0.52,2.06)	1.86 (0.40,3.32)
Change 13-15yr (kg/yr) or (%/yr)	-0.06 (-0.07,-0.05)	0.10 (0.09,0.11)	-2.44 (-5.07,0.20)	0.22 (-2.08,2.52)	-0.61 (-3.24,2.03)
Change 15-18yr (kg/yr) or (%/yr)	0.10 (0.09,0.10)	0.06 (0.05,0.06)	1.23 (-0.83,3.28)	-0.28 (-2.03,1.48)	-0.44 (-2.43,1.54)
Age 18yr (kg) or (%)	2.43 (2.41,2.46)	3.65 (3.10,4.20)	4.43 (-3.14,12.01)	-0.98 (-7.26,5.30)	6.61 (-0.74,13.96)
**Adjusted**					
Age 9yr (kg) or (%)	1.81 (1.74,1.87)	2.67 (2.14,3.19)	-0.13 (-7.03,6.77)	-3.49 (-9.23,2.25)	-0.21 (-6.66,6.24)
Change 9-13yr (kg/yr) or (%/yr)	0.11 (0.09,0.12)	0.15 (0.14,0.17)	0.56 (-0.94,2.06)	0.37 (-0.92,1.66)	1.33 (-0.12,2.79)
Change 13-15yr (kg/yr) or (%/yr)	-0.07 (-0.09,-0.04)	0.09 (0.07,0.12)	-2.41 (-5.05,0.24)	-0.02 (-2.32,2.29)	-0.78 (-3.42,1.87)
Change 15-18yr (kg/yr) or (%/yr)	0.10 (0.09,0.12)	0.06 (0.04,0.08)	1.25 (-0.82,3.32)	-0.20 (-1.97,1.56)	-0.41 (-2.41,1.59)
Age 18yr (kg) or (%)	2.42 (2.36,2.48)	3.64 (3.09,4.19)	0.96 (-6.17,8.09)	-2.67 (-8.69,3.34)	2.32 (-4.58,9.22)

CI, confidence interval; kg/yr, kilograms per year; %/yr, percentage per year.
[a] Fat mass was transformed using the natural log. All predicted mean values (kg) and rates of change per year (kg/yr) for the reference categories are on the log scale
[b] The difference between groups is back transformed from the log scale for ease of interpretation and is interpreted as the percentage difference in the mean level in original units comparing each category with the reference or percentage difference in change in original units per year (%/yr) comparing each category with the reference.

For peer review only

Supplemental Table S19 Mean trajectories of blood pressure and pulse rate estimated from multilevel models, by maternal depression during pregnancy

	Mean trajectory (95% CI) in males (no depression during pregnancy) (reference)	Mean trajectory (95% CI) in females (no depression during pregnancy) (reference)	Mean difference in trajectory (95% CI) comparing depression at 18 weeks only with the reference trajectory	Mean difference in trajectory (95% CI) comparing depression at 32 weeks only with the reference trajectory	Mean difference in trajectory (95% CI) comparing depression at 18 and 32 weeks with the reference trajectory
**SBP**					
**Unadjusted**					
Age 7yr (mmHg)	97.85 (97.49,98.20)	97.95 (97.59,98.31)	-0.18 (-1.31,0.95)	0.33 (-0.64,1.30)	0.30 (-0.75,1.35)
Change 7-12yr (mmHg/yr)	1.66 (1.57,1.74)	1.87 (1.79,1.96)	0.17 (-0.10,0.44)	-0.13 (-0.36,0.10)	-0.14 (-0.39,0.11)
Change 12-16yr (mmHg/yr)	5.82 (5.67,5.97)	3.81 (3.67,3.96)	0.11 (-0.35,0.58)	0.17 (-0.23,0.56)	0.38 (-0.05,0.82)
Change 16-18yr (mmHg/yr)	-3.98 (-4.32,-3.65)	-5.86 (-6.18,-5.54)	0.48 (-0.55,1.52)	-0.64 (-1.53,0.24)	-0.07 (-1.04,0.91)
Age 18yr (mmHg)	121.44 (120.97,121.91)	110.85 (110.42,111.28)	2.11 (0.67,3.55)	-0.93 (-2.17,0.31)	1.00 (-0.36,2.36)
**Adjusted**					
Age 7yr (mmHg)	98.97 (97.99,99.96)	99.11 (98.11,100.10)	-0.46 (-1.59,0.66)	0.16 (-0.80,1.13)	0.02 (-1.03,1.07)
Change 7-12yr (mmHg/yr)	1.48 (1.24,1.71)	1.70 (1.46,1.94)	0.18 (-0.09,0.45)	-0.12 (-0.35,0.11)	-0.14 (-0.39,0.11)
Change 12-16yr (mmHg/yr)	5.95 (5.55,6.35)	3.94 (3.53,4.35)	0.18 (-0.28,0.65)	0.23 (-0.17,0.63)	0.44 (-0.002,0.87)
Change 16-18yr (mmHg/yr)	-4.20 (-5.11,-3.29)	-6.11 (-7.02,-5.19)	0.23 (-0.80,1.27)	-0.88 (-1.77,0.01)	-0.28 (-1.26,0.70)
Age 18yr (mmHg)	121.76 (120.49,123.03)	111.15 (109.88,112.42)	1.62 (0.17,3.07)	-1.29 (-2.54,-0.05)	0.50 (-0.87,1.87)
**DBP**					
**Unadjusted**					
Age 7yr (mmHg)	56.11 (55.86,56.36)	56.96 (56.70,57.22)	0.24 (-0.58,1.06)	0.44 (-0.26,1.14)	0.31 (-0.45,1.07)
Change 7-12yr (mmHg/yr)	0.14 (0.07,0.21)	0.12 (0.05,0.19)	0.04 (-0.17,0.25)	0.03 (-0.15,0.21)	-0.07 (-0.27,0.12)
Change 12-16yr (mmHg/yr)	2.85 (2.73,2.98)	2.33 (2.20,2.45)	0.06 (-0.34,0.45)	-0.12 (-0.46,0.22)	-0.10 (-0.47,0.27)
Change 16-18yr (mmHg/yr)	-2.55 (-2.83,-2.26)	-1.12 (-1.39,-0.85)	0.17 (-0.69,1.03)	0.02 (-0.72,0.77)	0.70 (-0.12,1.51)
Age 18yr (mmHg)	63.14 (62.80,63.48)	63.76 (62.96,64.56)	0.99 (-0.03,2.02)	0.18 (-0.72,1.07)	0.95 (-0.02,1.91)
**Adjusted**					
Age 7yr (mmHg)	56.83 (56.11,57.54)	57.69 (56.97,58.41)	0.14 (-0.68,0.96)	0.37 (-0.33,1.07)	0.20 (-0.56,0.96)
Change 7-12yr (mmHg/yr)	0.05 (-0.13,0.23)	0.04 (-0.15,0.22)	0.03 (-0.18,0.24)	0.03 (-0.14,0.21)	-0.08 (-0.27,0.12)
Change 12-16yr (mmHg/yr)	2.94 (2.60,3.29)	2.42 (2.07,2.77)	0.15 (-0.24,0.55)	-0.02 (-0.36,0.32)	0.02 (-0.36,0.40)
Change 16-18yr (mmHg/yr)	-2.70 (-3.48,-1.92)	-1.31 (-2.08,-0.53)	-0.09 (-0.96,0.78)	-0.27 (-1.02,0.48)	0.34 (-0.48,1.17)
Age 18yr (mmHg)	63.46 (62.54,64.39)	64.13 (62.95,65.30)	0.73 (-0.30,1.77)	-0.07 (-0.96,0.83)	0.59 (-0.38,1.56)
**Pulse rate**					
**Unadjusted**					
Age 7yr (bpm)	82.49 (82.08,82.90)	85.76 (85.34,86.18)	0.36 (-0.96,1.68)	0.51 (-0.62,1.64)	0.07 (-1.16,1.29)
Change 7-12yr (bpm/yr)	-1.92 (-2.02,-1.82)	-1.76 (-1.86,-1.66)	-0.14 (-0.46,0.18)	0.03 (-0.24,0.30)	0.08 (-0.22,0.38)
Change 12-16yr (bpm/yr)	-0.73 (-0.88,-0.57)	-0.28 (-0.43,-0.12)	0.43 (-0.05,0.92)	0.24 (-0.17,0.65)	0.29 (-0.17,0.74)
Change 16-18yr (bpm/yr)	-4.08 (-4.43,-3.73)	-4.69 (-5.02,-4.36)	-0.65 (-1.72,0.41)	-0.83 (-1.74,0.08)	-0.19 (-1.19,0.81)
Age 18yr (bpm)	61.82 (61.31,62.33)	66.46 (65.99,66.93)	0.07 (-1.49,1.63)	-0.04 (-1.38,1.31)	1.22 (-0.25,2.68)
**Adjusted**					
Age 7yr (bpm)	82.71 (81.56,83.86)	85.94 (84.77,87.11)	0.24 (-1.09,1.56)	0.51 (-0.62,1.64)	0.05 (-1.19,1.28)
Change 7-12yr (bpm/yr)	-1.93 (-2.21,-1.66)	-1.76 (-2.04,-1.48)	-0.11 (-0.43,0.21)	0.04 (-0.23,0.31)	0.08 (-0.22,0.38)
Change 12-16yr (bpm/yr)	-1.05 (-1.47,-0.63)	-0.60 (-1.03,-0.17)	0.42 (-0.07,0.91)	0.21 (-0.21,0.62)	0.26 (-0.19,0.72)
Change 16-18yr (bpm/yr)	-3.61 (-4.56,-2.67)	-4.27 (-5.21,-3.33)	-0.74 (-1.80,0.33)	-0.91 (-1.82,0.01)	-0.29 (-1.29,0.71)
Age 18yr (bpm)	61.63 (60.25,63.01)	66.17 (64.79,67.55)	-0.12 (-1.68,1.44)	-0.26 (-1.61,1.09)	0.93 (-0.54,2.40)

bpm, beats per minute; bpm/yr, beats per minute per year; CI, confidence interval; mmHg, millimetres of mercury; mmHg/yr, millimetres of mercury per year.

For peer review only - http://bmjopen.bmj.com/site/about/guidelines.xhtml

Supplemental Table S20 Mean trajectories of lean mass estimated from multilevel models, by maternal depression during pregnancy

	Mean trajectory (95% CI) in males (no depression during pregnancy) (reference)	Mean trajectory (95% CI) in females (no depression during pregnancy) (reference)	Mean difference in trajectory (95% CI) comparing depression at 18 weeks only with the reference trajectory	Mean difference in trajectory (95% CI) comparing depression at 32 weeks only with the reference trajectory	Mean difference in trajectory (95% CI) comparing depression at 18 and 32 weeks with the reference trajectory
**Unadjusted**					
Age 9yr (kg)	23.89 (23.76,24.02)	20.77 (20.65,20.90)	-0.10 (-0.47,0.26)	-0.39 (-0.70,-0.07)	-0.18 (-0.53,0.17)
Change 9-13yr (kg/yr)	2.31 (2.25,2.37)	3.23 (3.17,3.28)	0.03 (-0.13,0.18)	-0.01 (-0.14,0.13)	-0.03 (-0.17,0.12)
Change 13-15yr (kg/yr)	7.68 (7.59,7.78)	1.57 (1.46,1.68)	-0.05 (-0.35,0.25)	-0.02 (-0.28,0.23)	-0.04 (-0.33,0.26)
Change 15-18yr (kg/yr)	2.47 (2.40,2.55)	0.38 (0.31,0.44)	-0.01 (-0.23,0.21)	0.002 (-0.19,0.19)	0.0005 (-0.21,0.22)
Age 18yr (kg)	55.91 (55.68,56.14)	37.95 (37.72,38.18)	-0.13 (-0.85,0.58)	-0.46 (-1.09,0.17)	-0.35 (-1.03,0.33)
**Adjusted**					
Age 9yr (kg)	23.83 (23.51,24.16)	20.74 (20.42,21.07)	-0.06 (-0.43,0.30)	-0.33 (-0.65,-0.02)	-0.16 (-0.51,0.18)
Change 9-13yr (kg/yr)	2.30 (2.16,2.44)	3.22 (3.08,3.36)	0.0001 (-0.16,0.16)	-0.01 (-0.14,0.13)	-0.04 (-0.18,0.11)
Change 13-15yr (kg/yr)	7.47 (7.21,7.73)	1.35 (1.08,1.62)	-0.03 (-0.33,0.27)	-0.03 (-0.29,0.23)	-0.02 (-0.32,0.27)
Change 15-18yr (kg/yr)	2.64 (2.45,2.83)	0.55 (0.36,0.74)	0.002 (-0.22,0.22)	0.01 (-0.18,0.20)	0.01 (-0.21,0.22)
Age 18yr (kg)	55.90 (55.27,56.53)	37.98 (37.34,38.61)	-0.11 (-0.83,0.60)	-0.40 (-1.02,0.23)	-0.33 (-1.02,0.35)

kg/yr, kilograms per year.

For peer review only

Supplemental Table S21 Mean trajectories of glucose estimated from multilevel models, by maternal depression during pregnancy

	Mean trajectory (95% CI) in males (no depression during pregnancy) (reference)	Mean trajectory (95% CI) in females (no depression during pregnancy) (reference)	Mean difference in trajectory (95% CI) comparing depression at 18 weeks only with the reference trajectory	Mean difference in trajectory (95% CI) comparing depression at 32 weeks only with the reference trajectory	Mean difference in trajectory (95% CI) c comparing depression at 18 and 32 weeks with the reference trajectory
**Unadjusted**					
Age 7yr (mmol/l)	4.20 (4.17,4.22)	4.13 (4.11,4.15)	-0.04 (-0.12,0.04)	-0.05 (-0.11,0.02)	-0.04 (-0.11,0.04)
Change 7-15yr (mmol/l/yr)	0.14 (0.14,0.15)	0.13 (0.13,0.14)	0.001 (-0.01,0.01)	0.002 (-0.01,0.01)	0.01 (-0.002,0.03)
Change 15-18yr (mmol/l/yr)	-0.08 (-0.09,-0.07)	-0.10 (-0.11,-0.09)	0.01 (-0.03,0.05)	- 0.003 (-0.04,0.03)	-0.01 (-0.05,0.03)
Age 18yr (mmol/l)	5.11 (5.09,5.14)	4.89 (4.87,4.92)	-0.01 (-0.08,0.07)	-0.03 (-0.10,0.04)	0.02 (-0.06,0.10)
**Adjusted**					
Age 7yr (mmol/l)	4.21 (4.14,4.28)	4.14 (4.08,4.21)	-0.04 (-0.12,0.03)	-0.04 (-0.11,0.02)	-0.03 (-0.10,0.04)
Change 7-15yr (mmol/l/yr)	0.14 (0.13,0.16)	0.13 (0.12,0.14)	0.001 (-0.01,0.01)	0.002 (-0.01,0.01)	0.01 (-0.002,0.03)
Change 15-18yr (mmol/l/yr)	-0.08 (-0.11,-0.04)	-0.10 (-0.13,-0.06)	0.01 (-0.03,0.05)	-0.001 (-0.04,0.03)	-0.01 (-0.05,0.03)
Age 18yr (mmol/l)	5.13 (5.06,5.20)	4.91 (4.84,4.98)	-0.01 (-0.09,0.06)	-0.03 (-0.10,0.04)	0.02 (-0.06,0.10)

CI, confidence interval; mmol/l, millimole per litre; mmol/l/year, millimoles per litre per year.

For peer review only

Supplemental Table S22 Mean trajectories of insulin and triglyceride estimated from multilevel models, by maternal depression during pregnancy

	Mean trajectory (95% CI) in males (no depression during pregnancy) (reference) [a]	Mean trajectory (95% CI) in females (no depression during pregnancy) (reference) [a]	Mean difference in trajectory (95% CI) comparing depression at 18 weeks only with the reference trajectory (% or %/yr) [b]	Mean difference in trajecto (95% CI) comparing depression at 32 weeks onl with the reference trajecto (% or %/yr) [b]	Mean difference in trajectory (95% CI) comparing depression at 18 and 32 weeks with the reference trajectory (% or %/yr) [b]
**Insulin**					
**Unadjusted**					
Birth (mu/l or %)	1.00 (0.90,1.10)	1.08 (0.99,1.18)	-8.47 (-47.50,30.55)	6.35 (-28.02,40.73)	4.26 (-29.26,37.78)
Change 0-9yr (mu/l/yr or %/yr)	0.04 (0.03,0.06)	0.05 (0.04,0.07)	2.05 (-3.91,8.00)	-0.56 (-5.32,4.20)	-2.07 (-6.87,2.73)
Change 9-15yr (mu/l/yr or %/yr)	0.13 (0.11,0.15)	0.14 (0.12,0.16)	-2.14 (-8.16,3.88)	-3.96 (-9.81,1.89)	3.18 (-3.53,9.89)
Change 15-18yr (mu/l/yr or %/yr)	-0.15 (-0.19,-0.11)	-0.13 (-0.17,-0.09)	5.89 (-7.22,19.00)	7.42 (-5.61,20.46)	-7.27 (-19.75,5.21)
Age 18yr (mu/l or %)	1.75 (1.67,1.84)	1.98 (1.89,2.06)	14.50 (-17.62,46.62)	-1.65 (-26.79,23.49)	-16.92 (-40.13,6.29)
**Adjusted**					
Birth (mu/l or %)	1.05 (0.77,1.32)	1.17 (0.89,1.44)	-8.23 (-47.25,30.78)	4.98 (-28.32,38.28)	14.00 (-22.85,50.85)
Change 0-9yr (mu/l/yr or %/yr)	0.02 (-0.02,0.06)	0.02 (-0.02,0.07)	1.86 (-4.02,7.74)	-0.61 (-5.27,4.06)	-3.06 (-7.79,1.67)
Change 9-15yr (mu/l/yr or %/yr)	0.18 (0.13,0.24)	0.18 (0.13,0.24)	-1.40 (-7.48,4.68)	-3.97 (-9.85,1.91)	2.66 (-3.99,9.30)
Change 15-18yr (mu/l/yr or %/yr)	-0.16 (-0.27,-0.05)	-0.14 (-0.25,-0.03)	5.68 (-7.34,18.69)	9.24 (-4.01,22.49)	-5.79 (-18.31,6.73)
Age 18yr (mu/l or %)	1.82 (1.59,2.05)	2.05 (1.82,2.28)	17.45 (-15.03,49.92)	1.61 (-24.14,27.37)	-15.65 (-38.77,7.47)
**Triglyceride**					
**Unadjusted**					
Birth (mmol/l or %)	-0.71 (-0.73,-0.69)	-0.70 (-0.72,-0.68)	1.36 (-4.96,7.68)	0.21 (-5.58,6.00)	-0.49 (-6.46,5.49)
Change 0-9yr (mmol/l/yr or %/yr)	0.08 (0.08,0.09)	0.09 (0.09,0.09)	0.06 (-0.90,1.03)	-0.09 (-0.95,0.77)	-0.23 (-1.15,0.68)
Change 9-18yr (mmol/l/yr or %/yr)	-0.04 (-0.04,-0.04)	-0.04 (-0.05,-0.04)	-0.46 (-1.33,0.41)	-0.35 (-1.11,0.42)	-0.22 (-1.07,0.63)
Age 18yr (mmol/l or %)	-0.33 (-0.34,-0.31)	-0.29 (-0.31,-0.27)	-2.20 (-7.98,3.59)	-3.67 (-8.77,1.43)	-4.44 (-10.11,1.23)
**Adjusted**					
Birth (mmol/l or %)	-0.66 (-0.72,-0.60)	-0.66 (-0.72,-0.60)	-0.67 (-6.76,5.42)	0.90 (-4.82,6.62)	-0.63 (-6.50,5.24)
Change 0-9yr (mmol/l/yr or %/yr)	0.08 (0.07,0.09)	0.09 (0.08,0.10)	0.24 (-0.72,1.20)	-0.14 (-0.99,0.72)	-0.18 (-1.09,0.73)
Change 9-18yr (mmol/l/yr or %/yr)	-0.04 (-0.05,-0.03)	-0.05 (-0.05,-0.04)	-0.61 (-1.48,0.26)	-0.46 (-1.22,0.31)	-0.41 (-1.26,0.44)
Age 18yr (mmol/l or %)	-0.31 (-0.37,-0.26)	-0.28 (-0.33,-0.22)	-3.93 (-9.63,1.77)	-4.38 (-9.45,0.70)	-5.77 (-11.39,-0.14)

CI, confidence interval; mmol/l, millimole per litre; mmol/l/year, millimoles per litre per year; %/yr, percentage per year

[a] Insulin and triglyceride were transformed using the natural log. All predicted mean values (mmol/l) and rates of change per year (mmol/l/yr) for the reference categories are on the log scale.

[b] The difference between groups is back transformed from the log scale for ease of interpretation and is interpreted as the percentage difference in the mean level in original units comparing each category with the reference or percentage difference in change in original units per year (%/yr) comparing each category with the reference.

For peer review only

For peer review only - http://bmjopen.bmj.com/site/about/guidelines.xhtml

Supplemental Table S23 Mean trajectories of HDL-C and non-HDL-c estimated from multilevel models, by maternal depression during pregnancy

	Mean trajectory (95% CI) in males (no depression during pregnancy) (reference)	Mean trajectory (95% CI) in females (no depression during pregnancy) (reference)	Mean difference in trajectory (95% CI) comparing depression at 18 weeks only with the reference trajectory	Mean difference in trajectory (95% CI) comparing depression at 32 weeks only with the reference trajectory	Mean difference in trajectory (95% CI) comparing depression at 18 and 32 weeks with the reference trajectory
**HDL-c**					
**Unadjusted**					
Birth (mmol/l)	0.50 (0.49,0.52)	0.55 (0.54,0.56)	-0.02 (-0.05,0.02)	-0.04 (-0.07,-0.01)	-0.001 (-0.04,0.03)
Change 0-7yr (mmol/l/yr)	0.15 (0.15,0.15)	0.13 (0.13,0.13)	0.002 (-0.01,0.01)	0.01 (0.001,0.01)	0.0001 (-0.01,0.01)
Change 7-18yr (mmol/l/yr)	-0.04 (-0.04,-0.03)	-0.01 (-0.01,-0.01)	0.001 (-0.003,0.01)	-0.005 (-0.01,-0.001)	-0.001 (-0.01,0.003)
Age 18yr (mmol/l)	1.15 (1.14,1.16)	1.33 (1.32,1.35)	0.01 (-0.03,0.05)	-0.03 (-0.07,0.003)	-0.02 (-0.06,0.03)
**Adjusted**					
Birth (mmol/l)	0.49 (0.46,0.53)	0.54 (0.51,0.58)	-0.01 (-0.04,0.03)	-0.04 (-0.07,-0.01)	0.004 (-0.03,0.04)
Change 0-7yr (mmol/l/yr)	0.15 (0.14,0.16)	0.13 (0.13,0.14)	0.001 (-0.01,0.01)	0.01 (0.001,0.01)	-0.001 (-0.01,0.01)
Change 7-18yr (mmol/l/yr)	-0.04 (-0.04,-0.03)	-0.01 (-0.01,-0.01)	0.002 (-0.002,0.01)	-0.004 (-0.01,0.0002)	-0.00004 (-0.004,0.004)
Age 18yr (mmol/l)	1.01 (0.98,1.05))	1.35 (1.31,1.39)	0.03 (-0.02,0.07)	-0.02 (-0.06,0.01)	-0.001 (-0.04,0.04)
**Non-HDL-c**					
**Unadjusted**					
Birth (mmol/l)	1.22 (1.20,1.25)	1.30 (1.27,1.33)	-0.06 (-0.17,0.05)	0.07 (-0.03,0.17)	-0.04 (-0.15,0.07)
Change 0-9yr (mmol/l/yr)	0.19 (0.19,0.19)	0.20 (0.20,0.21)	0.01 (-0.01,0.03)	-0.01 (-0.03,0.00)	0.005 (-0.01,0.02)
Change 9-18yr (mmol/l/yr)	-0.07 (-0.08,-0.07)	-0.07 (-0.08,-0.07)	0.01 (-0.01,0.03)	-0.002 (-0.02,0.01)	0.005 (-0.01,0.02)
Age 18yr (mmol/l)	2.28 (2.24,2.31)	2.48 (2.45,2.51)	0.11 (-0.03,0.24)	-0.07 (-0.19,0.06)	0.05 (-0.09,0.19)
**Adjusted**					
Birth (mmol/l)	1.20 (1.12,1.28)	1.27 (1.19,1.35)	-0.08 (-0.19,0.04)	0.07 (-0.03,0.18)	-0.03 (-0.15,0.08)
Change 0-9yr (mmol/l/yr)	0.19 (0.18,0.20)	0.21 (0.20,0.22)	0.01 (-0.01,0.03)	-0.01 (-0.03,0.00)	0.004 (-0.01,0.02)
Change 9-18yr (mmol/l/yr)	-0.07 (-0.08,-0.06)	-0.07 (-0.08,-0.06)	0.01 (-0.01,0.02)	-0.003 (-0.02,0.01)	0.004 (-0.01,0.02)
Age 18yr (mmol/l)	2.28 (2.19,2.36)	2.48 (2.39,2.57)	0.09 (-0.05,0.22)	-0.08 (-0.20,0.04)	0.04 (-0.10,0.18)

CI, confidence interval; mmol/l, millimole per litre; mmol/l, millimole per litre per year.

For peer review only

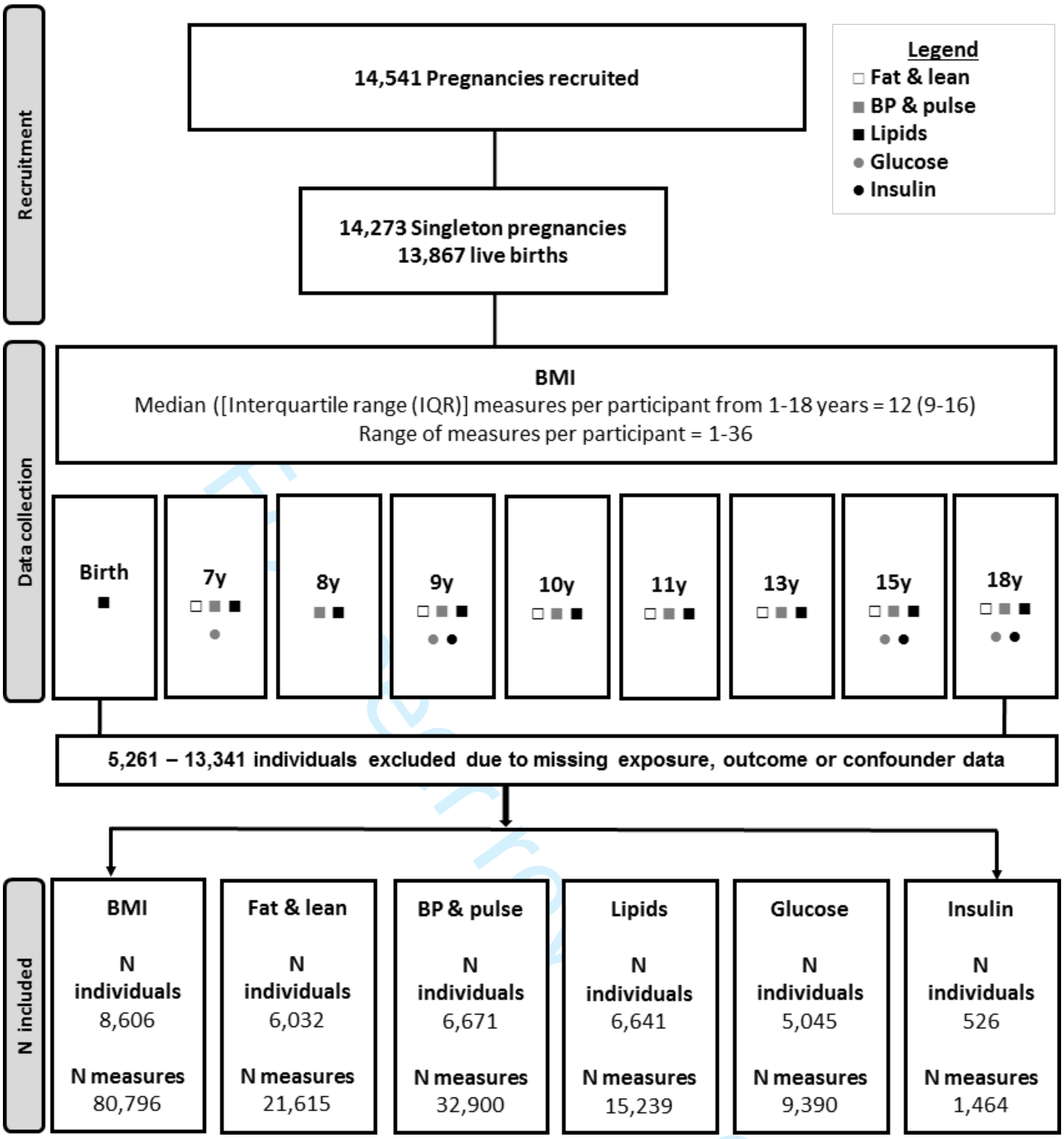

Supplemental Figure S1 Flow diagram of study design

Legend: BP, blood pressure; BMI, body mass index; y, years of age

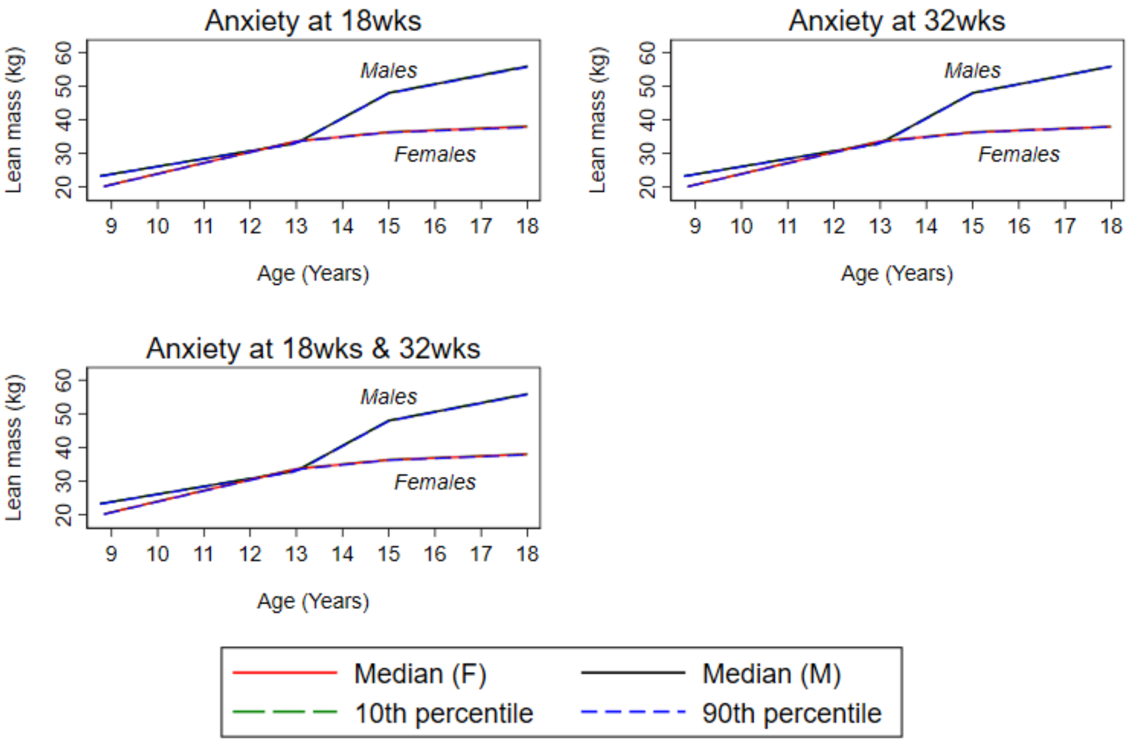

Supplemental Figure S2 Mean predicted confounder adjusted trajectories of lean mass (9 to 18 years), by continuous maternal anxiety levels during pregnancy

Legend: Trajectories are adjusted for maternal age at birth of offspring, parental household socioeconomic position, parity, maternal pre-pregnancy BMI and maternal smoking during pregnancy. Median, 10th and 90th percentile = 1, 4 and 9 at 18 weeks. Median, 10th and 90th percentile = 1, 4, 9 at 32 weeks. Mean of the 18 and 32 week measures used for analyses of anxiety at 18 weeks and 32 weeks; median, 10th and 90th percentile = 1, 4, 9 in this analysis.

review only

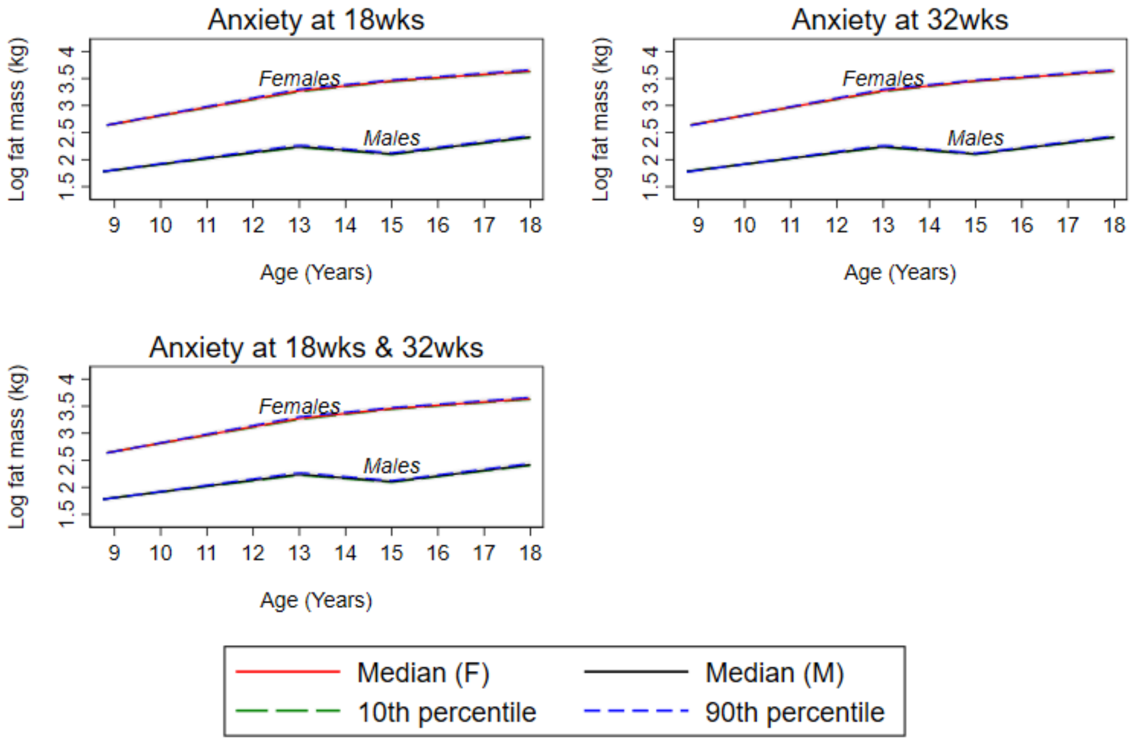

Supplemental Figure S3 Mean predicted confounder adjusted trajectories of log fat mass (9 to 18 years), by continuous maternal anxiety levels during pregnancy

Legend: Trajectories are adjusted for maternal age at birth of offspring, parental household socioeconomic position, parity, maternal pre-pregnancy BMI and maternal smoking during pregnancy. Median, 10th and 90th percentile = 1, 4 and 9 at 18 weeks. Median, 10th and 90th percentile = 1, 4, 10 at 32 weeks. Mean of the 18 and 32 week measures used for analyses of anxiety at 18 weeks and 32 weeks; median, 10th and 90th percentile = 1, 4, 9 in this analysis.

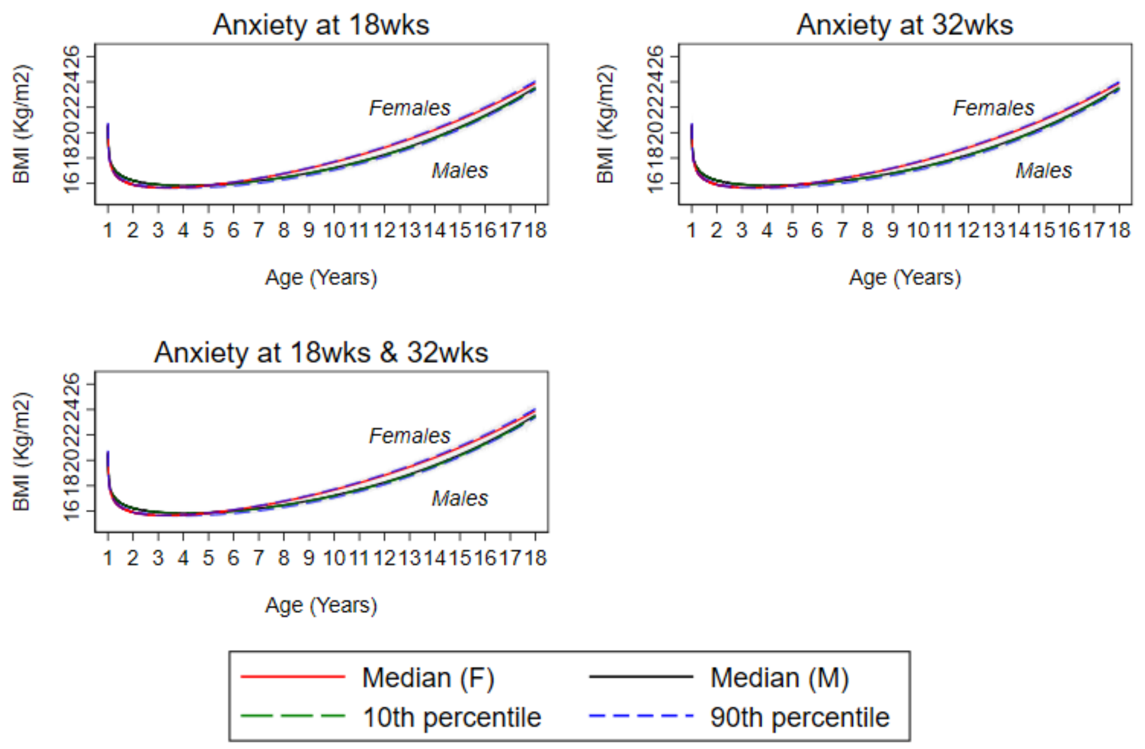

Supplemental Figure S4 Mean predicted confounder adjusted trajectories of log BMI (1 to 18 years), by continuous maternal anxiety levels during pregnancy

Legend: Trajectories are adjusted for maternal age at birth of offspring, parental household socioeconomic position, parity, maternal pre-pregnancy BMI and maternal smoking during pregnancy. Median, 10th and 90th percentile = 1, 4 and 9 at 18 weeks. Median, 10th and 90th percentile = 1, 4, 9 at 32 weeks. Mean of the 18 and 32 week measures used for analyses of anxiety at 18 weeks and 32 weeks; median, 10th and 90th percentile = 1, 4, 9 in this analysis.

For peer review only

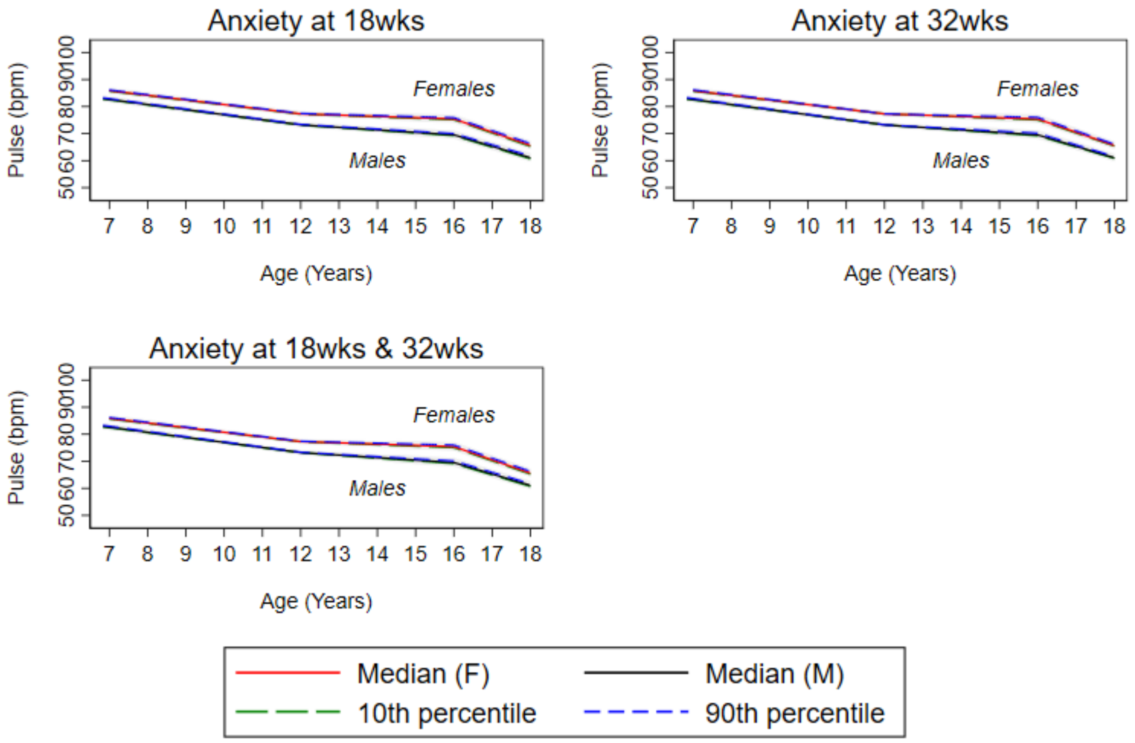

Supplemental Figure S5 Mean predicted confounder adjusted trajectories of pulse rate from 7 to 18 years by continuous maternal anxiety levels during pregnancy

Legend: Trajectories are adjusted for maternal age at birth of offspring, parental household socioeconomic position, parity, maternal pre-pregnancy BMI and maternal smoking during pregnancy. Median, 10th and 90th percentile = 1, 4 and 9 at 18 weeks. Median, 10th and 90th percentile = 1, 4, 10 at 32 weeks. Mean of the 18 and 32 week measures used for analyses of anxiety at 18 weeks and 32 weeks; median, 10th and 90th percentile = 1, 4, 9 in this analysis.

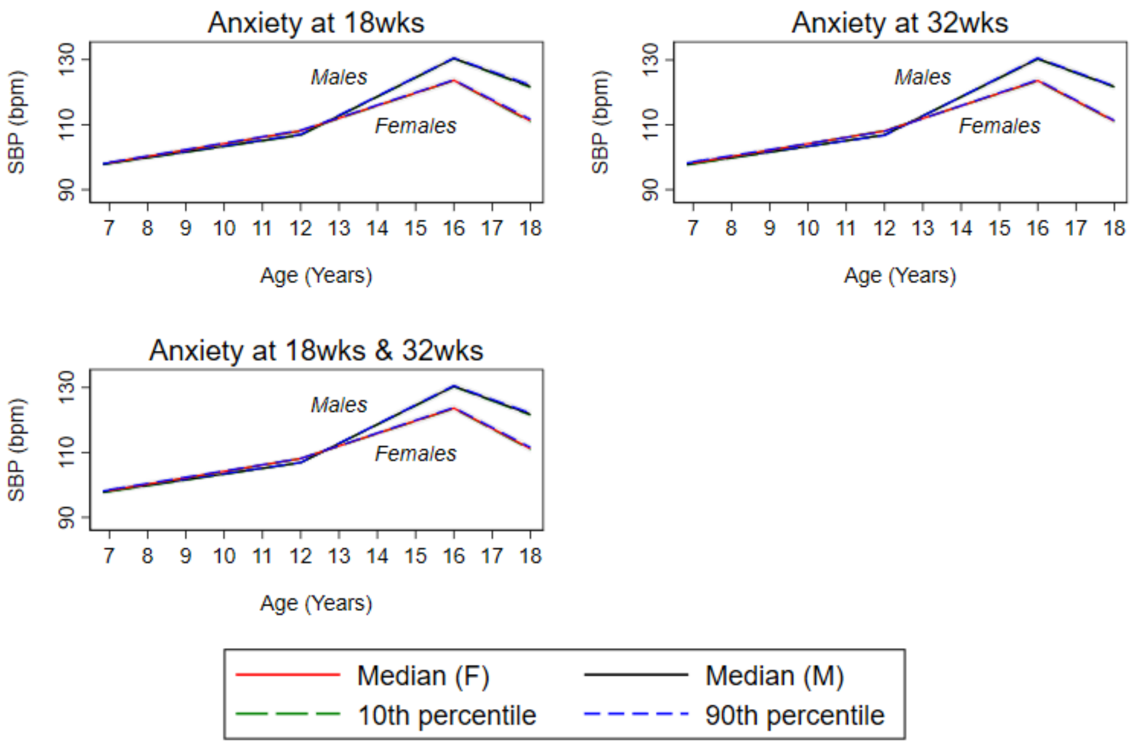

Supplemental Figure S6 Mean predicted confounder adjusted trajectories of SBP from 7 to 18 years by continuous maternal anxiety levels during pregnancy

Legend: Trajectories are adjusted for maternal age at birth of offspring, parental household socioeconomic position, parity, maternal pre-pregnancy BMI and maternal smoking during pregnancy. Median, 10th and 90th percentile = 1, 4 and 9 at 18 weeks. Median, 10th and 90th percentile = 1, 4, 10 at 32 weeks. Mean of the 18 and 32 week measures used for analyses of anxiety at 18 weeks and 32 weeks; median, 10th and 90th percentile = 1, 4, 9 in this analysis.

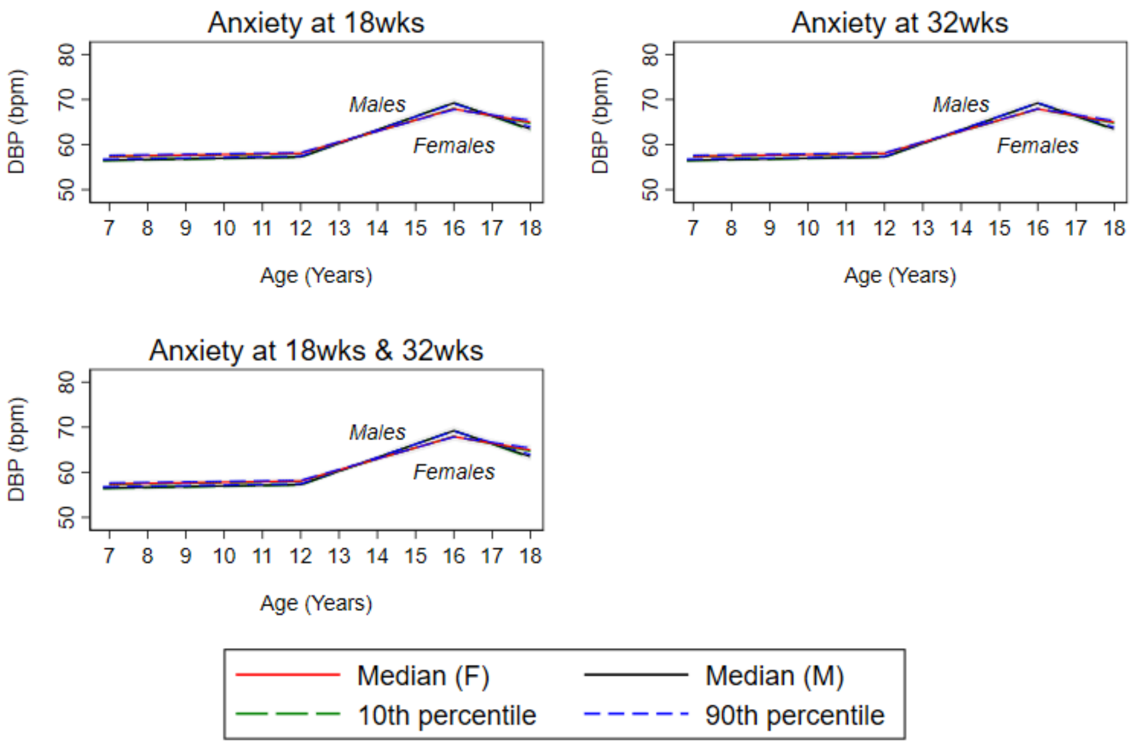

Supplemental Figure S7 Mean predicted confounder adjusted trajectories of DBP from 7 to 18 years by continuous maternal anxiety levels during pregnancy

Legend: Trajectories are adjusted for maternal age at birth of offspring, parental household socioeconomic position, parity, maternal pre-pregnancy BMI and maternal smoking during pregnancy. Median, 10th and 90th percentile = 1, 4 and 9 at 18 weeks. Median, 10th and 90th percentile = 1, 4, 10 at 32 weeks. Mean of the 18 and 32 week measures used for analyses of anxiety at 18 weeks and 32 weeks; median, 10th and 90th percentile = 1, 4, 9 in this analysis.

review only

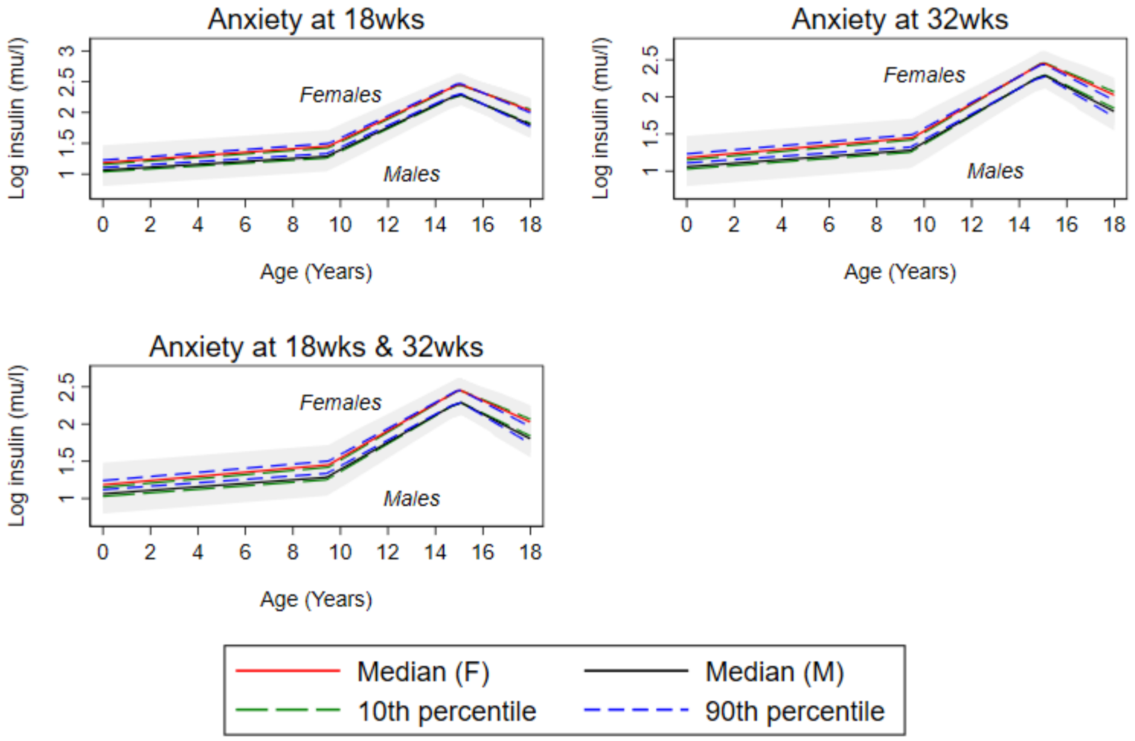

Supplemental Figure S8 Mean predicted confounder adjusted trajectories of log insulin from (birth to 18 years) by continuous maternal anxiety levels during pregnancy

Legend: Trajectories are adjusted for maternal age at birth of offspring, parental household socioeconomic position, parity, maternal pre-pregnancy BMI and maternal smoking during pregnancy. Median, 10th and 90th percentile = 1, 4 and 9 at 18 weeks. Median, 10th and 90th percentile = 1, 4, 9 at 32 weeks. Mean of the 18 and 32 week measures used for analyses of anxiety at 18 weeks and 32 weeks; median, 10th and 90th percentile = 1, 4, 9 in this analysis.

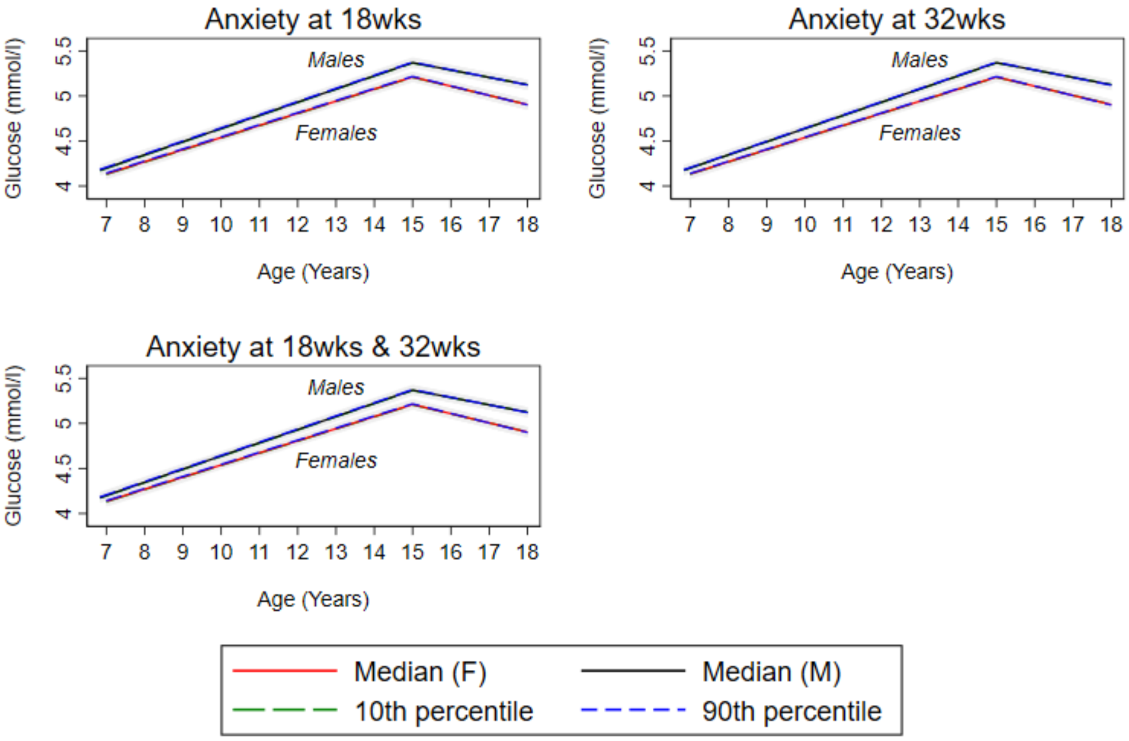

Supplemental Figure S9 Mean predicted confounder adjusted trajectories of glucose (7 to18 years), by continuous maternal anxiety levels during pregnancy

Legend: Trajectories are adjusted for maternal age at birth of offspring, parental household socioeconomic position, parity, maternal pre-pregnancy BMI and maternal smoking during pregnancy. Median, 10th and 90th percentile = 1, 4 and 9 at 18 weeks. Median, 10th and 90th percentile = 1, 4, 10 at 32 weeks. Mean of the 18 and 32 week measures used for analyses of anxiety at 18 weeks and 32 weeks; median, 10th and 90th percentile = 1, 4, 9 in this analysis.

view only

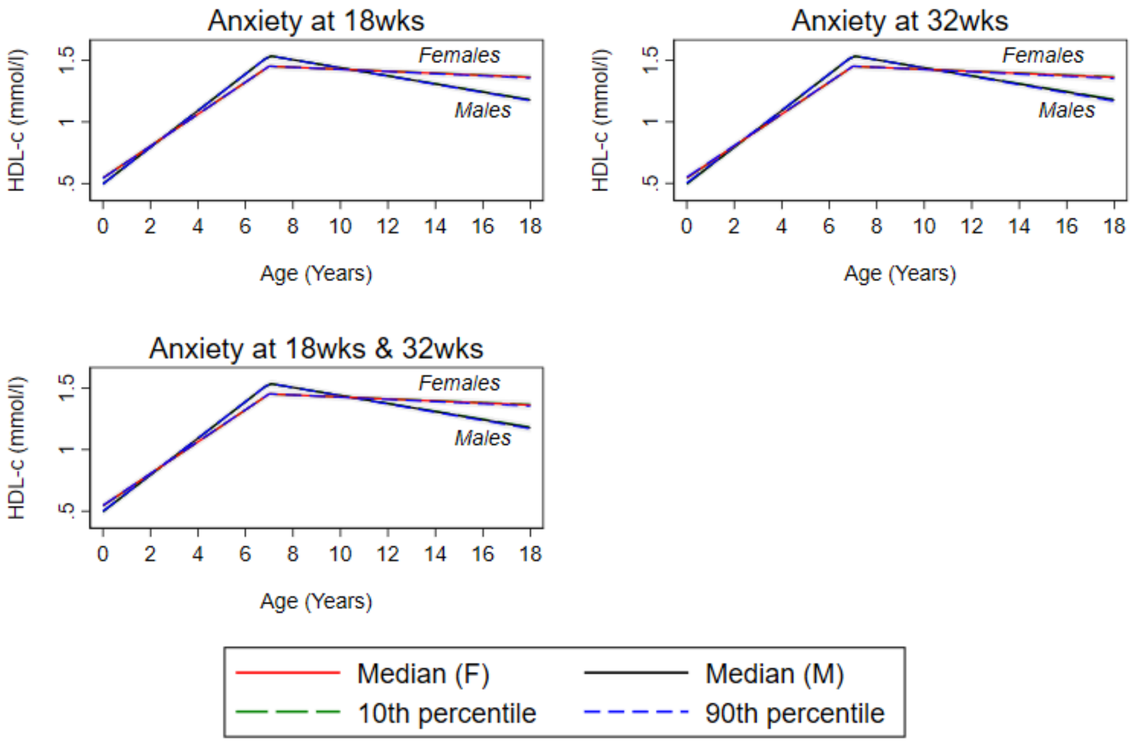

Supplemental Figure S10 Mean predicted confounder adjusted trajectories of HDL-c (birth to18 years), by continuous maternal anxiety levels during pregnancy

Legend: Trajectories are adjusted for maternal age at birth of offspring, parental household socioeconomic position, parity, maternal pre-pregnancy BMI and maternal smoking during pregnancy. Median, 10th and 90th percentile = 1, 4 and 9 at 18 weeks. Median, 10th and 90th percentile = 1, 4, 10 at 32 weeks. Mean of the 18 and 32 week measures used for analyses of anxiety at 18 weeks and 32 weeks; median, 10th and 90th percentile = 1, 4, 9 in this analysis.

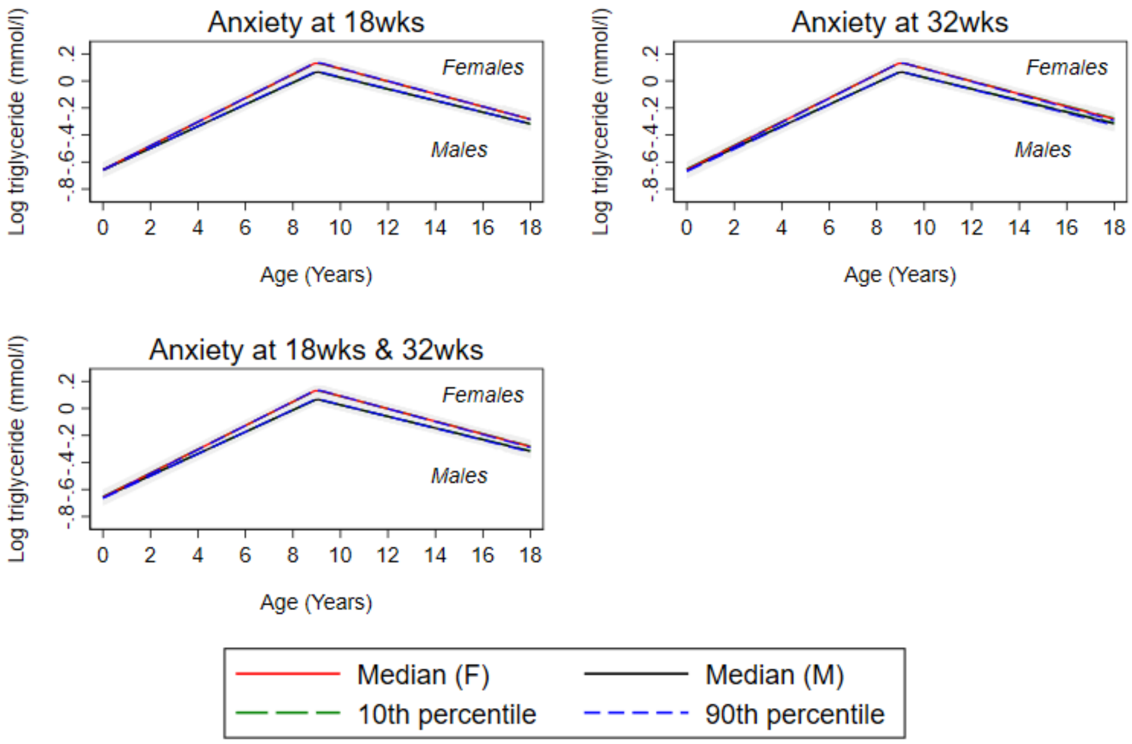

Supplemental Figure S11 Mean predicted confounder adjusted trajectories of triglyceride (birth to18 years), by continuous maternal anxiety levels during pregnancy

Legend: Trajectories are adjusted for maternal age at birth of offspring, parental household socioeconomic position, parity, maternal pre-pregnancy BMI and maternal smoking during pregnancy. Median, 10th and 90th percentile = 1, 4 and 9 at 18 weeks. Median, 10th and 90th percentile = 1, 4, 10 at 32 weeks. Mean of the 18 and 32 week measures used for analyses of anxiety at 18 weeks and 32 weeks; median, 10th and 90th percentile = 1, 4, 9 in this analysis.

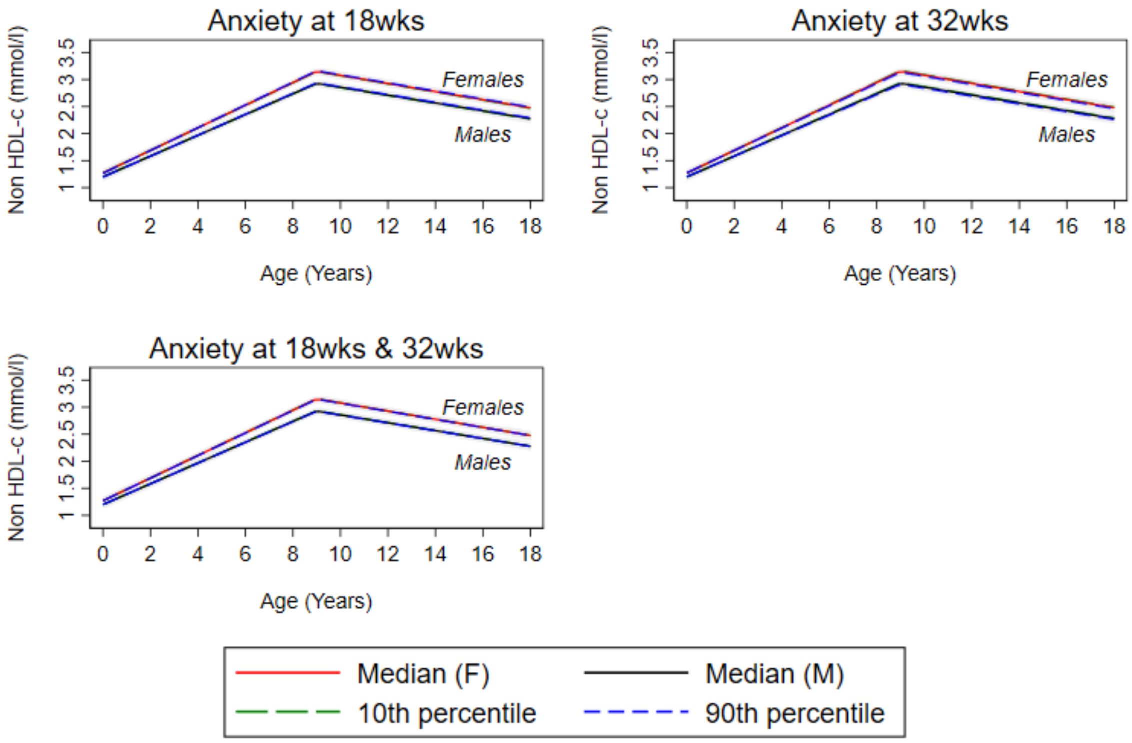

Supplemental Figure S12 Mean predicted confounder adjusted trajectories of non-HDL-c (birth to18 years), by continuous maternal anxiety levels during pregnancy

Legend: Trajectories are adjusted for maternal age at birth of offspring, parental household socioeconomic position, parity, maternal pre-pregnancy BMI and maternal smoking during pregnancy. Median, 10th and 90th percentile = 1, 4 and 9 at 18 weeks. Median, 10th and 90th percentile = 1, 4, 10 at 32 weeks. Mean of the 18 and 32 week measures used for analyses of anxiety at 18 weeks and 32 weeks; median, 10th and 90th percentile = 1, 4, 9 in this analysis.

For peer review only - http://bmjopen.bmj.com/site/about/guidelines.xhtml

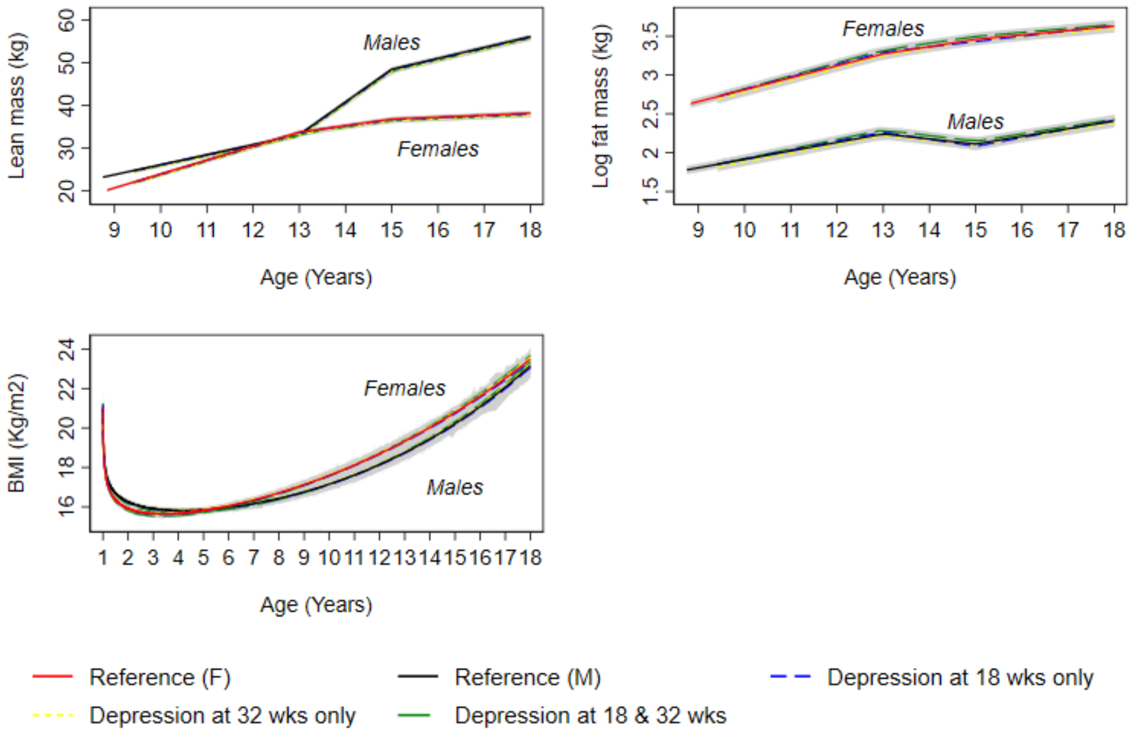

Supplemental Figure S13 Mean predicted confounder adjusted trajectories of lean mass (9 to 18 years), log fat mass (9 to 18 years) and log BMI (1 to 18 years), by maternal depression levels during pregnancy

Legend: Trajectories are adjusted for maternal age at birth of offspring, parental household socioeconomic position, parity, maternal pre-pregnancy BMI and maternal smoking during pregnancy.

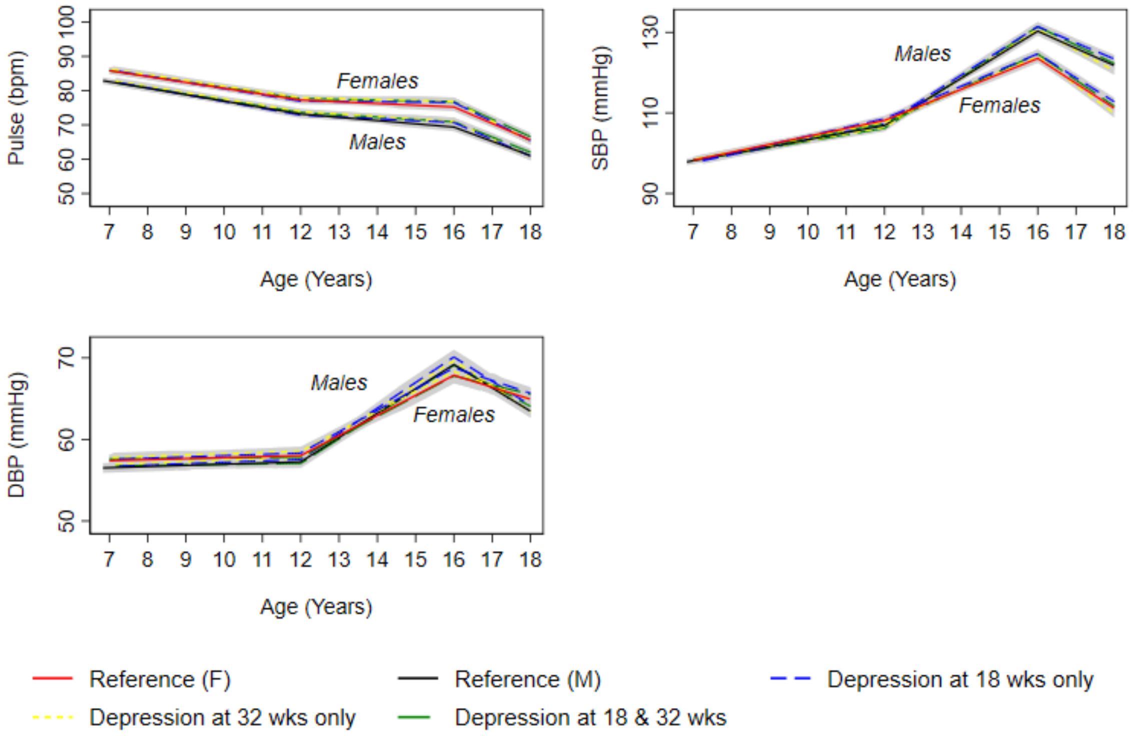

Supplemental Figure S14 Mean predicted confounder adjusted trajectories of pulse rate, SBP and DBP from 7 to 18 years, by maternal depression levels during pregnancy

Legend: DBP, diastolic blood pressure; SBP, systolic blood pressure.

Trajectories are adjusted for maternal age at birth of offspring, parental household socioeconomic position, parity, maternal pre-pregnancy BMI and maternal smoking during pregnancy. Depression at 18 and 32 weeks gestation defined as ≥85th percentile of depression score based on Edinburgh Postnatal Depression Scale for the whole cohort at each time point separately ((≥85th percentile = 13 at both 18 and 32 weeks gestation).

For peer review only

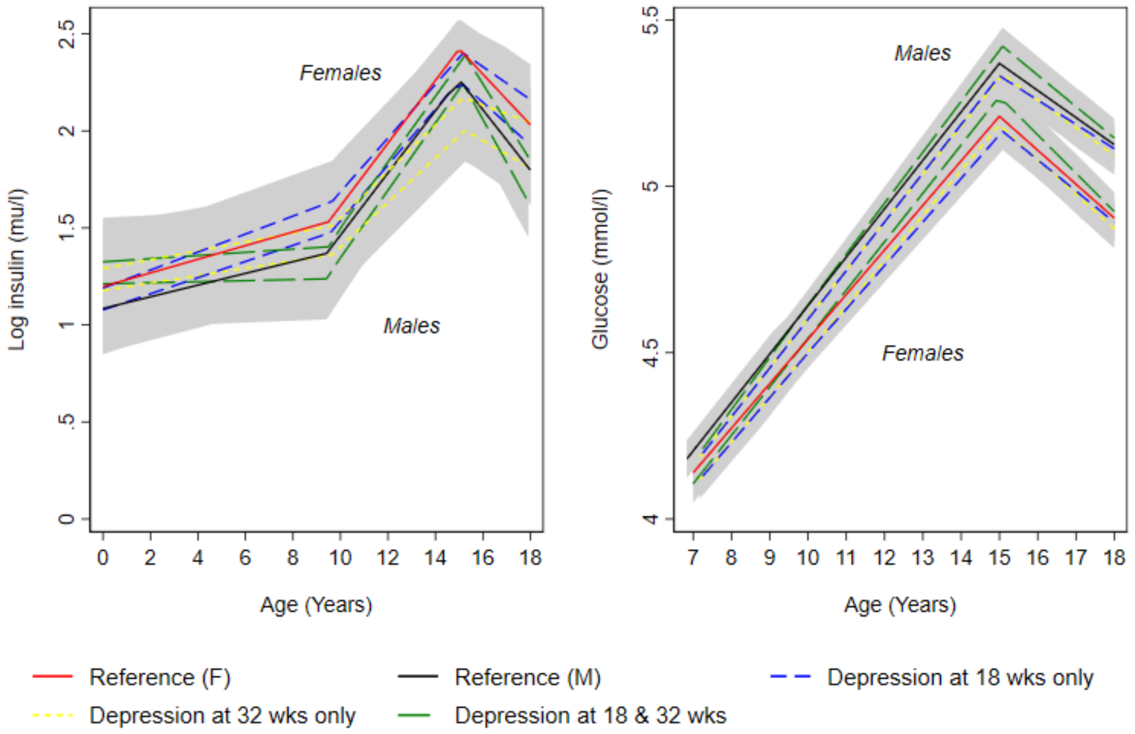

Supplemental Figure S15 Mean predicted confounder adjusted trajectories of log insulin (birth to 18 years) and glucose (7 to 18 years), by maternal depression levels during pregnancy

Legend: Trajectories are adjusted for maternal age at birth of offspring, parental household socioeconomic position, parity, maternal pre-pregnancy BMI and maternal smoking during pregnancy. Depression at 18 and 32 weeks gestation defined as ≥85th percentile of depression score based on Edinburgh Postnatal Depression Scale for the whole cohort at each time point separately ((≥85th percentile = 13 at both 18 and 32 weeks gestation).

For peer review only

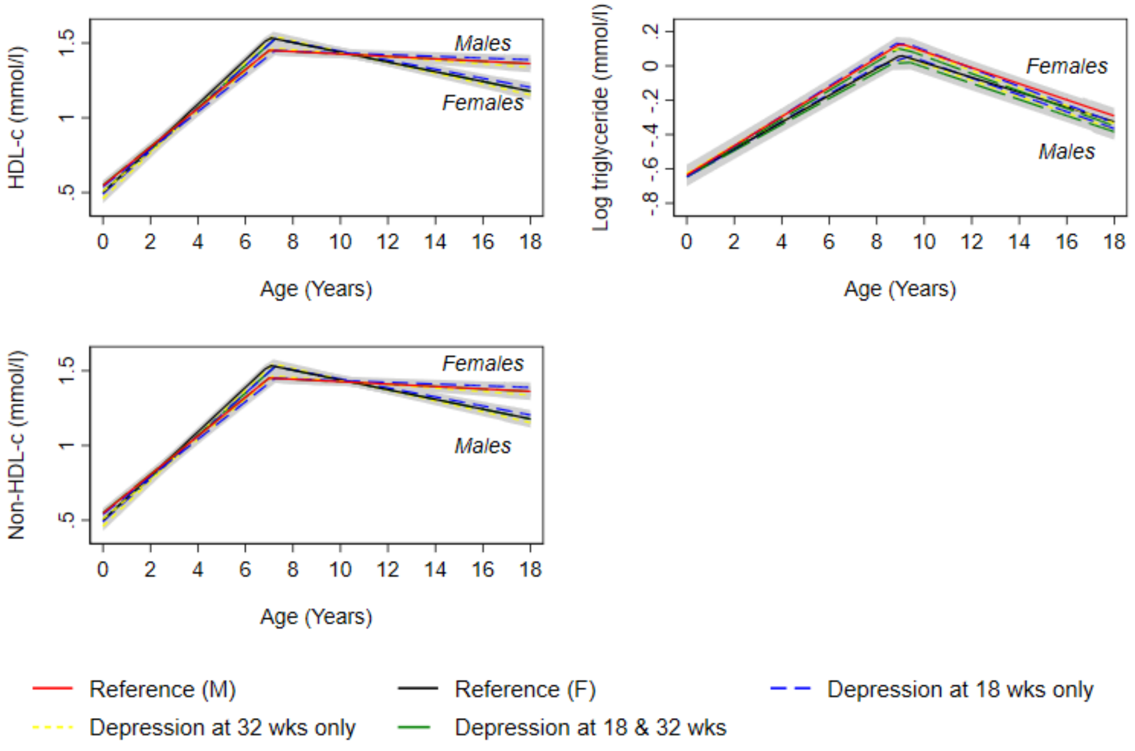

Supplemental Figure S16 Mean predicted confounder adjusted trajectories of HDL-c, log triglyceride and non-HDL-c from birth to 18 years, by maternal depression levels during pregnancy

Legend: Trajectories are adjusted for maternal age at birth of offspring, parental household socioeconomic position, parity, maternal pre-pregnancy BMI and maternal smoking during pregnancy. Depression at 18 and 32 weeks gestation defined as ≥85th percentile of depression score based on Edinburgh Postnatal Depression Scale for the whole cohort at each time point separately ((≥85th percentile = 13 at both 18 and 32 weeks gestation).

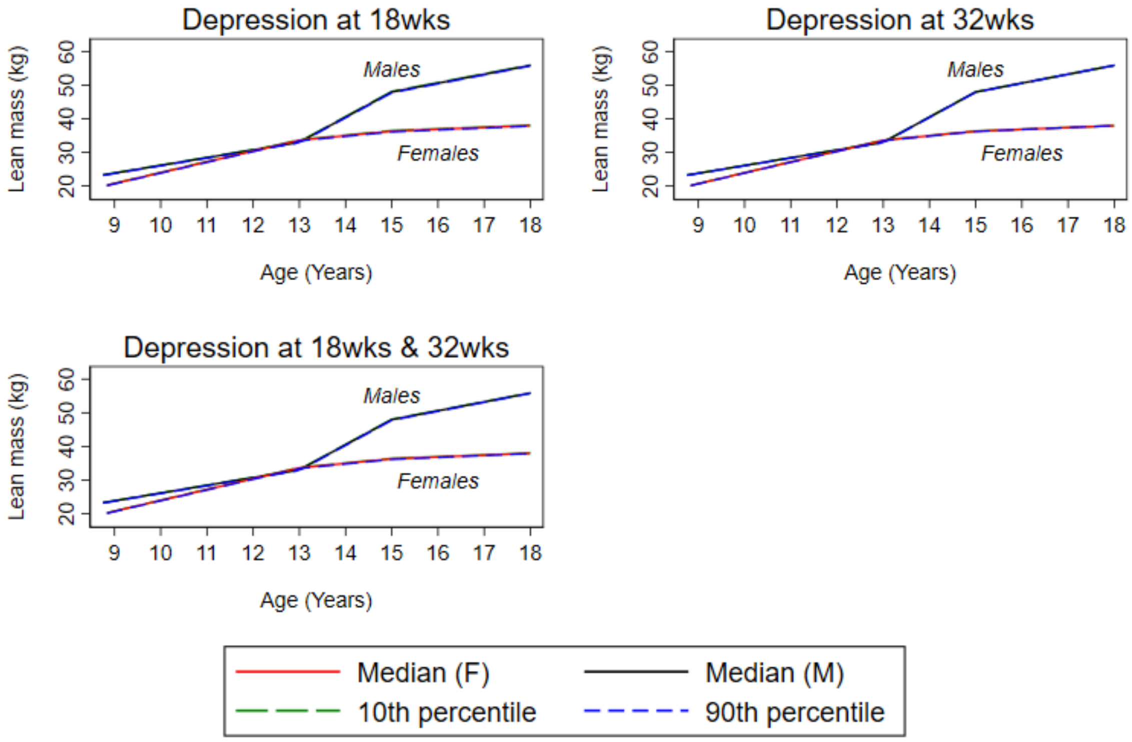

Supplemental Figure S17 Mean predicted confounder adjusted trajectories of lean mass (9 to 18 years), by continuous maternal depression levels during pregnancy

Legend: Trajectories are adjusted for maternal age at birth of offspring, parental household socioeconomic position, parity, maternal pre-pregnancy BMI and maternal smoking during pregnancy. Median, 10th and 90th percentile = 1, 6 and 12 at 18 weeks. Median, 10th and 90th percentile = 1, 6, 13 at 32 weeks. Mean of the 18 and 32 week measures used for analyses of anxiety at 18 weeks and 32 weeks; median, 10th and 90th percentile = 1.5, 5.5, 12 in this analysis.

r review only

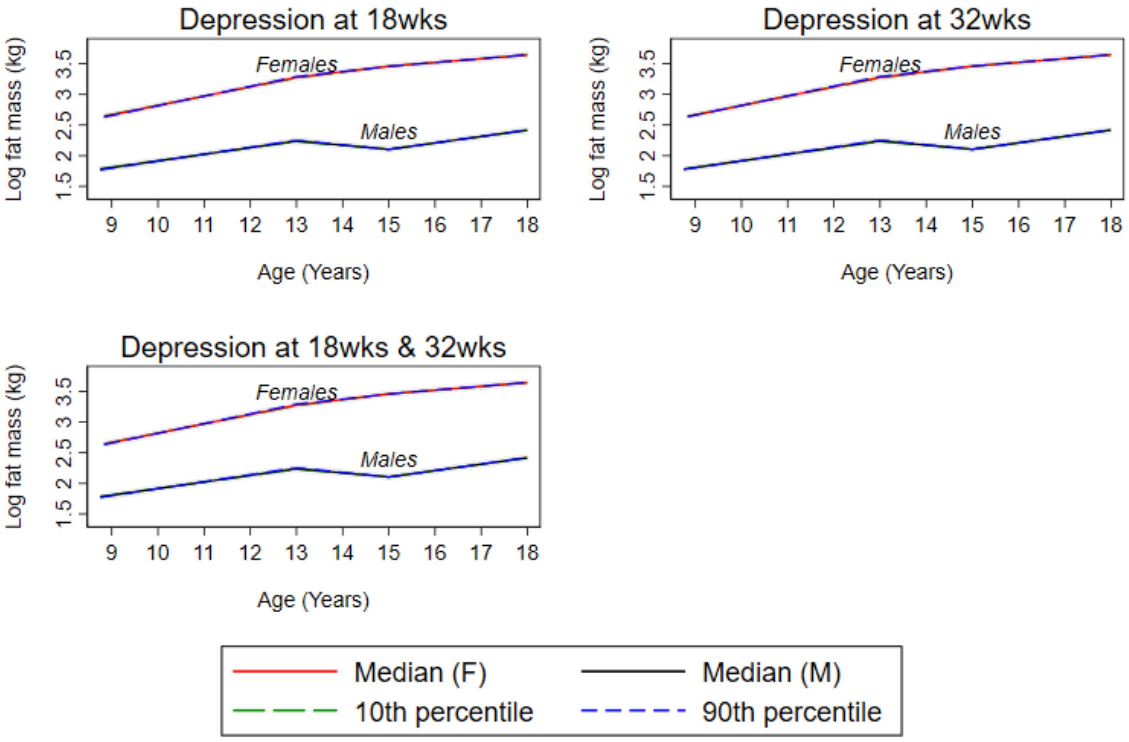

Supplemental Figure S18 Mean predicted confounder adjusted trajectories of log fat mass (9 to 18 years), by continuous maternal depression levels during pregnancy

Legend: Trajectories are adjusted for maternal age at birth of offspring, parental household socioeconomic position, parity, maternal pre-pregnancy BMI and maternal smoking during pregnancy. Median, 10th and 90th percentile = 1, 6 and 12 at 18 weeks. Median, 10th and 90th percentile = 1, 6, 13 at 32 weeks. Mean of the 18 and 32 week measures used for analyses of anxiety at 18 weeks and 32 weeks; median, 10th and 90th percentile = 1.5, 5.5, 12 in this analysis.

For peer review only - http://bmjopen.bmj.com/site/about/guidelines.xhtml

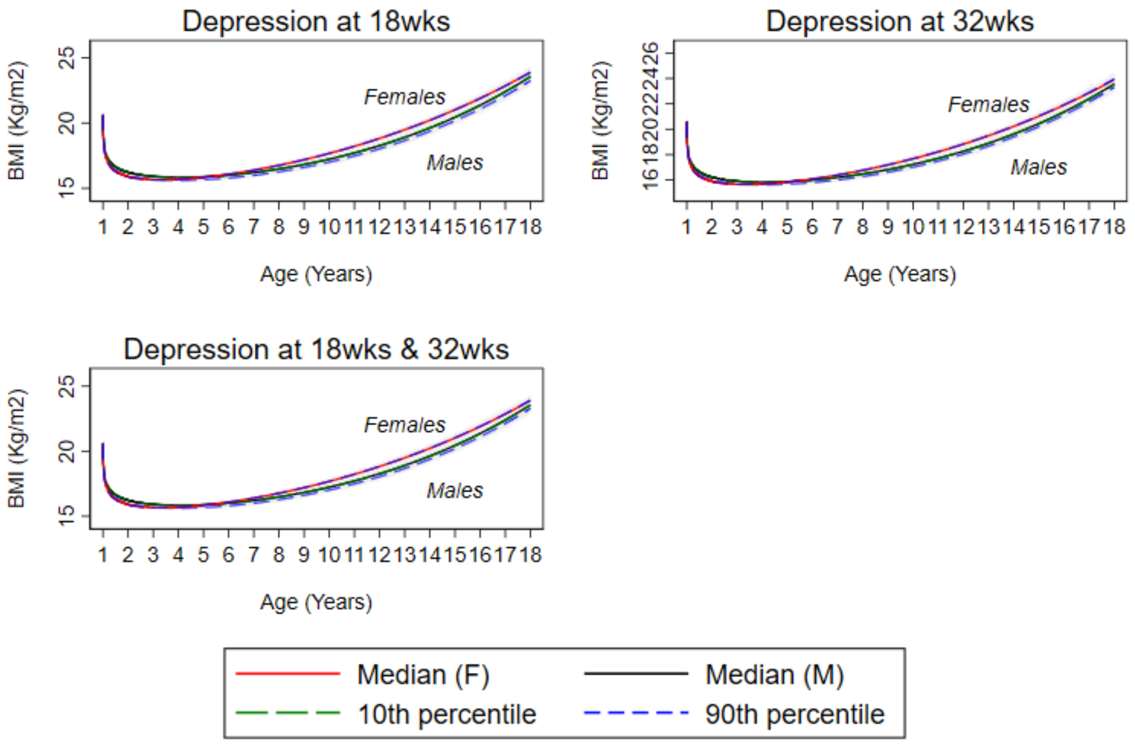

Supplemental Figure S19 Mean predicted confounder adjusted trajectories of log BMI (1 to 18 years), by continuous maternal depression levels during pregnancy

Legend: Trajectories are adjusted for maternal age at birth of offspring, parental household socioeconomic position, parity, maternal pre-pregnancy BMI and maternal smoking during pregnancy. Median, 10th and 90th percentile = 1, 6 and 13 at 18 weeks. Median, 10th and 90th percentile = 1, 6, 13 at 32 weeks. Mean of the 18 and 32 week measures used for analyses of anxiety at 18 weeks and 32 weeks; median, 10th and 90th percentile = 1.5, 5.5, 12 in this analysis.

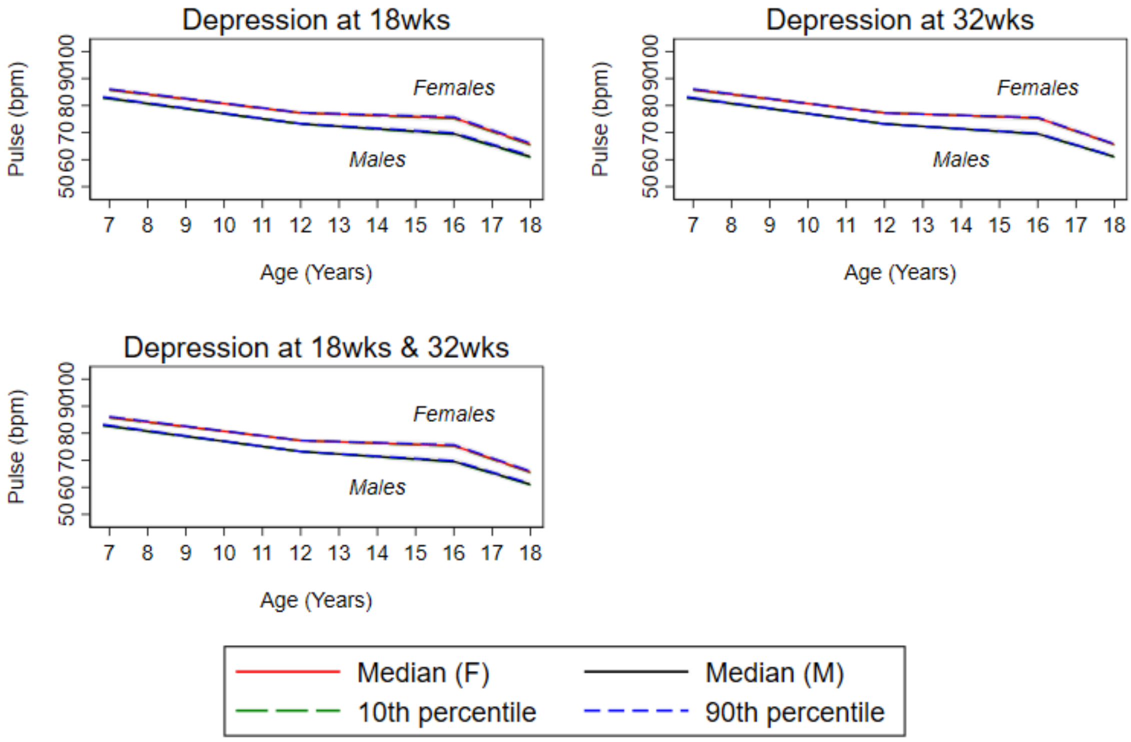

Supplemental Figure S20 Mean predicted confounder adjusted trajectories of pulse rate from 7 to 18 years by continuous maternal depression levels during pregnancy

Legend: Trajectories are adjusted for maternal age at birth of offspring, parental household socioeconomic position, parity, maternal pre-pregnancy BMI and maternal smoking during pregnancy. Median, 10th and 90th percentile = 1, 6 and 13 at 18 weeks. Median, 10th and 90th percentile = 1, 6, 13 at 32 weeks. Mean of the 18 and 32 week measures used for analyses of anxiety at 18 weeks and 32 weeks; median, 10th and 90th percentile = 1.5, 5.5, 12 in this analysis.

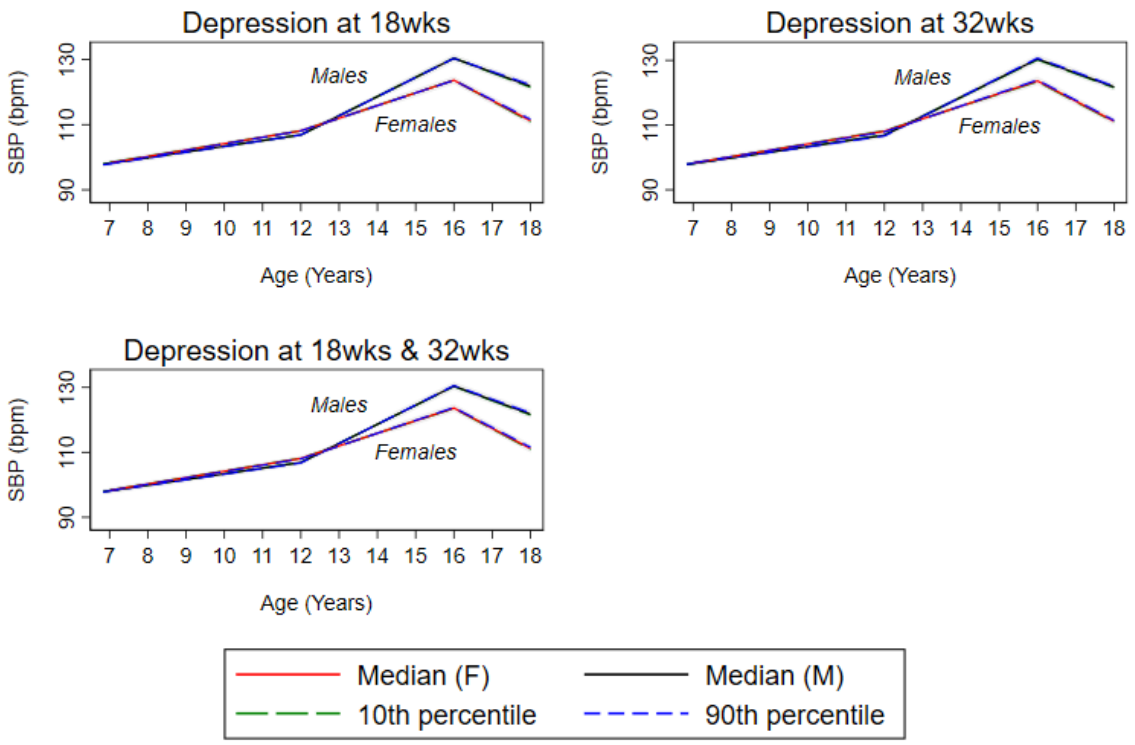

Supplemental Figure S21 Mean predicted confounder adjusted trajectories of SBP from 7 to 18 years by continuous maternal depression levels during pregnancy

Legend: Trajectories are adjusted for maternal age at birth of offspring, parental household socioeconomic position, parity, maternal pre-pregnancy BMI and maternal smoking during pregnancy. Median, 10th and 90th percentile = 1, 6 and 13 at 18 weeks. Median, 10th and 90th percentile = 1, 6, 13 at 32 weeks. Mean of the 18 and 32 week measures used for analyses of anxiety at 18 weeks and 32 weeks; median, 10th and 90th percentile = 1.5, 5.5, 12 in this analysis.

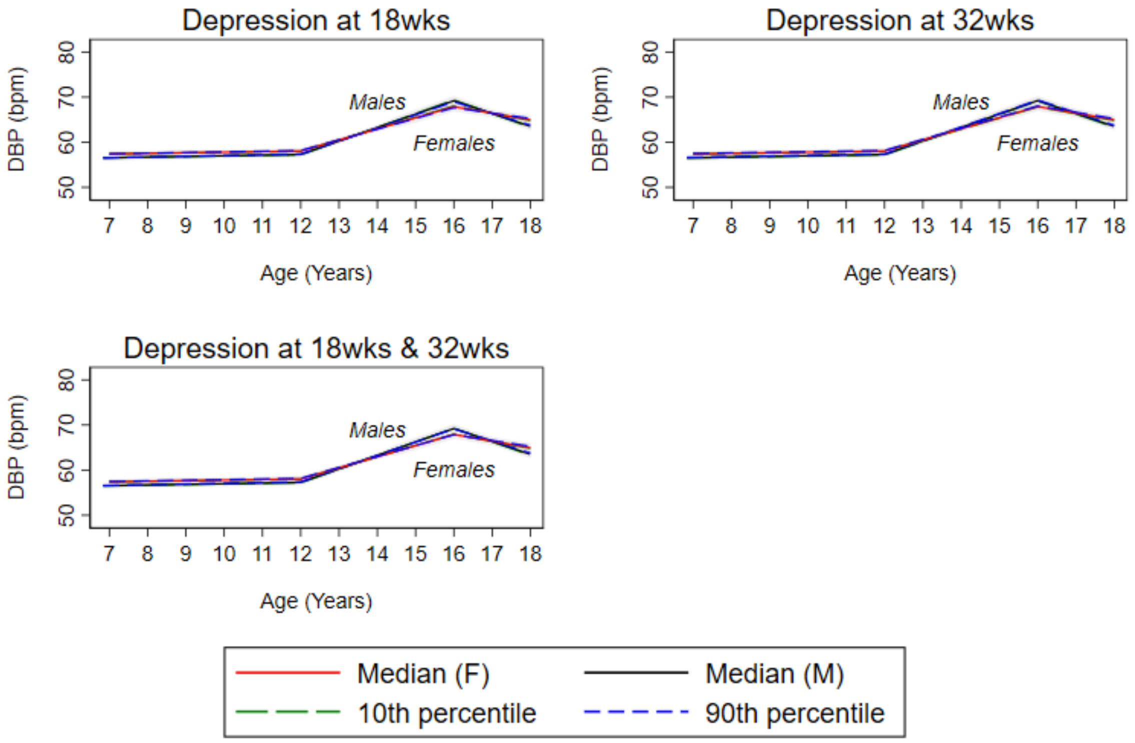

Supplemental Figure S22 Mean predicted confounder adjusted trajectories of DBP from 7 to 18 years by continuous maternal depression levels during pregnancy

Legend: Trajectories are adjusted for maternal age at birth of offspring, parental household socioeconomic position, parity, maternal pre-pregnancy BMI and maternal smoking during pregnancy. Median, 10th and 90th percentile = 1, 6 and 13 at 18 weeks. Median, 10th and 90th percentile = 1, 6, 13 at 32 weeks. Mean of the 18 and 32 week measures used for analyses of anxiety at 18 weeks and 32 weeks; median, 10th and 90th percentile = 1.5, 5.5, 12 in this analysis.

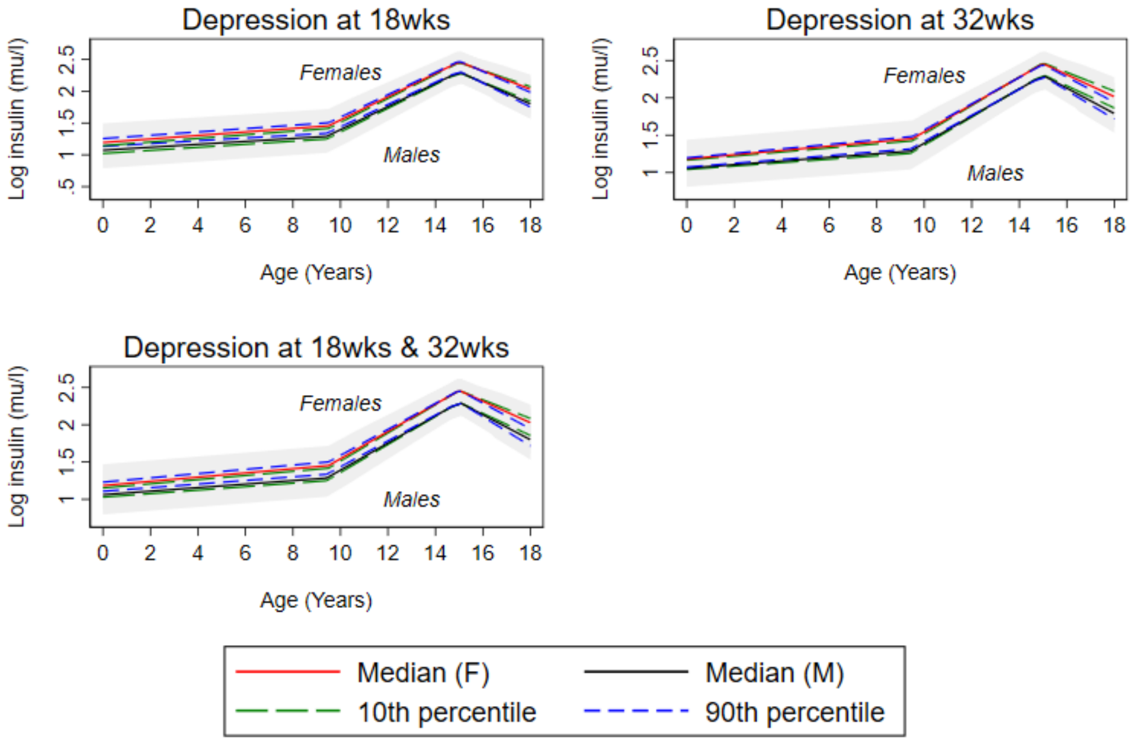

Supplemental Figure S23 Mean predicted confounder adjusted trajectories of log insulin from (birth to 18 years) by continuous maternal depression levels during pregnancy

Legend: Trajectories are adjusted for maternal age at birth of offspring, parental household socioeconomic position, parity, maternal pre-pregnancy BMI and maternal smoking during pregnancy. Median, 10th and 90th percentile = 1, 5 and 13 at 18 weeks. Median, 10th and 90th percentile = 0, 6, 13 at 32 weeks. Mean of the 18 and 32 week measures used for analyses of anxiety at 18 weeks and 32 weeks; median, 10th and 90th percentile = 1, 5.5, 12 in this analysis.

view only

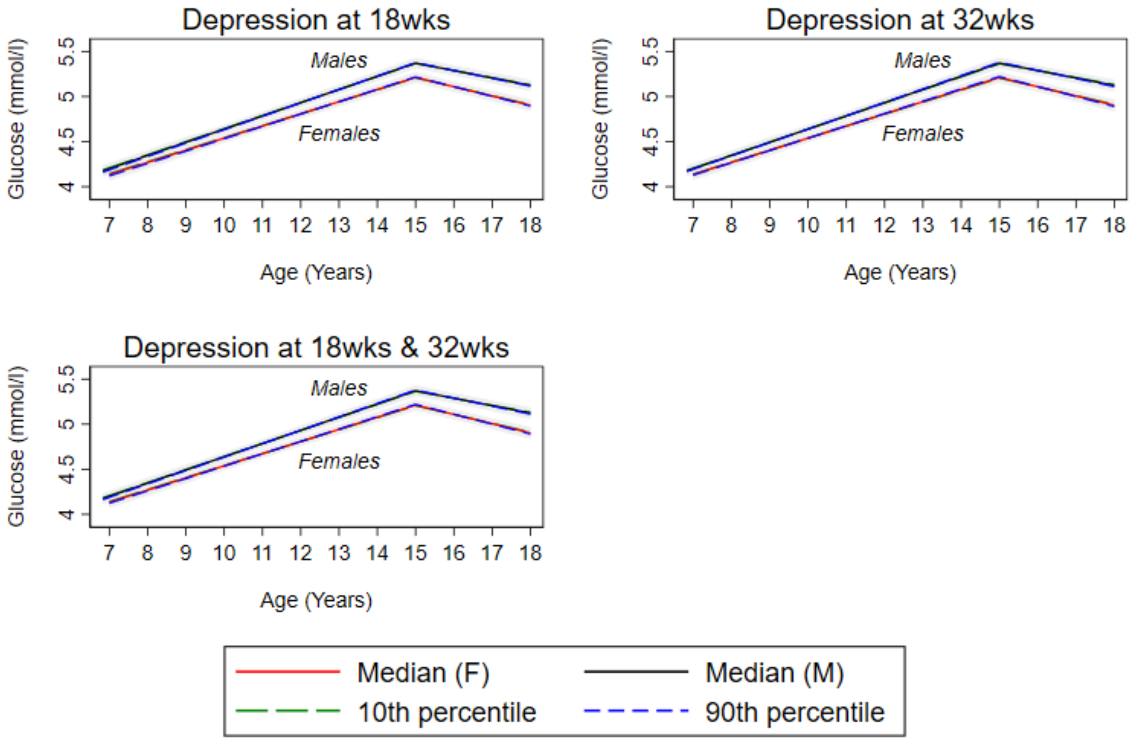

Supplemental Figure S24 Mean predicted confounder adjusted trajectories of glucose (7 to18 years), by continuous maternal depression levels during pregnancy

Legend: Trajectories are adjusted for maternal age at birth of offspring, parental household socioeconomic position, parity, maternal pre-pregnancy BMI and maternal smoking during pregnancy. Median, 10th and 90th percentile = 1, 6 and 13 at 18 weeks. Median, 10th and 90th percentile = 1, 6, 13 at 32 weeks. Mean of the 18 and 32 week measures used for analyses of anxiety at 18 weeks and 32 weeks; median, 10th and 90th percentile = 1.5, 5.5, 12 in this analysis.

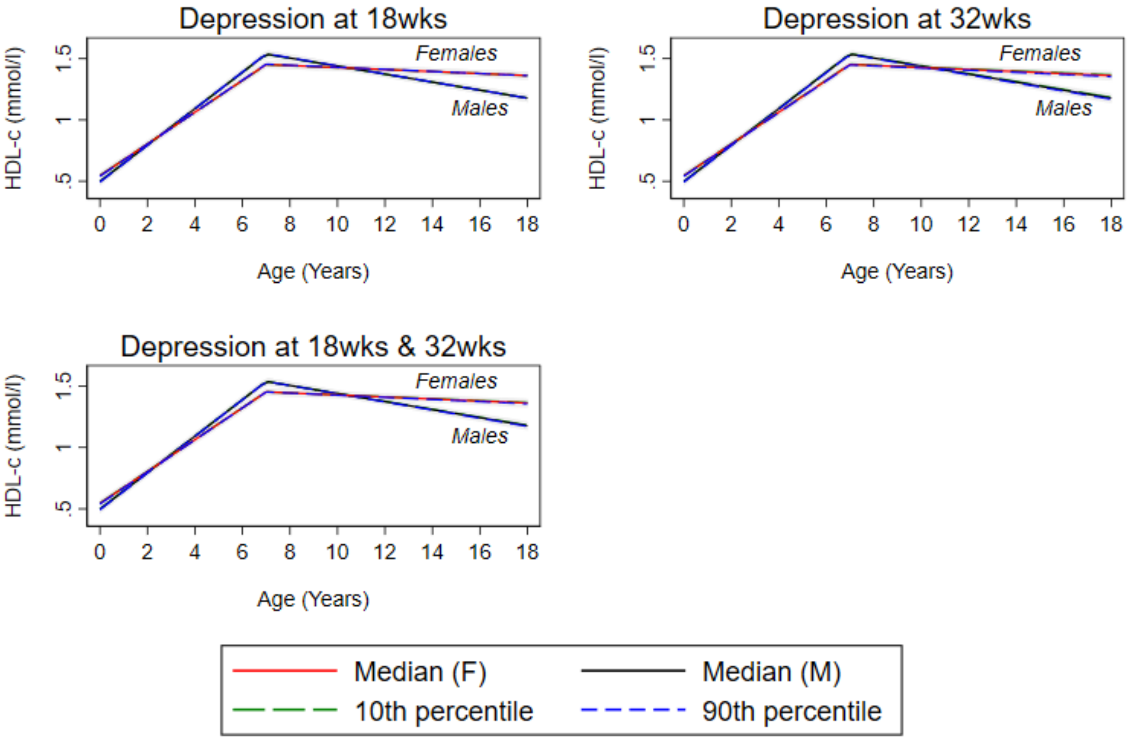

Supplemental Figure S25 Mean predicted confounder adjusted trajectories of HDL-c (birth to18 years), by continuous maternal depression levels during pregnancy

Legend: Trajectories are adjusted for maternal age at birth of offspring, parental household socioeconomic position, parity, maternal pre-pregnancy BMI and maternal smoking during pregnancy. Median, 10th and 90th percentile = 1, 6 and 13 at 18 weeks. Median, 10th and 90th percentile = 1, 6, 13 at 32 weeks. Mean of the 18 and 32 week measures used for analyses of anxiety at 18 weeks and 32 weeks; median, 10th and 90th percentile = 1.5, 5.5, 12.5 in this analysis.

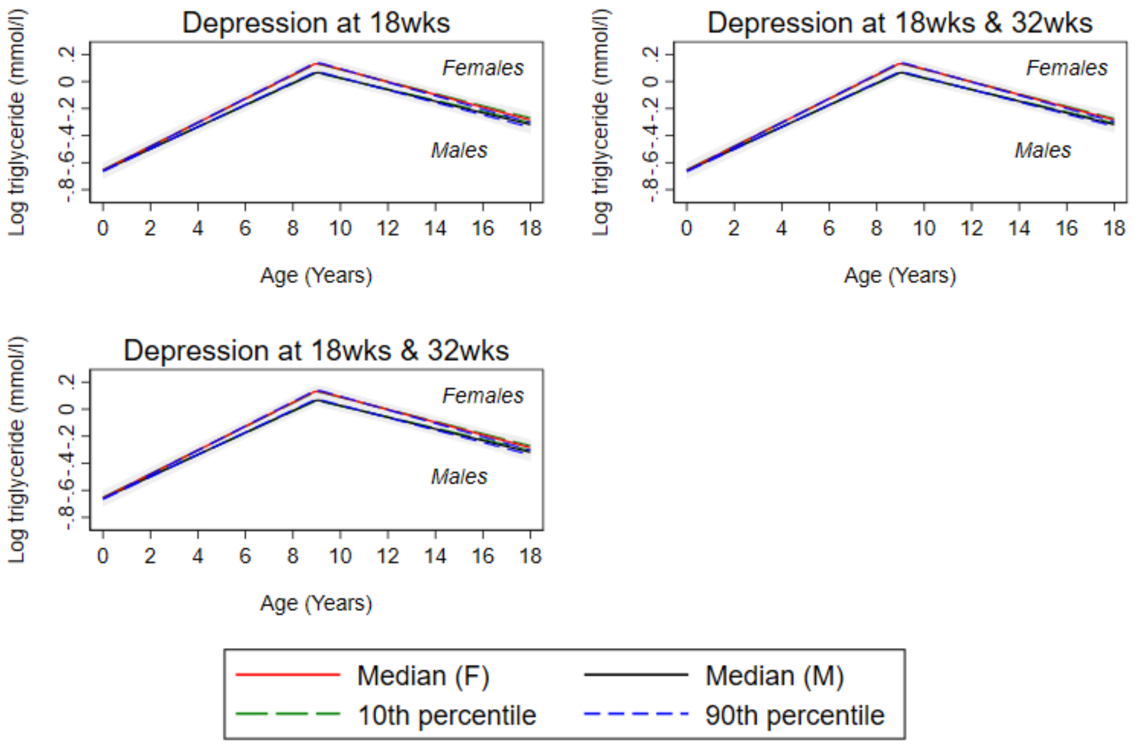

Supplemental Figure S26 Mean predicted confounder adjusted trajectories of triglyceride (birth to18 years), by continuous maternal depression levels during pregnancy

Legend: Trajectories are adjusted for maternal age at birth of offspring, parental household socioeconomic position, parity, maternal pre-pregnancy BMI and maternal smoking during pregnancy. Median, 10th and 90th percentile = 1, 6 and 13 at 18 weeks. Median, 10th and 90th percentile = 1, 6, 13 at 32 weeks. Mean of the 18 and 32 week measures used for analyses of anxiety at 18 weeks and 32 weeks; median, 10th and 90th percentile = 1.5, 6, 12.5 in this analysis.

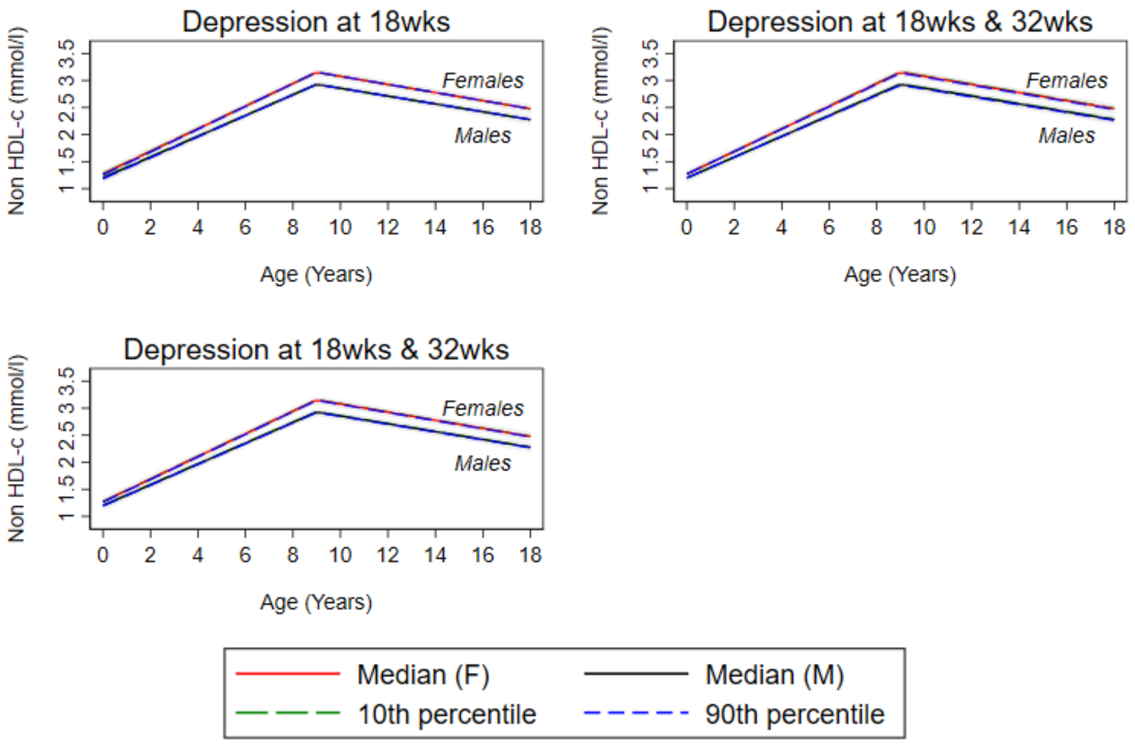

Supplemental Figure S27 Mean predicted confounder adjusted trajectories of non-HDL-c (birth to18 years), by continuous maternal depression levels during pregnancy
Legend: Trajectories are adjusted for maternal age at birth of offspring, parental household socioeconomic position, parity, maternal pre-pregnancy BMI and maternal smoking during pregnancy. Median, 10th and 90th percentile = 1, 6 and 13 at 18 weeks. Median, 10th and 90th percentile = 1, 6, 13 at 32 weeks. Mean of the 18 and 32 week measures used for analyses of anxiety at 18 weeks and 32 weeks; median, 10th and 90th percentile = 1.5, 6, 12.5 in this analysis.

STROBE Statement—Checklist of items that should be included in reports of ***cohort studies***

	Item No	Recommendation	Page No
**Title and abstract**	1	(*a*) Indicate the study's design with a commonly used term in the title or the abstract<br>(*b*) Provide in the abstract an informative and balanced summary of what was done and what was found	1
**Introduction**			
Background/rationale	2	Explain the scientific background and rationale for the investigation being reported	4-5
Objectives	3	State specific objectives, including any prespecified hypotheses	5
**Methods**			
Study design	4	Present key elements of study design early in the paper	6
Setting	5	Describe the setting, locations, and relevant dates, including periods of recruitment, exposure, follow-up, and data collection	6
Participants	6	(*a*) Give the eligibility criteria, and the sources and methods of selection of participants. Describe methods of follow-up<br>(*b*) For matched studies, give matching criteria and number of exposed and unexposed	6
Variables	7	Clearly define all outcomes, exposures, predictors, potential confounders, and effect modifiers. Give diagnostic criteria, if applicable	6-9
Data sources/ measurement	8*	For each variable of interest, give sources of data and details of methods of assessment (measurement). Describe comparability of assessment methods if there is more than one group	6-9
Bias	9	Describe any efforts to address potential sources of bias	n/a
Study size	10	Explain how the study size was arrived at	6
Quantitative variables	11	Explain how quantitative variables were handled in the analyses. If applicable, describe which groupings were chosen and why	9-11
Statistical methods	12	(*a*) Describe all statistical methods, including those used to control for confounding<br>(*b*) Describe any methods used to examine subgroups and interactions<br>(*c*) Explain how missing data were addressed<br>(*d*) If applicable, explain how loss to follow-up was addressed<br>(*e*) Describe any sensitivity analyses	9-11
**Results**			
Participants	13*	(a) Report numbers of individuals at each stage of study—eg numbers potentially eligible, examined for eligibility, confirmed eligible, included in the study, completing follow-up, and analysed<br>(b) Give reasons for non-participation at each stage<br>(c) Consider use of a flow diagram	12-13
Descriptive data	14*	(a) Give characteristics of study participants (eg demographic, clinical, social) and information on exposures and potential confounders<br>(b) Indicate number of participants with missing data for each variable of interest<br>(c) Summarise follow-up time (eg, average and total amount)	12-13
Outcome data	15*	Report numbers of outcome events or summary measures over time	12

For peer review only

For peer review only - http://bmjopen.bmj.com/site/about/guidelines.xhtml

Main results	16	(*a*) Give unadjusted estimates and, if applicable, confounder-adjusted estimates and their precision (eg, 95% confidence interval). Make clear which confounders were adjusted for and why they were included<br>(*b*) Report category boundaries when continuous variables were categorized<br>(*c*) If relevant, consider translating estimates of relative risk into absolute risk for a meaningful time period	12-16
Other analyses	17	Report other analyses done—eg analyses of subgroups and interactions, and sensitivity analyses	Supplementary files
**Discussion**			
Key results	18	Summarise key results with reference to study objectives	17
Limitations	19	Discuss limitations of the study, taking into account sources of potential bias or imprecision. Discuss both direction and magnitude of any potential bias	18
Interpretation	20	Give a cautious overall interpretation of results considering objectives, limitations, multiplicity of analyses, results from similar studies, and other relevant evidence	17-19
Generalisability	21	Discuss the generalisability (external validity) of the study results	18-19
**Other information**			
Funding	22	Give the source of funding and the role of the funders for the present study and, if applicable, for the original study on which the present article is based	20

*Give information separately for exposed and unexposed groups.

Note: An Explanation and Elaboration article discusses each checklist item and gives methodological background and published examples of transparent reporting. The STROBE checklist is best used in conjunction with this article (freely available on the Web sites of PLoS Medicine at http://www.plosmedicine.org/, Annals of Internal Medicine at http://www.annals.org/, and Epidemiology at http://www.epidem.com/). Information on the STROBE Initiative is available at http://www.strobe-statement.org.

For peer review only

BMJ Open

Maternal prenatal anxiety and depression and trajectories of cardiometabolic risk factors across childhood and adolescence: a prospective cohort study

Journal:	*BMJ Open*
Manuscript ID	bmjopen-2021-051681.R1
Article Type:	Original research
Date Submitted by the Author:	02-Nov-2021
Complete List of Authors:	Matvienko-Sikar, Karen; University College Cork<br>O Neill, Kate; University College Cork<br>Fraser, Abigail; University of Bristol, Population Health Sciences<br>Hayes, Catherine; Trinity College Dublin<br>Howe, Laura; University of Bristol, Population Health Sciences<br>Huizink, A. C.; VU University Amsterdam, Department of Clinical, Neuro<br>Kearney, Patricia; University College Cork<br>Khashan, Ali; University College Cork, School of Public Health; Irish Centre for Maternal and Child Health Research (INFANT) Centre, Cork University Maternity Hospital, Cork,<br>Redsell, Sarah A.; University of Nottingham<br>O'Keeffe, Linda M; University College Cork, School of Public Health; MRC Integrative Epidemiology Unit at the University of Bristol, Population Health Sciences
**Primary Subject Heading**:	Mental health
Secondary Subject Heading:	Cardiovascular medicine, Epidemiology, Obstetrics and gynaecology, Paediatrics, Public health
Keywords:	Anxiety disorders < PSYCHIATRY, Depression & mood disorders < PSYCHIATRY, Cardiac Epidemiology < CARDIOLOGY, Prenatal diagnosis < OBSTETRICS, EPIDEMIOLOGY

SCHOLARONE™
Manuscripts

I, the Submitting Author has the right to grant and does grant on behalf of all authors of the Work (as defined in the below author licence), an exclusive licence and/or a non-exclusive licence for contributions from authors who are: i) UK Crown employees; ii) where BMJ has agreed a CC-BY licence shall apply, and/or iii) in accordance with the terms applicable for US Federal Government officers or employees acting as part of their official duties; on a worldwide, perpetual, irrevocable, royalty-free basis to BMJ Publishing Group Ltd ("BMJ") its licensees and where the relevant Journal is co-owned by BMJ to the co-owners of the Journal, to publish the Work in this journal and any other BMJ products and to exploit all rights, as set out in our licence.

The Submitting Author accepts and understands that any supply made under these terms is made by BMJ to the Submitting Author unless you are acting as an employee on behalf of your employer or a postgraduate student of an affiliated institution which is paying any applicable article publishing charge ("APC") for Open Access articles. Where the Submitting Author wishes to make the Work available on an Open Access basis (and intends to pay the relevant APC), the terms of reuse of such Open Access shall be governed by a Creative Commons licence – details of these licences and which Creative Commons licence will apply to this Work are set out in our licence referred to above.

Other than as permitted in any relevant BMJ Author's Self Archiving Policies, I confirm this Work has not been accepted for publication elsewhere, is not being considered for publication elsewhere and does not duplicate material already published. I confirm all authors consent to publication of this Work and authorise the granting of this licence.

For peer review only

Maternal prenatal anxiety and depression and trajectories of cardiometabolic risk factors across childhood and adolescence: a prospective cohort study

Karen Matvienko-Sikar, School of Public Health, University College Cork, Ireland, Karen.msikar@ucc.ie
Kate N O' Neill, School of Public Health, University College Cork, Ireland, kate.oneill@ucc.ie
Abigail Fraser, Population Health Sciences, Bristol Medical School, University of Bristol, Oakfield House, Oakfield Grove, Bristol, UK, BS82BN, Abigail.fraser@bristol.ac.uk
Catherine Hayes, School of Medicine, Trinity College Dublin, Dublin, Ireland, Hayesc9@tcd.ie
Laura D Howe, Population Health Sciences, Bristol Medical School, University of Bristol, Oakfield House, Oakfield Grove, Bristol, UK, BS82BN, laura.howe@bristol.ac.uk
Anja C Huizink, Department of Clinical, Neuro- and Developmental Psychology, VU University Amsterdam, Amsterdam, The Netherlands, a.c.huizink@vu.nl
Patricia M Kearney, School of Public Health, University College Cork, Ireland, patricia.kearney@ucc.ie
Ali Khashan, Irish Centre for Maternal and Child Health Research (INFANT) Centre, Cork University Maternity Hospital, Cork, T12 YE02, Ireland; School of Public Health, University College Cork, Ireland, a.khashan@ucc.ie
Sarah Redsell, University of Nottingham, University Park, Nottingham, UK, NG7 2RD, sarah.redsell@nottingham.ac.uk
Linda M O'Keeffe[1, 2, 7], School of Public Health, University College Cork, Ireland; Population Health Sciences, Bristol Medical School, University of Bristol, Oakfield House, Oakfield Grove, Bristol, UK, BS82BN; MRC Integrative Epidemiology Unit at the University of Bristol, Oakfield House, Oakfield Grove, Bristol, UK, BS82BN, linda.okeeffe@ucc.ie

Corresponding author: Dr Karen Matvienko-Sikar, School of Public Health, 4th Floor Western Gateway Building, University College Cork, Ireland. karen.msikar@ucc.ie ,00353214205530

Word count: 3423

For peer review only

Abstract

Objectives: Quantifying long-term offspring cardiometabolic health risks associated with maternal prenatal anxiety and depression can guide cardiometabolic risk prevention. This study examines associations between maternal prenatal anxiety and depression, and offspring cardiometabolic risk from birth to 18 years.

Design. This study uses data from the Avon Longitudinal Study of Parents and Children (ALSPAC) cohort.

Participants. Participants were 526-8,606 mother-offspring pairs from the ALSPAC cohort.

Setting. British birth cohort set, Bristol, UK.

Primary and secondary outcomes. Exposures were anxiety (Crown-Crisp Inventory score) and depression (Edinburgh Postnatal Depression Scale score) measured at 18 and 32 weeks gestation. Outcomes were trajectories of offspring body mass index; fat mass; lean mass; pulse rate; glucose, diastolic and systolic blood pressure; triglycerides, high-density lipoprotein cholesterol and non-high-density lipoprotein cholesterol, and insulin from birth/early childhood to 18 years. Exposures were analysed categorically using clinically relevant, cut-offs and continuously to examine associations across the distribution of prenatal anxiety and depression.

Results. We found no strong evidence of associations between maternal anxiety and depression, and offspring trajectories of cardiometabolic risk factors. Depression at 18 weeks was associated with higher SBP at age 18 (1.62 mmHg (95% CI, 0.17, 3.07). Anxiety at 18

For peer review only - http://bmjopen.bmj.com/site/about/guidelines.xhtml

weeks was also associated with higher DBP at 7 years in unadjusted analyses (0.70 mmHg (95% CI, 0.02, 1.38); this difference persisted at age 18 years (difference at 18 years; 0.89 mmHg (95% CI, 0.05, 1.73). No associations were observed for body mass index; fat mass; lean mass; pulse rate; glucose; triglycerides, high-density lipoprotein cholesterol and non-high-density lipoprotein cholesterol, and insulin.

Conclusions. This is the first examination of maternal prenatal anxiety and depression and trajectories of offspring cardiometabolic risk. Our findings suggest that prevention of maternal prenatal anxiety and depression may have limited impact on offspring cardiometabolic health across the first two decades of life.

Keywords: Anxiety; Depression; Cardiometabolic Risk; Pregnancy: Child Trajectories

Article Summary

Strengths and limitations

- This study presents a prospective measurement of anxiety and depression twice during pregnancy, including analysis of repeated measures of 11 key cardiometabolic risk factors from birth to 18 years;
- Use of multilevel models accounting for clustering of repeated measures within individuals, and correlation between measures over time.
- Included participants tend to be predominantly white women, and were more advantaged than those excluded due to missing exposure, confounder or outcome data, limiting generalizability.
- Anxiety and depression exposures were self-reported.

For peer review only - http://bmjopen.bmj.com/site/about/guidelines.xhtml

Introduction

Maternal prenatal anxiety and depression are estimated to affect up to 25% of women[1]. Maternal prenatal anxiety and depression can result from a range of general and pregnancy-specific factors[2]. Maternal prenatal anxiety and depression are associated with adverse offspring outcomes such as low birth weight, short gestational age[3], obstetric complications[4], and poor offspring developmental and health outcomes[5,6]. Adverse offspring outcomes may result from intrauterine programming involving endocrine, inflammatory and immunological processes[6–8]. Epigenetic changes[8] and dysregulation of the maternal hypothalamic pituitary adrenal (HPA) axis are suggested to result from prenatal distress[9]; dysregulated prenatal HPA axis activity may programme foetal HPA axis activity, reactivity and later health outcomes[6,9]. It is recognised that there may be 'critical windows' of vulnerability during which exposure to prenatal anxiety and depression are particularly deleterious to offspring health[10,11]. However, chronic anxiety and/or depression (experienced over a prolonged period) during pregnancy, may also lead to adverse offspring outcomes[7]. It is further argued that such pathways, as well as behavioural and environmental mechanisms, increase risk of unfavourable offspring cardiometabolic health outcomes, including increased adiposity and poor cardiovascular function[6,12–14].

To date, inconsistent associations have been observed between maternal prenatal anxiety and depression, and child and adolescent cardiometabolic risk factors including high blood pressure[12,15], increased insulin resistance[12,14] and overweight[13,16–19]. Little is known about effects of timing of prenatal maternal anxiety and depression exposures on offspring cardiometabolic outcomes, though there is some evidence that later exposures may confer increased risk[13]. There is some limited evidence for differential associations by 'type' of

For peer review only

exposure, such as type of psychological distress exposure (e.g. stress, anxiety and depression), particularly for anthropometric outcomes[14], though this is not well understood. Studies examining associations of prenatal anxiety and depression with cardiometabolic health outcomes have predominantly examined associations with outcomes at a single time point [e.g.][12,14]. Examining early-life trajectories of cardiometabolic health outcomes provides insights into if and when associations emerge during childhood and adolescence, and whether associations persist over time. This is particularly important as cardiovascular risk originates early in life and can track to adulthood[20]. Such examinations are needed to highlight potential mechanisms, and inform the nature and timing of prevention efforts for both maternal prenatal anxiety and depression, and offspring cardiometabolic risk.

The objective of this study is to examine associations of maternal anxiety and depression with offspring cardiometabolic health outcome trajectories from birth to 18 years using data from the Avon Longitudinal Study of Parents and Children (ALSPAC).

For peer review only

Methods

Study participants

ALSPAC is a prospective birth cohort study in Southwest England[20,21]. Pregnant women resident in one of the three Bristol-based health districts with an expected delivery date between April 1, 1991 and December 31, 1992 were invited to participate. The study has been described elsewhere in detail[20,21]. ALSPAC initially enrolled a cohort of 14,451 pregnancies, from which 13,761 women provided informed consent and had 13,867 live births. Research clinics were held when the offspring were approximately 7, 9, 10, 11, 13, 15, and 18 years old. Ethical approval for the study was obtained from the ALSPAC Ethics and Law Committee and the Local Research Ethics Committees. The study website contains details of all the data that are available through a fully searchable data dictionary http://www.bristol.ac.uk/alspac/researchers/our-data/.

Study exposures

We derived and separately analysed associations between different indices of maternal prenatal anxiety and depression, and trajectories of cardiometabolic health outcomes.

Maternal self-report prenatal anxiety

Maternal prenatal anxiety was measured at 18 and 32 weeks gestation using the eight items from the anxiety subscale of Crown Crisp Experiential Index (CCEI[22]); further details in Supplementary methods S1. For our primary analysis, a score ≥85th percentile (≥85th percentile = 8) was used to define anxiety[6]. We then categorised prenatal anxiety as 'anxiety at 18 weeks', 'anxiety at 32 weeks', 'anxiety at both time points', and 'anxiety at neither time point' (reference group). In secondary analyses, anxiety was analysed as a continuous measure at 18 and 32 weeks separately and taking the mean of anxiety scores at 18 and 32 weeks to

For peer review only - http://bmjopen.bmj.com/site/about/guidelines.xhtml

explore associations with offspring cardiometabolic health across the entire distribution (to examine associations at preclinical and clinical levels).

Maternal self-report depression

Maternal prenatal depression was measured at 18 and 32 weeks gestation using the Edinburgh Postnatal Depression Scale (EPDS[23]); further details in Supplementary methods S1. For our primary analysis, a score of ≥13 was used to define clinical depression[20]. We categorised maternal prenatal depression as 'depression at 18 weeks', 'depression at 32 weeks', 'depression at both time points', and 'depression at neither time point' (reference group). The continuous measure of depression using the EPDS was also examined. In secondary analyses, depression was analysed as a continuous measure at 18 and 32 weeks separately and taking the mean of depression scores at 18 and 32 weeks to explore associations with offspring cardiometabolic health across the entire distribution (to examine associations at preclinical and clinical levels).

Study outcomes

Anthropometry

Body Mass Index (BMI: weight (kg) divided by height squared (m^2)) was calculated from 1 to 18 years using data from research clinics, routine offspring health clinics, health visitor records and parent-reported questionnaires. Whole body less head, and central fat and lean mass were derived from whole body dual energy X-ray absorptiometry (DEXA) scans assessed 5 times at ages 9, 11, 13, 15, and 18 using a Lunar prodigy narrow fan beam densitometer.

Systolic Blood Pressure (SBP), Diastolic Blood Pressure (DBP) and pulse rate

At each clinic (ages 7, 9, 10, 11, 13, 15 and 18) offspring SBP, DBP and pulse rate were measured at least twice. Measures were taken when the offspring was sitting and at rest with

For peer review only - http://bmjopen.bmj.com/site/about/guidelines.xhtml

the arm supported, using a validated device and a cuff size appropriate for upper arm circumference. The mean of the two final measures was used here.

Blood based biomarkers

Insulin was measured from cord blood at birth and from research clinic samples at age 9, 15 and 18 years. Non-fasting glucose was available at age 7; fasting glucose was available at age 9, 15 and 18 years.

Triglycerides, high-density lipoprotein cholesterol (HDL-c) and total cholesterol were measured in cord blood at birth and from venous blood subsequently. Samples were non-fasted at 7 and 9 years; fasting measures were available from clinics at 15 and 18 years. Non-HDL-c was calculated by subtracting HDL-c from total cholesterol at each measurement occasion. Trajectories of blood-based biomarkers are thus a combination of measures from cord blood, fasting bloods and non-fasting bloods, with most measures obtained through standard clinical chemistry assays.

Further details on measurement sources for outcomes are available in Supplementary methods S2.

Covariates

We considered the following as potential confounders: household social class, parity, and maternal age, education, smoking during pregnancy, pre-pregnancy BMI, all measured by mother- or mother's partner-completed questionnaires; details in Supplementary methods S3. Offspring birth weight is a potential mediator of associations of prenatal anxiety and depression with later child cardiometabolic outcomes[3,6]and was not adjusted for in our analyses.

For peer review only

For peer review only - http://bmjopen.bmj.com/site/about/guidelines.xhtml

Statistical analysis

Sex-specific patterns of change in each risk factor have been modelled previously using multilevel model[24,25] and were used here as outcome trajectories. Multilevel models estimate mean trajectories of the outcome while accounting for the non-independence (i.e. clustering) of repeated measurements within individuals, change in scale and variance of measures over time, and differences in the number and timing of measurements between individuals. Models include all available data from all eligible participants under a Missing at Random (MAR) assumption.

Trajectories of BMI were modelled using fractional polynomials[26]; all other risk factors were estimated using linear splines (two levels for all models: measurement occasion and individual). Fractional polynomial terms and linear spline periods were selected based on model fit statistics and examination of observed data. Lean mass and fat mass included three periods of change; from 9 to 13, 13 to 15, 15 to 18; SBP, DBP and pulse rate included three periods of change from 7 to 12, 12 to 16, 16 to 18; HDL-c included two periods of change from 0 to 7, 7 to 18; non-HDL-c and triglycerides included two periods of change from 0 to 9, 9 to 18; insulin included three periods of change, from 0 to 9, 9 to 15, 15 to 18; glucose included two periods of change from 7-15 and 15 to 18. Further information on the modelling of these trajectories is described in Supplementary methods S4 and Supplementary table Ss 1 to 7.

Association between maternal prenatal anxiety and depression and trajectories of offspring cardiometabolic health outcomes

Associations between maternal prenatal anxiety and depression, and trajectories of cardiometabolic risk factors were examined by including an interaction between the categories of each exposure (primary analyses) or mean scores for continuous analyses (secondary

For peer review only

analyses) and fractional polynomial age terms or linear spline periods. Based on previous modelling of outcomes, the mean outcome trajectory for each risk factor was allowed to vary by sex. We also explored whether associations between each prenatal maternal anxiety and depression exposure differed between females and males by including an interaction term between each exposure and sex. These analyses demonstrated no strong evidence of a sex interaction in the association of prenatal maternal anxiety and depression and cardiometabolic health outcome trajectories; thus all analyses were subsequently performed and presented sex-combined. We performed unadjusted and confounder adjusted analyses for all models.

Values of cardiometabolic risk factors that had a skewed distribution (BMI, fat mass, insulin and triglyceride) were (natural) log transformed prior to analysis; differences between each exposure category and the reference group from these models are calculated on the log scale. These values were then back transformed and are interpreted as the ratio of geometric means. Fat mass and lean mass were adjusted for height using the time-and sex-varying power of height that best resulted in a height-invariant measure[20]. All trajectories were modelled in MLwiN version 3.04, using the runmlwin command in Stata version 16.

Participants and measures included in analyses

Participants with measures of maternal prenatal anxiety and depression and at least one measure of a risk factor, and complete data on all confounders were included in analyses. Offspring that reported being pregnant at the 18-year clinic (n=6) were excluded from analyses at that time point only. Supplementary figure S 1 shows a flow diagram for the study.

For peer review only - http://bmjopen.bmj.com/site/about/guidelines.xhtml

Patient and public Involvement

This was a secondary analysis of data from the ALSPAC birth cohort and did not involve patient and public involvement.

For peer review only

For peer review only - http://bmjopen.bmj.com/site/about/guidelines.xhtml

Results

Supplementary table S8 shows the number of offspring with available measures of cardiometabolic risk factors at each age. The number of mother–offspring pairs included in analyses ranged from 526 participants (1,464 repeated measures) for analyses of insulin to 8,606 (80,796 repeated measures) for analyses of BMI. Prevalence of anxiety was 7.5% (n=651) at 18 weeks only, 9% (n=792) at 32 weeks only, and 12% (n=1026) at both 18 and 32 weeks. Prevalence of probable clinical depression was 5.5% (n=474) at 18 weeks only; 7.1% (n=610) at 32 weeks only; 6.3% (n=542) at both 18 and 32 weeks. Table 1 shows characteristics of participants by maternal prenatal anxiety levels (note, patterns of participant characteristics were similar for categories of prenatal maternal depression and are not shown here). Women with anxiety at any point during pregnancy (n=2469, 29%) were more likely to have lower education, and to smoke during pregnancy. Mother-offspring pairs included in analyses of BMI (n=8,606 and maximum available sample size of all analyses) tended to have higher education and lower levels of smoking during pregnancy compared to those excluded due to missing exposure, outcome or confounder data (n=5,261 to 13,341) (Supplementary table S9).

For peer review only - http://bmjopen.bmj.com/site/about/guidelines.xhtml

Table 1 Characteristics of ALSPAC participants included in analysis, by maternal anxiety levels during pregnancy

	No anxiety during pregnancy n=6,137	Anxiety at 18 weeks gestation only n=651	Anxiety at 32 weeks gestation only n=792	Anxiety at 18 & 32 weeks gestation n=1026
	**n (%)**	**n (%)**	**n (%)**	**n (%)**
**Household social class †**				
Professional	936(15.3)	74(11.4)	87(11.0)	93(9.1)
Managerial & Technical	2673(43.6)	282(43.3)	310(39.1)	408(39.8)
Non-Manual	1574(25.6)	174(26.7)	222(28.0)	284(27.7)
Manual	677(11.0)	86(13.2)	107(13.5)	167(16.3)
Part Skilled & Unskilled	277(4.5)	35(5.4)	66(8.3)	74(7.2)
**Maternal education**				
Less than O level	1410(23.0)	152(23.3)	249(31.4)	313(30.5)
O level*	2228(36.3)	257(39.5)	273(34.5)	409(39.9)
A level	1549(25.2)	167(25.7)	170(21.5)	207(20.2)
Degree or above	950(15.5)	75(11.5)	100(12.6)	97(9.5)
**Mother's Partner's highest educational qualification**				
Less than O level	1675(28.0)	189(30.0)	272(35.9)	352(36.1)
O level*	1320(22.1)	151(24.0)	176(23.2)	225(23.1)
A level	1711(28.6)	167(26.6)	196(25.9)	263(26.9)
Degree or Above	1271(21.3)	122(19.4)	114(15.0)	136(13.9)
**Maternal smoking during pregnancy**				
No	5040(82.1)	480(73.7)	587(74.1)	674(65.7)
Yes	1097(17.9)	171(26.3)	205(25.9)	352(34.3)
**Parity**				
0	2795(45.5)	319(49.0)	352(44.4)	427(41.6)
1	2286(37.2)	220(33.8)	289(36.5)	364(35.5)
2	1056(17.2)	112(17.2)	151(19.1)	235(22.9)
	**Mean (SD)**	**Mean (SD)**	**Mean (SD)**	**Mean (SD)**
Gestational age (weeks)	39.5(1.8)	39(1.7)	39(1.6)	39(1.8)
Birth weight (g)	3431(535)	3395(527)	3417(500)	3423(546)
Pre-pregnancy BMI (kg/m^2)	22.9(3.7)	23(4.2)	23(3.8)	23(4.0)
Maternal age (years)	29.0(4.5)	28(4.9)	28(5.0)	28(4.9)

Number of participants available for analyses of BMI (n=8,606) used as the denominator in this table given the varying sample sizes included in analyses. † Household social class was measured as the highest of the mother's or her partner's occupational social class using data on job title and details of occupation collected about the mother and her partner from the mother's questionnaire at 32 weeks gestation. Social class was derived using the standard occupational classification (SOC) codes developed by the United Kingdom Office of Population Census and Surveys and classified as I professional, II managerial and technical, IIINM non-manual, IIIM manual, and IV&V part skilled occupations and unskilled occupations

* O levels equate to current GCSEs demonstrating education level at aged 16.

For peer review only

Anxiety during pregnancy

In confounder-adjusted analyses, associations were not observed between anxiety at 18 and 32 weeks, and BMI and fat mass (Figure 1, Supplementary table S10 and S11). For instance, the difference in fat mass at 18 years between offspring of women who experienced anxiety at 18 and 32 weeks and those who did not was 4.77% (95% CI, -0.51, 10.05). Anxiety at 18 weeks only was also associated with higher DBP at 7 years in unadjusted analyses (0.70 mmHg (95% CI, 0.02, 1.38); this difference persisted at age 18 years (difference at 18 years; 0.89 mmHg (95% CI, 0.05, 1.73). (Figure 2 and Supplementary table S12).

In confounder-adjusted analyses, we found no strong evidence that prenatal anxiety was associated with trajectories of lean mass from 9 to 18 years (Figure 1 and Supplementary table S13), glucose from 7 to 18 years (Figure 3 and Supplementary table S 14) and insulin (Figure 3 and Supplementary table S15), triglyceride (Figure 4 and Supplementary table S15), HDL-c and non-HDL-c (Figure 4 and Supplementary table S16), all from birth to 18 years.

Continuous analyses

Results for all analyses were similar when prenatal maternal anxiety was examined as a continuous exposure at each time point separately or when the mean of the two measures was examined (see Supplementary figure S2- 12).

Depression during pregnancy

Associations were not observed between depression at both 18 and 32 weeks gestation, and BMI and fat mass (Supplementary figure S13, Supplementary table S17 and S18). For instance,

For peer review only

the difference in fat mass at 18 years for offspring of women who experienced depression at 18 and 32 weeks was 2.32% (95% CI, -4.58, 9.22). Depression at 18 weeks only was associated with higher SBP at age 18 (1.62 mmHg (95% CI, 0.17, 3.07). (Supplementary figure S14 and Supplementary table S19). We found no strong evidence that depression during pregnancy was associated with trajectories of DBP and pulse rate from 7 to 18 (Supplementary figure S14 and Supplementary table S19), lean mass from 9 to 18 years (Supplementary figure S13 and Supplementary table S20), glucose from 7 to 18 years (Supplementary figure S15 and Supplementary table S21), insulin (Supplementary figure S15 and Supplementary table S22), triglyceride (Supplementary figure S16 and Supplementary table S22), HDL-c and non-HDL-c (Supplementary figure S16 and Supplementary table S23), all from birth to 18 years.

Continuous analyses

Results for all analyses were similar when prenatal maternal depression was examined as a continuous exposure at each time point separately or when the mean of the two measures was examined (see Supplementary figures S17-27).

[Figures 1 to 4]

For peer review only

Discussion

In this large, contemporary prospective birth cohort study with repeated assessment of exposures and outcomes, we found no strong evidence that maternal anxiety and depression during pregnancy were associated with offspring cardiometabolic risk factors from birth to 18 years, regardless of whether exposure occurred at 18 weeks or 32 weeks gestation or both.

Confounder adjusted analyses did not indicate associations between prenatal maternal anxiety and depression and offspring anthropometric outcomes. Our findings differ from previous research suggesting maternal prenatal anxiety and depression adversely impact offspring health and developmental outcomes[12,13,27] . A recent examination of the impact of mid-pregnancy maternal prenatal depression and anxiety identified associations with higher triglycerides in females and higher pulse rate in males at 10 years[14]. However, similar to our study, this study did not find associations between maternal prenatal depression and anxiety and offspring blood pressure, glucose, insulin, or serum lipids[14]. Similarly, a number of prospective cohort studies have reported a lack of evidence to support associations between anxiety and depression and blood pressure[15], adiposity[17–19] and glucose and insulin resistance[12]; such studies examined cardiometabolic risk factors at static time points only however.

Attenuation of associations in adjusted analyses highlights that factors such as household social class, smoking during pregnancy, and BMI influence child cardiometabolic trajectories over time. This in line with consistent evidence linking sociodemographic factors, and maternal behaviours and weight status to child cardiometabolic risk [28–30]. As such, the impact of these factors on child health outcomes likely exceeds any impact of prenatal anxiety and depression in this cohort. While such factors attenuated some observed associations and

For peer review only - http://bmjopen.bmj.com/site/about/guidelines.xhtml

indicate potential areas for future research, we do not believe this attenuation suggests undetected mediators of foetal programming in this study. This is because factors, such as maternal BMI in pregnancy, do not represent mediators which sit along the causal pathway between exposure and outcome, but are instead confounders that represent common causes of both maternal mental health and offspring cardiovascular risk and that typically arise prior to both exposure and outcome. Lack of observed associations in the current study may also arise because in-utero maternal anxiety and depression exposure may be too early to impact on offspring cardiometabolic trajectories over the long term[13]. A recent review of associations between maternal stress and child weight outcomes found that later childhood exposure was more strongly associated with offspring weight than exposure in infancy[31]. Thus we may not have observed associations here because maternal prenatal anxiety and depression confers little impact on offspring cardiometabolic health across the early life course, irrespective of gestational timing of exposure. However, given our identification of some associations with offspring fat mass in this study, future work is needed to further examine effects of maternal prenatal anxiety and depression on offspring cardiometabolic health in the first two decades of life. The impact of postnatal anxiety and depression on child cardiometabolic trajectories in the ALSPAC cohort is currently being examined by the authors to further examine the effect of later exposure. Future research examining the impacts of on-going maternal mood disturbance across the perinatal period also warrants further examination.

Strengths and Limitations

Strengths of this study include: prospective measurement of anxiety and depression twice during pregnancy; analysis of repeated measures of 11 key cardiometabolic risk factors from birth to 18 years; use of multilevel models accounting for clustering of repeated measures within individuals, and correlation between measures over time. Examination of anxiety and

For peer review only - http://bmjopen.bmj.com/site/about/guidelines.xhtml

depression as both categorical and continuous exposures is a further strength, which enabled examination of exposures in terms of clinical cut-offs, and incremental increases in exposure levels across the entire distribution of anxiety and depression; this is important because exposure to subclinical levels my still result in adverse outcomes that would be missed if data were examined categorically only. Limitations include generalisability of findings, as included participants tend to be more advantaged than those excluded due to missing exposure, confounder or outcome data. The range in participant numbers between analyses due to participant follow-up may represent selection bias and further impact generalisability of findings. In addition, ALSPAC includes a high proportion (~98%) white women, limiting representativeness of the findings for non-White ethnicities and ability to perform subgroup analyses for different ethnicity groups. Similarly, ALSPAC included live births only; women experiencing acute and/or chronic distress may have experienced spontaneous abortion, leading to live birth bias[32]. Self-reporting of prenatal anxiety and depression is a further limitation because self-reports do not tend to correlate well with psychophysiological indicators that could program risk[33]. A further measurement limitation was the use of a generalized anxiety measure in the cohort rather than inclusion of pregnancy-specific anxiety measure. Pregnancy-specific anxiety is distinct from general anxiety[34] and is a robust risk factor for child health outcomes, beyond the impact of general anxiety and depression[29,35]. As such, future research should include a pregnancy-specific measure to determine potential differential effects on child cardiometabolic outcomes. In addition, the role of additional factors, such as prenatal antidepressant use could not be examined in the current study due to the low proportion of women reporting antidepressant use in the cohort; future research should consider the role of factors such as antidepressant use in potential associations between maternal mental health and child cardiometabolic health outcomes.

For peer review only - http://bmjopen.bmj.com/site/about/guidelines.xhtml

Conclusion

Our findings suggest that maternal prenatal anxiety and depression do not impact offspring cardiometabolic health outcomes in the first two decades of life; these findings may provide reassurance to women experiencing prenatal anxiety and/or depression that any impacts on offspring cardiometabolic health from birth to the end of adolescence are likely to be small. Approaches and strategies to prevent and/or reduce maternal prenatal anxiety and depression may have limited impact on offspring cardiometabolic health in the first two decades of life.

For peer review only

Figure Legends

Figure 1 Legend: Trajectories are adjusted for maternal age at birth of offspring, parental household socioeconomic position, parity, maternal pre-pregnancy BMI and maternal smoking during pregnancy. Anxiety at 18 and 32 weeks gestation is defined as ≥85th percentile of the anxiety subscale of the Crown-Crisp Index CCEI) for the whole cohort at each time point separately (≥85th percentile = 8 at both 18 and 32 weeks gestation).

Figure 2 Legend: DBP, diastolic blood pressure; SBP, systolic blood pressure.

Trajectories are adjusted for maternal age at birth of offspring, parental household socioeconomic position, parity, maternal pre-pregnancy BMI and maternal smoking during pregnancy. Anxiety at 18 and 32 weeks gestation is defined as ≥85th percentile of the anxiety subscale of the Crown-Crisp Index CCEI) for the whole cohort at each time point separately (≥85th percentile = 8 at both 18 and 32 weeks gestation).

Figure 3 Legend: HDL-c, high-density lipoprotein-cholesterol; non-HDL-c.

Trajectories are adjusted for maternal age at birth of offspring, parental household socioeconomic position, parity, maternal pre-pregnancy BMI and maternal smoking during pregnancy. Anxiety at 18 and 32 weeks gestation is defined as ≥85th percentile of the anxiety subscale of the Crown-Crisp Index CCEI) for the whole cohort at each time point separately (≥85th percentile = 8 at both 18 and 32 weeks gestation).

Figure 4 Legend: Trajectories are adjusted for maternal age at birth of offspring, parental household socioeconomic position, parity, maternal pre-pregnancy BMI and maternal smoking during pregnancy. Anxiety at 18 and 32 weeks gestation is defined as ≥85th

For peer review only - http://bmjopen.bmj.com/site/about/guidelines.xhtml

percentile of the anxiety subscale of the Crown-Crisp Index CCEI) for the whole cohort at each time point separately (≥85th percentile = 8 at both 18 and 32 weeks gestation).

Funding Statement

The UK Medical Research Council and Wellcome (Grant ref: 217065/Z/19/Z) and the University of Bristol provide core support for ALSPAC. This publication is the work of the authors and KMS and LMOK will serve as guarantors for the contents of this paper. KMS is supported by a Health Research Board of Ireland (HRB) Applying Research to Policy and Practice award (HRB-ARPP- A011). LMOK and KON are supported by a HRB Emerging Investigator Award (EIA-2019-007). KON is also supported by a HRB Research Leader Award (HRB RL/2013/7) and a HRB Interdisciplinary Capacity Enhancement Award (HRB ICE 2015-1026). LDH and AF are supported by Career Development Awards from the UK Medical Research Council (grants MR/M020894/1 and MR/M009351/1, respectively). LDH and AF work in a unit that receives funds from the UK Medical Research Council (grant MC_UU_00011/3, MC_UU_00011/6). The research funders had no role in the study design; data collection, analysis, and interpretation of data; writing of the manuscript; or the decision to submit the manuscript for publication.

Competing interests statement

None of the authors have any conflicts of interest to declare.

Data availability

Data used in this study was from the Avon Longitudinal Study of Parents and Children (ALSPAC), requests for access to this data can be made to the ALSPAC executive committee.

Author contributions

KMS conceptualised the study, conducted analyses, drafted and revised the manuscript. KON conducted analyses, and critically reviewed and revised the manuscript. AF critically reviewed and revised the manuscript. CH contributed to interpretation of findings and critically reviewed and revised the manuscript. LDH critically reviewed and revised the manuscript. ACH contributed to interpretation of findings and critically reviewed and revised the manuscript. PMK contributed to interpretation of findings and critically reviewed and revised the manuscript. AK contributed to interpretation of findings and critically reviewed and revised the manuscript. SR contributed to interpretation of findings and critically reviewed and revised the manuscript. LOK conceptualised the study, conducted analyses, drafted and revised the manuscript. All authors approved the final manuscript.

Acknowledgements

We are extremely grateful to all the families who took part in this study, the midwives for their help in recruiting them, and the whole ALSPAC team, which includes interviewers, computer and laboratory technicians, clerical workers, research scientists, volunteers, managers, receptionists and nurses. We are also grateful to Dr Darren Dahly in the Health Research Board Clinical Research Facility Cork for feedback on an earlier draft of this manuscript.

For peer review only - http://bmjopen.bmj.com/site/about/guidelines.xhtml

Ethics Statement

This study used secondary data from the ALSPAC cohort. Participants in the original cohort study provided informed consent to participate and for their data to be used as secondary data in subsequent research.

References

1. Pearson RM, Carnegie RE, Cree C, Rollings C, Rena-Jones L, Evans J, et al. Prevalence of Prenatal Depression Symptoms Among 2 Generations of Pregnant Mothers: The Avon Longitudinal Study of Parents and Children. JAMA network open. United States: American Medical Association; 2018;1:e180725.

2. Dunkel Schetter C. Psychological Science on Pregnancy: Stress Processes, Biopsychosocial Models, and Emerging Research Issues. Annu Rev Psychol. 2011;62:531–58.

3. Grote NK, Bridge JA, Gavin AR, Melville JL, Iyengar S, Katon WJ. A Meta-analysis of Depression During Pregnancy and the Risk of Preterm Birth, Low Birth Weight, and Intrauterine Growth Restriction. Arch Gen Psychiatry. 2010;67:1012.

4. Alder J, Fink N, Bitzer J, Hösli I, Holzgreve W. Depression and anxiety during pregnancy: A risk factor for obstetric, fetal and neonatal outcome? A critical review of the literature. The Journal of Maternal-Fetal & Neonatal Medicine. 2007;20:189–209.

5. O'Connor TG, Heron J, Golding J, Beveridge M, Glover V. Maternal antenatal anxiety and children's behavioural/emotional problems at 4 years. Br J Psychiatry. 2002;180:502–8.

6. Entringer S. Impact of stress and stress physiology during pregnancy on child metabolic function and obesity risk: Current Opinion in Clinical Nutrition and Metabolic Care. 2013;16:320–7.

7. Wadhwa P, Buss C, Entringer S, Swanson J. Developmental Origins of Health and Disease: Brief History of the Approach and Current Focus on Epigenetic Mechanisms. Semin Reprod Med. 2009;27:358–68.

8. Mulligan C, D'Errico N, Stees J, Hughes D. Methylation changes at *NR3C1* in newborns associate with maternal prenatal stress exposure and newborn birth weight. Epigenetics. 2012;7:853–7.

For peer review only

9. Entringer S, Buss C, Rasmussen JM, Lindsay K, Gillen DL, Cooper DM, et al. Maternal cortisol during pregnancy and infant adiposity: a prospective investigation. The Journal of Clinical Endocrinology & Metabolism. 2016;jc.2016-3025.

10. Van den Bergh BRH, Mulder EJH, Mennes M, Glover V. Antenatal maternal anxiety and stress and the neurobehavioural development of the fetus and child: links and possible mechanisms. A review. Neuroscience & Biobehavioral Reviews. 2005;29:237–58.

11. Glover V. Annual Research Review: Prenatal stress and the origins of psychopathology: an evolutionary perspective: Prenatal stress and the origins of psychopathology. Journal of Child Psychology and Psychiatry. 2011;52:356–67.

12. van Dijk AE, Dawe K, Deanfield J, Stronks K, Gemke RJBJ, Vrijkotte TGM, et al. The association of maternal prenatal psychosocial stress with vascular function in the child at age 10-11 years: findings from the Avon longitudinal study of parents and children. European journal of preventive cardiology. England: Sage; 2014;21:1097–108.

13. Vehmeijer FOL, C V Silva C, Derks IPM, El Marroun H, Oei EHG, Felix JF, et al. Associations of Maternal Psychological Distress during Pregnancy with Childhood General and Organ Fat Measures. Childhood obesity (Print). United States: Mary Ann Liebert; 2019;15:313–22.

14. Silva CCV, Vehmeijer FOL, El Marroun H, Felix JF, Jaddoe VWV, Santos S. Maternal psychological distress during pregnancy and childhood cardio-metabolic risk factors. Nutrition, metabolism, and cardiovascular diseases : NMCD. Netherlands: Elsevier; 2019;29:572–9.

15. van Dijk AE, van Eijsden M, Stronks K, Gemke RJBJ, Vrijkotte TGM. The association between prenatal psychosocial stress and blood pressure in the child at age 5-7 years. PloS one. United States: Public Library of Science; 2012;7:e43548.

16. Matvienko-Sikar K, Cooney J, Flannery C, Murphy J, Khashan A, Huizink A. Maternal stress in the first 1000 days and risk of childhood obesity: a systematic review. Journal of Reproductive and Infant Psychology. 2020;1–25.

17. Ertel KA, Huang T, Rifas-Shiman SL, Kleinman K, Rich-Edwards J, Oken E, et al. Perinatal weight and risk of prenatal and postpartum depressive symptoms. Annals of epidemiology. United States: Elsevier; 2017;27:695.

18. Braungart-Rieker JM, Lefever JB, Planalp EM, Moore ES. Body Mass Index at 3 Years of Age: Cascading Effects of Prenatal Maternal Depression and Mother-Infant Dynamics. The Journal of pediatrics. United States: Mosby; 2016;177:128.

19. Ingstrup KG, Schou Andersen C, Ajslev TA, Pedersen P, Sørensen TIA, Nohr EA. Maternal Distress during Pregnancy and Offspring Childhood Overweight. Journal of obesity. United States: Hindawi Publishing Corporation; 2012;2012:462845.

20. Fraser, A. O'Keeffe,L.M., Howe, L. Accounting for height in indices of body composition during childhood and adolescence. Wellcome open research. 4.

For peer review only - http://bmjopen.bmj.com/site/about/guidelines.xhtml

21. Boyd A, Golding J, Macleod J, Lawlor DA, Fraser A, Henderson J, et al. Cohort Profile: The 'Children of the 90s'—the index offspring of the Avon Longitudinal Study of Parents and Children. International Journal of Epidemiology. 2013;42:111–27.

22. Birtchnell J, Evans C, Kennard J. The total score of the Crown-Crisp Experiential Index: A useful and valid measure of psychoneurotic pathology. British Journal of Medical Psychology. 1988;61:255–66.

23. Cox JL, Holden JM, Sagovsky R. Detection of Postnatal Depression: Development of the 10-item Edinburgh Postnatal Depression Scale. Br J Psychiatry. 1987;150:782–6.

24. O'Keeffe LM, Simpkin AJ, Tilling K, Anderson EL, Hughes AD, Lawlor DA, et al. Sex-specific trajectories of measures of cardiovascular health during childhood and adolescence: A prospective cohort study. Atherosclerosis. 2018;278:190–6.

25. O'Keeffe LM, Simpkin AJ, Tilling K, Anderson EL, Hughes AD, Lawlor DA, et al. Data on trajectories of measures of cardiovascular health in the Avon Longitudinal Study of Parents and Children (ALSPAC). Data in Brief. 2019;23:103687.

26. Royston P, Altman DG. Regression Using Fractional Polynomials of Continuous Covariates: Parsimonious Parametric Modelling. Applied Statistics. 1994;43:429.

27. Fan F, Zou Y, Tian H, Zhang Y, Zhang J, Ma X, et al. Effects of maternal anxiety and depression during pregnancy in Chinese women on children's heart rate and blood pressure response to stress. Journal of human hypertension. England: Nature Publishing Group; 2016;30:171–6.

28. Boney CM. Metabolic Syndrome in Childhood: Association With Birth Weight, Maternal Obesity, and Gestational Diabetes Mellitus. PEDIATRICS. 2005;115:e290–6.

29. Lobel M, Cannella DL, Graham JE, DeVincent C, Schneider J, Meyer BA. Pregnancy-specific stress, prenatal health behaviors, and birth outcomes. Health Psychology. 2008;27:604–15.

30. Slopen N, Goodman E, Koenen KC, Kubzansky LD. Socioeconomic and Other Social Stressors and Biomarkers of Cardiometabolic Risk in Youth: A Systematic Review of Less Studied Risk Factors. Janda M, editor. PLoS ONE. 2013;8:e64418.

31. Tate EB, Wood W, Liao Y, Dunton GF. Do stressed mothers have heavier children? A meta-analysis on the relationship between maternal stress and child body mass index: Maternal stress and child obesity. Obes Rev. 2015;16:351–61.

32. Liew Z, Olsen J, Cui X, Ritz B, Arah OA. Bias from conditioning on live birth in pregnancy cohorts: an illustration based on neurodevelopment in children after prenatal exposure to organic pollutants. International Journal of Epidemiology. 2015;44:345–54.

33. Himes K, Simhan H. Plasma Corticotropin-Releasing Hormone and Cortisol Concentrations and Perceived Stress among Pregnant Women with Preterm and Term Birth. Amer J Perinatol. 2011;28:443–8.

34. Huizink AC, Mulder EJH, Robles de Medina PG, Visser GHA, Buitelaar JK. Is pregnancy anxiety a distinctive syndrome? Early Human Development. 2004;79:81–91.

For peer review only - http://bmjopen.bmj.com/site/about/guidelines.xhtml

35. Szekely E, Neumann A, Sallis H, Jolicoeur-Martineau A, Verhulst FC, Meaney MJ, et al. Maternal Prenatal Mood, Pregnancy-Specific Worries, and Early Child Psychopathology: Findings From the DREAM BIG Consortium. Journal of the American Academy of Child & Adolescent Psychiatry. 2021;60:186–97.

For peer review only

For peer review only - http://bmjopen.bmj.com/site/about/guidelines.xhtml

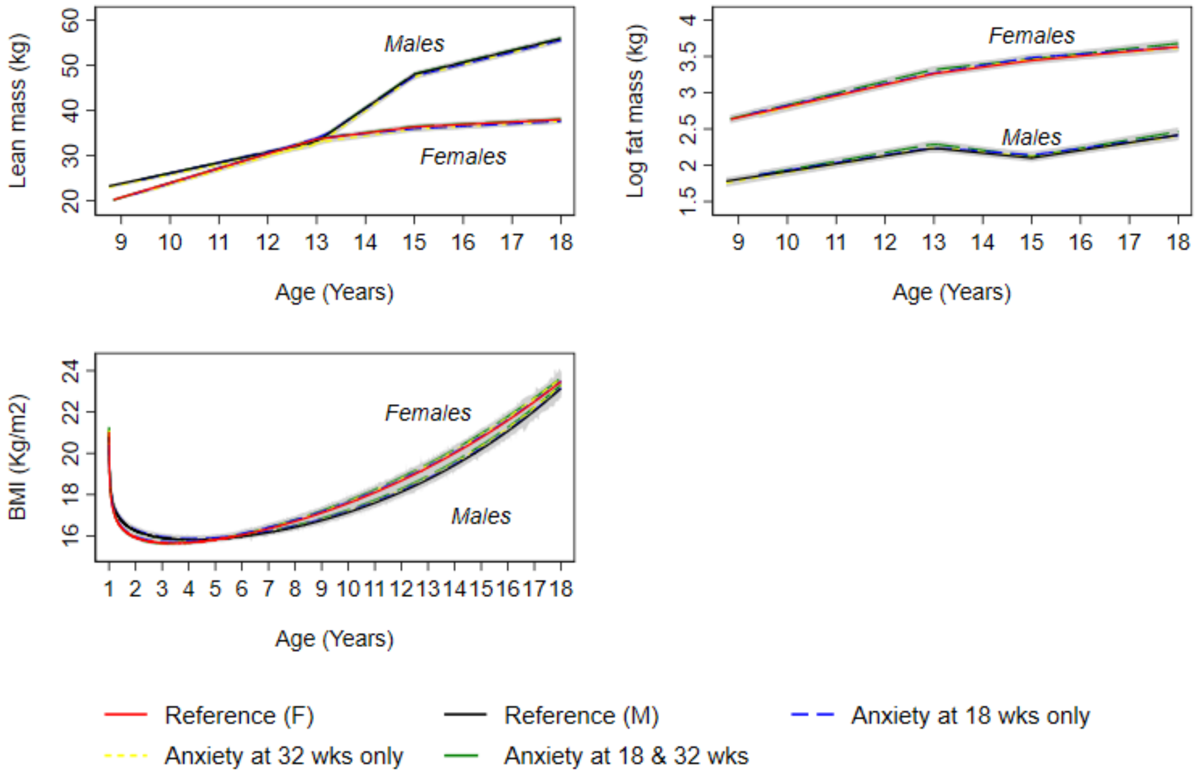

Figure 1 Mean predicted confounder adjusted trajectories of lean mass (9 to 18 years), log fat mass (9 to 18 years) and log BMI (1 to 18 years), by maternal anxiety levels during pregnancy

Legend: Trajectories are adjusted for maternal age at birth of offspring, parental household socioeconomic position, parity, maternal pre-pregnancy BMI and maternal smoking during pregnancy. Anxiety at 18 and 32 weeks gestation is defined as ≥85th percentile of the anxiety subscale of the Crown-Crisp Index CCEI) for the whole cohort at each time point separately (≥85th percentile = 8 at both 18 and 32 weeks gestation).

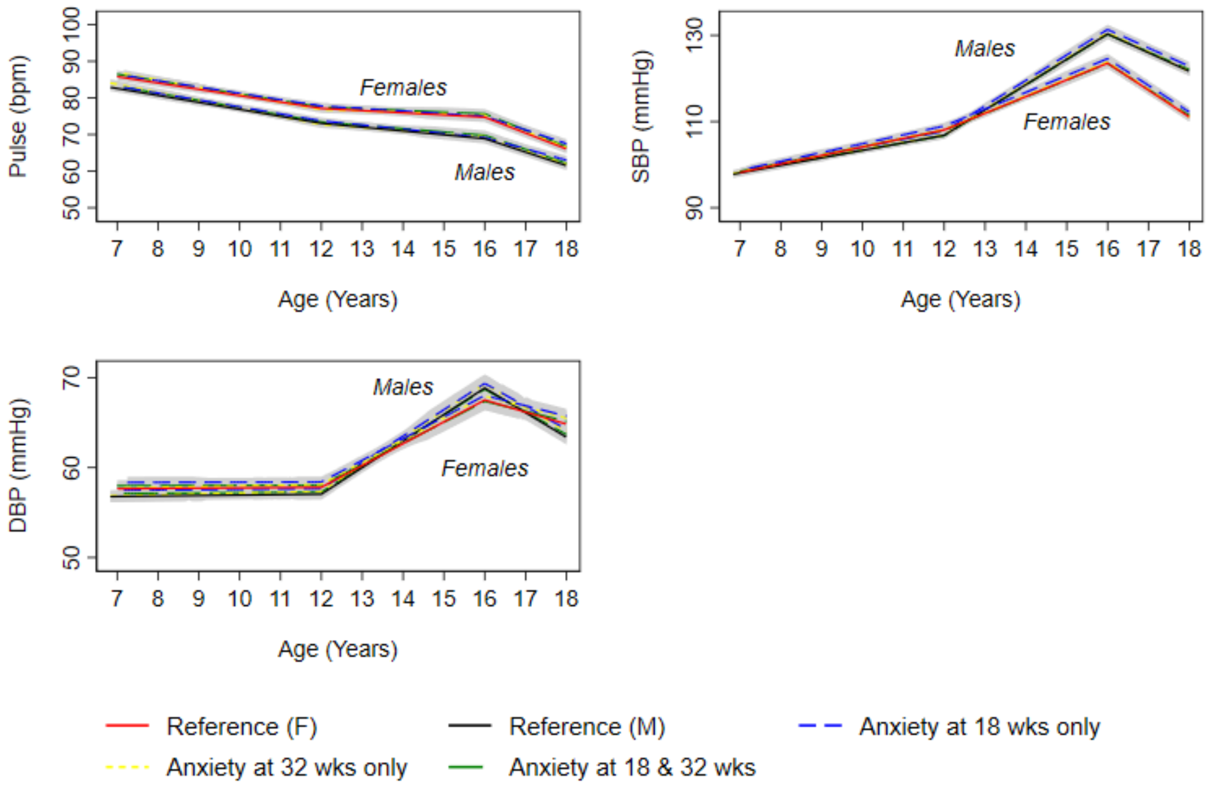

Figure 2 Mean predicted confounder adjusted trajectories of pulse rate, SBP and DBP from 7 to 18 years, by maternal anxiety levels during pregnancy

Legend: DBP, diastolic blood pressure; SBP, systolic blood pressure.
Trajectories are adjusted for maternal age at birth of offspring, parental household socioeconomic position, parity, maternal pre-pregnancy BMI and maternal smoking during pregnancy. Anxiety at 18 and 32 weeks gestation is defined as ≥85th percentile of the anxiety subscale of the Crown-Crisp Index CCEI) for the whole cohort at each time point separately (≥85th percentile = 8 at both 18 and 32 weeks gestation).

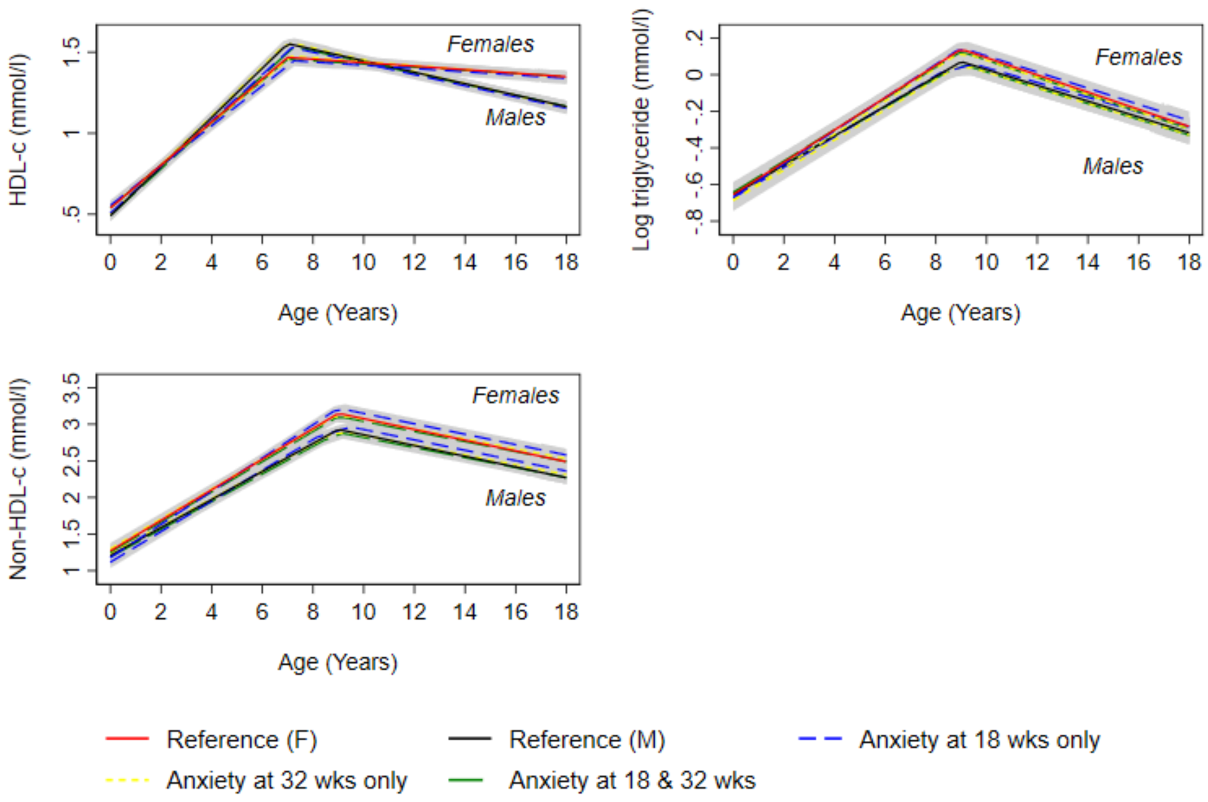

Figure 3 Mean predicted confounder adjusted trajectories of HDL-c, log triglyceride and non-HDL-c from birth to 18 years, by maternal anxiety levels during pregnancy

Legend: HDL-c, high-density lipoprotein-cholesterol; non-HDL-c.
Trajectories are adjusted for maternal age at birth of offspring, parental household socioeconomic position, parity, maternal pre-pregnancy BMI and maternal smoking during pregnancy. Anxiety at 18 and 32 weeks gestation is defined as ≥85th percentile of the anxiety subscale of the Crown-Crisp Index CCEI) for the whole cohort at each time point separately (≥85th percentile = 8 at both 18 and 32 weeks gestation).

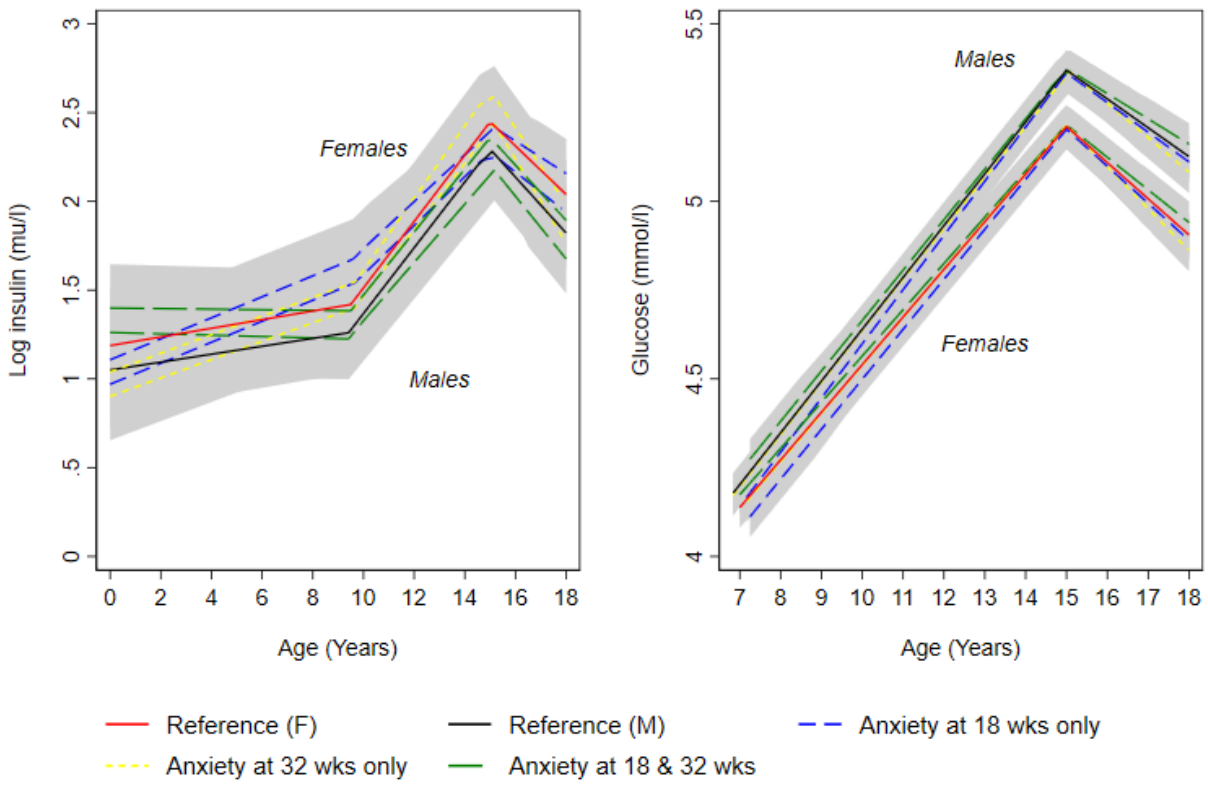

Figure 4 Mean predicted confounder adjusted trajectories of log insulin (birth to 18 years) and glucose (7 to 18 years), by maternal anxiety levels during pregnancy

Legend: Trajectories are adjusted for maternal age at birth of offspring, parental household socioeconomic position, parity, maternal pre-pregnancy BMI and maternal smoking during pregnancy. Anxiety at 18 and 32 weeks gestation is defined as ≥85th percentile of the anxiety subscale of the Crown-Crisp Index CCEI) for the whole cohort at each time point separately (≥85th percentile = 8 at both 18 and 32 weeks gestation).

review only

Supplemental Material

Maternal prenatal anxiety and depression and trajectories of cardiometabolic risk factors across childhood and adolescence: a prospective cohort study

Karen Matvienko-Sikar[1], Kate N O' Neill[1], Abigail Fraser[2], Catherine Hayes[3], Laura D Howe[2], Anja C Huizink[4], Patricia M Kearney[1], Ali Khashan[1,5], Sarah Redsell[6], Linda M O'Keeffe[1, 2, 7]

1. School of Public Health, University College Cork, Ireland

2. Population Health Sciences, Bristol Medical School, Oakfield House, Oakfield Grove, Bristol, UK, BS82BN

3. School of Medicine, Trinity College Dublin, Dublin, Ireland

4. Department of Clinical, Neuro- and Developmental Psychology, VU University Amsterdam, Amsterdam, The Netherlands

5. Irish Centre for Maternal and Child Research (INFANT) Centre, Cork University Maternity Hospital, Cork, T12 YE02, Ireland

6. University of Nottingham, University Park, Nottingham, UK, NG7 2RD

7. MRC Integrative Epidemiology Unit at the University of Bristol, Oakfield House, Oakfield Grove, Bristol, UK, BS82BN

Corresponding author: Dr Karen Matvienko-Sikar, School of Public Health, 4th Floor Western Gateway Building, University College Cork, Ireland. karen.msikar@ucc.ie

For peer review only

List of contents

Supplemental Methods
Supplemental Methods S1 Details of study exposure measurement and prevalence
Supplemental Methods S2 Details of measurement sources for cardiometabolic risk factors
Supplemental Methods S3 Details on measurement of confounders
Supplemental Methods S4 Details of model selection for outcomes

Supplemental Tables
Supplemental Table S1 Model details for log BMI trajectories
Supplemental Table S2 Model details for log fat mass trajectories
Supplemental Table S3 Model details for lean mass trajectories
Supplemental Table S4 Model details for SBP, DBP and pulse rate trajectories
Supplemental Table S5 Model details for glucose trajectories
Supplemental Table S6 Model details for log insulin and triglyceride trajectories
Supplemental Table S7 Model details for HDL-c and non-HDL-c trajectories
Supplemental Table S8 Number of participants with cardiometabolic measures at each time point
Supplemental Table S9 Characteristics at birth of the mothers of children included in models compared with those excluded due to missing exposure, outcome or confounder data
Supplemental Table S10 Mean trajectories of BMI estimated from multilevel models, by maternal anxiety during pregnancy
Supplemental Table S11 Mean trajectories of fat mass estimated from multilevel models, by maternal anxiety during pregnancy
Supplemental Table S12 Mean trajectories of blood pressure and pulse rate estimated from multilevel models, by maternal anxiety during pregnancy
Supplemental Table S13 Mean trajectories of lean mass estimated from multilevel models, by maternal anxiety during pregnancy
Supplemental Table S14 Mean trajectories of glucose estimated from multilevel models, by maternal anxiety during pregnancy
Supplemental Table S15 Mean trajectories of log insulin and triglyceride estimated from multilevel models, by maternal anxiety during pregnancy
Supplemental Table S16 Mean trajectories of HDL-c and non-HDL-c estimated from multilevel models, by maternal anxiety during pregnancy
Supplemental Table S17 Mean trajectories of BMI estimated from multilevel models, by maternal depression during pregnancy
Supplemental Table S18 Mean trajectories of fat mass estimated from multilevel models, by maternal depression during pregnancy
Supplemental Table S19 Mean trajectories of blood pressure and pulse rate estimated from multilevel models, by maternal depression during pregnancy
Supplemental Table S20 Mean trajectories of lean mass estimated from multilevel models, by maternal depression during pregnancy
Supplemental Table S21 Mean trajectories of glucose estimated from multilevel models, by maternal depression during pregnancy
Supplemental Table S22 Mean trajectories of log insulin and triglyceride estimated from multilevel models, by maternal depression during pregnancy
Supplemental Table S23 Mean trajectories of HDL-c and non-HDL-estimated from multilevel models, by maternal depression during pregnancy

Supplemental Figures
Supplemental Figure S1 Flow diagram of study design
Supplemental Figure S2 Mean predicted confounder adjusted trajectories of lean mass (9 to 18 years), by continuous maternal anxiety levels during pregnancy
Supplemental Figure S3 Mean predicted confounder adjusted trajectories of log fat mass (9 to 18 years), by continuous maternal anxiety levels during pregnancy
Supplemental Figure S4 Mean predicted confounder adjusted trajectories of log BMI (1 to 18 years), by continuous maternal anxiety levels during pregnancy
Supplemental Figure S5 Mean predicted confounder adjusted trajectories of pulse rate from 7 to 18 years by continuous maternal anxiety levels during pregnancy
Supplemental Figure S6 Mean predicted confounder adjusted trajectories of SBP from 7 to 18 years by continuous maternal anxiety levels during pregnancy

For peer review only - http://bmjopen.bmj.com/site/about/guidelines.xhtml

Supplemental Figure S7 Mean predicted confounder adjusted trajectories of DBP from 7 to 18 years by continuous maternal anxiety levels during pregnancy
Supplemental Figure S8 Mean predicted confounder adjusted trajectories of log insulin from (birth to 18 years) by continuous maternal anxiety levels during pregnancy
Supplemental Figure S9 Mean predicted confounder adjusted trajectories of glucose (7 to18 years), by continuous maternal anxiety levels during pregnancy
Supplemental Figure S10 Mean predicted confounder adjusted trajectories of HDL-c (birth to18 years), by continuous maternal anxiety levels during pregnancy
Supplemental Figure S11 Mean predicted confounder adjusted trajectories of triglyceride (birth to18 years), by continuous maternal anxiety levels during pregnancy
Supplemental Figure S12 Mean predicted confounder adjusted trajectories of non-HDL-c (birth to18 years), by continuous maternal anxiety levels during pregnancy
Supplemental Figure S13 Mean predicted confounder adjusted trajectories of lean mass (9 to 18 years), log fat mass (9 to 18 years) and log BMI (1 to 18 years), by categorical maternal depression levels during pregnancy
Supplemental Figure S14 Mean predicted confounder adjusted trajectories of pulse rate, SBP and DBP from 7 to 18 years, by categorical maternal depression levels during pregnancy
Supplemental Figure S15 Mean predicted confounder adjusted trajectories of log insulin (birth to 18 years) and glucose (7 to18 years), by categorical maternal depression levels during pregnancy
Supplemental Figure S16 Mean predicted confounder adjusted trajectories of HDL-c, triglyceride and non-HDL-c from birth to 18 years, by categorical maternal depression levels during pregnancy
Supplemental Figure S17 Mean predicted confounder adjusted trajectories of lean mass (9 to 18 years), by continuous maternal depression levels during pregnancy
Supplemental Figure S18 Mean predicted confounder adjusted trajectories of log fat mass (9 to 18 years), by continuous maternal depression levels during pregnancy
Supplemental Figure S19 Mean predicted confounder adjusted trajectories of log BMI (1 to 18 years), by continuous maternal depression levels during pregnancy
Supplemental Figure S20 Mean predicted confounder adjusted trajectories of pulse rate from 7 to 18 years by continuous maternal depression levels during pregnancy
Supplemental Figure S21 Mean predicted confounder adjusted trajectories of SBP from 7 to 18 years by continuous maternal depression levels during pregnancy
Supplemental Figure S22 Mean predicted confounder adjusted trajectories of DBP from 7 to 18 years by continuous maternal depression levels during pregnancy
Supplemental Figure S23 Mean predicted confounder adjusted trajectories of log insulin from (birth to 18 years) by continuous maternal depression levels during pregnancy
Supplemental Figure S24 Mean predicted confounder adjusted trajectories of glucose (7 to18 years), by continuous maternal depression levels during pregnancy
Supplemental Figure S25 Mean predicted confounder adjusted trajectories of HDL-c (birth to18 years), by continuous maternal depression levels during pregnancy
Supplemental Figure S26 Mean predicted confounder adjusted trajectories of triglyceride (birth to18 years), by continuous maternal depression levels during pregnancy
Supplemental Figure S27 Mean predicted confounder adjusted trajectories of non-HDL-c (birth to18 years), by continuous maternal depression levels during pregnancy

For peer review only

Supplemental Methods S1 Details of study exposure measurement and prevalence

Maternal self-report prenatal anxiety

Maternal prenatal anxiety was measured using the eight items from the anxiety subscale of Crown Crisp Experiential Index (CCEI). The CCEI is a validated self-report measure, with anxiety items rated on a 4-point scale ("very often" to "never"); a higher score is indicative of higher anxiety. Items ask women how they feel 'at this stage in pregnancy' and include "Do you sometimes feel panicky". This scale has previously demonstrated internal consistency coefficients above 0.80 in the ALSPAC cohort during pregnancy[8].

Maternal self-report depression

Prenatal depression was measured using the Edinburgh Postnatal Depression Scale (EPDS). This is a validated 10-item self-report measure, designed and used to screen women for depression before and after pregnancy. The EPDS has an internal consistency coefficient of .87[2]. Items ask women to rate how they felt in the last week in relation to statements such as, 'Things have been getting on top of me'. A higher score is indicative of greater depressive symptoms, with a score of 13 and above considered indicative of depression; the cut-off of 13 is typically used to determine presence of clinical depression[33].

For peer review only

Supplemental Methods S2 Details of measurement sources for cardiometabolic risk factors

Details on measurement of height and weight at research clinics

Data from age 1 onwards are included in this analysis. We did not include measures before 1 year because of the difficulty in accurately modelling BMI from birth through the whole of childhood due to its early peak followed by adiposity rebound. From 1 to 5 years, measures were available from routine child health clinics for most children and extracted from health visitor records, which form part of standard child care in the UK. Data were also available from research clinic measurements on a random 10% subsample of the cohort. All cohort members were invited to research clinics from age 7 onwards. Across all ages parent-reported measures were available.

At the clinics, crown-heel length for children aged four to 25 months was measured using a Harpenden Neonatometer and from 25 months onwards standing height was measured using a Leicester Height Measure; weight was measured using Fereday 100kg combined scale (four-month clinic), Soenhle scale or Seca scale model 724 (eight-month clinic), Seca 724 or Seca 835 (12-month clinic), Seca 835 (18 months onwards). From age 7 years, all children were invited to annual clinics, at which standing height was measured to the last complete mm using the Harpenden Stadiometer and weight was measured to the nearest 0.1kg using the Tanita Body Fat Analyser (Model TBF 305).

Details on measurement of blood pressure

A Dinamap 9301 Vital Signs Monitor (Morton Medical, London) was used at 7, 9, 11, 15 and 18 years; an Omron MI-5 was used at the 10-year clinic; a Dinamap 8100 Vital Signs Monitor (Morton Medical) was used at the 12-year clinic.

Details on measurement of blood based biomarkers

Non-fasting glucose was measured at age 7 years using Nuclear Magnetic Resonance (NMR) spectroscopy. In a random 10% of the cohort at age 9 years, fasting glucose and insulin were also available; these were taken as part of a continuation of an earlier sub-study called "Child in Focus" that included approximately 10% of the overall cohort. Fasting glucose and insulin were available from research clinics held when participants were 15 and 18 years old[54, 55].

Plasma lipid assays (triglyceride and high-density lipoprotein cholesterol (HDL-c)) were performed by modification of the standard Lipid Research Clinics Protocol using enzymatic reagents for lipid determination. All assay coefficients of variation were <5%. Samples were collected after an overnight fast and were analysed by the hexokinase method. Insulin was measured by an ELISA (Mercodia, Uppsala, Sweden) that does not cross-react with proinsulin. All assay coefficients of variation were <5%.

Details on measurement on biomarkers using Nuclear Magnetic Resonance (NMR) spectroscopy

A comprehensive profiling of offspring circulating lipids, lipoproteins, and metabolites was done by a high-throughput NMR metabolomics platform, providing a snapshot of offspring serum metabolome. At age 7, this was done on fasted blood samples and glucose is included in our analyses. At age 15 and 18, non-fasted bloods were used.

For peer review only - http://bmjopen.bmj.com/site/about/guidelines.xhtml

Supplemental Methods S3 Details on measurement of confounders

Data on confounders was collected using self-report measures at 18 and/or 32 weeks gestation.

Household social class was measured as the highest of the mother's or her partner's occupational social class using data on job title and details of occupation collected about the mother and her partner from the mother's questionnaire at 32 weeks gestation. Social class was derived using the standard occupational classification (SOC) codes developed by the United Kingdom Office of Population Census and Surveys and classified as I professional, II managerial and technical, IIINM non-manual, IIIM manual, and IV&V part skilled occupations and unskilled occupation.

Parity data was collected using a self-report item asking 'How many times have you been pregnant altogether before this time?', with responses categorised as 0, 1 or 2 based on participant responses.

Maternal age at delivery was collected as self-report data and measured in years.

Maternal Education maternal education was reported at 32 weeks gestation according to increasing levels of achievement. These levels were: less than an Ordinary level (O Level), which was categorised as no education or a certificate of secondary education, subject-specific qualifications at a lower level than O levels that were obtained by age 16 years; O level, which are subject-specific qualifications generally obtained at age 16 years; Advanced level (A Level), which are subject-specific qualifications generally obtained at age 18 years; and university degree or above.

Maternal smoking during pregnancy was self-reported in response to the question "How many times per day did you smoke" at 18 weeks; and "How many cigarettes per day are you yourself smoking at the moment" at 32 weeks. Prenatal smoking was categorised as No smoking or Yes smoking in the current study based on responses to these questions.

Maternal pre-pregnancy BMI data was collected using a self-report measure at 12 weeks gestation. Women were asked "What was your weight before you started this pregnancy? (please indicate whether stones, pounds or kilos)" and "How tall are you? (Please indicate whether feet, inches or metres)". BMI was calculated as kg/m^2.

For peer review only

Supplemental Methods S4 Details of model selection for outcomes

Two approaches, fractional polynomials and linear splines were used in the modelling of trajectories as described previously.

Fractional polynomials were used for BMI, due to the complex pattern of change in BMI during childhood and adolescence. Fractional polynomials involve raising age to many combinations of powers, resulting in a wide range of possible curves and offering more flexibility than standard polynomial approaches.

Linear splines were used to model all other outcomes, as too few measurement occasions were available to permit modelling using fractional polynomials or other age term combinations. Linear splines allow knot points to be fitted at different ages to derive periods of change that are approximately linear. Models were derived by initially examining observed data for each risk factor. We also plotted mean values for each risk factor on each measurement occasion to assist on decisions regarding knot points. We compared observed and predicted measurements for a selection of suitable models for each risk factor. We examined rates of change between time periods in order to examine whether changes between periods were similar or different. In cases where rates of change between two spline periods appeared identical, the fit of models with reduced splines was explored. We also compared model fit statistics (Akaike's Information Criterion) for several models with different knot points (with knot points placed at whole years closest to mean age at clinics due to a greater density of measures). Systolic blood pressure (SBP) and diastolic blood pressure (DBP) models have been modelled previously and are described elsewhere in detail.

In all models, age (in years) was centred at the first available measure. For each risk factor except insulin, we included all participants with at least one measure of the risk factor in each multilevel model, under a missing at random (MAR) assumption, to minimise selection bias. The observations of participants who reported being pregnant at the 18-year clinic were excluded from the multilevel models at that time point only (n=6). Models for insulin included participants with at least one measure before and after 11 years of age to improve model fit due to the sparsity of measures at the earlier time points (birth and 9 years).

BMI has been modelled previously using fractional polynomials and is described elsewhere[34]. BMI was modelled from age 1 as BMI is not considered an appropriate measure of adiposity in infants. Briefly, BMI was log transformed due to skewness of the data and fractional polynomials were used where age was raised to various combinations of powers (each of the following single powers, plus each combination of two powers: 0.5, 1, 2, 3, -0.5, -1, -2, natural log), from which we selected the best fitting curve (the one with the lowest likelihood value). The resulting curve contained three age terms including log age, log age* age and log age *age$^{\wedge 2}$. To account for the likely reduced accuracy of parent-reported measurements, a binary indicator of measurement source (research clinic or health records versus parent-report) was included as a fixed effect. The variance of measurement occasion-level residuals (the differences between observed and predicted measurements) was allowed to vary with age for log BMI. The model took the form of: log $BMI_{ij} = (\beta_0+u_{0j}+e_{0ij}) + (\beta_1+u_{1j})(\ln(age)_{ij}) + (\beta_2+u_{2j})(age*\ln(age)_{ij}) + (\beta_3+u_{3j})(age^2*\ln(age)_{ij}) + (\beta_8+e_{1ij})(measurement\_source_{ij}) + e_{ij}(age\_months_{ij})$ where for person j at measurement occasion i; β's represent fixed effect coefficients, u_{0j} to u_{3j} indicate person-specific random effects for the intercept and linear, quadratic and cubic age terms respectively, and e_1 represents the occasion-specific residuals or measurement error which was allowed to vary with age and according to measurement source.

Fat mass and **lean mass** were measured on 5 occasions between 9 and 18 years. Fat mass was log transformed due to skewness of the data. Knots were placed at 13 and 15 resulting in three periods of change; from 9-13, 13-15, 15-18. Both models were adjusted for a time-and sex-varying height co-variate which was included as a fixed effect For lean mass, the covariance between the second spline from 13 to 15 and third spline from 15 to 18 was set to zero to improve model convergence. The models took the form of: log fat $mass_{ij}$ / lean $mass_{ij} = \beta_0 + u_{0j} + (\beta_1+ u_{1j})s_{ij1} + (\beta_2+ u_{2j})s_{ij2} + (\beta_3 + u_{3j})s_{ij3} + \beta_4$ (age&sex adjusted height covariate)$_{ij} + e_{ij}$ where for person j at measurement occasion i; β_0 represents the fixed effect coefficient for the average intercept, β_1 to β_3 represent fixed effect coefficients for the average linear slopes of each linear spline, β_4 represents the fixed effect coefficient for the average difference in measurements between individuals of different heights, u_{0j} to u_{3j} indicate person-specific random effects for the intercept and slopes respectively, and e_{ij} represents the occasion-specific residuals or measurement error which was allowed to vary with age.

SBP, DBP and **pulse rate** were measured at 7 time points from 7 to 18 years[35-38]. The knots for all models were placed at 12 and 16 resulting in three periods of change; from 7 to 12, 12-16 and 16-18. All models included a

For peer review only - http://bmjopen.bmj.com/site/about/guidelines.xhtml

fixed effect to adjust for the use of the use of an Omron MI-5 machine to measure SBP in 10-year clinic which differed from all other clinics and a binary time indicator as a level 1 random effect of age less than or greater than 10 years to account for changing measurement error with age. The models took the form of: SBP_{ij} /DBP_{ij} /$pulse_{ij} = \beta_0 + u_{0j} + (\beta_1 + u_{1j})s_{ij1} + (\beta_2 + u_{2j})s_{ij2} + (\beta_3 + u_{3j})s_{ij3} + \beta_4(machine)_{ij} + e_{ij}(age\_binary_{ij})$ where for person j at measurement occasion i; β_0 represents the fixed effect coefficient for the average intercept, β_1 to β_3 represent fixed effect coefficients for the average linear slopes of each linear spline, β_4 represents the fixed effect coefficient for the average difference in measurements between the machine used at the 10 year clinic compared to the machine used at other clinics, u_{0j} to u_{3j} indicate person-specific random effects for the intercept and slopes respectively, and e_{ij} represents the occasion-specific residuals or measurement error which was allowed to vary with age.

Triglyceride and **HDL-c** were measured 5 times from birth to 18 years. Triglyceride was log transformed due to the skewness of the data. **Non-HDL-c** was derived by subtracting HDL-c from total cholesterol. Knots for triglyceride and non-HDL-c were placed at 9 and 15 years resulting in two periods of change; from birth to 9 years and 9 to 18. Knots were placed at age 7 and 15 years for HDL-c resulting in two periods of change; from birth to 7 years and 7 - 18 years. The models took the form of: log $triglyceride_{ij}$/$HDL\text{-}c_{ij}$/$non\text{-}HDL\text{-}c_{ij} = \beta_0 + u_{0j} + (\beta_1 + u_{1j})s_{ij1} + (\beta_2 + u_{2j})s_{ij2} + e_{ij}$ where for person j at measurement occasion i; β_0 represents the fixed effect coefficient for the average intercept, β_1 and β_2 represent fixed effect coefficients for the average linear slopes of each linear spline, u_{0j} to u_{3j} indicate person-specific random effects for the intercept and slopes respectively, and e_{ij} represents the occasion-specific residuals or measurement error.

Glucose was measured on four occasions (7, 9, 15, and 18). A knot was placed at 15 resulting in two periods of change; from 7 to 15 and 15 to 18. Due to few available repeated measures of glucose, we modelled the person-specific random effects as a single linear slope rather than a function of the splines as was done in all other linear spline models. This allowed person specific variation from the average trajectory but under the assumption that person-specific deviation from the mean trajectory was constant over time. The model took the form of: $glucose_{ij} = \beta_0 + u_{0j} + (\beta_1)s_{ij1} + (\beta_2)s_{ij2} + u_{1j}*age + e_{ij}$ where for person j at measurement occasion i; β_0 represents the fixed effect coefficient for the average intercept, β_1 and β_2 represent fixed effect coefficients for the average linear slopes of each linear spline, u_{0j} to u_{1j} indicate person-specific random effects for the intercept and slope respectively, and e_{ij} represents the occasion-specific residuals or measurement error.

Insulin was measured on four occasions (birth, 9, 15, and 18). Due to the sparsity of measures at birth and 9 years, the model for insulin was restricted to participants with at least one measure before and after age 11, to improve model fit. Knots were placed at 9 and 15 resulting in three periods of change; from birth to 9 years, 9 to 15 and 15 to 18. Insulin was log transformed due to skewness of the data. The model took the form of: log $insulin_{ij} = \beta_0 + u_{0j} + (\beta_1 + u_{1j})s_{ij1} + (\beta_2 + u_{2j})s_{ij2} + (\beta_3 + u_{3j}))s_{ij3+}\ e_{ij}$ where for person j at measurement occasion i; β_0 represents the fixed effect coefficient for the average intercept, β_1 to β_3 represent fixed effect coefficients for the average linear slopes of each linear spline, u_{0j} to u_{3j} indicate person-specific random effects for the intercept and slopes respectively, and e_{ij} represents the occasion-specific residuals or measurement error.

For peer review only

Supplemental Table S1 Model details for log BMI trajectories

	No of contributing individuals		Assessment of model fit			
	Total number of observations	Number of individuals with 1 measure	Mean observed BMI, log (kg/m^2) (SD) [a]	Mean predicted BMI, log (kg/m^2) (SD) [a]	Mean difference (observed – predicted), log (kg/m^2) [a]	95% level of agreement between observed and predicted, log (kg/m^2) [a]
**Overall**	80796	8606				
**1-3 years**	11042	7131	2.84 (0.09)	2.84 (0.06)	-0.01	-0.17 to 0.15
**3-7 years**	21216	7734	2.78 (0.10)	2.79 (0.07)	-0.01	-0.17 to 0.15
**7-9 years**	7033	6077	2.77 (0.12)	2.80 (0.10)	-0.03	-0.17 to 0.10
**9-11 years**	9850	5828	2.84 (0.14)	2.85 (0.12)	-0.01	-0.12 to 0.10
**11-13 years**	11069	5318	2.90 (0.16)	2.90 (0.13)	0.0002	-0.11 to 0.12
**13-15 years**	12118	5312	2.97 (0.16)	2.97 (0.14)	0.0002	-0.13 to 0.13
**15-18 years**	8468	4518	3.07 (0.16)	3.09 (0.15)	-0.02	-0.14 to 0.10

BMI, Body Mass Index; SD, standard deviation

[a] BMI is natural log transformed. All values are in log form.

For peer review only

Supplemental Table S2 Model details for log fat mass trajectories

	No of contributing individuals		Assessment of model fit			
	Total number of observations	Number of individuals with 1 measure	Mean observed fat mass, log (kg) (SD) [a]	Mean predicted fat mass, log (kg) (SD)[a]	Mean difference (observed – predicted), log (kg)[a]	95% level of agreement between observed and predicted, log (kg)[a]
**Overall**	21615	6032				
**9 years**	5134	5134	1.97 (0.57)	1.97 (0.54)	-0.01	-0.18 to 0.17
**9-13 years**	10099	10099	2.13 (0.59)	2.12 (0.56)	0.01	-0.20 to 0.21
**13-15 years**	4371	4371	2.44 (0.59)	2.46 (0.55)	-0.02	-0.28 to 0.24
**15-18 years**	7145	7145	2.63 (0.60)	2.63 (0.57)	0.01	-0.19 to 0.20

SD, standard deviation
[a] Fat mass is natural log transformed. All values are in log form.

For peer review only

Supplemental Table S3 Model details for lean mass trajectories

	No of contributing individuals		Assessment of model fit			
	Total number of observations	Number of individuals with 1 measure	Mean observed lean mass, kg (SD)	Mean predicted lean mass, kg (SD)	Mean difference (observed – predicted), kg	95% level of agreement between observed and predicted, kg
**Overall**	21615	6032				
**9 years**	5134	5134	24.56 (3.16)	24.57 (2.81)	-0.01	-2.53 to 2.52
**9-13 years**	10099	10099	27.10 (4.57)	27.11 (4.27)	0.00	-2.45 to 2.44
**13-15 years**	4371	4371	38.01 (6.41)	38.01 (5.97)	0.00	-2.94 to 2.95
**15-18 years**	7145	7145	44.35 (9.26)	44.35 (9.06)	0.003	-1.92 to 1.92

SD, standard deviation

For peer review only

For peer review only - http://bmjopen.bmj.com/site/about/guidelines.xhtml

Supplemental Table S4 Model details for SBP, DBP and pulse rate trajectories

	No of contributing individuals		Assessment of model fit			
	Total number of observations	Number of individuals with 1 measure	Mean observed SBP, DBP or pulse rate, (SD) [a]	Mean predicted SBP, DBP or pulse rate, (SD) [a]	Mean difference (observed – predicted) [a]	95% level of agreement between observed and predicted [a]
**SBP**						
**Overall**	32900	6671				
**7 years**	5672	5672	98.76 (9.13)	98.62 (5.37)	0.15	-10.70 to 11.00
**7-12 years**	20670	6479	102.50 (9.50)	102.70 (6.21)	-0.20	-11.80 to 11.40
**12-16 years**	8694	5090	115.82 (11.77)	115.35 (8.84)	0.46	-11.31 to 12.24
**16-18 years**	3536	3428	116.82 (10.15)	117.14 (7.44)	-0.31	-12.48 to 11.85
**DBP**						
**Overall**	32900	6671				
**7 years**	5672	5672	56.34 (6.55)	56.56 (3.37)	-0.22	-8.90 to 8.46
**7-12 years**	20670	6479	57.99 (6.92)	56.88 (3.39)	1.11	-9.09 to 11.31
**12-16 years**	8694	5090	61.07 (9.61)	61.92 (5.07)	-0.86	-12.62 to 10.91
16-18 years	3536	3428	64.17 (6.06)	64.49 (3.68)	-0.32	-13.14 to 12.50
**Pulse**						
**Overall**	32900	6671				
**7 years**	5672	5672	83.18 (10.65)	83.15 (6.11)	0.03	-13.08 to 13.13
**7-12 years**	20670	6479	77.57 (11.54)	78.91 (7.01)	-1.34	-15.93 to 13.25
**12-16 years**	8694	5090	74.00 (11.29)	74.08 (7.03)	-0.08	-13.88 to 13.72
**16-18 years**	3536	3428	65.59 (10.04)	65.85 (6.29)	-0.27	-14.69 to 14.16

DBP, diastolic blood pressure; SBP, systolic blood pressure; SD, standard deviation.
[a]Units are in mmHg for SBP and DBP and bpm for pulse rate.

For peer review only

Supplemental Table S5 Model details for glucose trajectories

	No of contributing individuals		Assessment of model fit			
	Total number of observations	Number of individuals with 1 measure	Mean observed glucose, mmol/l (SD)	Mean predicted glucose, mmol/l (SD)	Mean difference (observed – predicted), mmol/l	95% level of agreement between observed and predicted, mmol/l
**Overall**	9390	5045				
**7 years**	3907	3907	4.18 (0.50)	4.22 (0.24)	-0.03	-0.59 to 0.53
**7-15 years**	4576	4065	4.29 (0.55)	4.28 (0.27)	0.01	-0.59 to 0.61
**15-18 years**	4812	3203	5.12 (0.39)	5.13 (0.17)	-0.01	-0.65 to 0.62

Mmol/l, millimoles per litre; SD, standard deviation

For peer review only

Supplemental Table S6 Model details for log insulin and log triglyceride trajectories

	No of contributing individuals		Assessment of model fit			
	Total number of observations	Number of individuals with 1 measure	Mean observed triglycerides, (SD) [a]	Mean predicted triglycerides, (SD) [a]	Mean difference (observed – predicted)[a]	95% level of agreement between observed and predicted
**Log insulin**						
**Overall**	1464	526				
**Birth**	206	206	1.05 (0.48)	1.04 (0.17)	0.01	-0.63 to 0.64
**0-9 years**	206	206	1.05 (0.48)	1.04 (0.17)	0.01	-0.63 to 0.64
**9-15 years**	445	443	1.58 (0.62)	1.56 (0.39)	0.02	-0.48 to 0.52
**9-18 years**	813	523	2.07 (0.53)	2.07 (0.27)	0.0001	-0.63 to 0.63
**Log triglyceride**						
**Overall**	15239	6641				
**Birth**	2963	2963	-0.71 (0.41)	-0.71 (0.15)	-0.001	-0.52 to 0.51
**0-9 years**	6808	5581	-0.34 (0.53)	-0.35 (0.36)	0.002	-0.56 to 0.56
**9-18 years**	8431	4595	-0.14 (0.41)	-0.13 (0.22)	-0.002	-0.56 to 0.55

SD, standard deviation

[a] Insulin and triglyceride are natural log transformed. All values are in log form. Units for log insulin are in milliunits per litre. Units for log triglyceride are in millimoles per litre.

For peer review only

Supplemental Table S7 Model details for HDL-c and non-HDL-c trajectories

	No of contributing individuals		Assessment of model fit			
	Total number of observations	Number of individuals with 1 measure	Mean observed HDL-c, mmol/l (SD)	Mean predicted HDL-c, mmol/l (SD)	Mean difference (observed – predicted), mmol/l	95% level of agreement between observed and predicted, mmol/l
**Overall**	15239	6641				
**Birth**	2963	2963	0.52 (0.24)	0.52 (0.11)	-0.000002	-0.24 to 0.24
**0-7 years**	6808	2965	1.09 (0.56)	1.08 (0.52)	0.01	-0.24 to 0.25
**7-18 years**	12274	5466	1.39 (0.32)	1.39 (0.24)	-0.00002	-0.25 to 0.25
**Overall**	15239	6641				
**Birth**	2963	2963	1.22 (0.53)	1.25 (0.25)	-0.02	-0.59 to 0.54
**0-9 years**	6808	5581	2.15 (1.00)	2.10 (0.82)	0.05	-0.53 to 0.63
**9-18 years**	8431	4595	2.63 (0.65)	2.67 (0.52)	-0.04	-0.59 to 0.51

HDL-c, high density lipoprotein cholesterol; Non-HDL-c, non-high density lipoprotein cholesterol; Mmol/l, millimoles per litre; SD, standard deviation.

For peer review only

Supplemental Table S8 Number of participants with cardiometabolic health outcome measures at each time point

	Birth	Age 1	Age 7	Age 9	Age 10	Age 11	Age 12	Age 13	Age 15	Age 18
BMI [a]		x	x	x	x	x	x	x	x	x
Fat/lean mass				5,134		4,958		4,348	3,738	3,437
SBP/DBP/pulse rate			5,672	5,347	5,084	4,970	4,729		3,787	3,311
Glucose			3,907	662					2,485	2,336
Lipids [b]	2,863		3,846	3,617					2,485	2,328
Insulin	206		440						417	401

DBP, diastolic blood pressure; SBP, systolic blood pressure.

[a] Measures available at each of these approximate ages and at several ages in between but exact timing and number of BMI measures not shown as measures were available from questionnaires, routine child health records and research clinics at different mean ages from 1 to 18 years.

[b] Lipids include triglyceride, high density lipoprotein cholesterol (HDL-c) and non-HDL-c.

For peer review only

Supplemental Table S9 Characteristics at birth of the mothers of children included in models compared with those excluded due to missing exposure, outcome or confounder data

	Participants included n= 8,606	Participants excluded n= 5,261 – 13,341 [a]
	n (%)	n (%)
**Household social class**		
Professional	1190(13.8)	346(11.7)
Managerial & Technical	3673(42.7)	1148(38.9)
Non-Manual	2254(26.2)	690(23.4)
Manual	1037(12.0)	525(17.8)
Part Skilled & Unskilled	452(5.3)	240(8.1)
**Maternal education**		
Less than O level	2124(24.7)	1624(42.1)
O level	3167(36.8)	1151(29.8)
A level	2093(24.3)	700(18.1)
Degree or above	1222(14.2)	382(9.9)
**Partners highest educational qualification**		
Less than O level	2488(29.8)	1655(45.4)
O level	1872(22.4)	679(18.6)
A level	2337(28.0)	778(21.4)
Degree or Above	1643(19.7)	530(14.6)
**Maternal smoking during pregnancy**		
No	6781(78.8)	3171(67.7)
Yes	1825(21.2)	1516(32.3)
**Parity**		
0	3893(45.2)	1947(43.7)
1	3159(36.7)	1413(31.7)
2	1554(18.1)	1094(24.6)
	**Mean (SD)**	**Mean (SD)**
Child gestational age at birth	39.5(1.8)	37(8.1)
Birthweight (g)	3426(533)	3310(643)
Maternal BMI (kg/m^2)	23.0(3.8)	23(4.0)
Maternal age (years)	28.7(4.7)	27(5.2)

SD, standard deviation
[a] Denominators for excluded participants in this table vary due to different rates of missing data for characteristics shown.

For peer review only

Supplemental Table S10 Mean trajectories of BMI estimated from multilevel models, by maternal anxiety during pregnancy

	Mean trajectory (95% CI) in males (no anxiety during pregnancy) log (kg/m^2) (reference) [a]	Mean trajectory (95% CI) in females (no anxiety during pregnancy) log (kg/m^2) (reference) [a]	Mean difference in trajectory (95% CI) comparing anxiety at 18 weeks only with the reference trajectory (% difference in kg/m^2) [b]	Mean difference in trajectory (95% CI) anxiety at 32 weeks only with the reference trajectory (% difference in kg/m^2) [b]	Mean difference in trajectory (95% CI) comparing anxiety at 18 and 32 weeks with the reference trajectory (% difference in kg/m^2) [b]
**Unadjusted BMI**					
Age 1yr	2.90 (2.89,2.90)	2.89 (2.88,2.89)	0.42 (-1.00,1.84)	0.13 (-1.15,1.40)	0.3 (-0.85,1.53)
Age 3yr	2.77 (2.76,2.77)	2.75 (2.75,2.75)	0.65 (-0.08,1.38)	0.11 (-0.56,0.77)	0.0 (-0.60,0.60)
Age 7yr	2.79 (2.78,2.79)	2.80 (2.79,2.80)	0.95 (-0.01,1.91)	0.44 (-0.43,1.32)	0.6 (-0.20,1.39)
Age 9yr	2.82 (2.82,2.83)	2.84 (2.84,2.85)	1.06 (-0.11,2.24)	0.64 (-0.43,1.71)	0.9 (-0.06,1.89)
Age 11yr	2.87 (2.87,2.88)	2.90 (2.90,2.91)	1.16 (-0.18,2.50)	0.83 (-0.39,2.06)	1.2 (0.08,2.32)
Age 13yr	2.94 (2.93,2.95)	2.97 (2.96,2.97)	1.23 (-0.21,2.66)	1.03 (-0.28,2.34)	1.4 (0.24,2.65)
Age 15yr	3.02 (3.01,3.02)	3.04 (3.04,3.05)	1.27 (-0.23,2.76)	1.22 (-0.16,2.60)	1.6 (0.37,2.90)
Age 18yr	3.16 (3.15,3.16)	3.17 (3.16,3.18)	1.27 (-0.51,3.05)	1.50 (-0.16,3.17)	1.8 (0.29,3.33)
**Adjusted BMI**					
Age 1yr	2.88 (2.87,2.90)	2.87 (2.86,2.89)	0.36 (-1.06,1.79)	0.18 (-1.11,1.47)	0.3 (-0.91,1.51)
Age 3yr	2.77 (2.76,2.77)	2.75 (2.74,2.76)	0.40 (-0.32,1.13)	0.02 (-0.65,0.68)	-0.2 (-0.76,0.44)
Age 7yr	2.78 (2.78,2.79)	2.80 (2.79,2.81)	0.43 (-0.49,1.34)	0.27 (-0.57,1.11)	0.4 (-0.36,1.17)
Age 9yr	2.82 (2.81,2.83)	2.84 (2.83,2.86)	0.41 (-0.70,1.53)	0.39 (-0.63,1.41)	0.7 (-0.28,1.60)
Age 11yr	2.87 (2.86,2.89)	2.90 (2.89,2.91)	0.38 (-0.88,1.64)	0.49 (-0.66,1.65)	0.8 (-0.22,1.91)
Age 13yr	2.94 (2.92,2.95)	2.97 (2.95,2.98)	0.33 (-1.00,1.67)	0.57 (-0.66,1.80)	0.9 (-0.20,2.07)
Age 15yr	3.02 (3.00,3.03)	3.04 (3.03,3.06)	0.26 (-1.13,1.65)	0.61 (-0.67,1.90)	0.9 (-0.27,2.10)
Age 18yr	3.16 (3.14,3.18)	3.17 (3.15,3.19)	0.12 (-1.55,1.79)	0.61 (-0.96,2.19)	0.7 (-0.76,2.13)

CI, confidence interval; yr, year.

[a] BMI is presented in the natural log and values represent the mean predicted natural log of BMI at each age shown.

[b] Differences at each age are back transformed from the log scale and are interpreted as the percentage difference in the mean level in original units at each age comparing each category with the reference trajectory.

For peer review only

Supplemental Table S11 Mean trajectories of fat mass estimated from multilevel models, by maternal anxiety during pregnancy

	Mean trajectory (95% CI) in males (no anxiety during pregnancy) log (kg or kg/yr) (reference) [a]	Mean trajectory (95% CI) in females (no anxiety during pregnancy) log (kg or kg/yr) (reference) [a]	Mean difference in trajectory (95% CI) comparing anxiety at 18 weeks only with the reference trajectory (% or %/yr) [b]	Mean difference in trajectory (95% CI) comparing anxiety at 32 weeks only with the reference trajectory (% or %/yr) [b]	Mean difference in trajectory (95% CI) comparing anxiety at 18 and 32 weeks with the reference trajectory (% or %/yr) [b]
**Unadjusted**					
Age 9yr (kg) or (%)	1.79 (1.76,1.82)	2.63 (2.11,3.15)	3.62 (-2.42,9.67)	-1.46 (-6.78,3.87)	0.94 (-4.08,5.95)
Change 9-13yr (kg/yr) or (%/yr)	0.11 (0.11,0.12)	0.16 (0.15,0.17)	0.04 (-1.18,1.26)	1.00 (-0.15,2.15)	1.52 (0.44,2.60)
Change 13-15yr (kg/yr) or (%/yr)	-0.06 (-0.07,-0.05)	0.10 (0.09,0.11)	1.41 (-0.84,3.66)	0.21 (-1.86,2.28)	-1.62 (-3.49,0.26)
Change 15-18yr (kg/yr) or (%/yr)	0.10 (0.09,0.11)	0.06 (0.05,0.06)	-1.41 (-3.04,0.21)	-0.93 (-2.51,0.64)	0.81 (-0.65,2.27)
Age 18yr (kg) or (%)	2.43 (2.41,2.46)	3.64 (3.09,4.18)	2.28 (-3.83,8.40)	0.11 (-5.56,5.79)	6.33 (0.86,11.79)
**Adjusted**					
Age 9yr (kg) or (%)	1.81 (1.74,1.87)	2.66 (2.13,3.18)	1.56 (-4.21,7.32)	-2.02 (-7.17,3.14)	1.00 (-3.92,5.91)
Change 9-13yr (kg/yr) or (%/yr)	0.11 (0.09,0.12)	0.15 (0.14,0.17)	-0.13 (-1.34,1.08)	0.74 (-0.40,1.89)	1.19 (0.12,2.27)
Change 13-15yr (kg/yr) or (%/yr)	-0.07 (-0.09,-0.04)	0.09 (0.07,0.12)	1.56 (-0.70,3.82)	0.21 (-1.87,2.28)	-1.75 (-3.64,0.14)
Change 15-18yr (kg/yr) or (%/yr)	0.11 (0.09,0.12)	0.06 (0.05,0.08)	-1.47 (-3.10,0.16)	-0.93 (-2.51,0.64)	0.82 (-0.66,2.29)
Age 18yr (kg) or (%)	2.42 (2.35,2.48)	3.63 (3.08,4.18)	-0.32 (-6.10,5.45)	-1.46 (-6.88,3.97)	4.77 (-0.51,10.05)

CI, confidence interval; kg/yr, kilograms per year; %/yr, percentage per year.

[a] Fat mass was transformed using the natural log. All predicted mean values (kg) and rates of change per year (kg/yr) for the reference categories are on the log scale

[b] The difference between groups is back transformed from the log scale for ease of interpretation and is interpreted as the percentage difference in the mean level in original units comparing each category with the reference or percentage difference in change in original units per year (%/yr) comparing each category with the reference.

For peer review only - http://bmjopen.bmj.com/site/about/guidelines.xhtml

Supplemental Table S12 Mean trajectories of blood pressure and pulse rate estimated from multilevel models, by maternal anxiety during pregnancy

	Mean trajectory (95% CI) in males (no anxiety during pregnancy) (reference)	Mean trajectory (95% CI) in females (no anxiety during pregnancy) (reference)	Mean difference in trajectory (95% CI) comparing anxiety at 18 weeks only with the reference trajectory	Mean difference in trajectory (95% CI) comparing anxiety at 32 weeks only with the reference trajectory	Mean difference in trajectory (95% CI) comparing anxiety at 18 and 32 weeks with the reference trajectory
**SBP**					
**Unadjusted**					
Age 7yr (mmHg)	97.76 (97.39,98.12)	97.86 (97.48,98.23)	0.67 (-0.26,1.61)	0.34 (-0.52,1.20)	0.37 (-0.41,1.15)
Change 7-12yr (mmHg/yr)	1.65 (1.56,1.74)	1.87 (1.78,1.95)	0.08 (-0.14,0.31)	-0.01 (-0.22,0.19)	-0.07 (-0.25,0.12)
Change 12-16yr (mmHg/yr)	5.86 (5.70,6.01)	3.85 (3.70,4.00)	0.02 (-0.35,0.40)	0.02 (-0.33,0.38)	-0.04 (-0.36,0.28)
Change 16-18yr (mmHg/yr)	-4.06 (-4.40,-3.71)	-5.94 (-6.27,-5.61)	0.13 (-0.71,0.96)	-0.04 (-0.83,0.74)	0.42 (-0.30,1.15)
Age 18yr (mmHg)	121.32 (120.84,121.80)	110.73 (110.27,111.18)	1.44 (0.27,2.61)	0.28 (-0.82,1.38)	0.74 (-0.27,1.75)
**Adjusted**					
Age 7yr (mmHg)	98.91 (97.92,99.90)	99.04 (98.04,100.04)	0.53 (-0.40,1.47)	0.16 (-0.70,1.02)	0.23 (-0.55,1.01)
Change 7-12yr (mmHg/yr)	1.47 (1.24,1.71)	1.69 (1.45,1.93)	0.07 (-0.15,0.29)	-0.01 (-0.22,0.19)	-0.06 (-0.25,0.13)
Change 12-16yr (mmHg/yr)	5.97 (5.57,6.37)	3.97 (3.56,4.37)	0.04 (-0.33,0.42)	0.06 (-0.30,0.41)	-0.0001 (-0.32,0.32)
Change 16-18yr (mmHg/yr)	-4.24 (-5.16,-3.32)	-6.15 (-7.07,-5.23)	0.04 (-0.80,0.88)	-0.16 (-0.95,0.63)	0.22 (-0.52,0.95)
Age 18yr (mmHg)	121.67 (120.40,122.95)	111.07 (109.79,112.35)	1.16 (-0.02,2.33)	0.01 (-1.10,1.11)	0.38 (-0.64,1.40)
**DBP**					
**Unadjusted**					
Age 7yr (mmHg)	56.06 (55.80,56.32)	56.91 (56.64,57.18)	0.74 (0.06,1.41)	0.15 (-0.47,0.78)	0.40 (-0.17,0.96)
Change 7-12yr (mmHg/yr)	0.14 (0.07,0.21)	0.12 (0.05,0.19)	-0.02 (-0.19,0.16)	0.05 (-0.11,0.21)	-0.02 (-0.17,0.12)
Change 12-16yr (mmHg/yr)	2.87 (2.74,3.00)	2.34 (2.21,2.47)	-0.06 (-0.38,0.26)	-0.03 (-0.33,0.27)	-0.17 (-0.45,0.10)
Change 16-18yr (mmHg/yr)	-2.59 (-2.88,-2.30)	-1.17 (-1.45,-0.89)	0.27 (-0.43,0.98)	0.27 (-0.40,0.94)	0.46 (-0.14,1.07)
Age 18yr (mmHg)	63.05 (62.70,63.40)	63.65 (62.85,64.46)	0.97 (0.13,1.81)	0.84 (0.05,1.64)	0.52 (-0.20,1.24)
**Adjusted**					
Age 7yr (mmHg)	56.79 (56.08,57.51)	57.65 (56.93,58.38)	0.70 (0.02,1.38)	0.08 (-0.55,0.70)	0.33 (-0.24,0.90)
Change 7-12yr (mmHg/yr)	0.05 (-0.13,0.23)	0.03 (-0.15,0.22)	-0.02 (-0.19,0.15)	0.05 (-0.11,0.21)	-0.02 (-0.16,0.13)
Change 12-16yr (mmHg/yr)	2.95 (2.60,3.30)	2.43 (2.08,2.78)	-0.02 (-0.34,0.30)	0.03 (-0.27,0.33)	-0.09 (-0.37,0.19)
Change 16-18yr (mmHg/yr)	-2.73 (-3.52,-1.95)	-1.34 (-2.12,-0.55)	0.19 (-0.52,0.89)	0.11 (-0.56,0.78)	0.22 (-0.40,0.84)
Age 18yr (mmHg)	63.39 (62.46,64.31)	64.03 (62.85,65.21)	0.89 (0.05,1.73)	0.67 (-0.13,1.46)	0.31 (-0.42,1.04)
**Pulse rate**					
**Unadjusted**					
Age 7yr (bpm)	82.33 (81.90,82.75)	85.59 (85.16,86.03)	0.62 (-0.47,1.71)	1.18 (0.17,2.19)	0.68 (-0.23,1.59)
Change 7-12yr (bpm/yr)	-1.89 (-1.99,-1.79)	-1.73 (-1.83,-1.63)	0.01 (-0.26,0.27)	-0.30 (-0.54,-0.06)	-0.08 (-0.30,0.14)
Change 12-16yr (bpm/yr)	-0.71 (-0.87,-0.55)	-0.25 (-0.41,-0.10)	-0.09 (-0.48,0.30)	0.24 (-0.13,0.61)	0.17 (-0.16,0.51)
Change 16-18yr (bpm/yr)	-4.21 (-4.57,-3.85)	-4.83 (-5.17,-4.49)	0.62 (-0.24,1.49)	-0.03 (-0.84,0.78)	-0.04 (-0.78,0.70)
Age 18yr (bpm)	61.62 (61.10,62.15)	66.27 (65.77,66.76)	1.53 (0.26,2.80)	0.57 (-0.62,1.77)	0.92 (-0.17,2.01)
**Adjusted**					
Age 7yr (bpm)	82.57 (81.42,83.73)	85.80 (84.62,86.97)	0.62 (-0.47,1.71)	1.13 (0.12,2.14)	0.68 (-0.24,1.60)
Change 7-12yr (bpm/yr)	-1.90 (-2.18,-1.62)	-1.73 (-2.01,-1.45)	0.01 (-0.25,0.27)	-0.29 (-0.53,-0.05)	-0.06 (-0.29,0.16)
Change 12-16yr (bpm/yr)	-1.05 (-1.47,-0.63)	-0.60 (-1.03,-0.17)	-0.09 (-0.48,0.30)	0.22 (-0.15,0.58)	0.14 (-0.20,0.48)
Change 16-18yr (bpm/yr)	-3.67 (-4.62,-2.73)	-4.34 (-5.29,-3.39)	0.58 (-0.28,1.45)	-0.09 (-0.90,0.73)	-0.14 (-0.89,0.61)
Age 18yr (bpm)	61.52 (60.13,62.91)	66.06 (64.67,67.45)	1.48 (0.21,2.75)	0.37 (-0.83,1.56)	0.65 (-0.45,1.75)

bpm, beats per minute; bpm/yr, beats per minute per year; CI, confidence interval; mmHg, millimetres of mercury; mmHg/yr, millimetres of mercury per year.

For peer review only - http://bmjopen.bmj.com/site/about/guidelines.xhtml

Supplemental Table S13 Mean trajectories of lean mass estimated from multilevel models, by maternal anxiety during pregnancy

	Mean trajectory (95% CI) in males (no anxiety during pregnancy) (reference)	Mean trajectory (95% CI) in females (no anxiety during pregnancy) (reference)	Mean difference in trajectory (95% CI) comparing anxiety at 18 weeks only with the reference trajectory	Mean difference in trajectory (95% CI) comparing anxiety at 32 weeks only with the reference trajectory	Mean difference in trajectory (95% CI) comparing anxiety at 18 and 32 weeks with the reference trajectory
**Unadjusted**					
Age 9yr (kg)	23.89 (23.76,24.03)	20.78 (20.65,20.91)	-0.12 (-0.42,0.18)	-0.28 (-0.56,0.002)	-0.13 (-0.38,0.13)
Change 9-13yr (kg/yr)	2.30 (2.24,2.36)	3.22 (3.17,3.28)	0.11 (-0.02,0.24)	-0.10 (-0.22,0.02)	0.04 (-0.07,0.15)
Change 13-15yr (kg/yr)	7.69 (7.59,7.79)	1.58 (1.46,1.69)	-0.33 (-0.57, -0.08)	0.09 (-0.14,0.32)	0.003 (-0.21,0.21)
Change 15-18yr (kg/yr)	2.47 (2.40,2.55)	0.38 (0.31,0.45)	-0.0004 (-0.18,0.18)	0.04 (-0.13,0.21)	-0.03 (-0.18,0.13)
Age 18yr (kg)	55.91 (55.67,56.15)	37.96 (37.72,38.20)	-0.32 (-0.91,0.27)	-0.39 (-0.95,0.17)	-0.04 (-0.54,0.47)
**Adjusted**					
Age 9yr (kg)	23.84 (23.52,24.17)	20.76 (20.43,21.08)	-0.14 (-0.44,0.16)	-0.26 (-0.53,0.02)	-0.05 (-0.31,0.20)
Change 9-13yr (kg/yr)	2.30 (2.16,2.44)	3.22 (3.08,3.36)	0.09 (-0.04,0.22)	-0.10 (-0.22,0.01)	0.04 (-0.07,0.15)
Change 13-15yr (kg/yr)	7.48 (7.22,7.74)	1.36 (1.09,1.63)	-0.32 (-0.57,-0.07)	0.09 (-0.14,0.32)	0.01 (-0.21,0.22)
Change 15-18yr (kg/yr)	2.63 (2.44,2.82)	0.54 (0.35,0.73)	0.01 (-0.17,0.19)	0.05 (-0.12,0.22)	-0.02 (-0.17,0.14)
Age 18yr (kg)	55.90 (55.27,56.54)	37.99 (37.35,38.63)	-0.38 (-0.96,0.21)	-0.34 (-0.90,0.22)	0.07 (-0.44,0.58)

kg/yr, kilograms per year.

For peer review only

Supplemental Table S14 Mean trajectories of glucose estimated from multilevel models, by maternal anxiety during pregnancy

	Mean trajectory (95% CI) in males (no anxiety during pregnancy) (reference)	Mean trajectory (95% CI) in females (no anxiety during pregnancy) (reference)	Mean difference in trajectory (95% CI) comparing anxiety at 18 weeks only with the reference trajectory	Mean difference in trajectory (95% CI) comparing anxiety at 32 weeks only with the reference trajectory	Mean difference in trajectory (95% CI) c comparing anxiety at 18 and 32 weeks with the reference trajectory
**Unadjusted**					
Age 7yr (mmol/l)	4.19 (4.17,4.21)	4.13 (4.10,4.15)	-0.06 (-0.12,0.005)	-0.003 (-0.06,0.06)	0.03 (-0.02,0.08)
Change 7-15yr (mmol/l/yr)	0.15 (0.14,0.15)	0.13 (0.13,0.14)	0.007 (-0.005,0.02)	0.001 (-0.01,0.01)	-0.004 (-0.01,0.01)
Change 15-18yr (mmol/l/yr)	-0.08 (-0.09, -0.07)	-0.10 (-0.11, -0.09)	-0.003 (-0.04,0.03)	-0.02 (-0.05,0.01)	0.01 (-0.02,0.04)
Age 18yr (mmol/l)	5.11 (5.09,5.14)	4.89 (4.86,4.92)	-0.01 (-0.08,0.05)	-0.04 (-0.11,0.02)	0.03 (-0.02,0.09)
**Adjusted**					
Age 7yr (mmol/l)	4.21 (4.14,4.27)	4.14 (4.07,4.21)	-0.06 (-0.13,0.003)	-0.005 (-0.06,0.05)	0.04 (-0.02,0.09)
Change 7-15yr (mmol/l/yr)	0.14 (0.13,0.16)	0.13 (0.12,0.14)	0.01 (-0.005,0.02)	0.001 (-0.01,0.01)	-0.004 (-0.01,0.01)
Change 15-18yr (mmol/l/yr)	-0.08 (-0.11, -0.04)	-0.10 (-0.13, -0.06)	-0.003 (-0.04,0.03)	-0.02 (-0.05,0.02)	0.01 (-0.02,0.04)
Age 18yr (mmol/l)	5.13 (5.06,5.20)	4.91 (4.84,4.98)	-0.02 (-0.08,0.05)	-0.04 (-0.11,0.02)	0.04 (-0.02,0.09)

CI, confidence interval; mmol/l, millimole per litre; mmol/l/year, millimoles per litre per year.

For peer review only

Supplemental Table S15 Mean trajectories of insulin and triglyceride estimated from multilevel models, by maternal anxiety during pregnancy

	Mean trajectory (95% CI) in males (no anxiety during pregnancy) (reference) [a]	Mean trajectory (95% CI) in females (no anxiety during pregnancy) (reference) [a]	Mean difference in trajectory (95% CI) comparing anxiety at 18 weeks only with the reference trajectory (% or %/yr) [b]	Mean difference in trajectory (95% CI) comparing anxiety at 32 weeks only with the reference trajectory (% or %/yr) [b]	Mean difference in trajectory (95% CI) comparing anxiety at 18 and 32 weeks with the reference trajectory (% or %/yr) [b]
**Insulin**					
**Unadjusted**					
Birth (mu/l or %)	0.99 (0.89,1.09) 0.04	1.08 (0.99,1.18)	-7.29 (-31.57,16.99)	-13.73 (-38.87,11.41)	23.37 (-3.86,50.60)
Change 0-9yr (mu/l/yr or %/yr)	(0.03,0.06)	0.05 (0.03,0.06)	4.08 (-0.12,8.29)	2.82 (-1.64,7.27)	-2.58 (-5.91,0.75)
Change 9-15yr (mu/l/yr or %/yr)	0.14 (0.12,0.16)	0.14 (0.12,0.16)	-5.18 (-10.14, -0.22)	1.94 (-3.48,7.35)	-0.96 (-5.57,3.64)
Change 15-18yr (mu/l/yr or %/yr)	-0.14 (-0.18,-0.10)	-0.12 (-0.16, -0.08)	5.80 (-5.20,16.80)	-8.82 (-18.80,1.16)	-2.06 (-11.52,7.41)
Age 18yr (mu/l or %)	1.76 (1.68,1.85)	1.98 (1.89,2.06)	14.38 (-11.51,40.28)	-5.79 (-29.01,17.42)	-13.54 (-31.07,3.99)
**Adjusted**					
Birth (mu/l or %)	1.05 (0.78,1.32)	1.19 (0.91,1.46)	-7.71 (-31.62,16.20)	-13.80 (-38.45,10.85)	23.58 (-3.05,50.21)
Change 0-9yr (mu/l/yr or %/yr)	0.01 (-0.03,0.06)	0.02 (-0.03,0.06)	3.94 (-0.23,8.10)	2.63 (-1.76,7.02)	-2.66 (-5.93,0.62)
Change 9-15yr (mu/l/yr or %/yr)	0.19 (0.13,0.24)	0.19 (0.13,0.24)	-4.62 (-9.68,0.44)	1.58 (-3.85,7.01)	-0.96 (-5.60,3.67)
Change 15-18yr (mu/l/yr or %/yr)	-0.16 (-0.27,-0.05)	-0.14 (-0.24,-0.03)	4.58 (-6.45,15.62)	-7.53 (-17.55,2.48)	-1.89 (-11.31,7.53)
Age 18yr (mu/l or %)	1.82 (1.59,2.05)	2.04 (1.81,2.27)	12.52 (-13.06,38.11)	-5.44 (-28.32,17.45)	-13.57 (-30.83,3.70)
**Triglyceride**					
**Unadjusted**					
Birth (mmol/l or %)	-0.71 (-0.73,-0.69)	-0.70 (-0.72,-0.68)	-0.25 (-5.56,5.06)	-3.01 (-8.22,2.20)	1.66 (-2.92,6.24)
Change 0-9yr (mmol/l/yr or %/yr)	0.08 (0.08,0.09)	0.09 (0.09,0.09)	0.14 (-0.67,0.95)	0.19 (-0.60,0.98)	-0.35 (-1.04,0.33)
Change 9-18yr (mmol/l/yr or %/yr)	-0.04 (-0.04,-0.04)	-0.04 (-0.05,-0.04)	0.36 (-0.37,1.09)	0.08 (-0.60,0.77)	0.14 (-0.49,0.78)
Age 18yr (mmol/l or %)	-0.33 (-0.35,-0.31)	-0.30 (-0.32,-0.28)	4.34 (-0.82,9.51)	-0.60 (-5.30,4.11)	-0.26 (-4.66,4.13)
**Adjusted**					
Birth (mmol/l or %)	-0.66 (-0.72,-0.60)	-0.65 (-0.71,-0.59)	-1.50 (-6.63,3.63)	-2.86 (-7.96,2.25)	1.15 (-3.35,5.65)
Change 0-9yr (mmol/l/yr or %/yr)	0.08 (0.07,0.09)	0.09 (0.08,0.10)	0.25 (-0.55,1.06)	0.16 (-0.62,0.95)	-0.27 (-0.96,0.41)
Change 9-18yr (mmol/l/yr or %/yr)	-0.04 (-0.05,-0.04)	-0.05 (-0.05,-0.04)	0.28 (-0.45,1.00)	0.02 (-0.67,0.71)	-0.01 (-0.65,0.63)
Age 18yr (mmol/l or %)	-0.32 (-0.37,-0.26)	-0.28 (-0.34,-0.23)	3.29 (-1.83,8.42)	-1.24 (-5.92,3.44)	-1.35 (-5.73,3.04)

CI, confidence interval; mmol/l, millimole per litre; mmol/l/year, millimoles per litre per year; %/yr, percentage per year

[a] Insulin and triglyceride were transformed using the natural log. All predicted mean values and rates of change per year for the reference categories are on the log scale.

[b] The difference between groups is back transformed from the log scale for ease of interpretation and is interpreted as the percentage difference in the mean level in original units comparing each category with the reference or percentage difference in change in original units per year (%/yr) comparing each category with the reference.

For peer review only

Supplemental Table S16 Mean trajectories of HDL-C and non-HDL-c estimated from multilevel models, by maternal anxiety during pregnancy

	Mean trajectory (95% CI) in males (no anxiety during pregnancy) (reference)	Mean trajectory (95% CI) in females (no anxiety during pregnancy) (reference)	Mean difference in trajectory (95% CI) comparing anxiety at 18 weeks only with the reference trajectory	Mean difference in trajectory (95% CI) comparing anxiety at 32 weeks only with the reference trajectory	Mean difference in trajectory (95% CI) c comparing anxiety at 18 and 32 weeks with the reference trajectory
**HDL-c**					
**Unadjusted**					
Birth (mmol/l)	0.50 (0.49,0.51)	0.55 (0.53,0.56)	0.01 (-0.02,0.04)	0.01 (-0.02,0.04)	-0.003 (-0.03,0.02)
Change 0-7yr (mmol/l/yr)	0.15 (0.15,0.15)	0.13 (0.13,0.13)	-0.005 (-0.01,0.002)	0.0004 (-0.01,0.01)	-0.001 (-0.01,0.004)
Change 7-18yr (mmol/l/yr)	-0.04 (-0.04,-0.03)	-0.01 (-0.01,-0.01)	0.0003 (-0.003,0.004)	-0.002 (-0.01,0.001)	-0.00003 (-0.003,0.003)
Age 18yr (mmol/l)	1.15 (1.14,1.17)	1.33 (1.32,1.35)	-0.02 (-0.05,0.02)	-0.01 (-0.05,0.02)	-0.01 (-0.04,0.02)
**Adjusted**					
Birth (mmol/l)	0.49 (0.46,0.53)	0.54 (0.50,0.57)	0.02 (-0.02,0.05)	0.01 (-0.02,0.04)	0.003 (-0.02,0.03)
Change 0-7yr (mmol/l/yr)	0.15 (0.14,0.16)	0.13 (0.13,0.14)	-0.005 (-0.01,0.001)	0.001 (-0.01,0.01)	-0.002 (-0.01,0.003)
Change 7-18yr (mmol/l/yr)	-0.04 (-0.04,-0.03)	-0.01 (-0.01,-0.01)	0.001 (-0.003,0.004)	-0.001 (-0.005,0.002)	0.001 (-0.002,0.004)
Age 18yr (mmol/l)	1.16 (1.13,1.20)	1.35 (1.31,1.39)	-0.01 (-0.05,0.03)	-0.005 (-0.04,0.03)	0.0005 (-0.03,0.03)
**Non-HDL-c**					
**Unadjusted**					
Birth (mmol/l)	1.23 (1.20,1.26)	1.30 (1.27,1.33)	-0.08 (-0.18,0.01)	0.03 (-0.08,0.13)	-0.02 (-0.10,0.06)
Change 0-9yr (mmol/l/yr)	0.19 (0.18,0.19)	0.21 (0.20,0.21)	0.017 (0.001,0.03)	-0.005 (-0.02,0.01)	-0.002 (-0.01,0.01)
Change 9-18yr (mmol/l/yr)	-0.07 (-0.08,-0.07)	-0.07 (-0.08,-0.07)	0.004 (-0.01,0.02)	0.01 (-0.01,0.02)	0.01 (-0.01,0.02)
Age 18yr (mmol/l)	2.27 (2.23,2.30)	2.49 (2.45,2.52)	0.11 (-0.01,0.22)	0.04 (-0.07,0.15)	0.02 (-0.08,0.12)
**Adjusted**					
Birth (mmol/l)	1.21 (1.13,1.29)	1.27 (1.19,1.35)	-0.09 (-0.19,0.01)	0.03 (-0.08,0.13)	-0.02 (-0.10,0.06)
Change 0-9yr (mmol/l/yr)	0.19 (0.18,0.20)	0.21 (0.20,0.22)	0.02 (0.002,0.03)	-0.004 (-0.02,0.01)	-0.003 (-0.02,0.01)
Change 9-18yr (mmol/l/yr)	-0.07 (-0.08,-0.06)	-0.07 (-0.08,-0.06)	0.002 (-0.01,0.02)	0.01 (-0.01,0.02)	0.005 (-0.01,0.02)
Age 18yr (mmol/l)	2.27 (2.18,2.36)	2.49 (2.40,2.58)	0.09 (-0.02,0.20)	0.04 (-0.07,0.15)	0.001 (-0.10,0.10)

CI, confidence interval; mmol/l, millimole per litre; mmol/l, millimole per litre per year.

For peer review only

Supplemental Table S17 Mean trajectories of BMI estimated from multilevel models, by maternal depression during pregnancy

	Mean trajectory (95% CI) in males (no depression during pregnancy) log (kg/m^2) (reference) [a]	Mean trajectory (95% CI) in females (no depression during pregnancy) log (kg/m^2) (reference) [a]	Mean difference in trajectory (95% CI) comparing depression at 18 weeks only with the reference trajectory (% difference in kg/m^2) [b]	Mean difference in trajectory (95% CI) depression at 32 weeks only with the reference trajectory (% difference in kg/m^2) [b]	Mean difference in trajectory (95% CI) comparing depression at 18 and 32 weeks with the reference trajectory (% difference in kg/m^2) [b]
**Unadjusted BMI**					
Age 1yr	2.90 (2.89,2.91)	2.89 (2.88,2.89)	0.07 (-1.60,1.75)	-0.33 (-1.79,1.13)	0.3 (-1.32,1.91)
Age 3yr	2.77 (2.76,2.77)	2.75 (2.75,2.76)	0.13 (-0.72,0.98)	0.39 (-0.36,1.15)	-0.5 (-1.34,0.26)
Age 7yr	2.79 (2.78,2.79)	2.80 (2.79,2.80)	0.51 (-0.61,1.62)	0.61 (-0.38,1.59)	0.1 (-0.97,1.11)
Age 9yr	2.82 (2.82,2.83)	2.84 (2.84,2.85)	0.70 (-0.67,2.07)	0.71 (-0.50,1.92)	0.5 (-0.77,1.80)
Age 11yr	2.88 (2.87,2.88)	2.90 (2.90,2.91)	0.88 (-0.69,2.44)	0.85 (-0.53,2.23)	1.0 (-0.48,2.46)
Age 13yr	2.94 (2.94,2.95)	2.97 (2.96,2.98)	1.04 (-0.64,2.72)	1.02 (-0.46,2.50)	1.5 (-0.10,3.07)
Age 15yr	3.02 (3.01,3.02)	3.04 (3.04,3.05)	1.19 (-0.58,2.95)	1.24 (-0.31,2.80)	2.0 (0.31,3.66)
Age 18yr	3.16 (3.15,3.16)	3.17 (3.16,3.18)	1.37 (-0.77,3.50)	1.67 (-0.21,3.55)	2.7 (0.71,4.78)
**Adjusted BMI**					
Age 1yr	2.88 (2.87,2.90)	2.87 (2.86,2.89)	0.04 (-1.66,1.73)	-0.37 (-1.83,1.10)	0.2 (-1.45,1.82)
Age 3yr	2.77 (2.76,2.78)	2.75 (2.74,2.76)	-0.19 (-1.04,0.66)	0.24 (-0.52,0.99)	-0.8 (-1.63,-0.04)
Age 7yr	2.79 (2.78,2.80)	2.80 (2.79,2.81)	-0.15 (-1.22,0.92)	0.29 (-0.66,1.23)	-0.5 (-1.49,0.52)
Age 9yr	2.82 (2.81,2.83)	2.84 (2.83,2.86)	-0.14 (-1.44,1.16)	0.28 (-0.87,1.43)	-0.2 (-1.44,1.00)
Age 11yr	2.87 (2.86,2.89)	2.90 (2.89,2.92)	-0.15 (-1.62,1.32)	0.27 (-1.03,1.57)	0.0 (-1.35,1.43)
Age 13yr	2.94 (2.92,2.95)	2.97 (2.96,2.98)	-0.18 (-1.75,1.39)	0.28 (-1.11,1.66)	0.3 (-1.21,1.76)
Age 15yr	3.02 (3.00,3.03)	3.05 (3.03,3.06)	-0.24 (-1.88,1.40)	0.30 (-1.16,1.75)	0.5 (-1.09,2.03)
Age 18yr	3.16 (3.14,3.18)	3.17 (3.16,3.19)	-0.38 (-2.39,1.63)	0.37 (-1.41,2.15)	0.7 (-1.23,2.61)

CI, confidence interval; yr, year.

[a] BMI is presented in the natural log and values represent the mean predicted natural log of BMI at each age shown.

[b] Differences at each age are back transformed from the log scale and are interpreted as the percentage difference in the mean level in original units at each age comparing each category with the reference trajectory.

For peer review only - http://bmjopen.bmj.com/site/about/guidelines.xhtml

Supplemental Table S18 Mean trajectories of fat mass estimated from multilevel models, by maternal depression during pregnancy

	Mean trajectory (95% CI) in males (no depression during pregnancy) log (kg or kg/yr) (reference) [a]	Mean trajectory (95% CI) in females (no depression during pregnancy) log (kg or kg/yr) (reference) [a]	Mean difference in trajectory (95% CI) comparing depression at 18 weeks only with the reference trajectory (% or %/yr) [b]	Mean difference in trajectory (95% CI) depression at 32 weeks only with the reference trajectory (% or %/yr) [b]	Mean difference in trajectory (95% CI) comparing depression at 18 and 32 weeks with the reference trajectory (% or %/yr) [b]
**Unadjusted**					
Age 9yr (kg) or (%)	1.79 (1.76,1.82)	2.64 (2.12,3.16)	2.03 (-5.19,9.26)	-3.60 (-9.47,2.28)	1.61 (-5.11,8.33)
Change 9-13yr (kg/yr) or (%/yr)	0.12 (0.11,0.12)	0.16 (0.15,0.17)	0.90 (-0.60,2.41)	0.77 (-0.52,2.06)	1.86 (0.40,3.32)
Change 13-15yr (kg/yr) or (%/yr)	-0.06 (-0.07,-0.05)	0.10 (0.09,0.11)	-2.44 (-5.07,0.20)	0.22 (-2.08,2.52)	-0.61 (-3.24,2.03)
Change 15-18yr (kg/yr) or (%/yr)	0.10 (0.09,0.10)	0.06 (0.05,0.06)	1.23 (-0.83,3.28)	-0.28 (-2.03,1.48)	-0.44 (-2.43,1.54)
Age 18yr (kg) or (%)	2.43 (2.41,2.46)	3.65 (3.10,4.20)	4.43 (-3.14,12.01)	-0.98 (-7.26,5.30)	6.61 (-0.74,13.96)
**Adjusted**					
Age 9yr (kg) or (%)	1.81 (1.74,1.87)	2.67 (2.14,3.19)	-0.13 (-7.03,6.77)	-3.49 (-9.23,2.25)	-0.21 (-6.66,6.24)
Change 9-13yr (kg/yr) or (%/yr)	0.11 (0.09,0.12)	0.15 (0.14,0.17)	0.56 (-0.94,2.06)	0.37 (-0.92,1.66)	1.33 (-0.12,2.79)
Change 13-15yr (kg/yr) or (%/yr)	-0.07 (-0.09,-0.04)	0.09 (0.07,0.12)	-2.41 (-5.05,0.24)	-0.02 (-2.32,2.29)	-0.78 (-3.42,1.87)
Change 15-18yr (kg/yr) or (%/yr)	0.10 (0.09,0.12)	0.06 (0.04,0.08)	1.25 (-0.82,3.32)	-0.20 (-1.97,1.56)	-0.41 (-2.41,1.59)
Age 18yr (kg) or (%)	2.42 (2.36,2.48)	3.64 (3.09,4.19)	0.96 (-6.17,8.09)	-2.67 (-8.69,3.34)	2.32 (-4.58,9.22)

CI, confidence interval; kg/yr, kilograms per year; %/yr, percentage per year.
[a] Fat mass was transformed using the natural log. All predicted mean values (kg) and rates of change per year (kg/yr) for the reference categories are on the log scale
[b] The difference between groups is back transformed from the log scale for ease of interpretation and is interpreted as the percentage difference in the mean level in original units comparing each category with the reference or percentage difference in change in original units per year (%/yr) comparing each category with the reference.

For peer review only

Supplemental Table S19 Mean trajectories of blood pressure and pulse rate estimated from multilevel models, by maternal depression during pregnancy

	Mean trajectory (95% CI) in males (no depression during pregnancy) (reference)	Mean trajectory (95% CI) in females (no depression during pregnancy) (reference)	Mean difference in trajectory (95% CI) comparing depression at 18 weeks only with the reference trajectory	Mean difference in trajectory (95% CI) comparing depression at 32 weeks only with the reference trajectory	Mean difference in trajectory (95% CI) comparing depression at 18 and 32 weeks with the reference trajectory
**SBP**					
**Unadjusted**					
Age 7yr (mmHg)	97.85 (97.49,98.20)	97.95 (97.59,98.31)	-0.18 (-1.31,0.95)	0.33 (-0.64,1.30)	0.30 (-0.75,1.35)
Change 7-12yr (mmHg/yr)	1.66 (1.57,1.74)	1.87 (1.79,1.96)	0.17 (-0.10,0.44)	-0.13 (-0.36,0.10)	-0.14 (-0.39,0.11)
Change 12-16yr (mmHg/yr)	5.82 (5.67,5.97)	3.81 (3.67,3.96)	0.11 (-0.35,0.58)	0.17 (-0.23,0.56)	0.38 (-0.05,0.82)
Change 16-18yr (mmHg/yr)	-3.98 (-4.32,-3.65)	-5.86 (-6.18,-5.54)	0.48 (-0.55,1.52)	-0.64 (-1.53,0.24)	-0.07 (-1.04,0.91)
Age 18yr (mmHg)	121.44 (120.97,121.91)	110.85 (110.42,111.28)	2.11 (0.67,3.55)	-0.93 (-2.17,0.31)	1.00 (-0.36,2.36)
**Adjusted**					
Age 7yr (mmHg)	98.97 (97.99,99.96)	99.11 (98.11,100.10)	-0.46 (-1.59,0.66)	0.16 (-0.80,1.13)	0.02 (-1.03,1.07)
Change 7-12yr (mmHg/yr)	1.48 (1.24,1.71)	1.70 (1.46,1.94)	0.18 (-0.09,0.45)	-0.12 (-0.35,0.11)	-0.14 (-0.39,0.11)
Change 12-16yr (mmHg/yr)	5.95 (5.55,6.35)	3.94 (3.53,4.35)	0.18 (-0.28,0.65)	0.23 (-0.17,0.63)	0.44 (-0.002,0.87)
Change 16-18yr (mmHg/yr)	-4.20 (-5.11,-3.29)	-6.11 (-7.02,-5.19)	0.23 (-0.80,1.27)	-0.88 (-1.77,0.01)	-0.28 (-1.26,0.70)
Age 18yr (mmHg)	121.76 (120.49,123.03)	111.15 (109.88,112.42)	1.62 (0.17,3.07)	-1.29 (-2.54,-0.05)	0.50 (-0.87,1.87)
**DBP**					
**Unadjusted**					
Age 7yr (mmHg)	56.11 (55.86,56.36)	56.96 (56.70,57.22)	0.24 (-0.58,1.06)	0.44 (-0.26,1.14)	0.31 (-0.45,1.07)
Change 7-12yr (mmHg/yr)	0.14 (0.07,0.21)	0.12 (0.05,0.19)	0.04 (-0.17,0.25)	0.03 (-0.15,0.21)	-0.07 (-0.27,0.12)
Change 12-16yr (mmHg/yr)	2.85 (2.73,2.98)	2.33 (2.20,2.45)	0.06 (-0.34,0.45)	-0.12 (-0.46,0.22)	-0.10 (-0.47,0.27)
Change 16-18yr (mmHg/yr)	-2.55 (-2.83,-2.26)	-1.12 (-1.39,-0.85)	0.17 (-0.69,1.03)	0.02 (-0.72,0.77)	0.70 (-0.12,1.51)
Age 18yr (mmHg)	63.14 (62.80,63.48)	63.76 (62.96,64.56)	0.99 (-0.03,2.02)	0.18 (-0.72,1.07)	0.95 (-0.02,1.91)
**Adjusted**					
Age 7yr (mmHg)	56.83 (56.11,57.54)	57.69 (56.97,58.41)	0.14 (-0.68,0.96)	0.37 (-0.33,1.07)	0.20 (-0.56,0.96)
Change 7-12yr (mmHg/yr)	0.05 (-0.13,0.23)	0.04 (-0.15,0.22)	0.03 (-0.18,0.24)	0.03 (-0.14,0.21)	-0.08 (-0.27,0.12)
Change 12-16yr (mmHg/yr)	2.94 (2.60,3.29)	2.42 (2.07,2.77)	0.15 (-0.24,0.55)	-0.02 (-0.36,0.32)	0.02 (-0.36,0.40)
Change 16-18yr (mmHg/yr)	-2.70 (-3.48,-1.92)	-1.31 (-2.08,-0.53)	-0.09 (-0.96,0.78)	-0.27 (-1.02,0.48)	0.34 (-0.48,1.17)
Age 18yr (mmHg)	63.46 (62.54,64.39)	64.13 (62.95,65.30)	0.73 (-0.30,1.77)	-0.07 (-0.96,0.83)	0.59 (-0.38,1.56)
**Pulse rate**					
**Unadjusted**					
Age 7yr (bpm)	82.49 (82.08,82.90)	85.76 (85.34,86.18)	0.36 (-0.96,1.68)	0.51 (-0.62,1.64)	0.07 (-1.16,1.29)
Change 7-12yr (bpm/yr)	-1.92 (-2.02,-1.82)	-1.76 (-1.86,-1.66)	-0.14 (-0.46,0.18)	0.03 (-0.24,0.30)	0.08 (-0.22,0.38)
Change 12-16yr (bpm/yr)	-0.73 (-0.88,-0.57)	-0.28 (-0.43,-0.12)	0.43 (-0.05,0.92)	0.24 (-0.17,0.65)	0.29 (-0.17,0.74)
Change 16-18yr (bpm/yr)	-4.08 (-4.43,-3.73)	-4.69 (-5.02,-4.36)	-0.65 (-1.72,0.41)	-0.83 (-1.74,0.08)	-0.19 (-1.19,0.81)
Age 18yr (bpm)	61.82 (61.31,62.33)	66.46 (65.99,66.93)	0.07 (-1.49,1.63)	-0.04 (-1.38,1.31)	1.22 (-0.25,2.68)
**Adjusted**					
Age 7yr (bpm)	82.71 (81.56,83.86)	85.94 (84.77,87.11)	0.24 (-1.09,1.56)	0.51 (-0.62,1.64)	0.05 (-1.19,1.28)
Change 7-12yr (bpm/yr)	-1.93 (-2.21,-1.66)	-1.76 (-2.04,-1.48)	-0.11 (-0.43,0.21)	0.04 (-0.23,0.31)	0.08 (-0.22,0.38)
Change 12-16yr (bpm/yr)	-1.05 (-1.47,-0.63)	-0.60 (-1.03,-0.17)	0.42 (-0.07,0.91)	0.21 (-0.21,0.62)	0.26 (-0.19,0.72)
Change 16-18yr (bpm/yr)	-3.61 (-4.56,-2.67)	-4.27 (-5.21,-3.33)	-0.74 (-1.80,0.33)	-0.91 (-1.82,0.01)	-0.29 (-1.29,0.71)
Age 18yr (bpm)	61.63 (60.25,63.01)	66.17 (64.79,67.55)	-0.12 (-1.68,1.44)	-0.26 (-1.61,1.09)	0.93 (-0.54,2.40)

bpm, beats per minute; bpm/yr, beats per minute per year; CI, confidence interval; mmHg, millimetres of mercury; mmHg/yr, millimetres of mercury per year.

For peer review only - http://bmjopen.bmj.com/site/about/guidelines.xhtml

Supplemental Table S20 Mean trajectories of lean mass estimated from multilevel models, by maternal depression during pregnancy

	Mean trajectory (95% CI) in males (no depression during pregnancy) (reference)	Mean trajectory (95% CI) in females (no depression during pregnancy) (reference)	Mean difference in trajectory (95% CI) comparing depression at 18 weeks only with the reference trajectory	Mean difference in trajectory (95% CI) comparing depression at 32 weeks only with the reference trajectory	Mean difference in trajectory (95% CI) comparing depression at 18 and 32 weeks with the reference trajectory
**Unadjusted**					
Age 9yr (kg)	23.89 (23.76,24.02)	20.77 (20.65,20.90)	-0.10 (-0.47,0.26)	-0.39 (-0.70,-0.07)	-0.18 (-0.53,0.17)
Change 9-13yr (kg/yr)	2.31 (2.25,2.37)	3.23 (3.17,3.28)	0.03 (-0.13,0.18)	-0.01 (-0.14,0.13)	-0.03 (-0.17,0.12)
Change 13-15yr (kg/yr)	7.68 (7.59,7.78)	1.57 (1.46,1.68)	-0.05 (-0.35,0.25)	-0.02 (-0.28,0.23)	-0.04 (-0.33,0.26)
Change 15-18yr (kg/yr)	2.47 (2.40,2.55)	0.38 (0.31,0.44)	-0.01 (-0.23,0.21)	0.002 (-0.19,0.19)	0.0005 (-0.21,0.22)
Age 18yr (kg)	55.91 (55.68,56.14)	37.95 (37.72,38.18)	-0.13 (-0.85,0.58)	-0.46 (-1.09,0.17)	-0.35 (-1.03,0.33)
**Adjusted**					
Age 9yr (kg)	23.83 (23.51,24.16)	20.74 (20.42,21.07)	-0.06 (-0.43,0.30)	-0.33 (-0.65,-0.02)	-0.16 (-0.51,0.18)
Change 9-13yr (kg/yr)	2.30 (2.16,2.44)	3.22 (3.08,3.36)	0.0001 (-0.16,0.16)	-0.01 (-0.14,0.13)	-0.04 (-0.18,0.11)
Change 13-15yr (kg/yr)	7.47 (7.21,7.73)	1.35 (1.08,1.62)	-0.03 (-0.33,0.27)	-0.03 (-0.29,0.23)	-0.02 (-0.32,0.27)
Change 15-18yr (kg/yr)	2.64 (2.45,2.83)	0.55 (0.36,0.74)	0.002 (-0.22,0.22)	0.01 (-0.18,0.20)	0.01 (-0.21,0.22)
Age 18yr (kg)	55.90 (55.27,56.53)	37.98 (37.34,38.61)	-0.11 (-0.83,0.60)	-0.40 (-1.02,0.23)	-0.33 (-1.02,0.35)

kg/yr, kilograms per year.

For peer review only - http://bmjopen.bmj.com/site/about/guidelines.xhtml

Supplemental Table S21 Mean trajectories of glucose estimated from multilevel models, by maternal depression during pregnancy

	Mean trajectory (95% CI) in males (no depression during pregnancy) (reference)	Mean trajectory (95% CI) in females (no depression during pregnancy) (reference)	Mean difference in trajectory (95% CI) comparing depression at 18 weeks only with the reference trajectory	Mean difference in trajectory (95% CI) comparing depression at 32 weeks only with the reference trajectory	Mean difference in trajectory (95% CI) c comparing depression at 18 and 32 weeks with the reference trajectory
**Unadjusted**					
Age 7yr (mmol/l)	4.20 (4.17,4.22)	4.13 (4.11,4.15)	-0.04 (-0.12,0.04)	-0.05 (-0.11,0.02)	-0.04 (-0.11,0.04)
Change 7-15yr (mmol/l/yr)	0.14 (0.14,0.15)	0.13 (0.13,0.14)	0.001 (-0.01,0.01)	0.002 (-0.01,0.01)	0.01 (-0.002,0.03)
Change 15-18yr (mmol/l/yr)	-0.08 (-0.09,-0.07)	-0.10 (-0.11,-0.09)	0.01 (-0.03,0.05)	- 0.003 (-0.04,0.03)	-0.01 (-0.05,0.03)
Age 18yr (mmol/l)	5.11 (5.09,5.14)	4.89 (4.87,4.92)	-0.01 (-0.08,0.07)	-0.03 (-0.10,0.04)	0.02 (-0.06,0.10)
**Adjusted**					
Age 7yr (mmol/l)	4.21 (4.14,4.28)	4.14 (4.08,4.21)	-0.04 (-0.12,0.03)	-0.04 (-0.11,0.02)	-0.03 (-0.10,0.04)
Change 7-15yr (mmol/l/yr)	0.14 (0.13,0.16)	0.13 (0.12,0.14)	0.001 (-0.01,0.01)	0.002 (-0.01,0.01)	0.01 (-0.002,0.03)
Change 15-18yr (mmol/l/yr)	-0.08 (-0.11,-0.04)	-0.10 (-0.13,-0.06)	0.01 (-0.03,0.05)	-0.001 (-0.04,0.03)	-0.01 (-0.05,0.03)
Age 18yr (mmol/l)	5.13 (5.06,5.20)	4.91 (4.84,4.98)	-0.01 (-0.09,0.06)	-0.03 (-0.10,0.04)	0.02 (-0.06,0.10)

CI, confidence interval; mmol/l, millimole per litre; mmol/l/year, millimoles per litre per year.

For peer review only

Supplemental Table S22 Mean trajectories of insulin and triglyceride estimated from multilevel models, by maternal depression during pregnancy

	Mean trajectory (95% CI) in males (no depression during pregnancy) (reference) [a]	Mean trajectory (95% CI) in females (no depression during pregnancy) (reference) [a]	Mean difference in trajectory (95% CI) comparing depression at 18 weeks only with the reference trajectory (% or %/yr) [b]	Mean difference in trajecto (95% CI) comparing depression at 32 weeks onl with the reference trajector (% or %/yr) [b]	Mean difference in trajectory (95% CI) comparing depression at 18 and 32 weeks with the reference trajectory (% or %/yr) [b]
**Insulin**					
**Unadjusted**					
Birth (mu/l or %)	1.00 (0.90,1.10)	1.08 (0.99,1.18)	-8.47 (-47.50,30.55)	6.35 (-28.02,40.73)	4.26 (-29.26,37.78)
Change 0-9yr (mu/l/yr or %/yr)	0.04 (0.03,0.06)	0.05 (0.04,0.07)	2.05 (-3.91,8.00)	-0.56 (-5.32,4.20)	-2.07 (-6.87,2.73)
Change 9-15yr (mu/l/yr or %/yr)	0.13 (0.11,0.15)	0.14 (0.12,0.16)	-2.14 (-8.16,3.88)	-3.96 (-9.81,1.89)	3.18 (-3.53,9.89)
Change 15-18yr (mu/l/yr or %/yr)	-0.15 (-0.19,-0.11)	-0.13 (-0.17,-0.09)	5.89 (-7.22,19.00)	7.42 (-5.61,20.46)	-7.27 (-19.75,5.21)
Age 18yr (mu/l or %)	1.75 (1.67,1.84)	1.98 (1.89,2.06)	14.50 (-17.62,46.62)	-1.65 (-26.79,23.49)	-16.92 (-40.13,6.29)
**Adjusted**					
Birth (mu/l or %)	1.05 (0.77,1.32)	1.17 (0.89,1.44)	-8.23 (-47.25,30.78)	4.98 (-28.32,38.28)	14.00 (-22.85,50.85)
Change 0-9yr (mu/l/yr or %/yr)	0.02 (-0.02,0.06)	0.02 (-0.02,0.07)	1.86 (-4.02,7.74)	-0.61 (-5.27,4.06)	-3.06 (-7.79,1.67)
Change 9-15yr (mu/l/yr or %/yr)	0.18 (0.13,0.24)	0.18 (0.13,0.24)	-1.40 (-7.48,4.68)	-3.97 (-9.85,1.91)	2.66 (-3.99,9.30)
Change 15-18yr (mu/l/yr or %/yr)	-0.16 (-0.27,-0.05)	-0.14 (-0.25,-0.03)	5.68 (-7.34,18.69)	9.24 (-4.01,22.49)	-5.79 (-18.31,6.73)
Age 18yr (mu/l or %)	1.82 (1.59,2.05)	2.05 (1.82,2.28)	17.45 (-15.03,49.92)	1.61 (-24.14,27.37)	-15.65 (-38.77,7.47)
**Triglyceride**					
**Unadjusted**					
Birth (mmol/l or %)	-0.71 (-0.73,-0.69)	-0.70 (-0.72,-0.68)	1.36 (-4.96,7.68)	0.21 (-5.58,6.00)	-0.49 (-6.46,5.49)
Change 0-9yr (mmol/l/yr or %/yr)	0.08 (0.08,0.09)	0.09 (0.09,0.09)	0.06 (-0.90,1.03)	-0.09 (-0.95,0.77)	-0.23 (-1.15,0.68)
Change 9-18yr (mmol/l/yr or %/yr)	-0.04 (-0.04,-0.04)	-0.04 (-0.05,-0.04)	-0.46 (-1.33,0.41)	-0.35 (-1.11,0.42)	-0.22 (-1.07,0.63)
Age 18yr (mmol/l or %)	-0.33 (-0.34,-0.31)	-0.29 (-0.31,-0.27)	-2.20 (-7.98,3.59)	-3.67 (-8.77,1.43)	-4.44 (-10.11,1.23)
**Adjusted**					
Birth (mmol/l or %)	-0.66 (-0.72,-0.60)	-0.66 (-0.72,-0.60)	-0.67 (-6.76,5.42)	0.90 (-4.82,6.62)	-0.63 (-6.50,5.24)
Change 0-9yr (mmol/l/yr or %/yr)	0.08 (0.07,0.09)	0.09 (0.08,0.10)	0.24 (-0.72,1.20)	-0.14 (-0.99,0.72)	-0.18 (-1.09,0.73)
Change 9-18yr (mmol/l/yr or %/yr)	-0.04 (-0.05,-0.03)	-0.05 (-0.05,-0.04)	-0.61 (-1.48,0.26)	-0.46 (-1.22,0.31)	-0.41 (-1.26,0.44)
Age 18yr (mmol/l or %)	-0.31 (-0.37,-0.26)	-0.28 (-0.33,-0.22)	-3.93 (-9.63,1.77)	-4.38 (-9.45,0.70)	-5.77 (-11.39,-0.14)

CI, confidence interval; mmol/l, millimole per litre; mmol/l/year, millimoles per litre per year; %/yr, percentage per year

[a] Insulin and triglyceride were transformed using the natural log. All predicted mean values (mmol/l) and rates of change per year (mmol/l/yr) for the reference categories are on the log scale.

[b] The difference between groups is back transformed from the log scale for ease of interpretation and is interpreted as the percentage difference in the mean level in original units comparing each category with the reference or percentage difference in change in original units per year (%/yr) comparing each category with the reference.

For peer review only

Supplemental Table S23 Mean trajectories of HDL-C and non-HDL-c estimated from multilevel models, by maternal depression during pregnancy

	Mean trajectory (95% CI) in males (no depression during pregnancy) (reference)	Mean trajectory (95% CI) in females (no depression during pregnancy) (reference)	Mean difference in trajectory (95% CI) comparing depression at 18 weeks only with the reference trajectory	Mean difference in trajectory (95% CI) comparing depression at 32 weeks only with the reference trajectory	Mean difference in trajectory (95% CI) comparing depression at 18 and 32 weeks with the reference trajectory
**HDL-c**					
**Unadjusted**					
Birth (mmol/l)	0.50 (0.49,0.52)	0.55 (0.54,0.56)	-0.02 (-0.05,0.02)	-0.04 (-0.07,-0.01)	-0.001 (-0.04,0.03)
Change 0-7yr (mmol/l/yr)	0.15 (0.15,0.15)	0.13 (0.13,0.13)	0.002 (-0.01,0.01)	0.01 (0.001,0.01)	0.0001 (-0.01,0.01)
Change 7-18yr (mmol/l/yr)	-0.04 (-0.04,-0.03)	-0.01 (-0.01,-0.01)	0.001 (-0.003,0.01)	-0.005 (-0.01,-0.001)	-0.001 (-0.01,0.003)
Age 18yr (mmol/l)	1.15 (1.14,1.16)	1.33 (1.32,1.35)	0.01 (-0.03,0.05)	-0.03 (-0.07,0.003)	-0.02 (-0.06,0.03)
**Adjusted**					
Birth (mmol/l)	0.49 (0.46,0.53)	0.54 (0.51,0.58)	-0.01 (-0.04,0.03)	-0.04 (-0.07,-0.01)	0.004 (-0.03,0.04)
Change 0-7yr (mmol/l/yr)	0.15 (0.14,0.16)	0.13 (0.13,0.14)	0.001 (-0.01,0.01)	0.01 (0.001,0.01)	-0.001 (-0.01,0.01)
Change 7-18yr (mmol/l/yr)	-0.04 (-0.04,-0.03)	-0.01 (-0.01,-0.01)	0.002 (-0.002,0.01)	-0.004 (-0.01,0.0002)	-0.00004 (-0.004,0.004)
Age 18yr (mmol/l)	1.01 (0.98,1.05))	1.35 (1.31,1.39)	0.03 (-0.02,0.07)	-0.02 (-0.06,0.01)	-0.001 (-0.04,0.04)
**Non-HDL-c**					
**Unadjusted**					
Birth (mmol/l)	1.22 (1.20,1.25)	1.30 (1.27,1.33)	-0.06 (-0.17,0.05)	0.07 (-0.03,0.17)	-0.04 (-0.15,0.07)
Change 0-9yr (mmol/l/yr)	0.19 (0.19,0.19)	0.20 (0.20,0.21)	0.01 (-0.01,0.03)	-0.01 (-0.03,0.00)	0.005 (-0.01,0.02)
Change 9-18yr (mmol/l/yr)	-0.07 (-0.08,-0.07)	-0.07 (-0.08,-0.07)	0.01 (-0.01,0.03)	-0.002 (-0.02,0.01)	0.005 (-0.01,0.02)
Age 18yr (mmol/l)	2.28 (2.24,2.31)	2.48 (2.45,2.51)	0.11 (-0.03,0.24)	-0.07 (-0.19,0.06)	0.05 (-0.09,0.19)
**Adjusted**					
Birth (mmol/l)	1.20 (1.12,1.28)	1.27 (1.19,1.35)	-0.08 (-0.19,0.04)	0.07 (-0.03,0.18)	-0.03 (-0.15,0.08)
Change 0-9yr (mmol/l/yr)	0.19 (0.18,0.20)	0.21 (0.20,0.22)	0.01 (-0.01,0.03)	-0.01 (-0.03,0.00)	0.004 (-0.01,0.02)
Change 9-18yr (mmol/l/yr)	-0.07 (-0.08,-0.06)	-0.07 (-0.08,-0.06)	0.01 (-0.01,0.02)	-0.003 (-0.02,0.01)	0.004 (-0.01,0.02)
Age 18yr (mmol/l)	2.28 (2.19,2.36)	2.48 (2.39,2.57)	0.09 (-0.05,0.22)	-0.08 (-0.20,0.04)	0.04 (-0.10,0.18)

CI, confidence interval; mmol/l, millimole per litre; mmol/l, millimole per litre per year.

For peer review only

Supplemental Figure S1 Flow diagram of study design

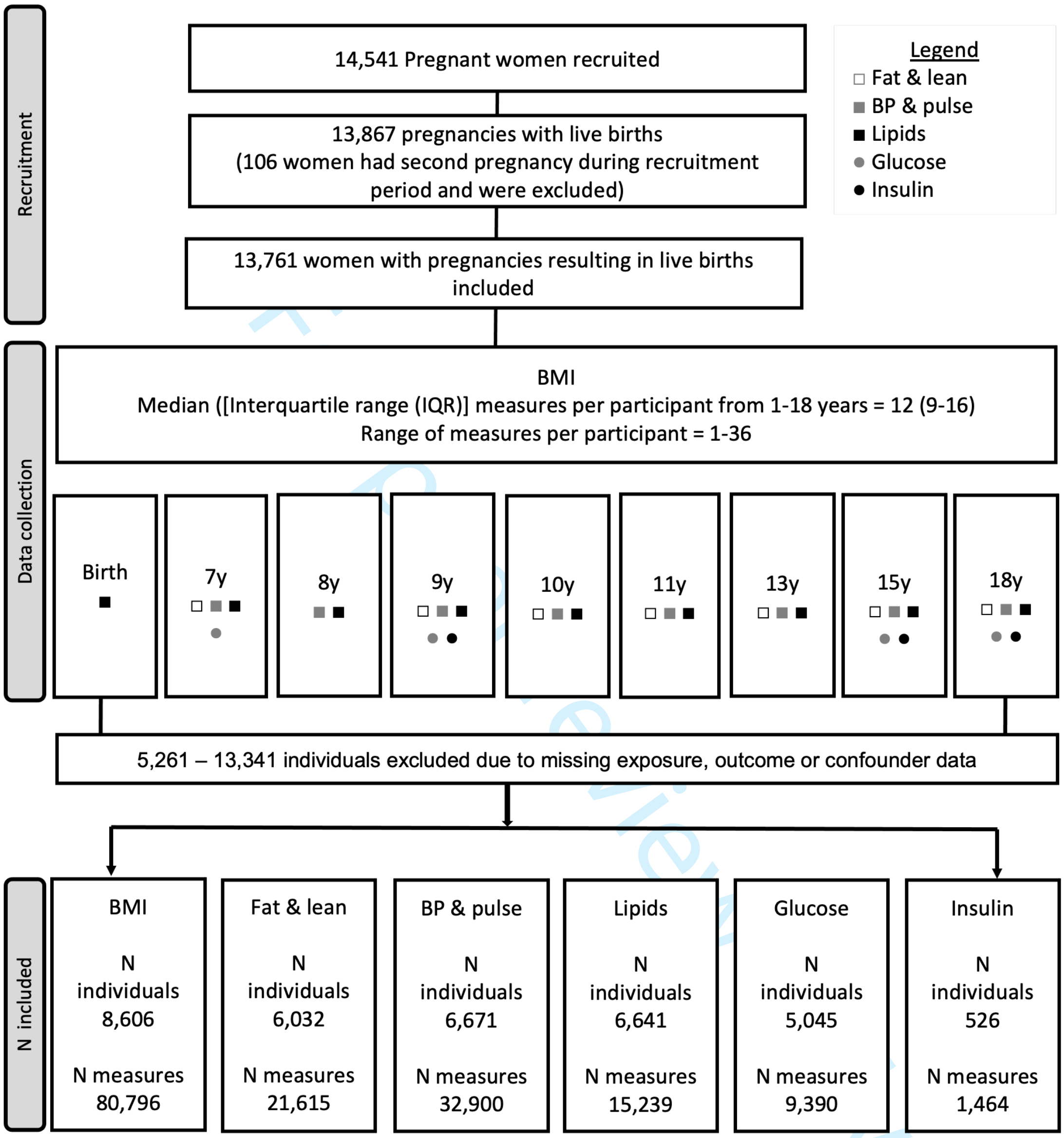

Legend: BP, blood pressure; BMI, body mass index; y, years of age

For peer review only - http://bmjopen.bmj.com/site/about/guidelines.xhtml

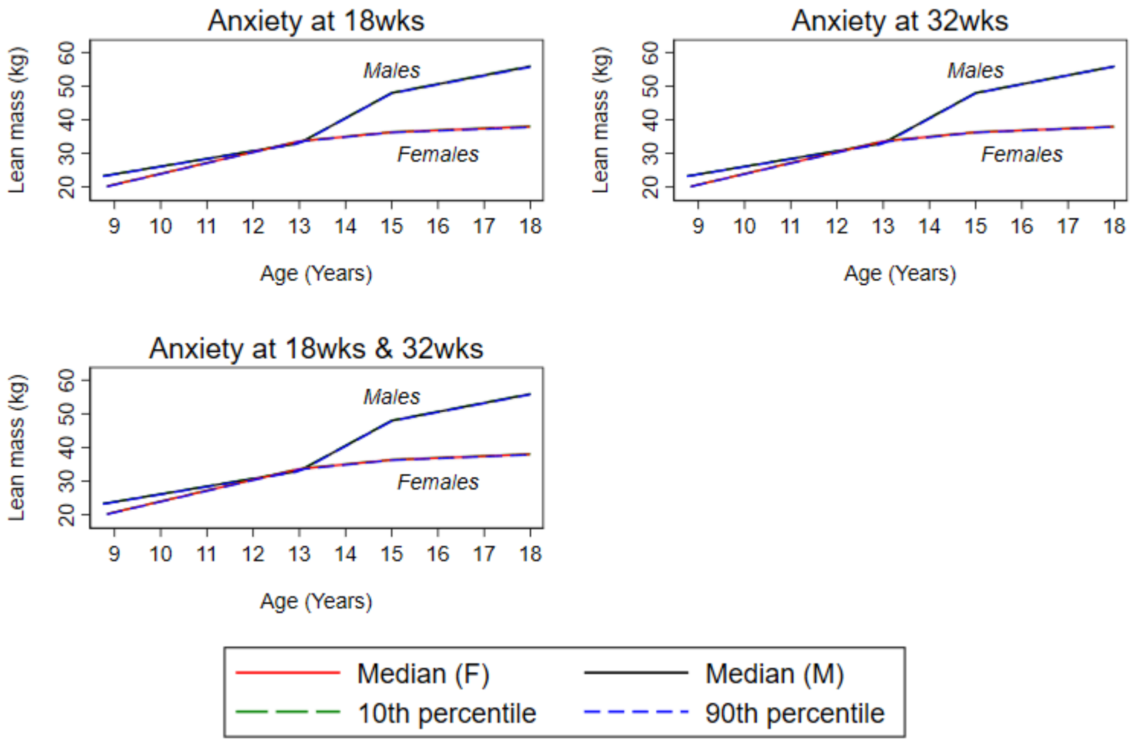

Supplemental Figure S2 Mean predicted confounder adjusted trajectories of lean mass (9 to 18 years), by continuous maternal anxiety levels during pregnancy

Legend: Trajectories are adjusted for maternal age at birth of offspring, parental household socioeconomic position, parity, maternal pre-pregnancy BMI and maternal smoking during pregnancy. Median, 10th and 90th percentile = 1, 4 and 9 at 18 weeks. Median, 10th and 90th percentile = 1, 4, 9 at 32 weeks. Mean of the 18 and 32 week measures used for analyses of anxiety at 18 weeks and 32 weeks; median, 10th and 90th percentile = 1, 4, 9 in this analysis.

For peer review only - http://bmjopen.bmj.com/site/about/guidelines.xhtml

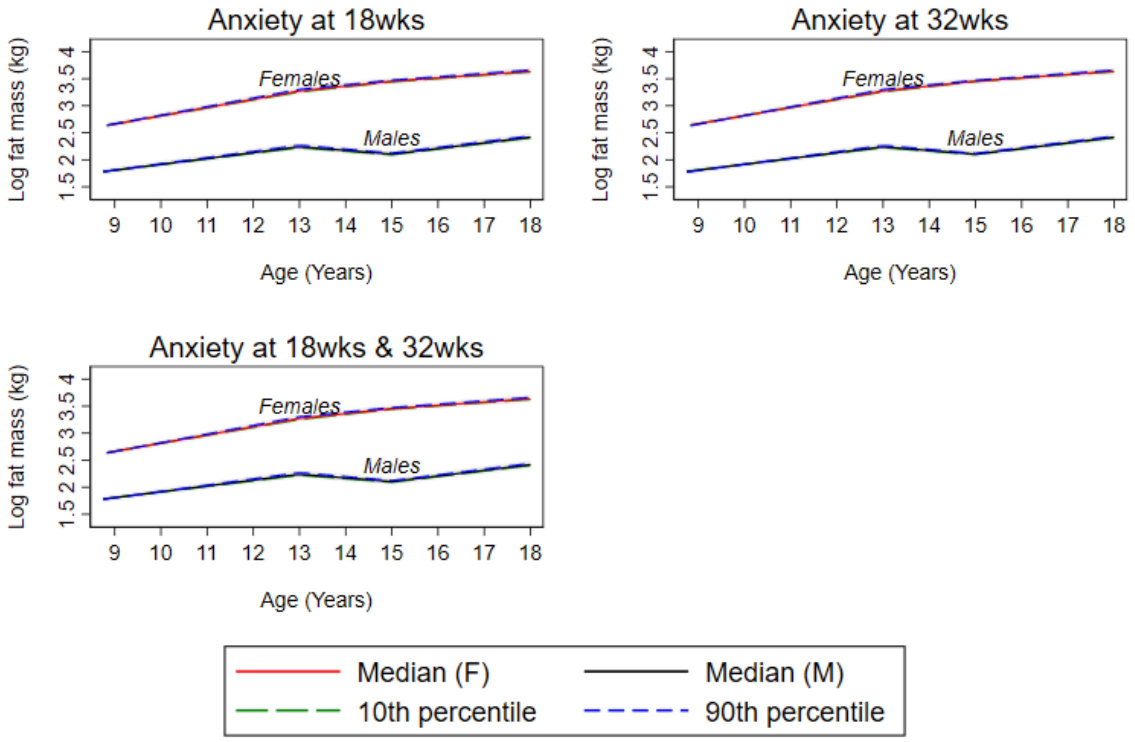

Supplemental Figure S3 Mean predicted confounder adjusted trajectories of log fat mass (9 to 18 years), by continuous maternal anxiety levels during pregnancy

Legend: Trajectories are adjusted for maternal age at birth of offspring, parental household socioeconomic position, parity, maternal pre-pregnancy BMI and maternal smoking during pregnancy. Median, 10th and 90th percentile = 1, 4 and 9 at 18 weeks. Median, 10th and 90th percentile = 1, 4, 10 at 32 weeks. Mean of the 18 and 32 week measures used for analyses of anxiety at 18 weeks and 32 weeks; median, 10th and 90th percentile = 1, 4, 9 in this analysis.

For peer review only - http://bmjopen.bmj.com/site/about/guidelines.xhtml

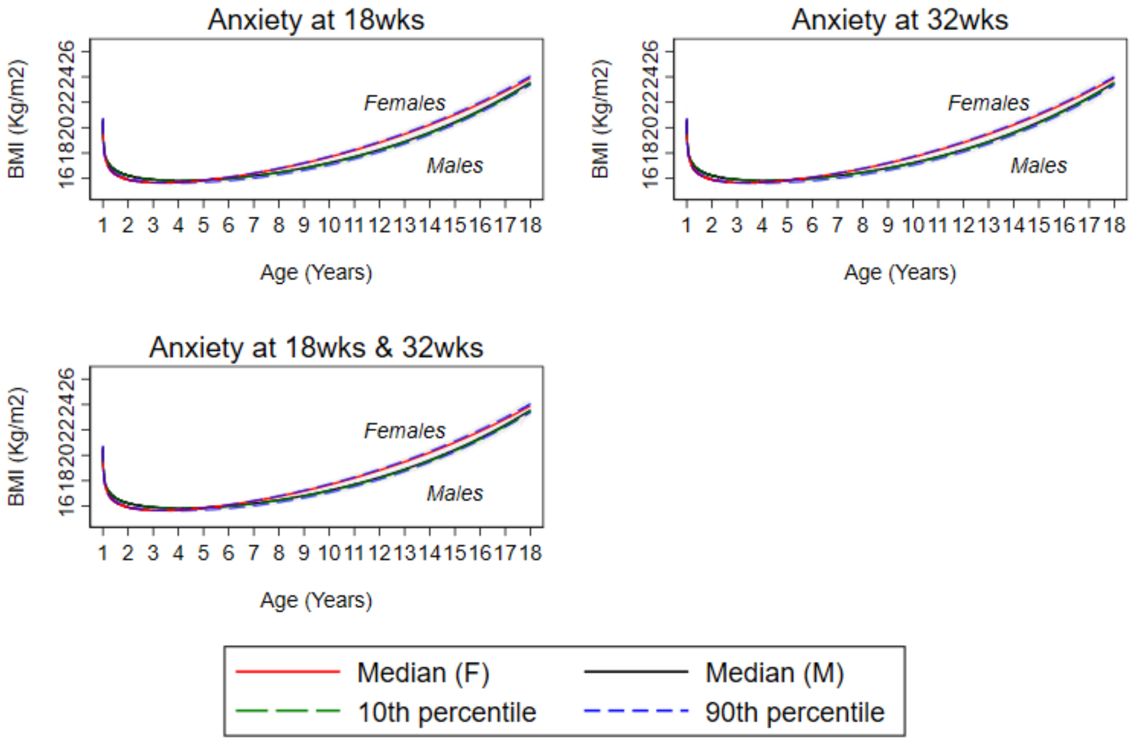

Supplemental Figure S4 Mean predicted confounder adjusted trajectories of log BMI (1 to 18 years), by continuous maternal anxiety levels during pregnancy

Legend: Trajectories are adjusted for maternal age at birth of offspring, parental household socioeconomic position, parity, maternal pre-pregnancy BMI and maternal smoking during pregnancy. Median, 10th and 90th percentile = 1, 4 and 9 at 18 weeks. Median, 10th and 90th percentile = 1, 4, 9 at 32 weeks. Mean of the 18 and 32 week measures used for analyses of anxiety at 18 weeks and 32 weeks; median, 10th and 90th percentile = 1, 4, 9 in this analysis.

For peer review only - http://bmjopen.bmj.com/site/about/guidelines.xhtml

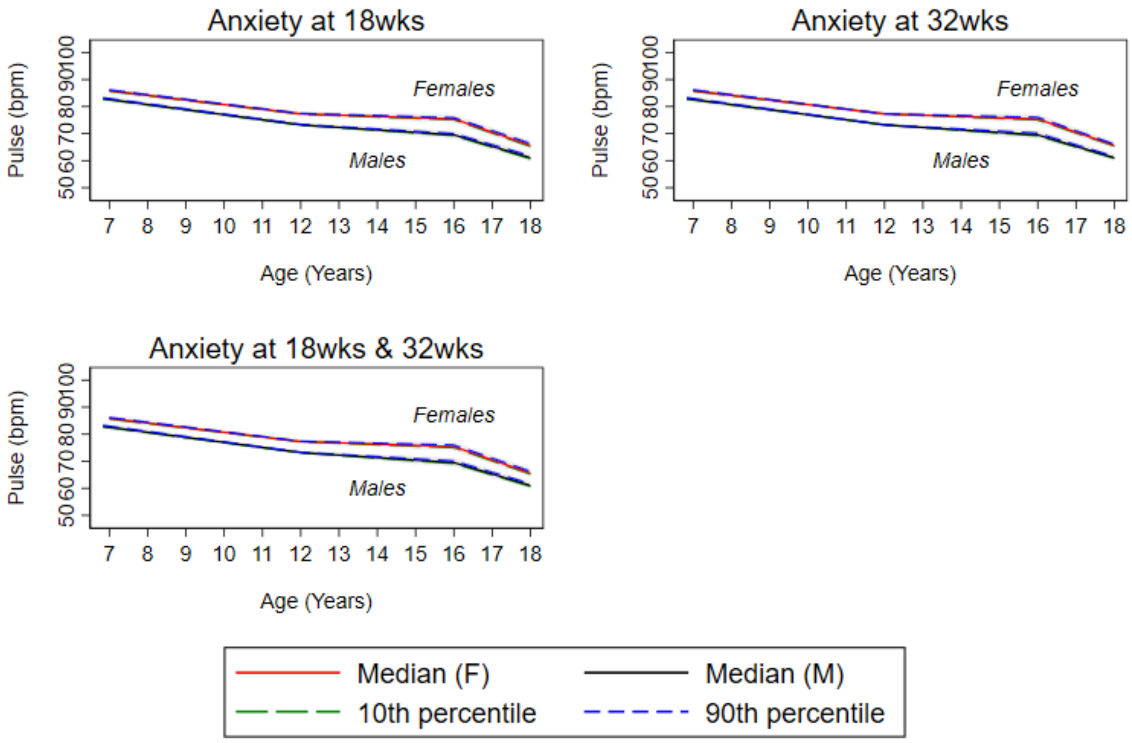

Supplemental Figure S5 Mean predicted confounder adjusted trajectories of pulse rate from 7 to 18 years by continuous maternal anxiety levels during pregnancy

Legend: Trajectories are adjusted for maternal age at birth of offspring, parental household socioeconomic position, parity, maternal pre-pregnancy BMI and maternal smoking during pregnancy. Median, 10th and 90th percentile = 1, 4 and 9 at 18 weeks. Median, 10th and 90th percentile = 1, 4, 10 at 32 weeks. Mean of the 18 and 32 week measures used for analyses of anxiety at 18 weeks and 32 weeks; median, 10th and 90th percentile = 1, 4, 9 in this analysis.

For peer review only - http://bmjopen.bmj.com/site/about/guidelines.xhtml

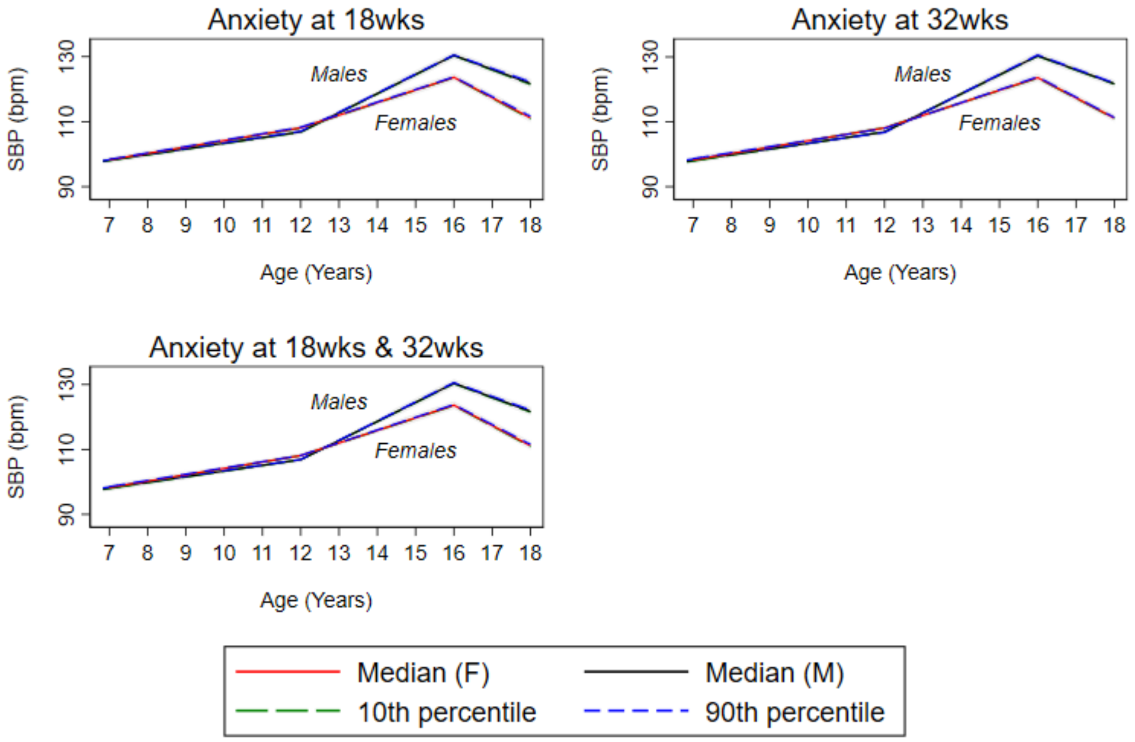

Supplemental Figure S6 Mean predicted confounder adjusted trajectories of SBP from 7 to 18 years by continuous maternal anxiety levels during pregnancy

Legend: Trajectories are adjusted for maternal age at birth of offspring, parental household socioeconomic position, parity, maternal pre-pregnancy BMI and maternal smoking during pregnancy. Median, 10th and 90th percentile = 1, 4 and 9 at 18 weeks. Median, 10th and 90th percentile = 1, 4, 10 at 32 weeks. Mean of the 18 and 32 week measures used for analyses of anxiety at 18 weeks and 32 weeks; median, 10th and 90th percentile = 1, 4, 9 in this analysis.

For peer review only - http://bmjopen.bmj.com/site/about/guidelines.xhtml

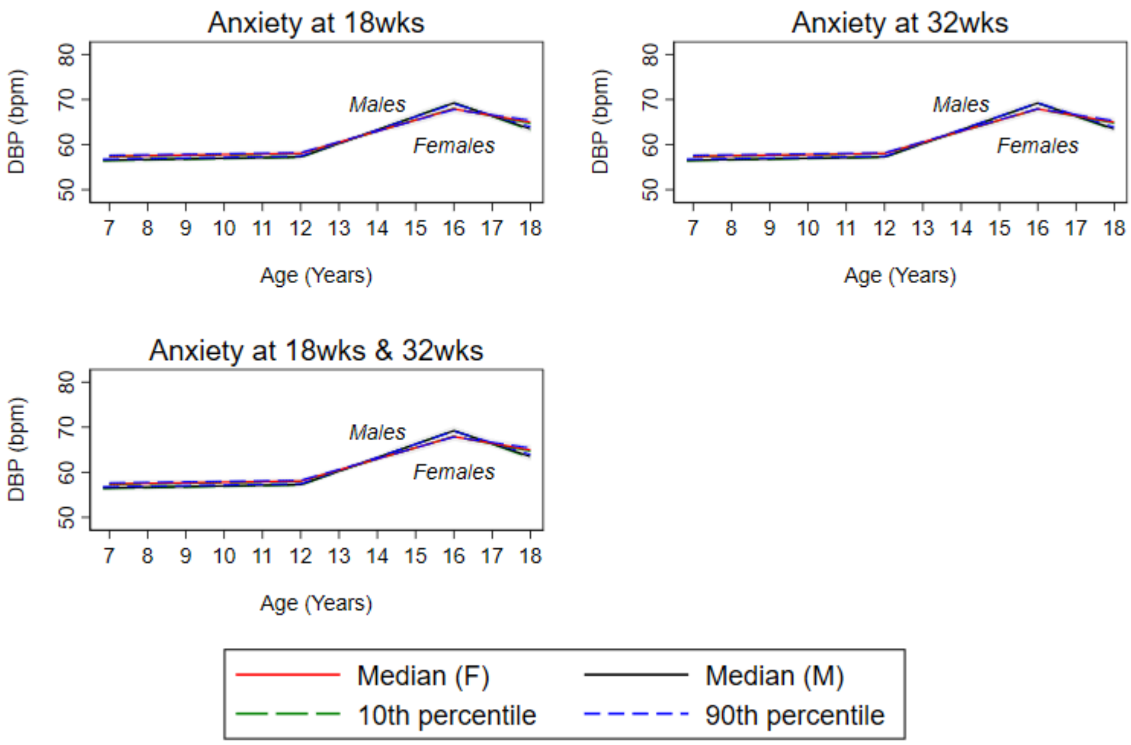

Supplemental Figure S7 Mean predicted confounder adjusted trajectories of DBP from 7 to 18 years by continuous maternal anxiety levels during pregnancy

Legend: Trajectories are adjusted for maternal age at birth of offspring, parental household socioeconomic position, parity, maternal pre-pregnancy BMI and maternal smoking during pregnancy. Median, 10th and 90th percentile = 1, 4 and 9 at 18 weeks. Median, 10th and 90th percentile = 1, 4, 10 at 32 weeks. Mean of the 18 and 32 week measures used for analyses of anxiety at 18 weeks and 32 weeks; median, 10th and 90th percentile = 1, 4, 9 in this analysis.

For peer review only - http://bmjopen.bmj.com/site/about/guidelines.xhtml

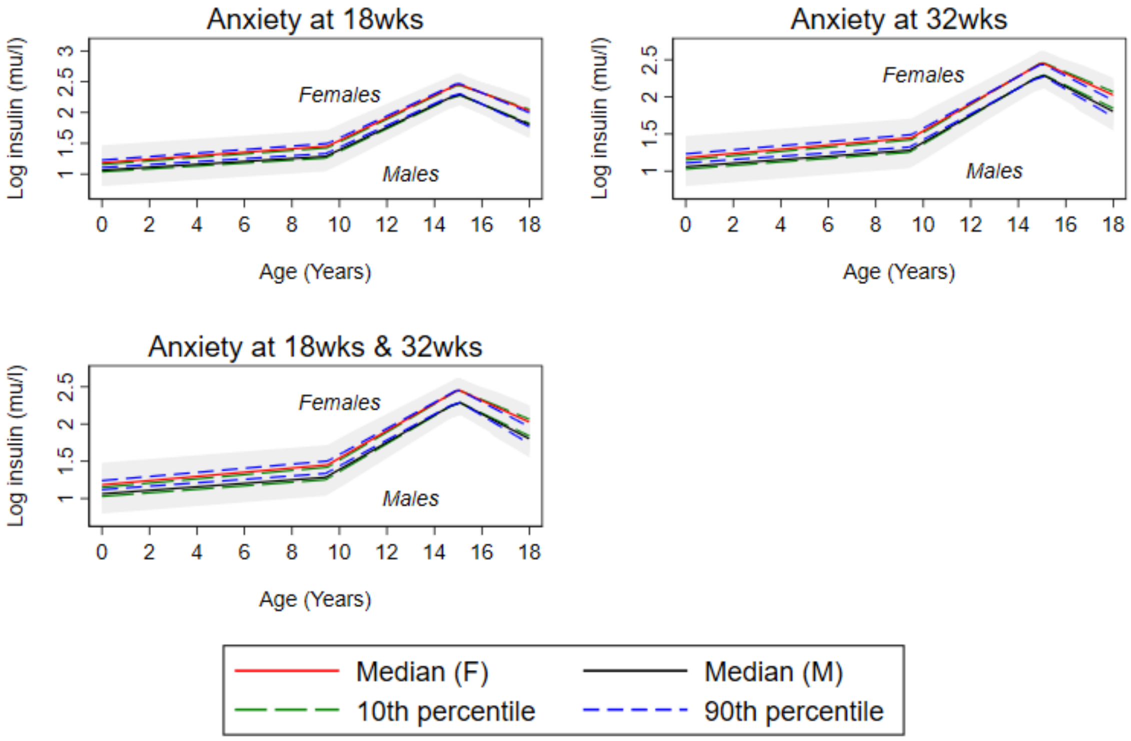

Supplemental Figure S8 Mean predicted confounder adjusted trajectories of log insulin from (birth to 18 years) by continuous maternal anxiety levels during pregnancy

Legend: Trajectories are adjusted for maternal age at birth of offspring, parental household socioeconomic position, parity, maternal pre-pregnancy BMI and maternal smoking during pregnancy. Median, 10th and 90th percentile = 1, 4 and 9 at 18 weeks. Median, 10th and 90th percentile = 1, 4, 9 at 32 weeks. Mean of the 18 and 32 week measures used for analyses of anxiety at 18 weeks and 32 weeks; median, 10th and 90th percentile = 1, 4, 9 in this analysis.

For peer review only - http://bmjopen.bmj.com/site/about/guidelines.xhtml

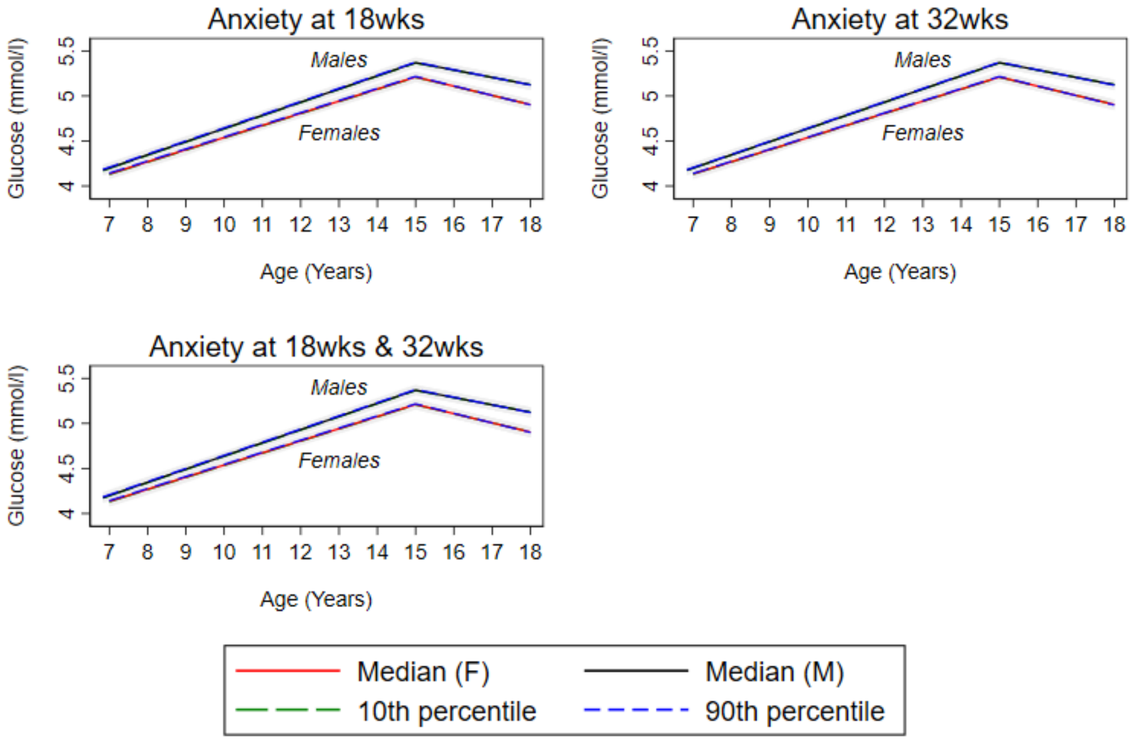

Supplemental Figure S9 Mean predicted confounder adjusted trajectories of glucose (7 to18 years), by continuous maternal anxiety levels during pregnancy

Legend: Trajectories are adjusted for maternal age at birth of offspring, parental household socioeconomic position, parity, maternal pre-pregnancy BMI and maternal smoking during pregnancy. Median, 10th and 90th percentile = 1, 4 and 9 at 18 weeks. Median, 10th and 90th percentile = 1, 4, 10 at 32 weeks. Mean of the 18 and 32 week measures used for analyses of anxiety at 18 weeks and 32 weeks; median, 10th and 90th percentile = 1, 4, 9 in this analysis.

For peer review only - http://bmjopen.bmj.com/site/about/guidelines.xhtml

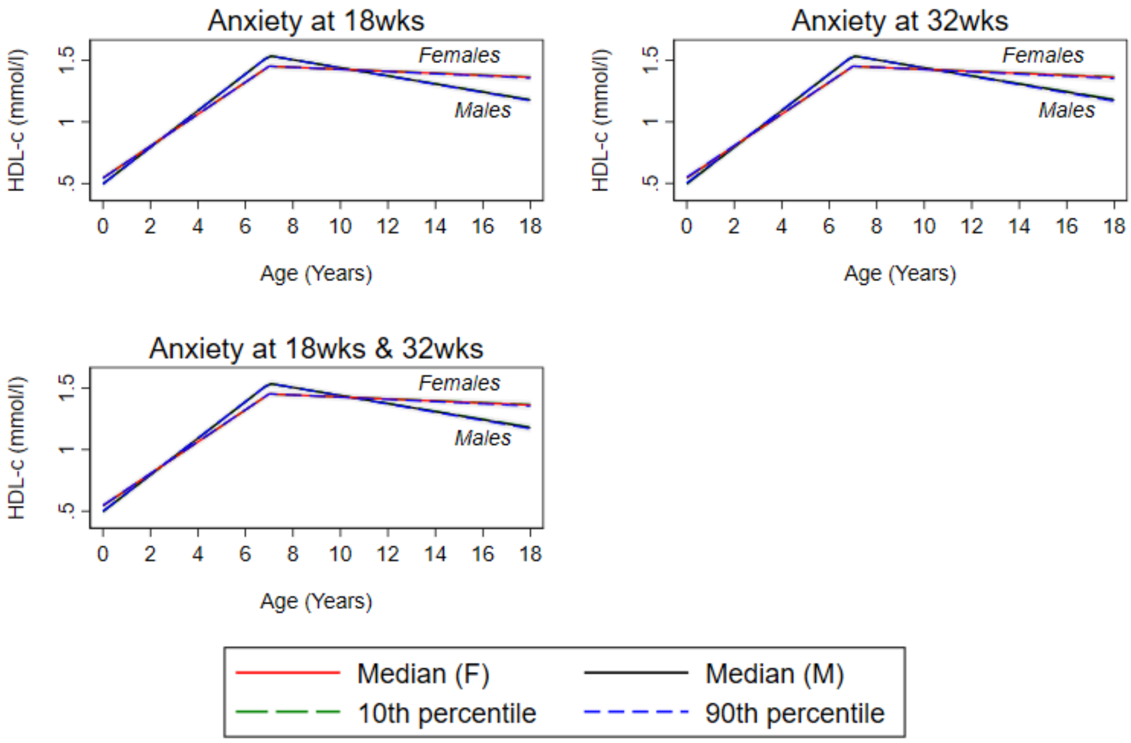

Supplemental Figure S10 Mean predicted confounder adjusted trajectories of HDL-c (birth to18 years), by continuous maternal anxiety levels during pregnancy

Legend: Trajectories are adjusted for maternal age at birth of offspring, parental household socioeconomic position, parity, maternal pre-pregnancy BMI and maternal smoking during pregnancy. Median, 10th and 90th percentile = 1, 4 and 9 at 18 weeks. Median, 10th and 90th percentile = 1, 4, 10 at 32 weeks. Mean of the 18 and 32 week measures used for analyses of anxiety at 18 weeks and 32 weeks; median, 10th and 90th percentile = 1, 4, 9 in this analysis.

For peer review only - http://bmjopen.bmj.com/site/about/guidelines.xhtml

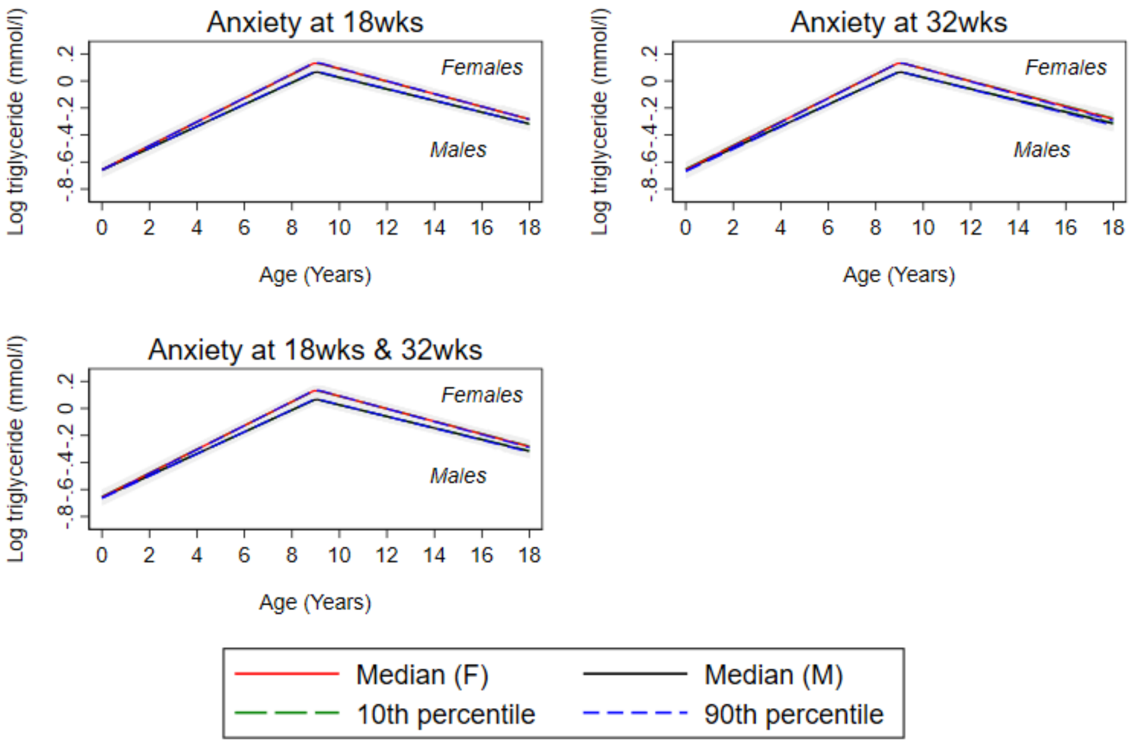

Supplemental Figure S11 Mean predicted confounder adjusted trajectories of triglyceride (birth to18 years), by continuous maternal anxiety levels during pregnancy

Legend: Trajectories are adjusted for maternal age at birth of offspring, parental household socioeconomic position, parity, maternal pre-pregnancy BMI and maternal smoking during pregnancy. Median, 10th and 90th percentile = 1, 4 and 9 at 18 weeks. Median, 10th and 90th percentile = 1, 4, 10 at 32 weeks. Mean of the 18 and 32 week measures used for analyses of anxiety at 18 weeks and 32 weeks; median, 10th and 90th percentile = 1, 4, 9 in this analysis.

For peer review only - http://bmjopen.bmj.com/site/about/guidelines.xhtml

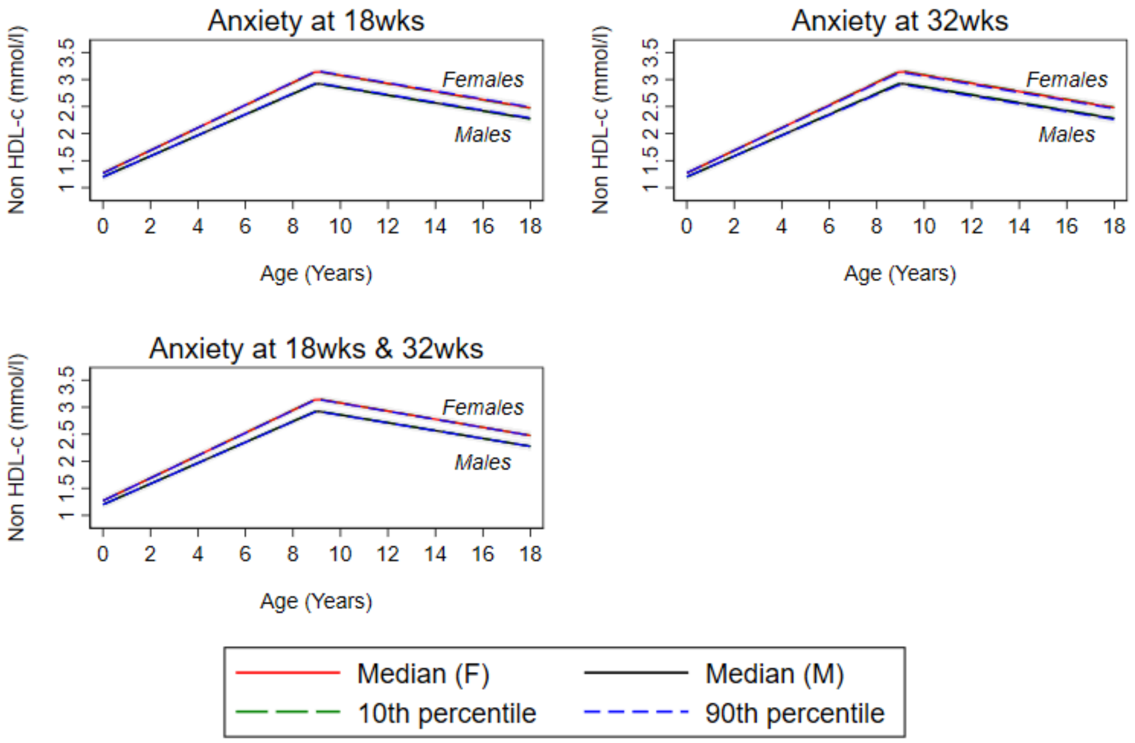

Supplemental Figure S12 Mean predicted confounder adjusted trajectories of non-HDL-c (birth to18 years), by continuous maternal anxiety levels during pregnancy

Legend: Trajectories are adjusted for maternal age at birth of offspring, parental household socioeconomic position, parity, maternal pre-pregnancy BMI and maternal smoking during pregnancy. Median, 10th and 90th percentile = 1, 4 and 9 at 18 weeks. Median, 10th and 90th percentile = 1, 4, 10 at 32 weeks. Mean of the 18 and 32 week measures used for analyses of anxiety at 18 weeks and 32 weeks; median, 10th and 90th percentile = 1, 4, 9 in this analysis.

For peer review only - http://bmjopen.bmj.com/site/about/guidelines.xhtml

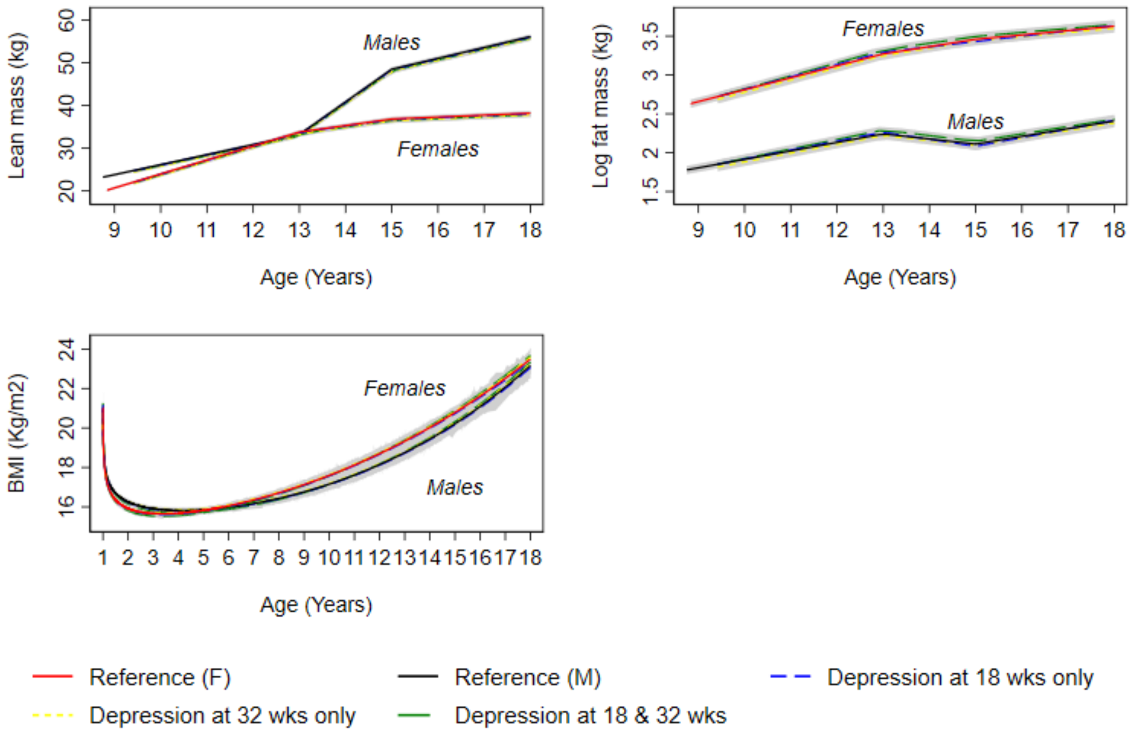

Supplemental Figure S13 Mean predicted confounder adjusted trajectories of lean mass (9 to 18 years), log fat mass (9 to 18 years) and log BMI (1 to 18 years), by maternal depression levels during pregnancy

Legend: Trajectories are adjusted for maternal age at birth of offspring, parental household socioeconomic position, parity, maternal pre-pregnancy BMI and maternal smoking during pregnancy.

For peer review only - http://bmjopen.bmj.com/site/about/guidelines.xhtml

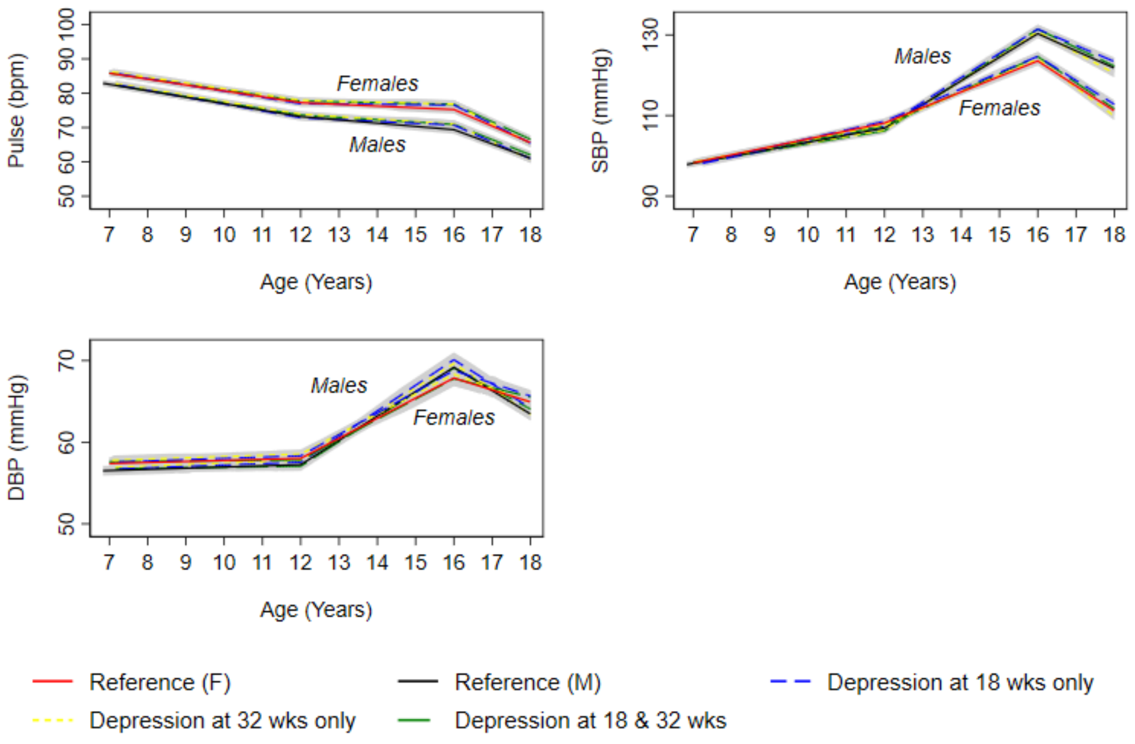

Supplemental Figure S14 Mean predicted confounder adjusted trajectories of pulse rate, SBP and DBP from 7 to 18 years, by maternal depression levels during pregnancy

Legend: DBP, diastolic blood pressure; SBP, systolic blood pressure.

Trajectories are adjusted for maternal age at birth of offspring, parental household socioeconomic position, parity, maternal pre-pregnancy BMI and maternal smoking during pregnancy. Depression at 18 and 32 weeks gestation defined as ≥85th percentile of depression score based on Edinburgh Postnatal Depression Scale for the whole cohort at each time point separately ((≥85th percentile = 13 at both 18 and 32 weeks gestation).

For peer review only

For peer review only - http://bmjopen.bmj.com/site/about/guidelines.xhtml

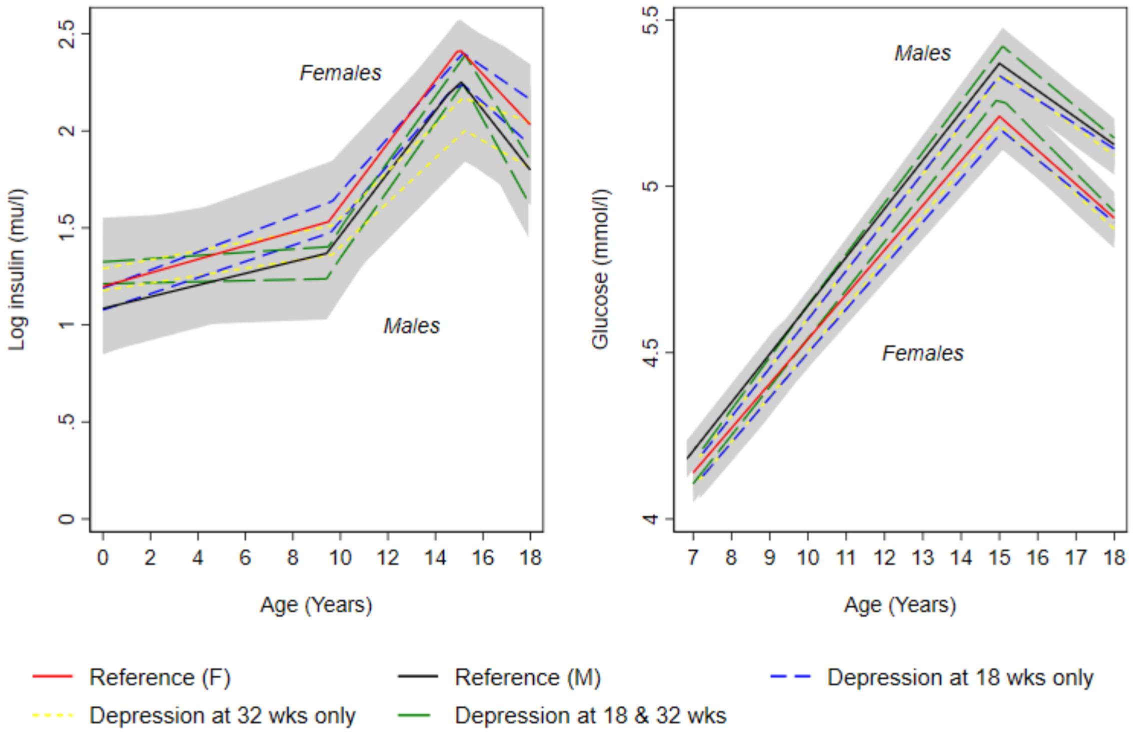

Supplemental Figure S15 Mean predicted confounder adjusted trajectories of log insulin (birth to 18 years) and glucose (7 to 18 years), by maternal depression levels during pregnancy

Legend: Trajectories are adjusted for maternal age at birth of offspring, parental household socioeconomic position, parity, maternal pre-pregnancy BMI and maternal smoking during pregnancy. Depression at 18 and 32 weeks gestation defined as ≥85th percentile of depression score based on Edinburgh Postnatal Depression Scale for the whole cohort at each time point separately ((≥85th percentile = 13 at both 18 and 32 weeks gestation).

For peer review only - http://bmjopen.bmj.com/site/about/guidelines.xhtml

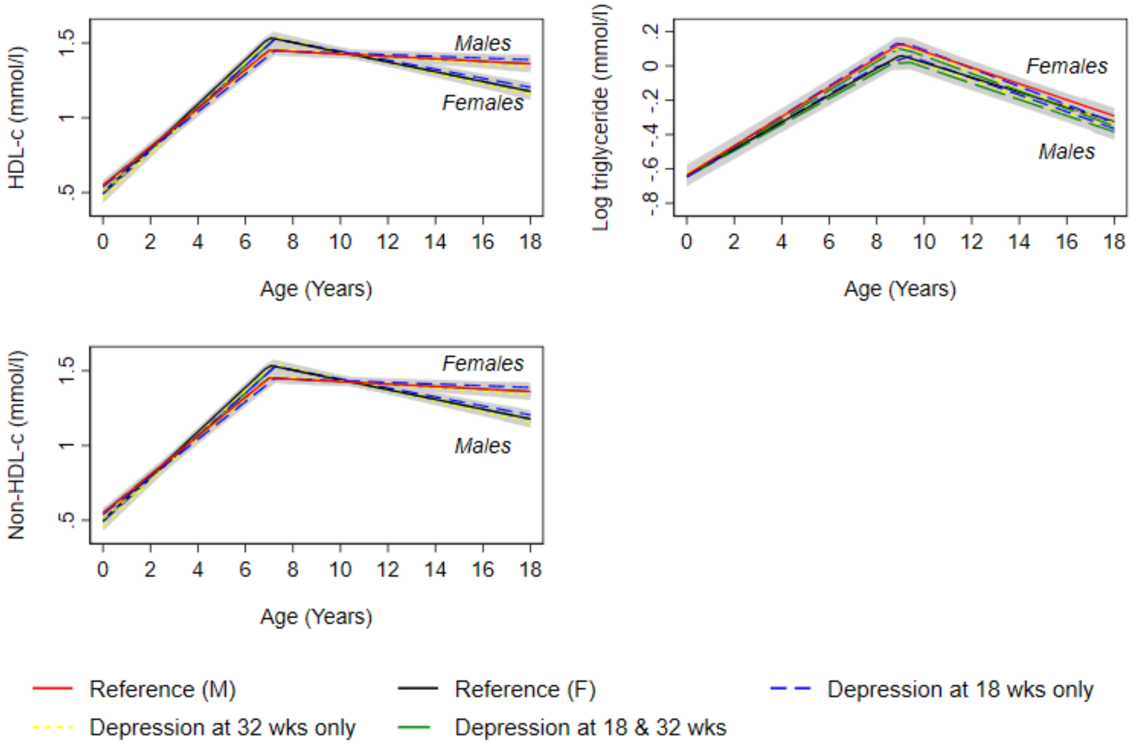

Supplemental Figure S16 Mean predicted confounder adjusted trajectories of HDL-c, log triglyceride and non-HDL-c from birth to 18 years, by maternal depression levels during pregnancy

Legend: Trajectories are adjusted for maternal age at birth of offspring, parental household socioeconomic position, parity, maternal pre-pregnancy BMI and maternal smoking during pregnancy. Depression at 18 and 32 weeks gestation defined as ≥85th percentile of depression score based on Edinburgh Postnatal Depression Scale for the whole cohort at each time point separately ((≥85th percentile = 13 at both 18 and 32 weeks gestation).

For peer review only - http://bmjopen.bmj.com/site/about/guidelines.xhtml

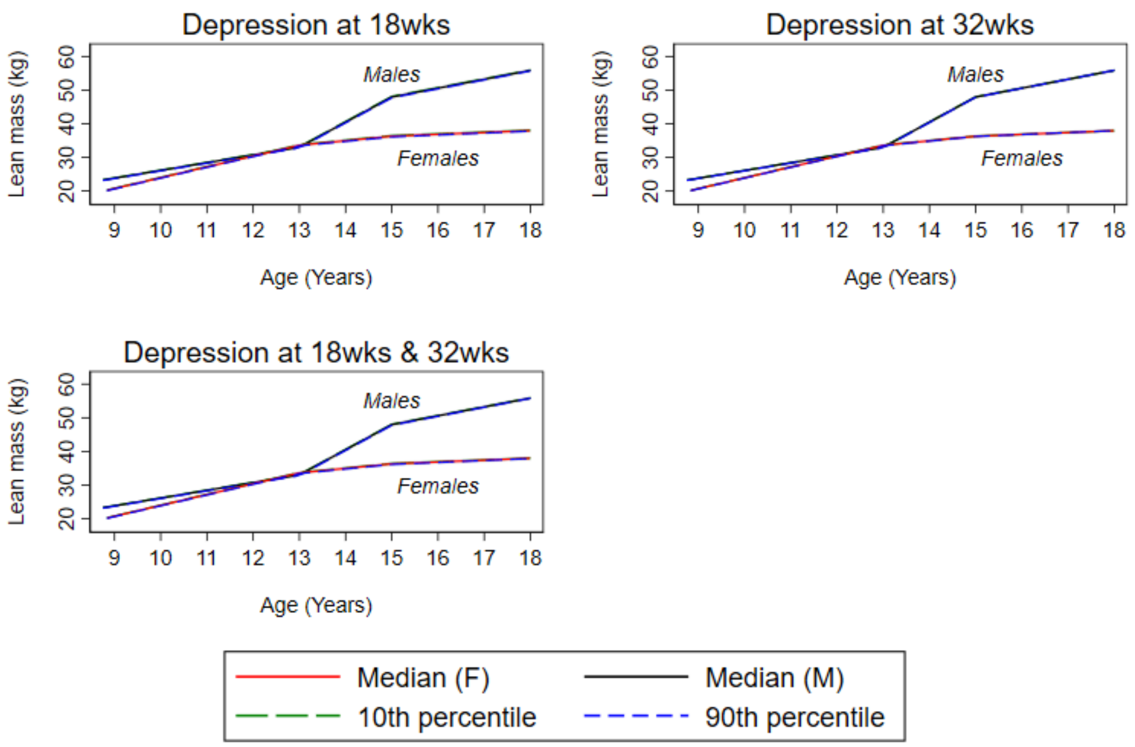

Supplemental Figure S17 Mean predicted confounder adjusted trajectories of lean mass (9 to 18 years), by continuous maternal depression levels during pregnancy
Legend: Trajectories are adjusted for maternal age at birth of offspring, parental household socioeconomic position, parity, maternal pre-pregnancy BMI and maternal smoking during pregnancy. Median, 10th and 90th percentile = 1, 6 and 12 at 18 weeks. Median, 10th and 90th percentile = 1, 6, 13 at 32 weeks. Mean of the 18 and 32 week measures used for analyses of anxiety at 18 weeks and 32 weeks; median, 10th and 90th percentile = 1.5, 5.5, 12 in this analysis.

For peer review only - http://bmjopen.bmj.com/site/about/guidelines.xhtml

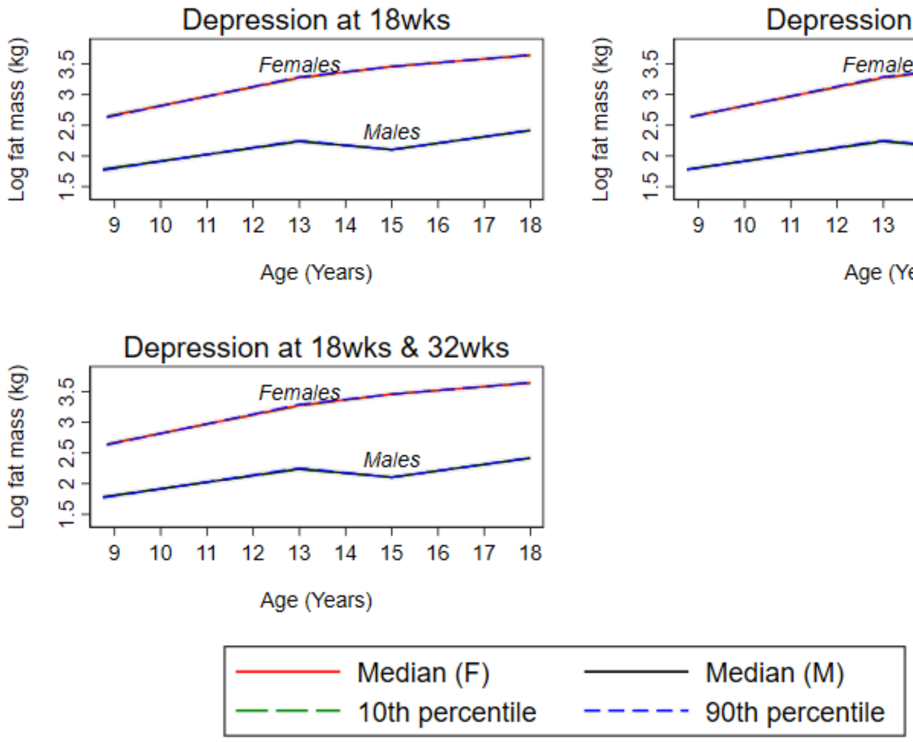

Supplemental Figure S18 Mean predicted confounder adjusted trajectories of log fat mass (9 to 18 years), by continuous maternal depression levels during pregnancy

Legend: Trajectories are adjusted for maternal age at birth of offspring, parental household socioeconomic position, parity, maternal pre-pregnancy BMI and maternal smoking during pregnancy. Median, 10th and 90th percentile = 1, 6 and 12 at 18 weeks. Median, 10th and 90th percentile = 1, 6, 13 at 32 weeks. Mean of the 18 and 32 week measures used for analyses of anxiety at 18 weeks and 32 weeks; median, 10th and 90th percentile = 1.5, 5.5, 12 in this analysis.

For peer review only - http://bmjopen.bmj.com/site/about/guidelines.xhtml

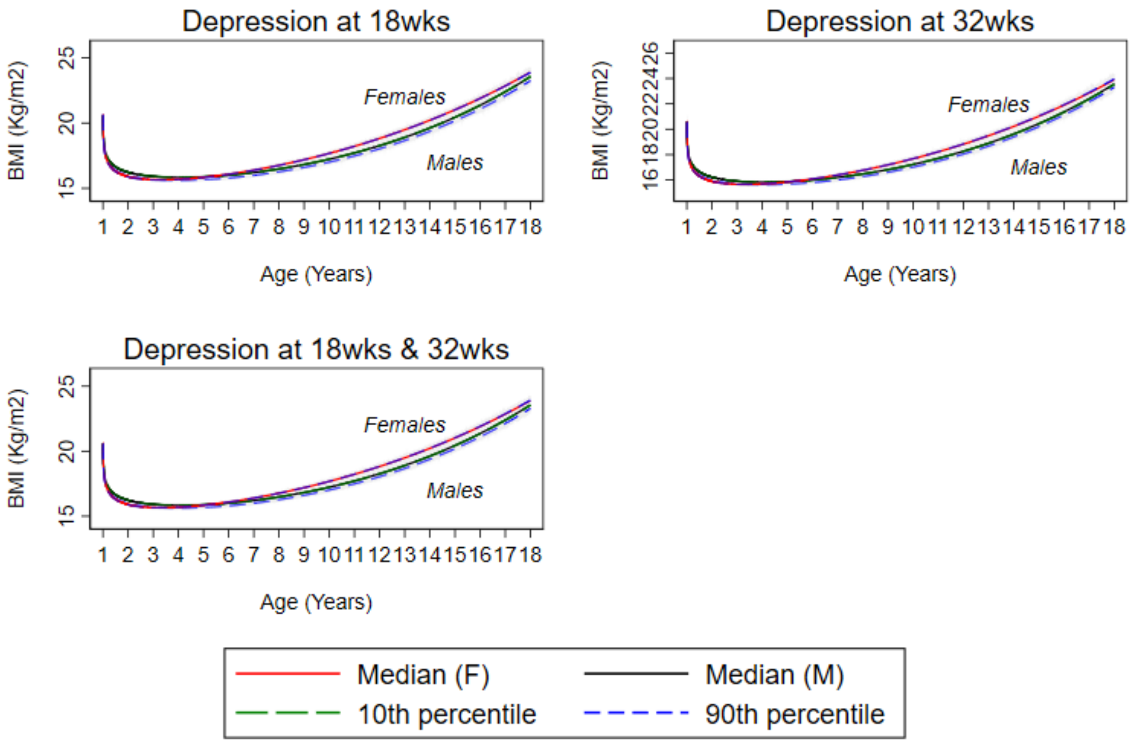

Supplemental Figure S19 Mean predicted confounder adjusted trajectories of log BMI (1 to 18 years), by continuous maternal depression levels during pregnancy

Legend: Trajectories are adjusted for maternal age at birth of offspring, parental household socioeconomic position, parity, maternal pre-pregnancy BMI and maternal smoking during pregnancy. Median, 10th and 90th percentile = 1, 6 and 13 at 18 weeks. Median, 10th and 90th percentile = 1, 6, 13 at 32 weeks. Mean of the 18 and 32 week measures used for analyses of anxiety at 18 weeks and 32 weeks; median, 10th and 90th percentile = 1.5, 5.5, 12 in this analysis.

For peer review only

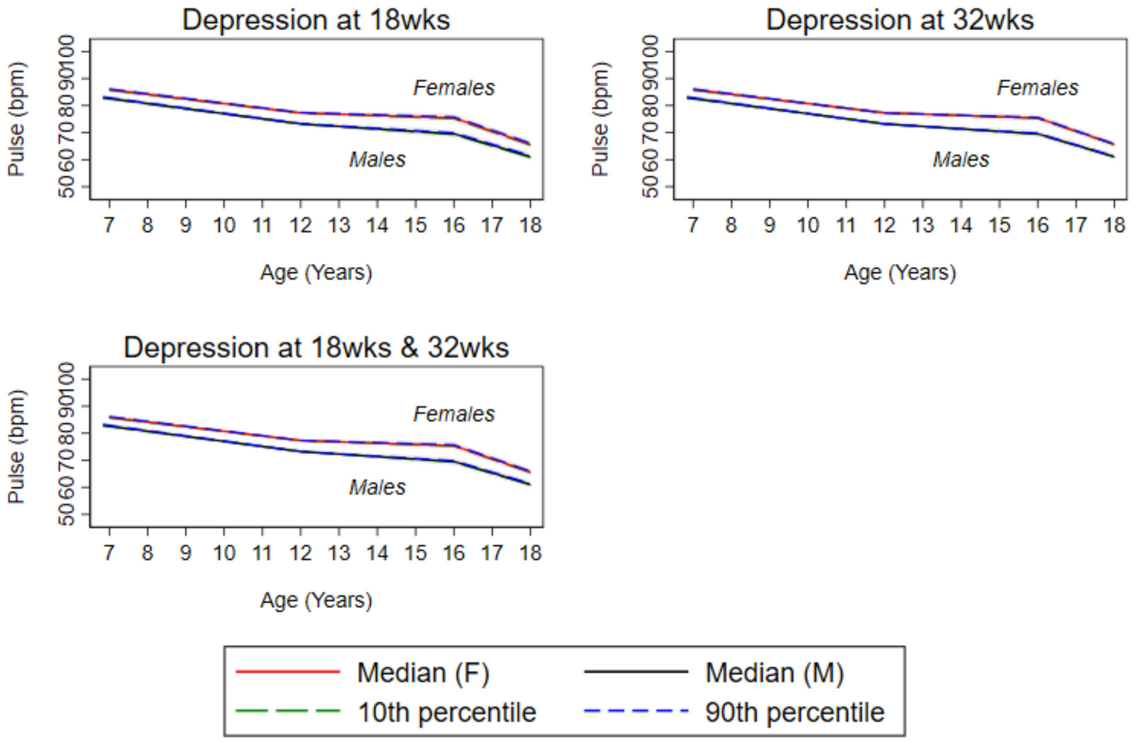

Supplemental Figure S20 Mean predicted confounder adjusted trajectories of pulse rate from 7 to 18 years by continuous maternal depression levels during pregnancy

Legend: Trajectories are adjusted for maternal age at birth of offspring, parental household socioeconomic position, parity, maternal pre-pregnancy BMI and maternal smoking during pregnancy. Median, 10th and 90th percentile = 1, 6 and 13 at 18 weeks. Median, 10th and 90th percentile = 1, 6, 13 at 32 weeks. Mean of the 18 and 32 week measures used for analyses of anxiety at 18 weeks and 32 weeks; median, 10th and 90th percentile = 1.5, 5.5, 12 in this analysis.

For peer review only - http://bmjopen.bmj.com/site/about/guidelines.xhtml

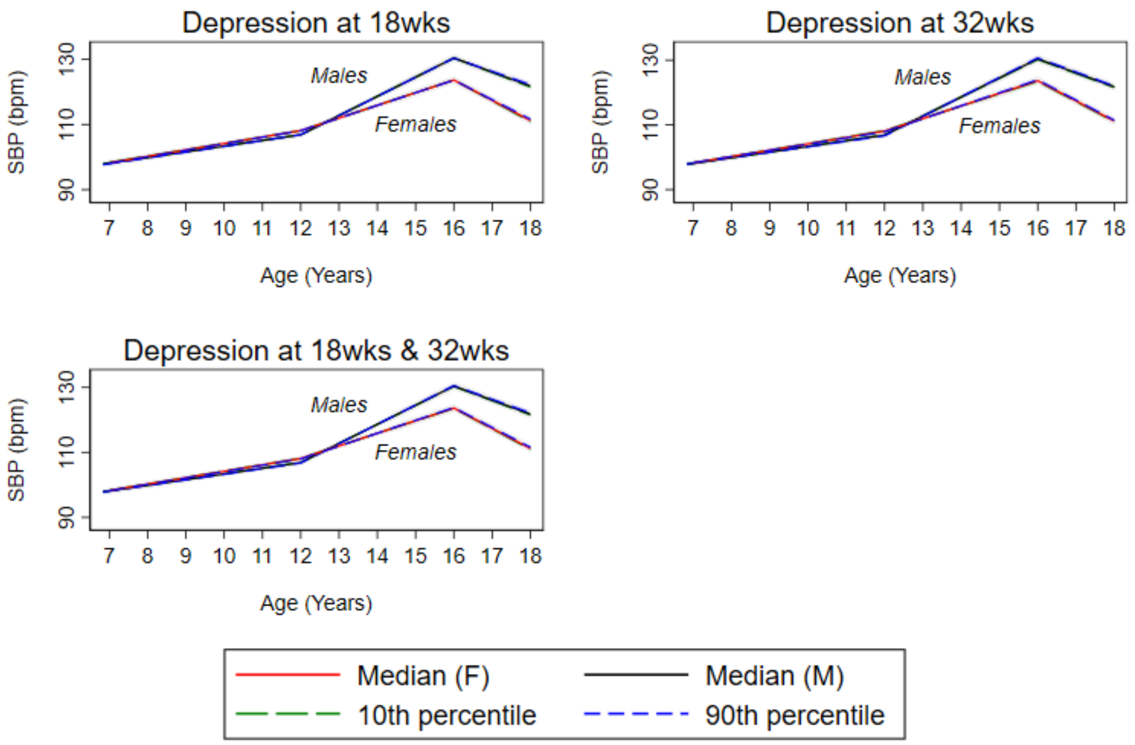

Supplemental Figure S21 Mean predicted confounder adjusted trajectories of SBP from 7 to 18 years by continuous maternal depression levels during pregnancy

Legend: Trajectories are adjusted for maternal age at birth of offspring, parental household socioeconomic position, parity, maternal pre-pregnancy BMI and maternal smoking during pregnancy. Median, 10th and 90th percentile = 1, 6 and 13 at 18 weeks. Median, 10th and 90th percentile = 1, 6, 13 at 32 weeks. Mean of the 18 and 32 week measures used for analyses of anxiety at 18 weeks and 32 weeks; median, 10th and 90th percentile = 1.5, 5.5, 12 in this analysis.

For peer review only - http://bmjopen.bmj.com/site/about/guidelines.xhtml

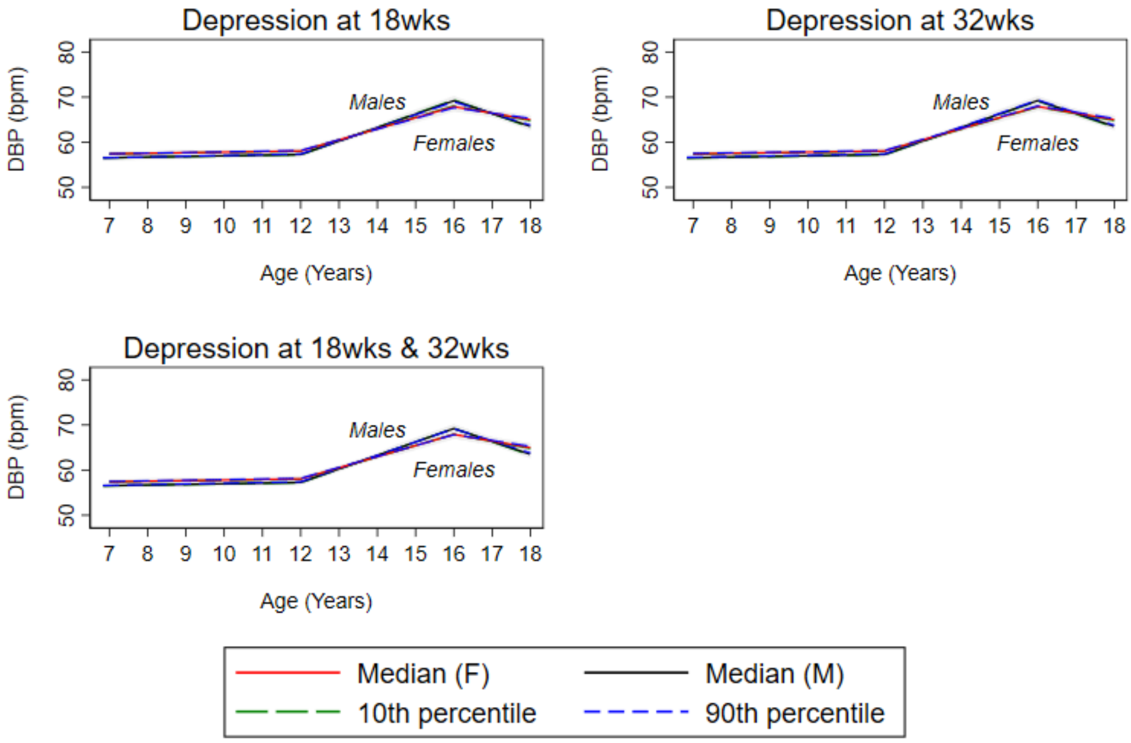

Supplemental Figure S22 Mean predicted confounder adjusted trajectories of DBP from 7 to 18 years by continuous maternal depression levels during pregnancy

Legend: Trajectories are adjusted for maternal age at birth of offspring, parental household socioeconomic position, parity, maternal pre-pregnancy BMI and maternal smoking during pregnancy. Median, 10th and 90th percentile = 1, 6 and 13 at 18 weeks. Median, 10th and 90th percentile = 1, 6, 13 at 32 weeks. Mean of the 18 and 32 week measures used for analyses of anxiety at 18 weeks and 32 weeks; median, 10th and 90th percentile = 1.5, 5.5, 12 in this analysis.

For peer review only - http://bmjopen.bmj.com/site/about/guidelines.xhtml

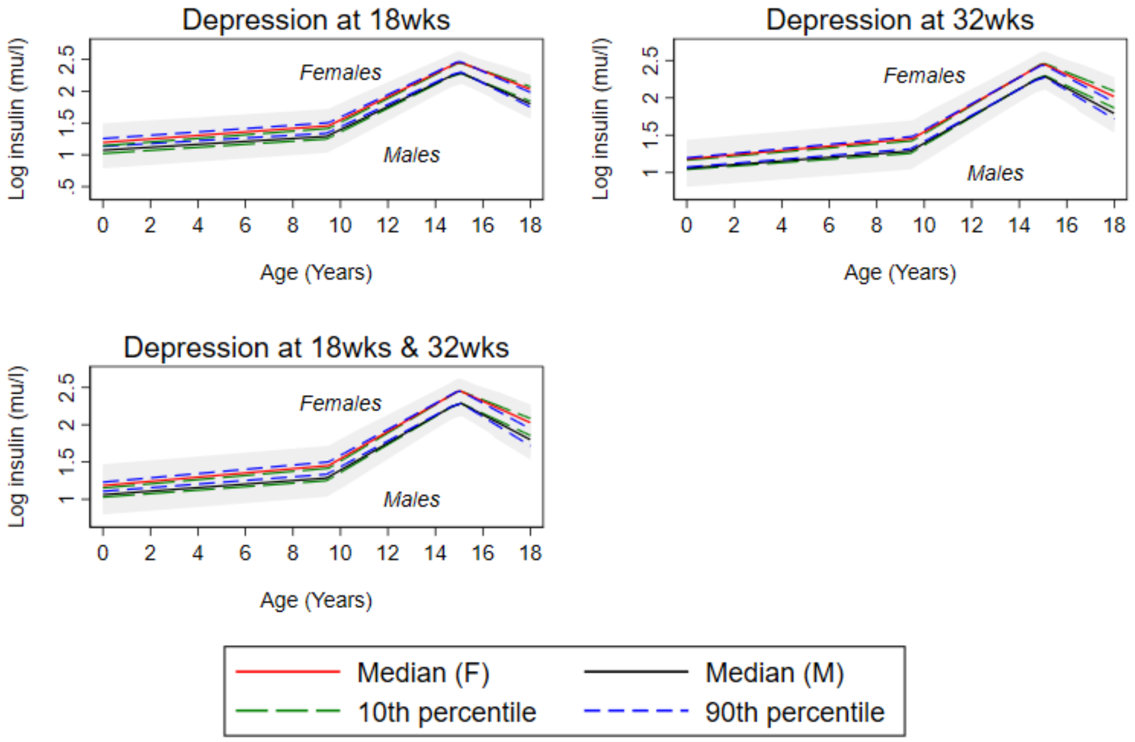

Supplemental Figure S23 Mean predicted confounder adjusted trajectories of log insulin from (birth to 18 years) by continuous maternal depression levels during pregnancy
Legend: Trajectories are adjusted for maternal age at birth of offspring, parental household socioeconomic position, parity, maternal pre-pregnancy BMI and maternal smoking during pregnancy. Median, 10th and 90th percentile = 1, 5 and 13 at 18 weeks. Median, 10th and 90th percentile = 0, 6, 13 at 32 weeks. Mean of the 18 and 32 week measures used for analyses of anxiety at 18 weeks and 32 weeks; median, 10th and 90th percentile = 1, 5.5, 12 in this analysis.

For peer review only - http://bmjopen.bmj.com/site/about/guidelines.xhtml

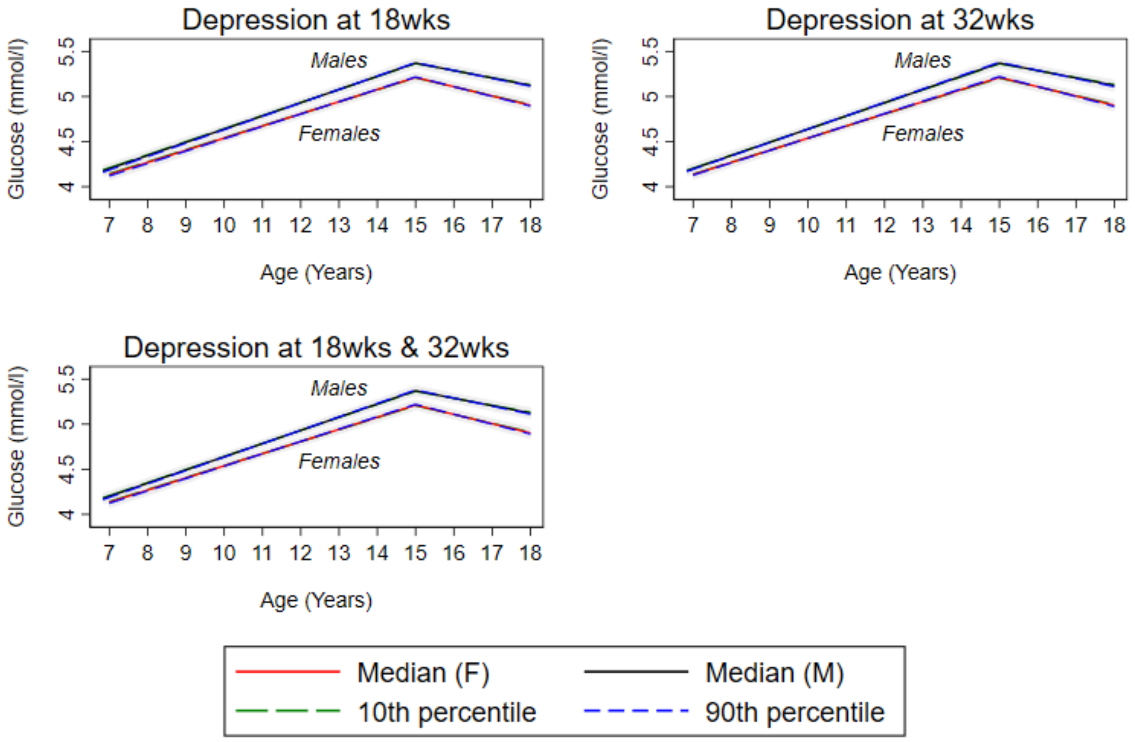

Supplemental Figure S24 Mean predicted confounder adjusted trajectories of glucose (7 to18 years), by continuous maternal depression levels during pregnancy
Legend: Trajectories are adjusted for maternal age at birth of offspring, parental household socioeconomic position, parity, maternal pre-pregnancy BMI and maternal smoking during pregnancy. Median, 10th and 90th percentile = 1, 6 and 13 at 18 weeks. Median, 10th and 90th percentile = 1, 6, 13 at 32 weeks. Mean of the 18 and 32 week measures used for analyses of anxiety at 18 weeks and 32 weeks; median, 10th and 90th percentile = 1.5, 5.5, 12 in this analysis.

For peer review only - http://bmjopen.bmj.com/site/about/guidelines.xhtml

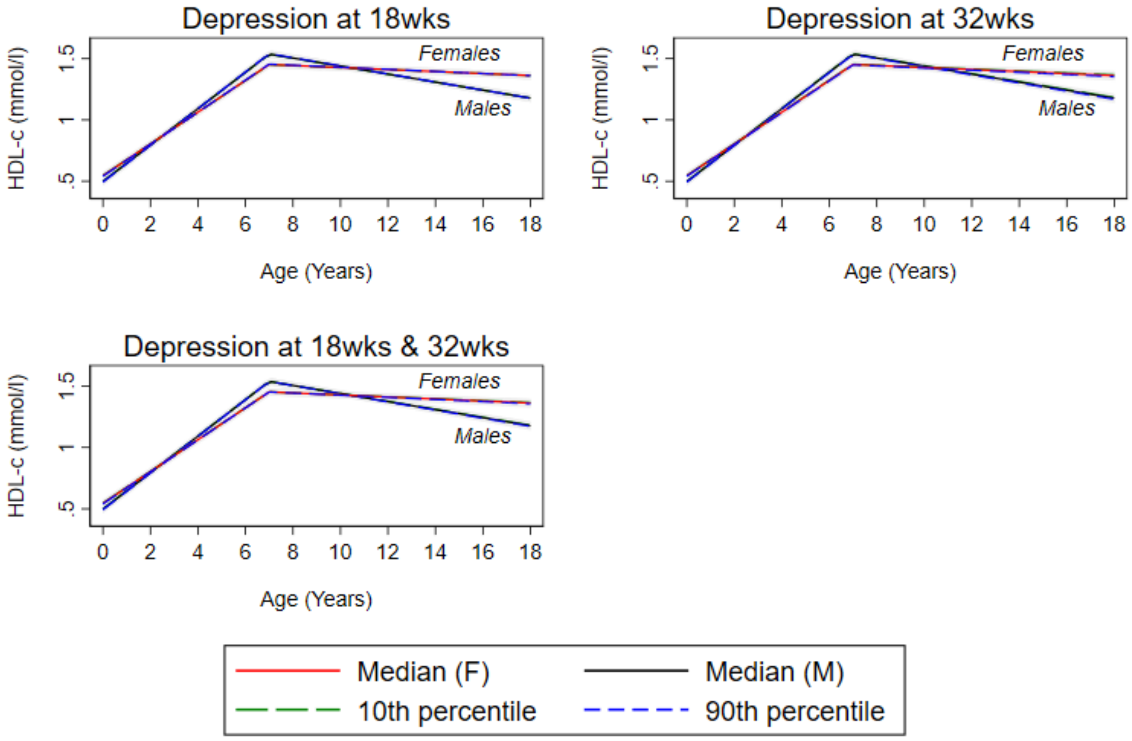

Supplemental Figure S25 Mean predicted confounder adjusted trajectories of HDL-c (birth to18 years), by continuous maternal depression levels during pregnancy

Legend: Trajectories are adjusted for maternal age at birth of offspring, parental household socioeconomic position, parity, maternal pre-pregnancy BMI and maternal smoking during pregnancy. Median, 10th and 90th percentile = 1, 6 and 13 at 18 weeks. Median, 10th and 90th percentile = 1, 6, 13 at 32 weeks. Mean of the 18 and 32 week measures used for analyses of anxiety at 18 weeks and 32 weeks; median, 10th and 90th percentile = 1.5, 5.5, 12.5 in this analysis.

For peer review only - http://bmjopen.bmj.com/site/about/guidelines.xhtml

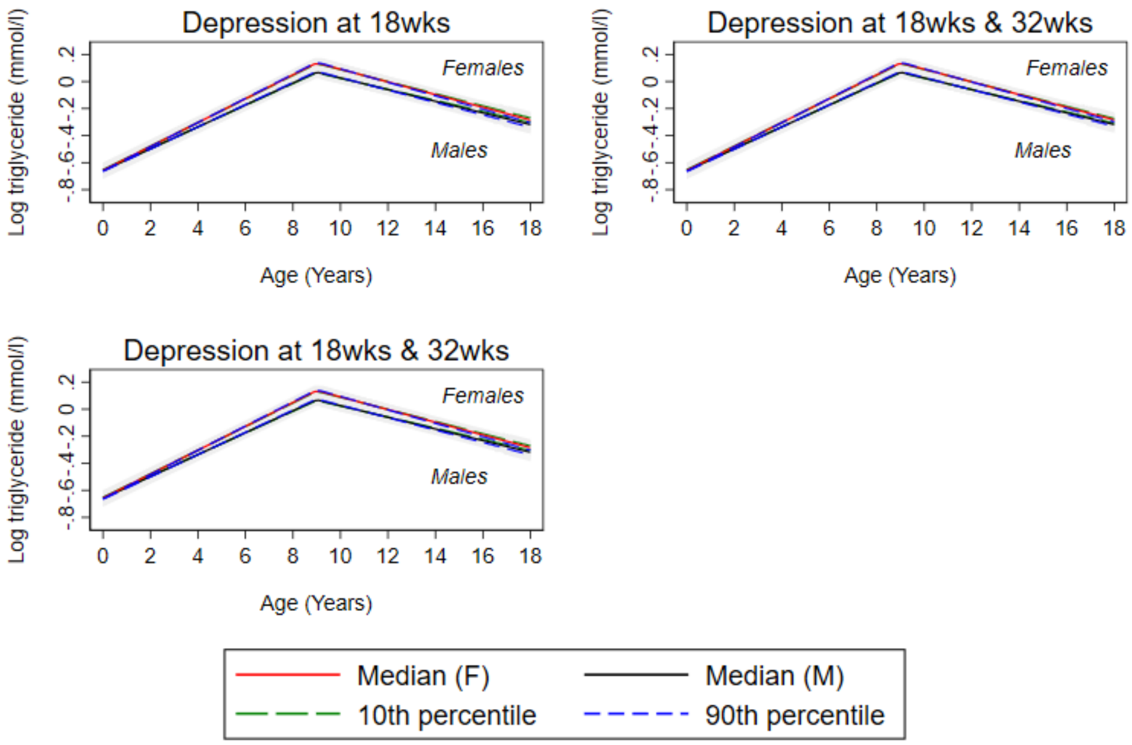

Supplemental Figure S26 Mean predicted confounder adjusted trajectories of triglyceride (birth to18 years), by continuous maternal depression levels during pregnancy

Legend: Trajectories are adjusted for maternal age at birth of offspring, parental household socioeconomic position, parity, maternal pre-pregnancy BMI and maternal smoking during pregnancy. Median, 10th and 90th percentile = 1, 6 and 13 at 18 weeks. Median, 10th and 90th percentile = 1, 6, 13 at 32 weeks. Mean of the 18 and 32 week measures used for analyses of anxiety at 18 weeks and 32 weeks; median, 10th and 90th percentile = 1.5, 6, 12.5 in this analysis.

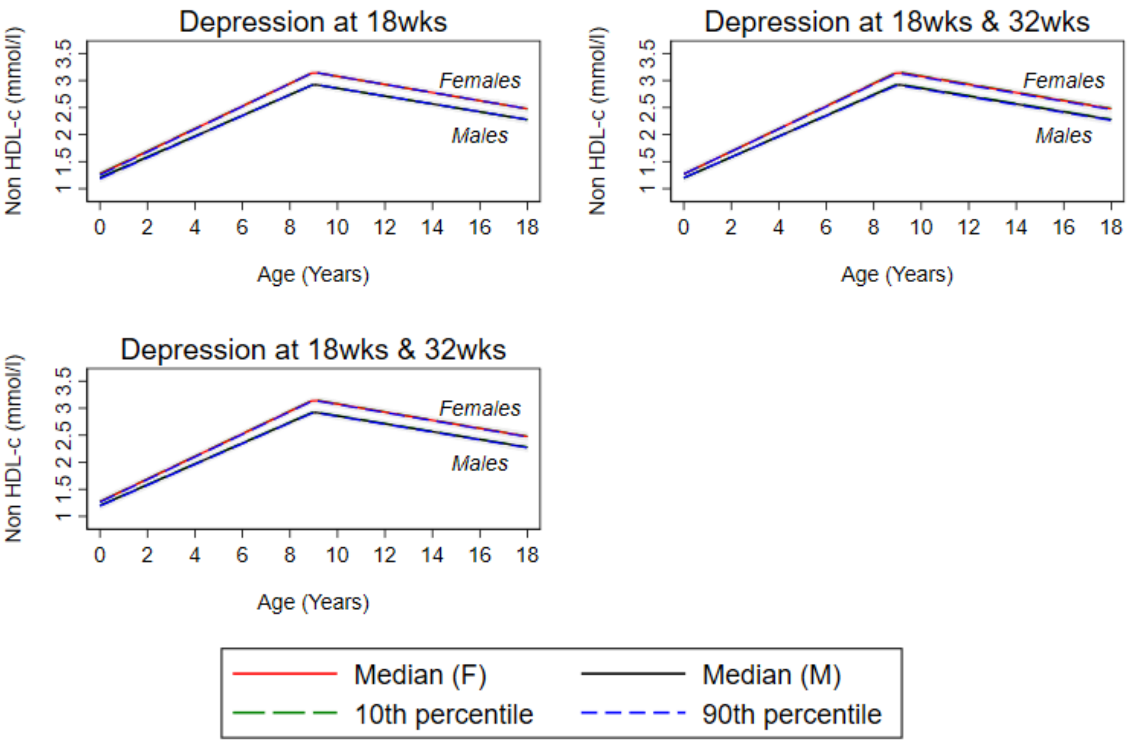

Supplemental Figure S27 Mean predicted confounder adjusted trajectories of non-HDL-c (birth to18 years), by continuous maternal depression levels during pregnancy

Legend: Trajectories are adjusted for maternal age at birth of offspring, parental household socioeconomic position, parity, maternal pre-pregnancy BMI and maternal smoking during pregnancy. Median, 10th and 90th percentile = 1, 6 and 13 at 18 weeks. Median, 10th and 90th percentile = 1, 6, 13 at 32 weeks. Mean of the 18 and 32 week measures used for analyses of anxiety at 18 weeks and 32 weeks; median, 10th and 90th percentile = 1.5, 6, 12.5 in this analysis.

For peer review only - http://bmjopen.bmj.com/site/about/guidelines.xhtml

STROBE Statement—Checklist of items that should be included in reports of ***cohort studies***

	Item No	Recommendation	Page No
**Title and abstract**	1	(*a*) Indicate the study's design with a commonly used term in the title or the abstract<br>(*b*) Provide in the abstract an informative and balanced summary of what was done and what was found	1
**Introduction**			
Background/rationale	2	Explain the scientific background and rationale for the investigation being reported	4-5
Objectives	3	State specific objectives, including any prespecified hypotheses	5
**Methods**			
Study design	4	Present key elements of study design early in the paper	6
Setting	5	Describe the setting, locations, and relevant dates, including periods of recruitment, exposure, follow-up, and data collection	6
Participants	6	(*a*) Give the eligibility criteria, and the sources and methods of selection of participants. Describe methods of follow-up<br>(*b*) For matched studies, give matching criteria and number of exposed and unexposed	6
Variables	7	Clearly define all outcomes, exposures, predictors, potential confounders, and effect modifiers. Give diagnostic criteria, if applicable	6-9
Data sources/ measurement	8*	For each variable of interest, give sources of data and details of methods of assessment (measurement). Describe comparability of assessment methods if there is more than one group	6-9
Bias	9	Describe any efforts to address potential sources of bias	n/a
Study size	10	Explain how the study size was arrived at	6
Quantitative variables	11	Explain how quantitative variables were handled in the analyses. If applicable, describe which groupings were chosen and why	9-11
Statistical methods	12	(*a*) Describe all statistical methods, including those used to control for confounding<br>(*b*) Describe any methods used to examine subgroups and interactions<br>(*c*) Explain how missing data were addressed<br>(*d*) If applicable, explain how loss to follow-up was addressed<br>(*e*) Describe any sensitivity analyses	9-11
**Results**			
Participants	13*	(a) Report numbers of individuals at each stage of study—eg numbers potentially eligible, examined for eligibility, confirmed eligible, included in the study, completing follow-up, and analysed<br>(b) Give reasons for non-participation at each stage<br>(c) Consider use of a flow diagram	12-13
Descriptive data	14*	(a) Give characteristics of study participants (eg demographic, clinical, social) and information on exposures and potential confounders<br>(b) Indicate number of participants with missing data for each variable of interest<br>(c) Summarise follow-up time (eg, average and total amount)	12-13
Outcome data	15*	Report numbers of outcome events or summary measures over time	12

For peer review only

Main results	16	(*a*) Give unadjusted estimates and, if applicable, confounder-adjusted estimates and their precision (eg, 95% confidence interval). Make clear which confounders were adjusted for and why they were included<br>(*b*) Report category boundaries when continuous variables were categorized<br>(*c*) If relevant, consider translating estimates of relative risk into absolute risk for a meaningful time period	12-16
Other analyses	17	Report other analyses done—eg analyses of subgroups and interactions, and sensitivity analyses	Supplementary files
**Discussion**			
Key results	18	Summarise key results with reference to study objectives	17
Limitations	19	Discuss limitations of the study, taking into account sources of potential bias or imprecision. Discuss both direction and magnitude of any potential bias	18
Interpretation	20	Give a cautious overall interpretation of results considering objectives, limitations, multiplicity of analyses, results from similar studies, and other relevant evidence	17-19
Generalisability	21	Discuss the generalisability (external validity) of the study results	18-19
**Other information**			
Funding	22	Give the source of funding and the role of the funders for the present study and, if applicable, for the original study on which the present article is based	20

*Give information separately for exposed and unexposed groups.

Note: An Explanation and Elaboration article discusses each checklist item and gives methodological background and published examples of transparent reporting. The STROBE checklist is best used in conjunction with this article (freely available on the Web sites of PLoS Medicine at http://www.plosmedicine.org/, Annals of Internal Medicine at http://www.annals.org/, and Epidemiology at http://www.epidem.com/). Information on the STROBE Initiative is available at http://www.strobe-statement.org.

For peer review only

For peer review only - http://bmjopen.bmj.com/site/about/guidelines.xhtml
